# A novel H2A-A127 variant is associated with human cancer and enhances tumor-related phenotypes in *Drosophila melanogaster* models

**DOI:** 10.3389/fonc.2026.1814908

**Published:** 2026-07-13

**Authors:** Zeinab AlHajj Hassan, Hassan Dakik, Patricia Arreba-Tutusaus, Felice Frey, Sarah Mantash, Amanda Mitchell, Meaghan Boileau, Sagi Abelson, Malak Kleit, Zahraa Hayek, Jana Awada, Roy El Darzi, Kolja Eppert, Margret Shirinian

**Affiliations:** 1 Department of Experimental Pathology, Immunology and Microbiology, Faculty of Medicine, American University of Beirut, Beirut, Lebanon; 2 Department of Pediatrics, McGill University, Montreal, QC, Canada; 3 Research Institute of the McGill University Health Centre, Montreal, QC, Canada; 4 Department of Oncology, Hematology, Clinical Immunology, and Rheumatology, University Hospital Tübingen, Tübingen, Germany; 5 Princess Margaret Cancer Centre, University Health Network, Toronto, ON, Canada; 6 Department of Pediatric Oncology, Dana-Farber Cancer Institute, Boston Children’s Hospital and Harvard Medical School, Boston, MA, United States; 7 Ontario Institute for Cancer Research, Toronto, ON, Canada; 8 Department of Molecular Genetics, University of Toronto, Toronto, ON, Canada

**Keywords:** acute myeloid leukemia, *Drosophila melanogaster*, histone gene duplication, histone H2A, oncohistones, histone H2A-A127V

## Abstract

**Introduction:**

Acute Myeloid Leukemia (AML) is characterized by genetic and epigenetic dysregulation in myeloid progenitor cells. Histone H3 mutations disrupt chromatin and transcription, contributing to disease, but identifying mutations in histone genes is challenging due to sequence duplication. In particular, histone H2A genes H2AC18 and H2AC19, which share identical sequences, have been largely overlooked.

**Methods:**

Cancer genomic datasets were interrogated to identify novel variants, and *in vivo* functional assays were conducted using Drosophila melanogaster tumor models.

**Results:**

a recurrent Alanine to Valine substitution at position 127 (H2A-A127V) was identified in AML and also found in solid tumors, suggesting a broader cancer association. Using a Drosophila melanogaster model, H2A-A127V was shown to promote eye tumor phenotypes and, in the eyeful tumor model, intensified tissue overgrowth. The variant also interacts with the Enhancer of zeste (E(z)), a PRC2 complex member. *E(z)* downregulation in the presence of H2A-A127V enhanced ectopic growth, suggesting PRC2’s role in disease progression. Analysis of the 1000 Genomes Project revealed that H2A-A127V is an uncommon polymorphism (1.44%), potentially predisposing carriers to cancer.

**Discussion:**

These findings uncover a novel cancer-associated histone variant and emphasize the need to consider duplicated histone genes in mutation analyses to better understand cancer development.

## Introduction

Over the last decade, advances in next-generation sequencing (NGS) technologies have enabled the identification of novel oncogenic mutations, including a large panel of alterations in genes coding for epigenetic regulators, including histones (1). These aberrations are common across various cancer types and, in many cases, serve as the initiating event leading to cancer, particularly in acute myeloid leukemia (AML) (1, 2). Canonical histones are represented by a large panel of non-allelic paralogues that share remarkable identity in their primary sequence and this can thwart the detection of oncogenic mutations, resulting in the incomplete understanding of oncogenesis (3).

Oncohistones harboring mutations in the tail of histone H3.1 and H3.3 variants (Lysine 27 to Methionine/Isoleucine substitution K27M/I and Glycine 34 to Arginine/Valine substitution G34R/V) were first identified in aggressive childhood glioma (4, 5). We and others later reported histone H3 mutations (K27M/I) in acute myeloid leukemia (AML) and found that they increase disease aggressivity (6, 7). We also found that H3-K27M can exist in preleukemia and may contribute to leukemogenesis by expanding preleukemic hematopoietic stem cells (HSCs) and skewing myeloid differentiation with erythroid blockage (6). These mutations also increased hemocyte numbers in *Drosophila melanogaster* larvae, demonstrating that they affect fundamental hematopoietic processes and are conserved throughout development (6). Recently, an in-depth pan-cancer analysis using cBioPortal expanded the repertoire of oncogenic histone H2A and H2B and H3 mutations beyond tail marks to include missense mutations in the core/globular regions, including in histone H2B variants (8–10). Despite the power and widespread use of NGS technologies, a common shortcoming is the inefficient handling of reads that align to multiple genomic locations, named multi-mapped reads, especially in short-read sequencing (11–13). A comprehensive examination of multiple sequencing platforms and pipelines revealed that hundreds of genes with repetitive or shared sequences are consistently affected by this technical issue (14). NGS multi-mapped reads are often discarded by mutation-calling algorithms, leading to the underrepresentation of key coding regions and significantly affecting the detection of mutations in duplicated or repetitive regions. Additionally, multi-mapped reads also result in the underestimation of read counts for RNA-seq expression analysis (13).

Gene duplication occurs when one gene gives rise to another functionally indistinguishable paralogue. It is one of the mechanisms driving molecular evolution by providing new genetic material for the selection of novel specialized functions (15, 16). Canonical histones are among the most conserved genes across eukaryote species and are represented by a large panel of non-allelic paralogues, termed histone variants, with substantial sequence identity (3). While mutations in the histone H2A family have been found in cancer (8), none were previously reported for the H2AC18 and H2AC19 variants. These are two duplicated H2A genes with identical sequences located ~ 9kb apart on chromosome 1. The H2AC18 and H2AC19 genes were among a list of 958 genes containing multi-mapped reads that are handled poorly by alignment algorithms and their expression in RNA-seq datasets was shown to be severely underestimated by various aligners (14). We therefore hypothesized that point mutations and variations in the two identical H2AC18/H2AC19 genes are overlooked due to the elimination of multi-mapped reads.

Here, we identified a novel, recurring Alanine to Valine substitution at position 127 (A127V) in H2AC18/H2AC19 in ~5-8% of AML samples, as well as other H2A A127 variants that occur somatically in solid tumors. Using a well-established *Drosophila* eye tumor model (17), we confirmed that this variant enhances the tumor eye phenotype. Specifically, we found that expression of Human H2AC18/19-A127V, confers a survival disadvantage and alters behavior in a non-tumor sensitized background in *Drosophila*, while also exacerbating eye phenotypes and promoting tissue overgrowth in tumorous eyeful flies *in vivo*.

## Materials and methods

### Analysis of public datasets

#### Leucegene AML dataset

Unprocessed paired-ended fastq files (2 x 100-bp libraries) from Leucegene’s RNA-seq dataset were downloaded from NCBI’s Sequence Read Archive (SRA), using the following accession numbers: PRJNA263397 (82 samples), PRJNA214592 (43 samples), PRJNA229548 (27 samples), PRJNA278364 (22 samples) and PRJNA278767 (263 samples). Most Leucegene samples have multiple sequencing runs, and only one was used to represent each sample. The data were processed on Compute Canada clusters using the RNA-seq module of Genpipes (18). Raw reads were trimmed using Trimmomatic (19) v0.36 to remove Illumina adaptors and sequencing-primer associated reads, then aligned to GRCh38/hg38 using STAR with default parameters. PCR duplicate reads were then collapsed by Picard (v2.9). Aligned sequences (Bam files) were inspected for SNPs in H2AC18 and H2AC19 genes using the Integrative Genome Viewer software (IGV - v2.11.2) (20).

#### Validation AML dataset

Raw sequencing data for the validation dataset are publicly available under NCBI BioProject PRJNA820017 (21). Targeted DNA sequencing of the 3’ end of H2AC18/H2AC19 genes was conducted on the Illumina MiSeq platform, as described previously (6), using the following specific primers: GCGTCTTGCCTAACATCCAG and CAGCTCCAGGTTCGCTATTC.

#### The 1000-genomes project

Aligned bam files from the 1000-genomes project (GRCh37/hg19) were readily accessible from the server within the IGV software (v2.11.2) (20).

### Fly husbandry and handling

ey-Gal4; UAS-DL, GS88A8/CyO and ey-Gal4, UAS-DL/CyO (22) were kindly provided by Dr. Bassem Hassan. w; UAS H3.3 K27 M was kindly provided by Dr. Nada Jabado (17). Flag HIST1 H2A A4A 127V/Tm3Sb and HIST1 H2A A4A wt/Tm3Sb (This study). The wild-type control *w^1118^* was obtained from Bloomington Drosophila Stock Center (BDSC). Flies were raised on the standard cornmeal-agar-yeast medium at 25 °C, and crosses were generated at 25 °C and 29 °C depending on the experimental context. Female virgins were detected by the presence of meconium, a black dot in their abdomen. RNAi fly lines used: *ADD1* RNAi (VDRC 104095, 12739), *Asx* RNAi (VDRC 330440), *dTet* RNAi (VDRC 102273), *lozenge* RNAi (VDRC 330539), *Nlp* RNAi (VDRC 22623), *p53* RNAi (VDRC 10692), *gfp* RNAi (Dr. Martin Hasselblatt), *Kdm2* RNAi (BDSC 33699), *Pc* RNAi (BDSC 36070), *E(z)* RNAi (BDSC 27993), *Jarid2* RNAi (BDSC 32891), *Su(z)12* RNAi (BDSC 33402).

The tumor model eyeful flies overexpress Delta, ligand of the Notch signaling, in addition to the overexpression of two Polycomb group epigenetic silencers lola and pipsqueak. Deregulation of *lola and psq (pipsqueak)* refers to the use of previously characterized insertional mutations. The use of the Delta/Notch system in our sensitized eyeless fly line is not intended to imply a direct link between AML and Delta/Notch signaling. Rather, this system provides a sensitized background to enhance phenotypic detection, allowing us to amplify subtle effects of the histone variants and quantify phenotypes more effectively. It is worth noting that in these RNAi screening experiments, the *gfp* RNAi control flies show slight invagination in some flies which is considered an eye fold phenotype.

### Transgenic fly generation

Human H2A A4A wt and HIST1 H2A A4A 127V were inserted into fly cloning and transformation vector pUASTattB (23) (Custom DNA Constructs). Flag tag was introduced at the N-terminus to allow the detection of the expression of the constructs. Transgenic flies carrying transgene on the third chromosome were generated by BestGene using BDSC_8622 stock.

### Phenotypes scoring

Eye phenotypes and macroscopic metastasis were examined using light microscopy. The scoring scale used for the eyeful screen was as follows: two broad classifications of eye phenotypes: 1- eye folds, where eyes are considered tumorous “hyperplastic” if they show at least one-fold in the tissue, and they are scored individually. 2- metastasis is defined as amorphous red tissue spread in locations distinct from the eye, and they are counted as incidences. The eye phenotype criterion is further categorized into three divisions based on the extent of the severity of the folds: minor (enlarged eyes with no folds), moderate (1–4 folds), and major (>4 folds) severity. Samples were collected from more than 3 biological replicates. Representative images were taken using Olympus microscope and were exported using cellSens software. These light microscopy images were further processed using ImageJ.

### *Drosophila* activity monitor

The *Drosophila* activity monitor (Trikinetics Inc., Waltham, MA, USA) system was used to record and compare the activity and sleep data. Because female flies are prone to laying eggs, which might interfere with the infrared beam detection and result in erroneous activity and sleep data, previous research has shown that the Drosophila Activity Monitor (DAM) system is predominantly used with male flies. One day old male flies were loaded individually into glass activity tubes (5 mm × 65 mm) containing sucrose (5%) and agar (1%) media and sealed with paraffin to avoid dehydration throughout the 30 days analysis (24). The tubes were then placed in monitors equipped with individual channels with an infrared light beam to detect the movement of the flies when interrupted. The DAM system was maintained under a 12/12 h light/dark cycle at a constant humidity (80%) and temperature (25 °C). Trikinetics data acquisition software (DAMSystem308, Trikinetics Inc.) saves the activity data of each fly as channel counts per 5 min. The acquired data can be processed to quantify locomotor activity, sleep duration, and survival of the flies while considering a 5 min of immobility as sleep and >24 h as death as previously reported. The analyzed activity and sleep acquisitions were calculated for each day of the experiment as an average from the data of all living flies at this time and then displayed throughout the experiment (25, 26). Calculation and statistical analysis of data were performed using Microsoft Excel and Graphpad Prism (Graphpad Software, La Jolla, CA, USA).

The Drosophila Activity Monitor (DAM) assay is typically conducted using male flies, as female egg-laying behavior can interfere with accurate measurements of activity and sleep. In our experiments, we analyzed 32 male flies per genotype, which falls within the standard range for DAM-based behavioral assays (24, 27). For the H3.3 experiments, only 15 flies were analyzed due to limited availability of the specific transgenic lines. Nevertheless, this sample size is consistent with prior studies that have successfully detected robust phenotypic differences under similar experimental conditions.

DAM experiments are routinely terminated at day 30, and the genetic background of the control may influence survival. In our experiments, all flies were dead by 30 days, which could be inferred from our sensitized control that expresses Delta ligand in the eye, which may itself alter lifespan, explaining the observed differences with other survival profiles in other studies.

### Western blot

For each sample, 30 adult fly heads were put in a total volume of 30µL of 2x Laemmli buffer containing 4% protease inhibitor. Head tissues were homogenized by a pestle tip for 3 min, then the tip was cleaned with an additional 10 µL of the Laemmli buffer. Samples were then sonicated for 15 min at 4 °C and then kept on ice for 10 min before centrifugation for another 15 min at 14,000 g. The supernatant was collected followed by heating at 95 Celsius for 10 min. The protein concentration was measured using a Nanodrop Spectrophotometer, and then a final 6% beta-mercaptoethanol was added. Samples were loaded onto 12% acrylamide gel and were run with the Color-coded Prestained Protein Marker, Broad Range (11–250 kDa) #14208 as a molecular weight marker at 70 V till the wells were empty then switched at 100 V until the run is complete. Next, the gel was removed from the running apparatus and placed onto a Nitrocellulose membrane of a pore size of 0.2 mm for optimal retention of histone proteins and into a transferring apparatus. Transfer was done at 4 °C, at 100 V for 10 min then at 60 V for 30 min. The membrane was then soaked in Ponceau, washed, then placed in blocking milk solution (5% milk in PBS-Tween 0.05%) for 1 h. Following, the nitrocellulose membrane was incubated in blocking solution containing primary antibody (anti-Ub-H2A, Cell signaling technology 8240S, 1:2000; anti-H3K27me3, C36B11, 1:1000; anti-β actin (rabbit), Abcam ab8227, 1:2000) overnight. Three washes with PBS-Tween for 10 min each were done, and then the membrane was incubated in a blocking solution containing HRP-conjugated secondary antibody (ECL Rb HRP-NA934 IgG (donkey), 1:5000) at room temperature for 2 h. The membrane was washed three times with PBS-Tween for 10 min each. The bands were imaged using the Chemidoc MP machine using ECL Clarity Max (Biorad). Band intensities were normalized to loading controls and averaged across three independent Western blot replicates using Fiji.

### Real-time reverse transcription polymerase chain reaction

#### Eye extraction and tissue collection

Whole eyes were extracted immediately after sacrifice to prepare the sample for subsequent RT-qPCR analysis. Each adult Drosophila eye was removed using sterile tools while submerged in PBS. 12 to 15 eyes were dissected from three separate biological replicates for each gene RNAi knockdown. The extracted eyes were then immersed in PBS and frozen for preservation.

#### RNA extraction and cDNA synthesis

RNA Extraction: RNA was extracted using the TRI Reagent (Sigma-Aldrich). 1000 µL of Trizol was added to the samples, followed by manual homogenization with a syringe. Samples were incubated at room temperature for 5 min, then Chloroform (Sigma-Aldrich) was subsequently added, and samples were vortexed and then centrifuged at 21,000 g for 10 min at 4 °C. The supernatant containing RNA was collected in a new tube and cold isopropanol was added. Samples were kept at room temperature for 10 min to allow for maximum RNA precipitation before centrifugation at 21,000 g for 30 min at 4 °C. The RNA pellet was subsequently washed with 75% ethanol and centrifuged at maximum speed for 10 min each at 4 °C. The ethanol was discarded, and the pellet was air-dried on ice before being resuspended in nuclease-free water. Extracted RNA was stored at -80 °C for long-term preservation, and working aliquots were stored at -20 °C.

RNA Purity Assessment: All samples, control and treated, were then measured to calculate their concentrations (ng/ul) and to assess RNA purity using a spectrophotometer or Nanodrop. Samples with A260/A280 >1.8 and A260/A230 > 2 fulfilled the purity criteria since they were free of DNA, protein, and chemical contamination.

cDNA Synthesis: Following RNA extraction and purity assessment, all RNA samples were converted to cDNA using iScript™ cDNA Synthesis Kit (Bio-Rad), which included Reaction mix, Reverse Transcriptase, and nuclease-free water. According to the BioRad kit cDNA kit protocol, RNA volumes of 1 ul were pipetted from each diluted RNA sample and mixed with 4 ul of reaction mix, 1 ul of Reverse transcriptase, and 14 ul of nuclease-free water to reach a total of 20 ul solution in each cDNA tube. All cDNA tubes were run on a thermal cycler for 35 minutes using the standard iScript protocol. The resulting cDNA was then stored at 20 °C for short-term or -80 °C for long-term.

#### Quantitative reverse transcriptase PCR protocol and analysis

For each primer, the reaction was carried out using a total volume of 10 μL, composed of 9 μL master mix (SYBR Green, forward and reverse primers, and nuclease-free water) and 1 μL of target cDNA template. Negative controls consisted of 9 ul master mix plus 1 ul of nuclease-free water instead of cDNA. After all the samples were prepared, gene amplifications were performed on the Bio-Rad CFX PCR machine using the standard SYBR Green protocol, which is divided into an initial denaturation and enzyme activation at 95 °C, and Forward and reverse Primers (**Table 1**) repetitive cycles of denaturation at 95 °C, annealing phase at 55-60 °C, extension phase at 72 °C, and concluded with melt curve analysis to confirm product specificity. All reactions were conducted in technical replicates, and Cq values were averaged after checking replicate consistency. Negative controls were studied to ensure the absence of amplification. The ΔCq method was used to normalize our target genes to the housekeeping RpL11 gene to compare gene expression in the sample with its internal control (RpL11). Then, ΔΔCt (Delta–Delta Ct) values were calculated relative to control values to determine fold changes in gene expression. Only assays showing single peak melt curves, no primer dimer formation, and acceptable efficiency were included in the final analysis.

**Table 1:** Primers used for quantitative RT-PCR validation.

	Forward primer (5’ to 3’)	Reverse primer (5’ to 3’)
Jarid2 (1)	GTCAAAAGGCCAAGAAGCAG	TTGCAGGCAGTAAATGTTGC
Ez (1)	CAGCAAGGAACTGGAGGAAG	ATCATCTTCGCCCTGTTTTG
dTet	CCGGTGTGTTCTGTGGTATCTG	ATGAAGGATCAACCGCAGCAG
kdm2 (2)	GTCCAAATGCAAAAGGCGTG	AGATTCGAGCTTCTCGGCAAC
nlp	TGGGATGTGGACGAGGACTA	CTCTCCGGCCTTCAATACGG
Pc	TCAAGTGGAAGGGCTGGAAC	CGATGAGGTGGTGGCTTGAT
P53 (3)	GCCGCCTCCTTAATCATGCC	GCCGAGACTGCGACGACTC
Add1 (1)	AATAAGCAGTGGCGGGTGAT	GTCATCTTCGCCACAGTCCA
Add1 (2)	AATAAGCAGTGGCGGGTGAT	GTCATCTTCGCCACAGTCCA
RpL (11)	GGGTATTCGCCGTAACGAGA	CCACGCTCCAGAATCTCCTC

### Statistical analysis

All statistical calculations were conducted using Graphpad Prism. For phenotype scoring, Chi-square test was used to calculate the statistical significance between the assigned groups. For DAM data and western blot quantification, a two-tailed student t-test was used to determine the significance of sleep, fragmented sleep, and activity. Survival data analysis was done by using the Log-rank test. A P-value greater than 0.05 was considered non-significant. P-values of all phenotypic screenings are present in **Supplementary Table 1**.

For RT-qPCR, data were first assessed for normality using the Shapiro–Wilk test and for homogeneity of variances using Levene’s test. Unpaired two-tailed Student’s t-tests were used to compare gene expression levels between *gfp* RNAi control samples and target gene RNAi knockdown samples. This statistical approach was applied to determine whether RNAi-mediated knockdown induced significant differences in gene expression between groups (p < 0.05). Data are presented as mean ± SEM, and outliers were identified and excluded using the GraphPad Prism outlier test.

## Results

### Novel A127V missense mutation in H2AC18/H2AC19 H2A histones identified in AML patient samples after accounting for multi-mapped reads

Given that H2AC18 and H2AC19 genes contain multi-mapped reads that can mask nucleotide variants, we examined whether variants are detectable in summarized sequencing data from large public cohorts of normal and cancer samples. H2AC18 and H2AC19 are duplicated genes with identical nucleotide and protein sequences and so we will hereafter refer to them as H2AC18/19. gnomAD v2.1.1 (28), a large allele frequency database spanning 125,748 exome sequences and 15,708 whole-genome sequences of diverse ancestries, did not list any single nucleotide polymorphism (SNP) variants in H2AC18/19. In contrast, a panel of 13 other histone H2A variants, collectively accounted for 11959 SNPs. Similar results were found for gnomAD v3.1.1 database (28), which comprises 76,156 genomes. We also found that H2AC18/19 mutations are absent from the Mutation Annotation Format (MAF) files of a curated list of 105,260 cancer samples from 226 different studies on cBioportal (9). These data suggest two possibilities: that variants in H2AC18/19 do not exist or that they are not recognized, potentially due to the elimination of multi-mapped reads from upstream analysis.

To address the possibility that variants in H2AC18/19 in cancer samples are missed due to standard analysis pipelines, we examined unprocessed raw sequencing files from Leucegene’s RNA-seq dataset, which includes a diverse panel of 437 AML samples (29, 30) (**Table 2**). After alignment, instead of using mutation callers that subject aligned reads to multi-mapping filtering, we inspected the samples for H2AC18/19 mutations using the integrative genome viewer (IGV) (31). Analysis of these reads uncovered a recurring A127V missense variant in 5.03% of AML samples (**Table 2**; **Supplementary Data Sheet 1**). To validate this result, we examined an independent RNA-seq dataset of 62 AML samples (NCBI BioProject PRJNA820017) (21), consisting of a mix of bone marrow and peripheral blood specimens. We found that 5 patients (~8%) carry the H2AC18/19 A127V mutation. This was confirmed by sequencing of genomic DNA extracts using the Illumina MiSeq platform (**Supplementary Figure 1**), where mononuclear cells were isolated using Ficoll purification, and DNA was extracted from bulk, unsorted samples (without blast cell enrichment). This revealed variant allele frequencies (VAFs) of 0.2-0.27 (**Supplementary Figure 1**). Assuming heterozygosity, this indicates that virtually all cells (80-100%) in those samples possess one mutant A127V allele and three wild-type alleles for the combination of H2AC18 and H2AC19. Overall, these findings establish that the H2AC18/19 genes harbor previously unidentified missense variants in AML.

**Table 2:** Leucegene RNA-seq Dataset of 437 samples from AML patients used to identify H2AC18/19 mutation expression in AML.

Leucegene dataset	SRA accession numbers	Total number of samples	Samples with H2AA4-A127V
GSE62190	PRJNA263397	82 samples	4 samples
GSE49642	PRJNA214592	43 samples	4 samples
GSE52656	PRJNA229548	27 samples	2 samples
GSE66917	PRJNA278364	22 samples	1 sample
GSE67039	PRJNA278767	263 samples	11 samples
Total		437 samples	22 samples

### H2A A127 variants occurs somatically in human cancer

To determine if histone H2A A127 mutations also occur in solid tumors, we examined pan-cancer WES data from cBioPortal for missense mutations in H2A histones at the same amino-acid position as H2AC18/19-A127. Multisequence alignment revealed that 15 H2A histone variants possess A127-equivalent amino acids (**Supplementary Figure 2**). We identified ten cancer patients carrying variants at A127 (or equivalent positions) in six H2A histones (**Table 3**). Interestingly, 50% of these patients possess Diffuse Large B-Cell Lymphomas (3 cases) or Chronic Lymphocytic Leukemias/Small Lymphocytic Lymphomas (2 cases), despite both malignancies only constituting 3.46% of the queried cancer sample (Fisher test: OR = 27.9; p-value=0.00001083). This suggests that A127 and its equivalent variants may contribute to the development of these two mature B-cell lymphoid malignancies. Notably, the H2A A127 sequence was wild-type in the adjutant normal tissues of four out of the five patients with matched WES data, indicating that the H2A A127 variants are cancer-associated mutations (**Table 3**). Thus H2A-A127 mutations can be somatic alterations acquired during disease development or progression.

**Table 3:** A summary of cBioPortal samples with A127 equivalent mutations in other H2A variants.

									Tumor tissue			Normal tissue		
Gene	Sample ID	Cancer type detailed	Chr.	Start pos	End pos	Ref	Var	Protein change	Variant reads	Ref reads	VAF	Variant reads	Ref reads	VAF
H2AC1	IC-9635EPN	Ependymoma (Glioma)	6	25726374	25726374	C	A	A128S	25	22	0.53	25	22	0.53
H2AC4	TCGA-AA-3492-01	Colon Adenocarcinoma	6	26033417	26033417	G	A	A127V	32	33	0.49	0	52	0
H2AC15	CSCC-32-T	Cutaneous Squamous Cell Carcinoma	6	27805739	27805739	C	A	A127S	31	125	0.2	0	178	0
H2AC17	cll_iuopa_2015_464	Chronic Lymphocytic Leukemia/Small Lymphocytic Lymphoma	6	27860548	27860548	G	C	A127G	–	–	–	–	–	–
H2AC17	DLBCL-LS2357	Diffuse Large B-Cell Lymphoma, NOS	6	27860548	27860548	G	A	A127V	12	111	0.1	–	–	–
H2AC17	SP116697	Diffuse Large B-Cell Lymphoma, NOS	6	27860549	27860549	C	T	A127T	4	30	0.12	–	–	–
H2AC17	PCNSL_3	Diffuse Large B-Cell Lymphoma, NOS	6	27860548	27860548	G	A	A127V	–	–	–	–	–	–
H2AC17	SCLL-0363*	Chronic Lymphocytic Leukemia/Small Lymphocytic Lymphoma	6	27860548	27860548	G	C	A127G	55	46	0.54	0	77	0
H2AC17	SCLL-0363*	Chronic Lymphocytic Leukemia/Small Lymphocytic Lymphoma	6	27860549	27860549	C	T	A127T	56	49	0.53	0	80	0
H2AC20	CLCA_0273	Hepatocellular Carcinoma	1	149858901	149858901	C	T	A126V	–	–	–	–	–	–
H2AC25	TCGA-23-1116-01	Serous Ovarian Cancer	1	228645139	228645139	G	T	A127D	125	49	0.72	0	60	0

VAF, variant allele frequency. *Two substitutions at the same codon are present in this sample.

### H2AC18/19-A127V is an uncommon, cancer-associated polymorphism

We next examined the possibility that the H2A-A127V alteration is more frequent in hypermutator phenotype by assessing the mutation status of common oncogenes (including IDH1/2, NPM1, FLT3ITD and TP53) as well as cytogenetic risk groups and normal vs abnormal karyotype in the Leucegene dataset. There was no correlation and, thus, the presence of the mutation does not correlate with a hypermutator phenotype (**Supplementary Table 2**).

To determine if H2AC18/19-A127V also occurred in the general population, we inspected aligned reads from the 1000 Genomes Project (2565 samples of 26 different ancestries) [1] using the integrative genomics viewer (IGV). Our analysis revealed that H2AC18/19-A127V is a previously unknown polymorphism with a frequency of 1.44% (**Supplementary Table 3**, **Supplementary Data Sheet 2**). A127V was enriched in people of European and Americas origin compared to other ancestries (OR = 14.29 and p-value <0.001). The somatic occurrence of the A127 variants combined with our functional data suggests that it might be a cancer predisposing polymorphism in these individuals.

### H2AC19 (HIST2H2AA4) A127V variant enhances tumor progression

We next examined the evolutionary conservation of H2AC19 across species. Our alignment results showed that the alanine residue identified in human H2AC19 is conserved, although the exact position is 1–3 aa different in yeast and *Drosophila* (**Supplementary Figure 3**). We sought to overexpress the human H2AC19 variants in *Drosophila* to assess transformation phenotypes *in vivo* as the conservation of core histone structure and chromatin regulators permits functional interrogation of human proteins in this model.

**Figure 1:**
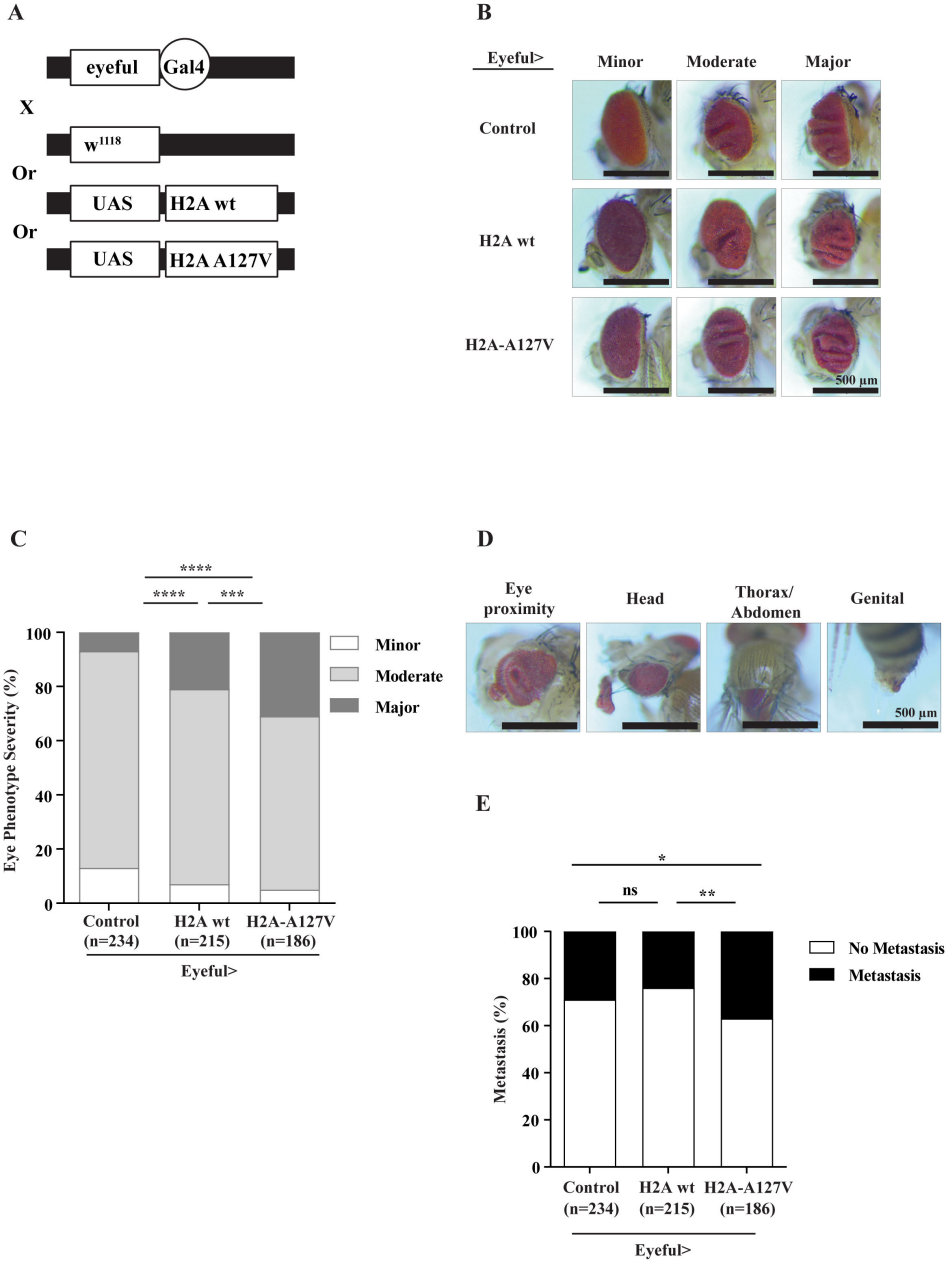
H2A-127V increases the severity of the eye tumor phenotype. **(A)** A schematic indicating the flies used in expressing H2A wt and H2A-127V using the eyeful promoter. **(B)** Light microscopy images showing the three categories of observed eye phenotype: minor (enlarged eyes with no folds), moderate (1–4 folds), and major (>4 folds) severity, at 25 °C with wt or H2A-A127V mutant transgene. Scale bar, 500µm. **(C)** Scoring the severity of eye phenotype (%) for each corresponding genotype, at 25 °C (***, p-value <0.001 and ****, p-value <0.0001; chi-square test). **(D)** Light microscopy images of the four locations of observed metastasis in the three genotypes. Scale bar, 500µm. **(E)** Scoring the percentage of metastasis incidence for each corresponding genotype, at 25 °C (*, p-value <0.05 and **, p-value <0.01; chi-square test). Flies were collected from more than 3 biological replicates.

To assess the functional impact of the human H2AC19 A127V variant in cancer, we used the well-studied *Drosophila* eye tumor models: ‘sensitized’ and ‘eyeful’ flies. The sensitized flies overexpress the Delta ligand of the Notch pathway driven by eye promotor *eyeless*, which induces an enlarged eye phenotype and not a tumor phenotype. Eyeful flies comprise the same genetic background of Delta overexpression, in addition to dysregulation of two additional genes; *lola* and *psq (pipsqueak)* due to insertional mutations (22, 32). *lola* and *psq* mutations confer tumorigenic potential by disrupting epigenetic pathways. Deregulation of *lola* and *psq (pipsqueak)* lead to transcriptional upregulation of both genes and confer tumorigenic potential by disrupting epigenetic regulatory pathways, as previously reported (22). This model is particularly valuable for investigating potential polymorphisms, as it provides differing genetic backgrounds to assess the effects of naturally benign SNPs in the context of a non-tumorous sensitized background compared to a tumorous one. The tumor phenotype in eyeful flies is represented by folds and roughness in the eye tissue, in addition to metastatic tissue in 3% of the flies. Therefore, using these tools, we can screen for mutations that interact with delta overexpression in both non-tumor and tumor contexts, and that may either enhance or reduce the inherent phenotypes in these eye models (22, 32). We therefore used two broad classifications of eye phenotypes: the first is eye folds, where eyes are considered tumorous “hyperplastic” if they show at least one-fold in the tissue, and they are scored individually. The second is metastasis which is defined as amorphous red tissue spread in locations distinct from the eye, and they are counted as incidences. To assess whether H2A-A127V can drive progression of a pre-established tumor, we overexpressed H2AC19 wild type and A127V mutant in the eye tissue using the eyeful tumorous model (**Figure 1A**). The eye phenotype criterion is further categorized into three divisions based on the extent of the severity of the folds: minor, moderate, and major (**Figure 1B**). Eyeful flies expressing the H2A-A127V mutation developed significant major eye severity in 31% of the flies’ eyes compared to 21% for H2A wild type (p-value=0.0005) and 7% for control (*w^1118^* flies crossed to the eyeful background) (p-value <0.0001) (**Figure 1C**). Metastatic phenotypes were recorded at 5 distinct locations: the proximity of the eye, head, thorax, abdomen, and noticeably in the genital area of one H2A-A127V female fly (**Figure 1D**). Similarly, the H2A-A127Vs conferred a significantly higher rate of metastasis incidences (37%) in comparison to the wild-type (24%, p-value=0.0070) and the control (29%, p-value=0.0311) (**Figure 1E**). This data demonstrates that the H2A-A127V mutant promotes tumor development in the eyeful model.

### H2A-A127V variant interacts with knockdown of AML-related genes

**Figure 2:**
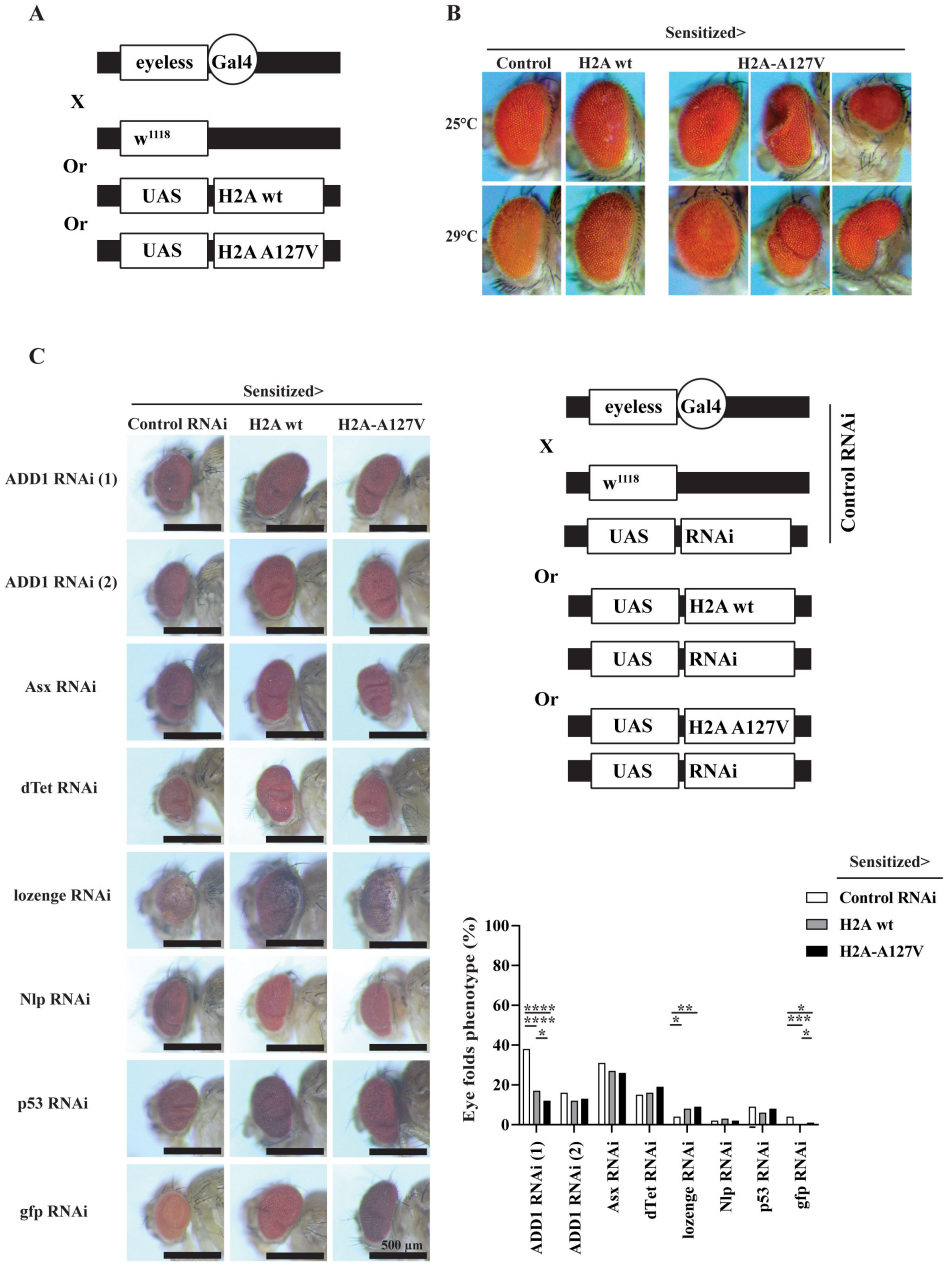
Screening of AML-related genes in the context of H2A-A127V mutation reveals deleterious or rescuing phenotypic changes. **(A)** A schematic indicating the flies used in expressing H2A wt and H2A-127V using the eyeless (sensitized) promoter. **(B)** H2A A 127V induces partial eye phenotypes in the sensitized model (almost all flies had normal eyes, and only 1 out of 100 at 25 °C and 2 out of 100 at 29 °C displayed a more severe phenotype). Light microscopy images of eyes in control, H2A wt, and the H2A-A127V mutants. Three different phenotypes were observed with H2A-A127V varying between small, rough, and tumorous eyes. Upper Panel experiment was performed at 25 °C screening 200 flies and the lower panel experiment was performed at 29 °C screening 100 flies. **(C)** Light microscopy images of eye phenotypes in H2A wt and H2A-A127V mutants crossed with RNAi lines targeting AML-related genes (n is number of flies screened): *ADD1* (1) (Control RNAi n=165, H2A wt n=205, H2A-A127V n=267), *ADD1* (2) (Control RNAi n=183, H2A wt n=199, H2A-A127V n=220), *Asx* (Control RNAi n=182, H2A wt n=201, H2A-A127V n=187), *dTet* (Control RNAi n=200, H2A wt n=229, H2A-A127V n=252), *lozenge* (Control RNAi n=237, H2A wt n=204, H2A-A127V n=200), *Nlp* (Control RNAi n=201, H2A wt n=200, H2A-A127V n=246) and *p53* (Control RNAi n=202, H2A wt n=208, H2A-A127V n=207) in a sensitized model. A schematic illustrating the rescue experiments are indicated. Control RNAi was expressed alone in the eye by the sensitized driver that provided the baseline phenotype provoked by the RNAi itself. gfp RNAi is a control for the RNAi system (Control RNAi n=197, H2A wt n=256, H2A-A127V n=214). Scoring eye fold phenotypes (%) in H2A wt (grey bars) and H2A-A127V mutant (black bars) eyes following knockdown of AML-related genes in comparison to Control RNAi (white bars) (*p-value <0.05, **p-value <0.01, ***p-value <0.001 and ****p-value <0.0001; chi-square test). Scale bar, 500µm. Flies were collected from more than 3 biological replicates.

Our previous data showed enhancement of the tumor incidence in the eyeful “tumor” model. To further assess whether H2A-A127V drives overgrowth and tumor phenotypes at baseline levels, we used the sensitized non-tumor eye model (**Figure 2A**). We found that almost all flies had normal eyes and only 1 out of 200 at 25 °C and 2 out of 200 at 29 °C displayed a more severe phenotype (**Figure 2B**). This indicates that the histone H2A-A127V mutation may display a limited oncogenic potential, transforming a sensitized background into more tumor context compared to the eyeful expression. For this reason, we utilized the sensitized non-tumorous model in the upcoming experiments to genetically combine the H2A-A127V mutants with other AML-inducing gene knockdowns, as well as genes involved in epigenetic regulation within the polycomb repressive complex PRC1 and PRC2 protein families. This sensitized non-tumorous model serves as an effective system for detecting even subtle changes in the relatively normal-looking eyes, to which the A127V mutation and other critical gene knockdowns are introduced.

Many common human genes that are mutated in AML (2, 33)(ASXL1 (34), TET2 (35), RUNX1 (36), NPM1 (37) and TP53 (38)) are highly conserved in the fly model; hence, we can take advantage of the UAS-Gal4 system to downregulate (RNAi) these homologs (*ADD1*, *Asx*, *dTet*, *lozenge*, *Nlp*, and *p53*) in the *in vivo* model (**Supplementary Table 4**). To test whether the H2A-A127V mutation combined with AML oncogenes could have a synergistic or antagonistic effect in a sensitized background, we generated a screening line using H2A transgenic lines (Sensitized > H2A wt and sensitized > H2A-A127V) and combined it with RNAi lines targeting AML-related genes (*ADD1*, *Asx*, *dTet*, *lozenge*, *Nlp*, and *p53*) and assessed the eye fold phenotype. Short hairpin RNA interference (shRNAi) for the genes to be tested in combination with H2A-A127V were expressed alone in the eye by the sensitized driver to provide a baseline phenotype that may be provoked by knocking down the gene alone. Screening lines were crossed with *gfp* RNAi as a control for the RNAi system. No significant effects of human histone H2A wt or H2A-A127V were observed compared to the gene knockdowns alone in case of *Asx*, *dTet*, *Nlp* and *p53* (**Figure 2C**). In the case of *ADD1* knockdown, which encodes a heterochromatin protein 1 interactor that associates with methylated H3K9 (39), knocking it down induced eye folds in 38.5% of flies’ eyes. However, when combined with H2A wild-type, the number of flies exhibiting eye folds is reduced (17.6%). This reduction becomes even more pronounced when *ADD1* knockdown is combined with the H2A-A127V mutant (12.9%). A comparison of H2A wt and H2A-A127V with *ADD1* knockdown reveals a significant reduction in the number of eyes with folds in A127V compared to H2A wt (p-value=0.0474) (**Figure 2C**) indicating rescuing effect with *ADD1* knockdown. On the contrary, in the case of *lozenge* knockdown, the eye phenotype percentage is significantly increased from 4.5% in *lozenge* shRNAi alone to 8% (p-value=0.0348) when combined with H2A wt and to 9% (p-value=0.0099) when combined with the H2A-A127V mutant. *lozenge*, which encodes an alpha-subunit of the transcription factor complex core binding factor, is involved in transcription regulation and plays important role during eye development. Knocking down *lozenge* alone results in a rough eye phenotype but doesn’t enhance the eye folds phenotype, as the percentage is not significant in comparison to the *gfp* RNAi alone control. Combining *lozenge* knockdown with the H2A wt or the H2A-A127V mutant form shows synergistic effect with respect to eye fold severity.

### H2A-A127V mutation produces deleterious phenotypes when combined with E(z) knock down

**Figure 3:**
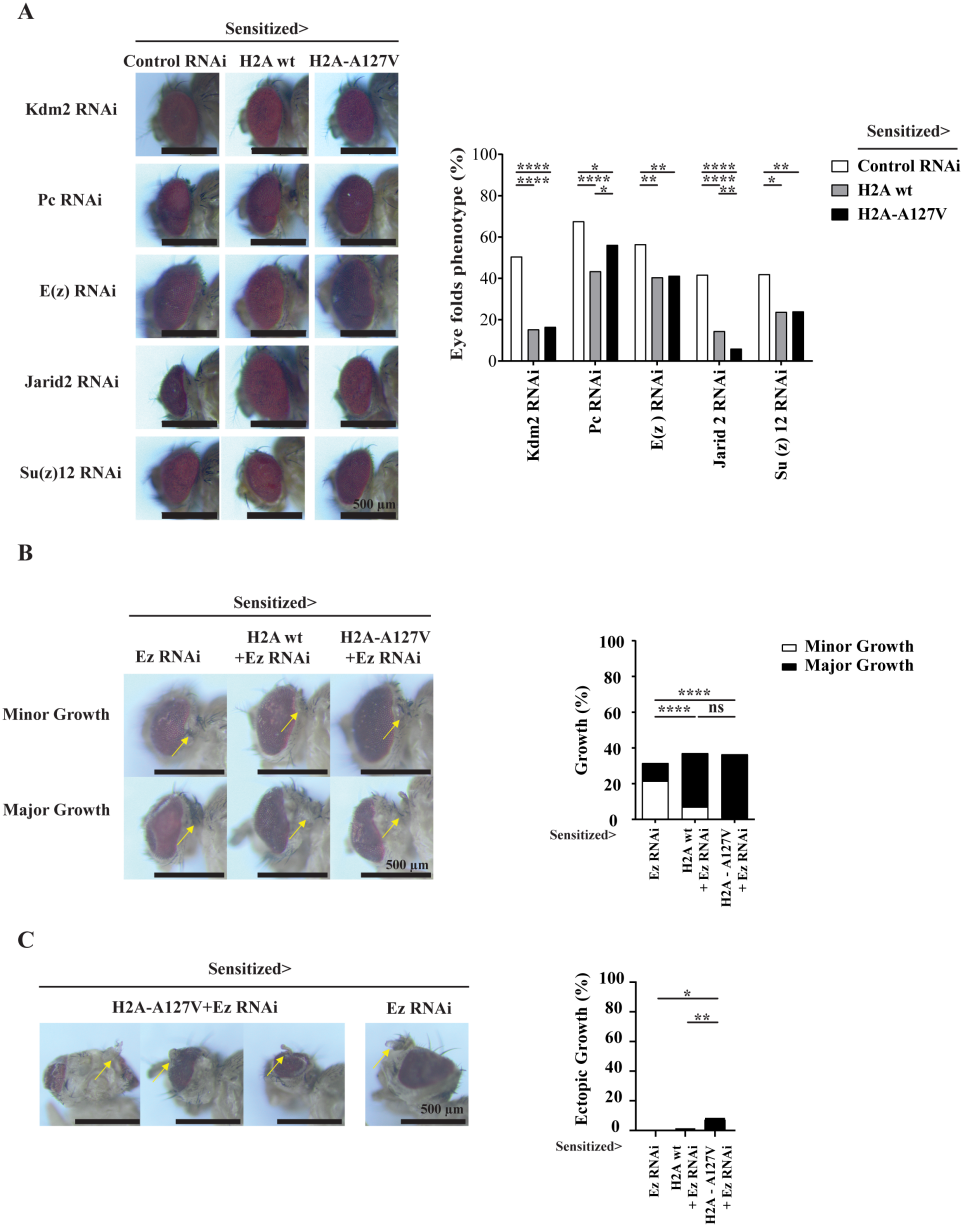
*E(z)* RNAi enhances eye growth and ectopic leg formation in H2A-A127V flies. **(A)** Light microscopy images of eye phenotypes in H2A wt and H2A-A127V mutants crossed with RNAi lines targeting PRC1/PRC2 components (n is number of flies screened): *Kdm2* (Control RNAi n=99, H2A wt n=62, H2A-A127V n=100), *Pc* (Control RNAi n=108, H2A wt n=92, H2A-A127V n=105), *E(z)* (Control RNAi n=100, H2A wt n=100, H2A-A127V n=103), *Jarid2* (Control RNAi n=134, H2A wt n=100, H2A-A127V n=100), and *Su(z)12* (Control RNAi n=50, H2A wt n=40, H2A-A127V n=50) in a sensitized model. Control RNAi was expressed alone in the eye by the sensitized driver that provided the baseline phenotype provoked by the RNAi itself. Scoring eye fold phenotypes (%) in H2A wt (grey bars) and H2A-A127V mutant (black bars) eyes after knockdown of PRC1 and PRC2 components in *Drosophila melanogaster* in comparison to Control RNAi (white bars) (*p-value <0.05, **p-value <0.01 and ****p-value <0.0001; chi-square test). Scale bar, 500µm. **(B)** Light microscopy images show two growth phenotypes in *E(z)* RNAi groups varying between minor (observed as small sprouts) and major (observed as big masses) growth that are strikingly protruding from the head. Scoring the percentage of major and minor growth for each corresponding genotype (****p-value <0.0001; chi-square test). Scale bar, 500µm. **(C)** Light microscopy images of the ectopic growth phenotypes in the eyes of (H2A-A127V + *E(z)* RNAi) and *E(z)* RNAi flies. Scoring the percentage of ectopic growth characterized by legs protruding from the antennae in (H2A-A127V + *E(z)* RNAi) and *E(z)* RNAi flies (*p-value <0.05 and **p-value <0.01; chi-square test). (*E(z)* RNAi n=100, H2A wt n=100, H2A-A127V n=103). Scale bar, 500µm. Flies were collected from more than 3 biological replicates.

Lysine residues in H2A undergo monoubiquitination mediated by the polycomb repressive complex 1 (PRC1), adding the ubiquitin group to K119 of H2A (40, 41). H2A-A127V lies in the proximity of the H2A mono-ubiquitination site. We therefore hypothesized that this mutation could affect H2A ubiquitination and, in turn, the expression of genes downstream. Similarly, the polycomb repressive complex 2 (PRC2) is recruited to trimethylate H3K27, further reinforcing the repressive chromatin state (41, 42). Flies expressing histone H2A-A127V mutant or wt with sensitized *eyeless* driver show preliminary reduced levels of H2A Lys119 Ubiquitin and H3K27 trimethyl in the adult heads compared to the control. To investigate whether the downregulation of PRC1 or PRC2 complex components, in combination with H2A mutation, could have a synergistic or antagonistic effect in a sensitized background, we employed a strategy similar to that used for the AML oncogenes. Specifically, we crossed screening H2A transgene lines (Sensitized > H2A wt and sensitized > H2A-A127V) with shRNAi lines targeting key PRC1 complex components (*Kdm2* and *Pc*) and PRC2 components (*E(z)*, *Jarid2*, *Su(z)12*) (**Supplementary Table 5**). The resulting eye fold phenotype was then assessed (**Figure 3A**). Knocking down components of the PRC1 and PRC2 resulted in enhanced eye fold phenotypes in the sensitized background. In the case of *Kdm2*, *E(z)* and *Su(z)12* shRNAi knockdown in the sensitized background, the combination with both the H2A-A127V mutant or with the H2A wt resulted in similar suppression of this eye fold phenotype (**Figure 3A**). Of particular interest was the knockdown of *Jarid2* (Jumonji, AT-rich interactive domain 2), which encodes a scaffold protein that recruits the Polycomb repressive complex 2 by binding mono-ubiquitylated H2A lysine 119 (43, 44). The H2A-A127V mutant rescued the enhanced eye phenotype resulted by *Jarid2* knockdown alone (p-value <0.0001), leading to a greater reduction in the percentage of eye fold phenotypes compared with the wild-type H2A in combination with *Jarid2* knockdown (p = 0.0051). We observe the contrary when knocking down *Pc*, which encodes a PRC1 component that interacts with histone H3K27me3. In this case, the wild-type H2A rescued the enhanced eye phenotype resulted by *Pc* knockdown alone (p-value <0.0001) better than the H2A-A127V mutant, leading to a greater reduction in the percentage of eye fold phenotypes compared with the H2A-A127V mutant in combination with *Pc* knockdown (p-value=0.0118).

The combination of both the H2A-A127V mutant and the H2A wt resulted in similar suppression of this eye fold phenotype conferred by *E(z)* shRNAi as mentioned above. In addition to the eye fold phenotype, *E(z)* knockdown (alone and in combination with H2A-A127V mutant or the H2A wt) led to the formation of a novel prominent growth that is protruding from the head in these flies, located behind individual eyes, here referred to as growth (**Figure 3B**). The growth phenotype was classified into two groups (minor and major growth) and was scored per individual eye. Flies expressing the H2A-A127V and *E(z)* shRNAi developed higher percentages of major growth (35.4%), slightly higher compared to flies expressing H2A wt with *E(z)* shRNAi (30%, p-value=0.2432) and significantly higher than flies expressing *E(z)* shRNAi alone (10%, p-value <0.0001) (**Figure 3B**). Importantly, we also observe projections of ectopic growth only in flies expressing the H2A-A127V with *E(z)* shRNAi (8% of the flies), which is significantly higher than flies expressing H2A wt with *E(z)* shRNAi (p-value=0.0192) or *E(z)* shRNAi alone (p-value=0.0045) with the latter groups showing minimal or no growth, respectively (**Figure 3C**). This suggests that H2A-A127V promotes tumor development in the *E(z)* knockdown flies, shifting the phenotype toward more pronounced growth defects, where only the H2A mutant resulted in ectopic leg formation in combination with *E(z)* knockdown, a phenotype that the H2A wt failed to promote.

### Expression of H2A-A127V mutant in flies reduces their life span and leads to defects in activity and sleep patterns

**Figure 4:**
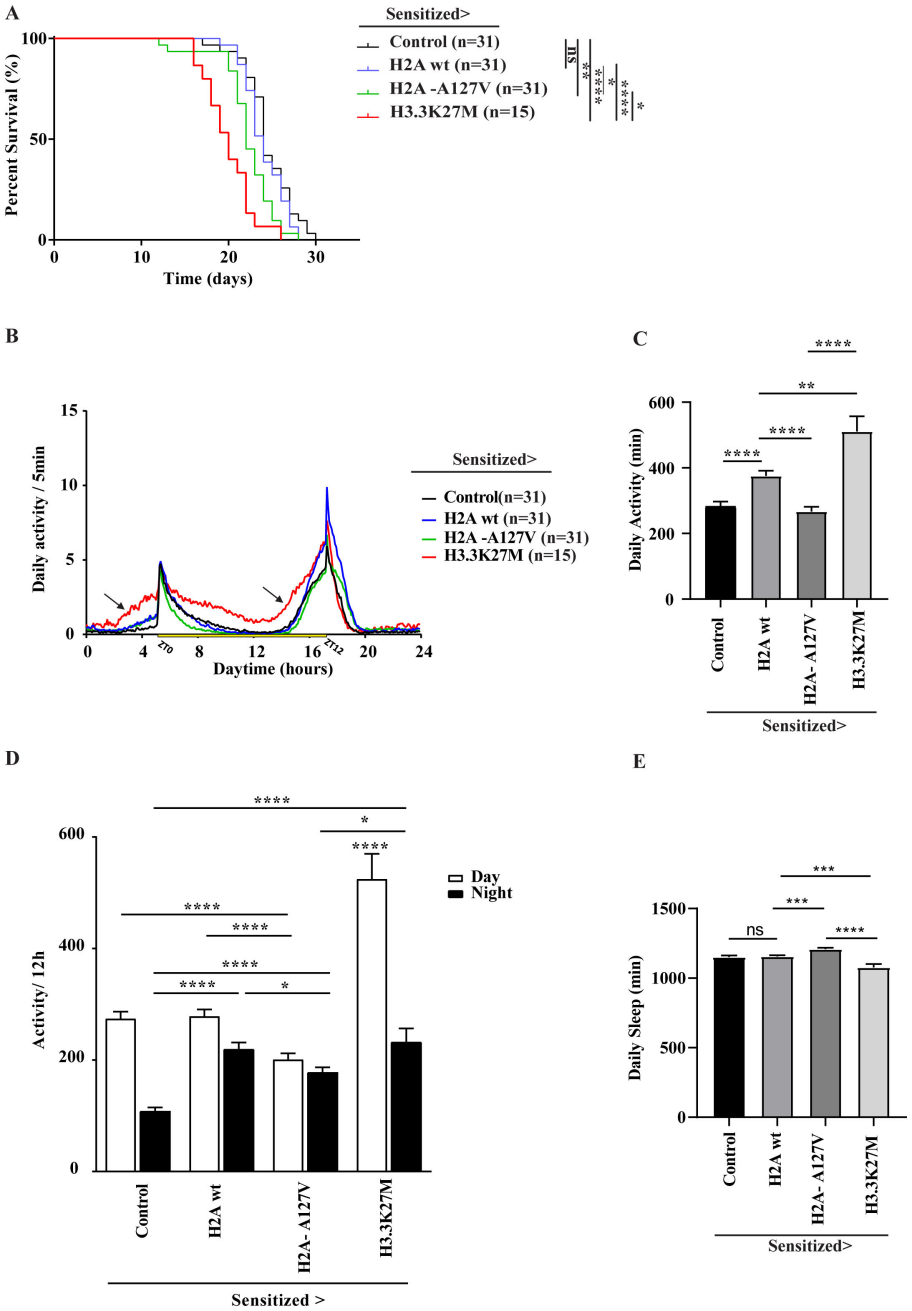
Expression of H2A-A127V sensitized non-tumorous flies results in reduced survival and behavioral disrupted phenotypes. **(A)** Kaplan-Meier curve showing the survival of male flies expressing H2A-A127V (n=31), H3.3K27M (n=15), H2A wt (n=31), and control (n=31) with sensitized *eyeless* driver. Monitoring of the flies was done with a *Drosophila* Activity Monitor with a detection window of 5 min over 30 days. Statistical significance was performed using the Mantel-Haenszel test to analyze the difference between survival curves (* p-value < 0.05, **p-value < 0.01, ****p-value < 0.0001, ns: p-value > 0.05). **(B)** The activity levels of individual male flies for each group were measured in 5 min bins and averaged to acquire a representative activity profile. Arrows highlight the anticipatory activity before the light (On-Off) transition states. ZT0 represents the morning peak and ZT 12 indicates the evening peak. **(C)** Graph illustrating the daily average activity of the male flies over 24 h intervals (Unpaired t-test; **p-value < 0.01, ****p-value < 0.0001). **(D)** Graph presenting the average locomotor activity over 12 h intervals (during the day: light is on, during the night: light is off) (Unpaired t-test; (Unpaired t-test; * p-value < 0.05, ****p-value < 0.0001). The asterisk over H3.3K27M during the day is in comparison to each of the other three groups. **(E)** Graph demonstrating the daily average sleep of the male flies over 24 h intervals (Unpaired t-test; ***p-value <0.001, ****p-value < 0.0001, ns: p-value > 0.05). The locomotor activity and sleep parameters of the flies were examined over 30 days. Error bars represent the standard error of the mean (SEM).

To further understand whether expression of H2A-A127V mutants in the sensitized eye model affects fly fitness and activity, we performed behavioral assays using the *Drosophila* activity monitor system (DAM). The importance of examining both lifespan and behavior in the context of histone mutations is highlighted in studies that have demonstrated how changes in histone proteins can disrupt cellular processes like DNA repair, gene expression regulation, and chromatin structure, all of which are linked to aging and fitness (45, 46). Additionally, in *Drosophila*, heterozygous mutations in the complex responsible for methylating H3K27, particularly in the PRC2 components E(z) and ESC, lead to a reduction in global H3K27me3 levels and an extension of male lifespan (47). The following flies were included in the assessment: control (*w^1118^* flies crossed to the sensitized background), H2A wt., H2A-A127V flies. We also used H3.3K27M mutation as control. H2A-A127V mutant flies displayed an overall decrease in the adult lifespan (median survival = 22) compared to the H2A wt (Median survival = 24, p-value=0.012) and the control (median survival = 24, p-value= 0.0014) (**Figure 4A**). H3.3K27M mutant flies displayed a more significant life span reduction (median survival = 20) compared to the H2A-A127V (median survival = 22, p-value= 0.0179) and control (median survival = 24, p-value <0.0001) (**Figure 4A**). On the other hand, the lifespan in the H2A wt flies (median survival = 24) was comparable to the control (median survival = 24, p-value=0.282) indicating no difference in the survival between the control groups. Our data indicates that the expression of the H2A-A127V mutation impaired the lifespan of flies albeit less than H3.3K27M mutation.

Disruptions in the circadian rhythm are implicated in tumorigenesis and cancer progression (48). Therefore, we investigated potential changes in fly behavior, focusing on activity and sleep patterns. Using the DAM system, the impact of H2A-A127V expression on fly behavior such as sleep, and activity was assessed over 30 days. H2A-A127V mutants and control flies demonstrated a bimodal activity pattern with two major activity bouts peaking at ZT0 (the morning peak) and ZT12 (the evening peak) (**Figure 4B**). H2A-A127V mutant flies exhibited lower activity after the ZT0 and ZT12, whereas H3.3K27M flies displayed an increased activity during the anticipation phase before ZT0 and ZT12 (**Figure 4B**). H2A-A127V mutant flies showed remarkably diminished daily locomotor activity (p-value < 0.0001; **Figure 4C**) which was more evident during the day (p-value < 0.0001) and night (p-value= 0.011) when conducted over 12 h compared to the H2A wt flies (**Figure 4D**). In contrast, the average daily activity was higher in H3.3K27M flies as compared to the H2A wt flies (**Figure 4C**; p-value= 0.0013) with the highest activity more eminent during the day (p-value < 0.0001; **Figure 4D**). H2A wt flies also showed an overall increase in the average daily locomotor activity compared to control (p-value < 0.0001; **Figure 4C**). This effect was most notable during the night (p-value < 0.0001; **Figure 4D**). These outcomes denote that H3.3K27M flies may exhibit higher activity during the light phase, whereas H2A-A127V flies display lower activity during the light and dark phases.

Alongside the activity profile, changes in sleep pattern were monitored and the average daily sleep was significantly higher in H2A-A127V mutants compared to H2A wt (**Figure 4E** p-value= 0.0001). Conversely, H3.3K27M flies reported a smaller amount of total sleep with respect to the H2A wt. (**Figure 4E**; p-value= 0.0006). The average daily sleep was similar in control and H2A wt flies (**Figure 4E**; p-value= 0.73). This implies that H2A-A127V flies may display longer sleep phases, whereas H3.3K27M flies exhibit shorter sleep phases. Although the overall daily activity of H2A wt flies was impaired, their sleep behavior remained unchanged, suggesting that the A127V variant not only enhances tumor growth but also affects overall fly fitness by worsening survival and resulting in flies exhibiting lower activities.

Together, these survival and activity assays provide additional evidence that A127V is a functionally relevant variant, with phenotypic effects that can be detected beyond tumor-related contexts.

## Discussion

Genetic alterations in histone H2A variants have been suggested as drivers for tumorigenesis, as well as potential biomarkers for early detection (49–51). In this work, we found that mutations in H2AC18 and H2AC19 variants are absent from public cohorts of healthy (gnomAD) and cancer (cBioPortal) samples due to the elimination of multi-mapped reads. We investigated the mutational profile of H2AC18/H2AC19 in AML and identified a previously unknown missense mutation, A127V. This H2A-A127V promotes survival and behavior changes in the *Drosophila* sensitized eye model and a major eye severity phenotype further pushing the tumor development in eyeful flies.

The filtration of multi-mapped reads can interfere with the mutational analysis of 958 genes that have repetitive or identical sequences (14). Given that approximately 3% of genes are oncogenic (584 tier 1 oncogenes/tumor suppressor genes recognized in the human genome of approximately 18,000 genes (COSMIC, Catalogue Of Somatic Mutations In Cancer)), novel mutations may be masked by multi-mapped filtering in approximately 30 oncogenes or tumor suppressor genes. Furthermore, the exclusion of these sequences can result in significant underestimation of expression in RNA-seq datasets, meaning that the important transcriptional changes in filtered oncogenes or tumor suppressor genes may be overlooked (14). Therefore, careful examination of duplicated sequences is an important step to uncover novel oncogenes and mutational events.

The complete sequence identity between the H2AC18 and H2AC19 genes prompted us to examine them in cancer samples. We identified the H2AC18/19 A127V variant in AML and other equivalent variants in cancer, with both a remarkable enrichment in hematological malignancies and somatic occurrence. Critically, the combined evidence of our *in vivo* functional analysis, the high frequency of this variant in specific cancer subtypes, and the somatic occurrence in cancer samples strongly supports a role for this variant in cancer development or progression. We also identified the H2AC18/19 A127V variant in a subset of the healthy population, especially within the European ancestry, and this raises the possibility that the H2A-A127 variants may increase risk for cancer development in these individuals. In support of this, many studies have detected strong associations between genetic polymorphisms and disease occurrence (52).

Our study provides evidence that the H2A-A127V mutation enhances tumor progression and modifies phenotypic outcomes in both tumor and sensitized non-tumor models of *Drosophila*. By employing the eyeful model, which serves as a robust system to study tumorigenesis, we demonstrated that the H2A-A127V mutation significantly aggravates tumor development, as indicated by an increase in severe eye fold phenotypes and metastatic incidence. Furthermore, the use of a sensitized non-tumor model highlighted the mutation’s subtle but measurable impact in transforming a pre-tumor background into a more aggressive state, particularly when combined with key oncogenic and epigenetic regulators. The eyeful model revealed a clear tumor-promoting role for H2A-A127V, with a significant increase in both major eye fold severity and metastatic spread. This underscores the potential of the A127V mutation to exacerbate tumor phenotypes in a genetically primed environment. Notably, the metastases observed at distinct body locations, including the genital region, highlights that H2A-A127V may influence cellular pathways associated with tumor invasion and metastasis, making it a candidate for further investigation in cancer models. Both *lola* and *psq*, which are mutated in the eyeful model, encode chromatin-associated transcriptional regulators. Lola is a BTB-zinc finger transcription factor and Psq is a pipsqueak/BTB domain factor that modulate gene expression through chromatin compaction and Polycomb-related mechanisms (53). As a core histone, H2A variant and mutant forms are known to alter nucleosome stability and chromatin accessibility at target loci (3). We therefore speculate that H2A-A127V, by altering nucleosome composition at regulatory regions, may sensitize chromatin to the transcriptional dysregulation driven by *lola* and *psq* overexpression, thereby enhancing their tumorigenic output. Though the precise molecular mechanism connecting them remains to be fully understood.

A notable observation in our Drosophila functional studies is that wild-type H2A overexpression produces a modest enhancement of the eyeful baseline phenotype, which may appear to limit the specificity of H2A-A127V effects. However, several lines of evidence support the mutation-specific nature of the A127V variant. First, H2A-WT overexpression in the sensitized but non-tumorous model produced no detectable eye phenotype, demonstrating that histone overexpression alone is insufficient to drive tumorigenic effects and that a pre-established epigenetically dysregulated background is required. Second, H2A-A127V consistently and significantly exacerbates both fold severity and metastasis rates beyond H2A-WT in the eyeful model, indicating that the differential effect is attributable to the mutation rather than to non-specific histone dosage. This is consistent with our previous findings showing that H3.3-WT overexpression produces no phenotypes in either sensitized or non-sensitized contexts, whereas H3.3 K27M and K36M oncohistone mutants generate distinct tumorigenic outcomes (17). Finally, results from an independent transgenic line confirmed that the enhanced phenotype is specifically associated with the A127V substitution and is not a consequence of transgene background or histone dosage effects (**Supplementary Figure 4**).

Several studies have shown that alanine substitutions could alter protein structure and chemical properties (54, 55). The H2AC18/19-A127V mutation is located at a flexible C-terminal tail unique to H2A histones (13, 56, 57). Unlike the unstructured N-terminal tail common to all core histone subunits, the C-terminal amino acids of H2A variants display a high degree of heterogeneity (57), and have been linked to the regulation of chromatin dynamics, remodeling, and histone H1 binding (56). In the sensitized model, the limited baseline phenotypes observed with the H2A-A127V mutation alone suggest that its oncogenic potential requires a cooperating genetic context. This was evident when combining the mutation with knockdowns of *ADD1* or *Jarid2*, where a rescuing effect was observed. The significant reduction in eye folds with *ADD1* or *Jarid2* knockdown with wild-type H2A was further amplified by the A127V mutation. This supports a potential interaction between H2A modifications and heterochromatin organization mediated by ADD1. As Jarid2 functions to recruit PRC2 via mono-ubiquitylated H2A, the enhanced reduction suggests that A127V might impact PRC2 recruitment, thereby altering the repressive chromatin landscape. These two observations point to the possibility of the H2A-A127V mutant and the H2A wild-type allow restoration of repressive chromatin lost by *ADD1* and *Jarid2* knockdowns. On the other hand, a synergistic effect was shown when combining the mutation with knockdown of *lozenge*. The eye phenotype observed when combining lozenge knockdown with H2A-A127V is modest but statistically significant only when compared to the RNAi control, while the difference relative to H2A-WT is there but minimal. This pattern suggests that both wild-type H2A and the A127V variant share the capacity to modulate the transcriptional program disrupted by lozenge loss, and that the A127V substitution confers only a marginal additional effect in this context Lozenge is reported to activate genes that promote apoptosis during eye development (58). This supports a potential interaction between H2A modifications and transcription regulation mediated by Lozenge, where potentially wild-type human histone H2A and the A127V mutant further repress the genes no longer activated by Lozenge to enhance the eye fold phenotype. These observations strongly suggest that H2A-A127V interact with chromatin remodeling defects. The stronger effect of the A127V mutant suggests it is better at restoring repression in these three backgrounds.

Deregulation of genes encoding the polycomb repressive complexes has been documented in several hematological malignancies where they exhibit tumor-suppressive or oncogenic functions (59). PRC1 and PRC2 complexes display catalytic activities specific to the H2A lysine 119 ubiquitination (H2AK119ub1) and H3 lysine 27 trimethylation (H3K27me3) respectively (60). H2A-A127V lies in the proximity of the H2AK119 mono-ubiquitination site, raising the possibility that the H2A-A127V mutation may disrupt the K119 ubiquitination and, in turn, the expression of downstream genes. This effect could occur through several plausible routes. A single substitution can alter chromatin biology by changing local hydrophobicity or side-chain packing, thereby affecting histone-histone contacts within the nucleosome, histone-DNA affinity, or interactions with chaperones and chromatin remodeling factors. In addition, the substitution may perturb nearby post-translational modification sites or influence the recruitment of chromatin regulators. Alternatively, it could alter variant deposition dynamics, leading to mislocalization of the mutated histone and reduced accessibility to the PRC1 complex, ultimately affecting H2AK119 mono-ubiquitination. The interaction of H2A-A127V with epigenetic regulators, particularly components of the PRC1 and PRC2 complexes, provided further insights into its role in chromatin dynamics. Despite differences in DNA methylation, Drosophila retains highly conserved Polycomb group proteins and H3K27me3-mediated regulation, allowing it to serve as an informative *in vivo* model to study the functional impact of human histone variants on chromatin and gene regulation. While no significant differences were observed between wild-type H2A and A127V in combination with *Kdm2, E(z)*or *Su(z)12* knockdowns, the combination still attenuated eye fold phenotypes compared to individual knockdowns of these genes. This indicates that expression of H2A may subtly modulate chromatin states in these contexts. As the A127V is a conservative mutation but slightly bulkier [CH(CH3)2 instead of CH3], which could explain why we don’t observe a difference in the eye phenotype between H2A-A127V mutant and the H2A wild-type in the case of *Kdm2*, *Su(z)12*, or *E(z)* shRNAi knockdowns. As a possible future direction, it would be worth assessing whether a bulkier side group or a polar or charged side group would have a stronger effect, resulting in a difference from the H2A wild-type.

Knocking down the PRC2 component *E(z)* in the H2A-A127V mutant/sensitized model resulted in inducing overgrowth and ectopic leg formation although eye fold severity is comparable in both H2A wild type and H2A-A127V. This suggests that the H2A-A127V mutation may interact with PRC2-mediated pathways. These findings align with previously established roles of H3K27me3 and PRC2 deregulation in cancer, where Ezh2 has been shown to play a critical role in leukemia progression and activity (59, 61, 62). The depletion of *E(z)* in H2A-A127Vs likely diminished H3K27 trimethylation, weakening the repressive chromatin state. This disruption could impair tumor suppressor pathways and contribute to the ectopic growths observed in the H2A-A127V flies. Other more sensitive methods such as mass spectrometry or ChIP-seq will be required to rigorously assess the impact of H2A-A127V on H2AK119 ubiquitination and H3K27 trimethylation levels. ChIP-seq in particular would allow us to determine whether the changes in these marks are global or locus-specific should be employed to assess ubiquitination of K119 (**Supplementary Figures 5, 6**).

Finally, the expression of the H2A-A127V mutation exerted detrimental impacts on the fly activity and sleep shaping the circadian behavior. Circadian rhythms, driven by endogenous molecular clocks allow flies to anticipate environmental changes and contribute to courtship and eclosion during development (63). These molecular rhythms however are highly dependent on environmental parameters including the light (64). Therefore, it is predicted that when the light perception organ in the *Drosophila* model is disrupted there will be profound changes in the circadian rhythms. These changes are observed in our study, where locomotion activity, as well as survival, is attenuated in sensitized flies expressing the histone H2A mutation. In addition, the deregulation in the circadian behavior increases with promoted disruption in the *Drosophila* eyes, as reported in the comparison between histone H2A and H3 mutants, where H3.3K27M is more penetrant. Consequently, H3.3K27M flies exhibit hyperactivity, whereas H2A-A127V flies demonstrate lethargic behavior, potentially reflecting distinct underlying molecular perturbations that nevertheless disrupt circadian homeostasis to varying degrees. Both mutants ultimately converge on an oncogenic phenotype marked by reduced lifespan, though with different magnitudes. The heightened activity observed in H3.3K27M mutants may contribute to increased physiological stress and corresponds with the most pronounced adult lethality, while H2A-A127V mutants show a milder yet still significant reduction in lifespan. In support of our observations in flies, a recent study identified an association between cancer and disrupted activity and sleep behaviors, where the deregulation of the circadian rhythm potentially contributes to tumorigenesis (65). While the underlying mechanism by which the H2A-A127V variant drive cellular growth is conserved, the physiological outcome of this homeostatic disruption can not be generalized to other tissues as we have tested adult eye model and fly survival in this current study. Therefore, the functional consequences of the variant observed in the eye tissue model may not reflect potential outcomes when expressed in the hematopoietic system of higher organisms. Nevertheless, these findings provide evidence that this substitution has the capacity to deregulate homeostatic functions in the tissues in which it is expressed.

In summary, we identified a novel A127V mutation in H2AC18 and H2AC19 genes as an overlooked SNP that is linked to hematopoietic malignancies. Our functional studies identify H2A-A127V as a key modifier of tumor development and progression in both sensitized and tumorous Drosophila models. Its interaction with AML-related genes and epigenetic regulators within the PRC1 and PRC2 complexes highlights its potential involvement in chromatin-mediated mechanisms driving tumorigenesis. These results highlight the need to explore genomic data to identify overlooked oncogenic mutations that may exist across cancer types.

## Data Availability

The original contributions presented in the study are included in the article/**Supplementary Material**. Further inquiries can be directed to the corresponding authors.

## Supplementary material

Supplementary Table 1 Table summarizing the p-values of all comparisons from the eyeful, AML and PRC1/2 screens.

Supplementary Table 2 Cytogenetic and mutational characteristics of H2A-A127V and H2A-wt AML samples in the Leucegene dataset.

Supplementary Table 3 Table illustrating the enrichment of H2AC18/19-A127V mutation in several populations using the 1000 Genomes Project.

Supplementary Table 4 Table summarizing highly conserved human genes that are frequently mutated in AML in *Drosophila melanogaster*.

Supplementary Table 5 Table summarizing highly conserved human genes of PRC1 and PRC2 components in *Drosophila melanogaster*.

Supplementary Figure 1 Coverage tracks for the 5 AML samples carrying A127V in the validation cohort.

Supplementary Figure 2 Multi-sequence alignment of the c-terminal domain of histone H2A variants.

Supplementary Figure 3 Alignment of human H2AC19 with orthologous H2A proteins from chimpanzee (PANTR; H2AC19), mouse (Hist2h2aa1), *Saccharomyces cerevisiae* (yeast; HTA1 and HTA2), and *Drosophila melanogaster* (DROME; His2A).

Supplementary Figure 4 Scoring the severity of eye phenotype (%) for different H2A wt lines under the control of eyeful driver, at 25 °C (***, p-value <0.001 and ****, p-value <0.0001; chi-square test). 5-M2-H2A A4A wt and 5-M1-H2A A4A wt (the H2A wt line used in this study) are different batches of transgenic lines sent by the company, having the H2A inserted in the same locus. 1-M1-H2AD wt and 3-M3-H2A BG wt are transgenic flies of other H2A variants. The same scoring system as **Figure 1** was used: minor (enlarged eyes with no folds), moderate (1–4 folds), and major (>4 folds) severity. Flies were collected from more than 3 biological replicates.

Supplementary Figure 5 The levels of H2A Lys119 Ubiquitin and H3K27 trimethyl are decreased in both the wt and mutant form of H2A. **(A)** Representative Western blot profile on protein extracts from adult heads of flies expressing histone H2A-A127V mutant, H2A wt or control driven by sensitized *eyeless* driver. The blots were probed with anti-β actin, anti-Ub-H2A and anti-H3K27me3. The first lane represents the control, the second lane represents the H2A wt and the third lane represents the H2A-A127V. The cropped blots are from the same membrane. **(B)** Quantification of H2A Lys119 ubiquitination and H3K27 levels in adult fly heads expressing H2A-A127V, H2A wild-type (wt), or control. Band intensities were normalized to loading controls and averaged across three independent Western blot replicates (Unpaired t-test; * p-value < 0.05, **p-value < 0.01). Error bars represent the standard deviation (SD).

Supplementary Figure 6 Replicate experiments corresponding to the Western blot shown in **Supplementary Figure 5**. **(A)** colorimetric and **(B)** chemiluminescence images of full blots from three independent replicates, shown at multiple exposure times (10 sec, 20 sec, 30 sec, 40 sec, and 50 sec). The cropped blot in **Supplementary Figure 5** is from replicate 3.

Supplementary Figure 7 RNAi knockdown validation. **(A)** *Jarid 2* RNAi knockdown samples exhibited a non-significant decrease in *Jarid 2* expression compared with *gfp* RNAi controls **(B)**. *p53* RNAi samples exhibited a non- significant decrease in *p53* expression compared with *gfp* RNAi controls **(C)**. *Kdm2* RNAi samples exhibited a non-significant decrease in *Kdm2* expression compared with *gfp* RNAi controls **(D)**. *E(z)* RNAi samples exhibited a non-significant decrease in *E(z)* expression compared with *gfp* RNAi controls. **(E)** *Nlp* RNAi samples exhibited a non-significant decrease in *Nlp* expression compared with *gfp* RNAi controls **(F)** *Pc* RNAi samples exhibited a non-significant decrease in *Pc* expression compared with *gfp* RNAi controls **(G)** *ADD1* RNAi (1) samples exhibited a non-significant decrease in *ADD1* expression compared with *gfp* RNAi controls in the first RNAi fly line. **(H)** *ADD1* RNAi (2) samples exhibited a non-significant decrease in *ADD1* expression compared with *gfp* RNAi controls in the second RNAi fly line. (I) *dTet* RNAi samples exhibited a non-significant decrease in *dTet* expression compared with *gfp* RNAi controls. Statistical comparisons were performed using an unpaired two-tailed Student’s t-test (ns; p > 0.05). Data points represent individual biological replicates, bars indicate group means, and error bars represent the standard deviation (SEM). GFP, green fluorescent protein; RNAi, RNA interference; P53, tumor protein p53; Kdm2-23, lysine demethylase 2-23; EZ, enhancer of zeste gene; NLP, neuropeptide-like protein; PC, pyruvate carboxylase; Jarid, Jumanji AT-rich interactive domain-containing protein; Add, adducin; ns, non-significant.

Supplementary Data Sheet 1 Leucegene coverage tracks showing the last 147 nucleotides of H2AC19 gene corresponding to the last 48 C-terminal amino acids. Tracks were generated and visualized using IGV 2.11.2.

Supplementary Data Sheet 2 1000-genomes coverage tracks showing the last 147 nucleotides of H2AC19 gene corresponding to the last 48 C-terminal amino acids. Tracks were generated and visualized using IGV 2.11.2.
