# Supplementary material for: A novel H2A-A127 variant is associated with human cancer and enhances tumor-related phenotypes in Drosophila melanogaster models
Source: Front Oncol. 2026 Jul 13;16:1814908. doi: 10.3389/fonc.2026.1814908 (PMC13402181; doi:10.3389/fonc.2026.1814908)

GSE49642
(43 samples)

1.GSM1203305-GSM1203324

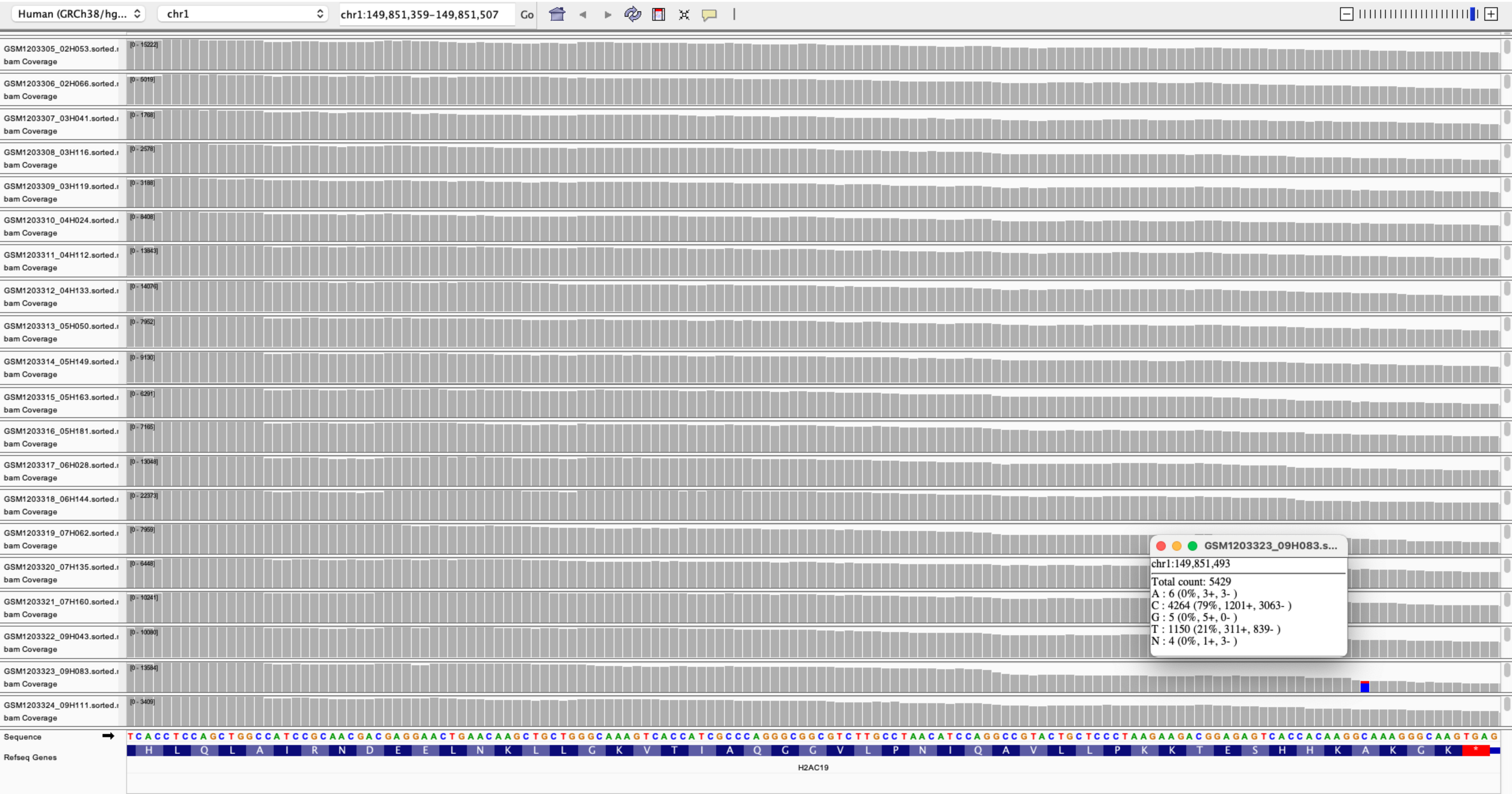

2.GSM1203325-GSM1203344

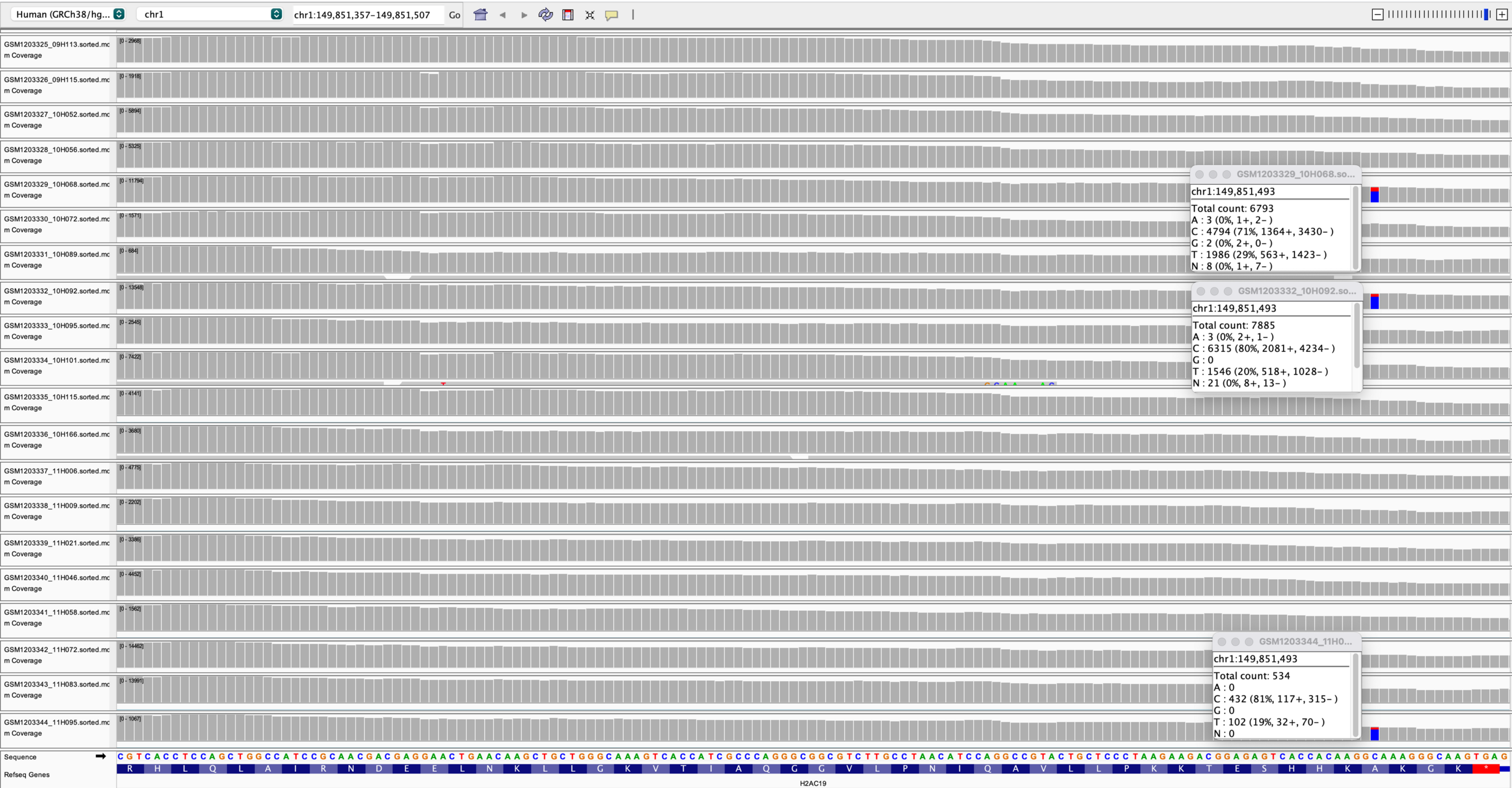

3.GSM1203345-GSM1203347

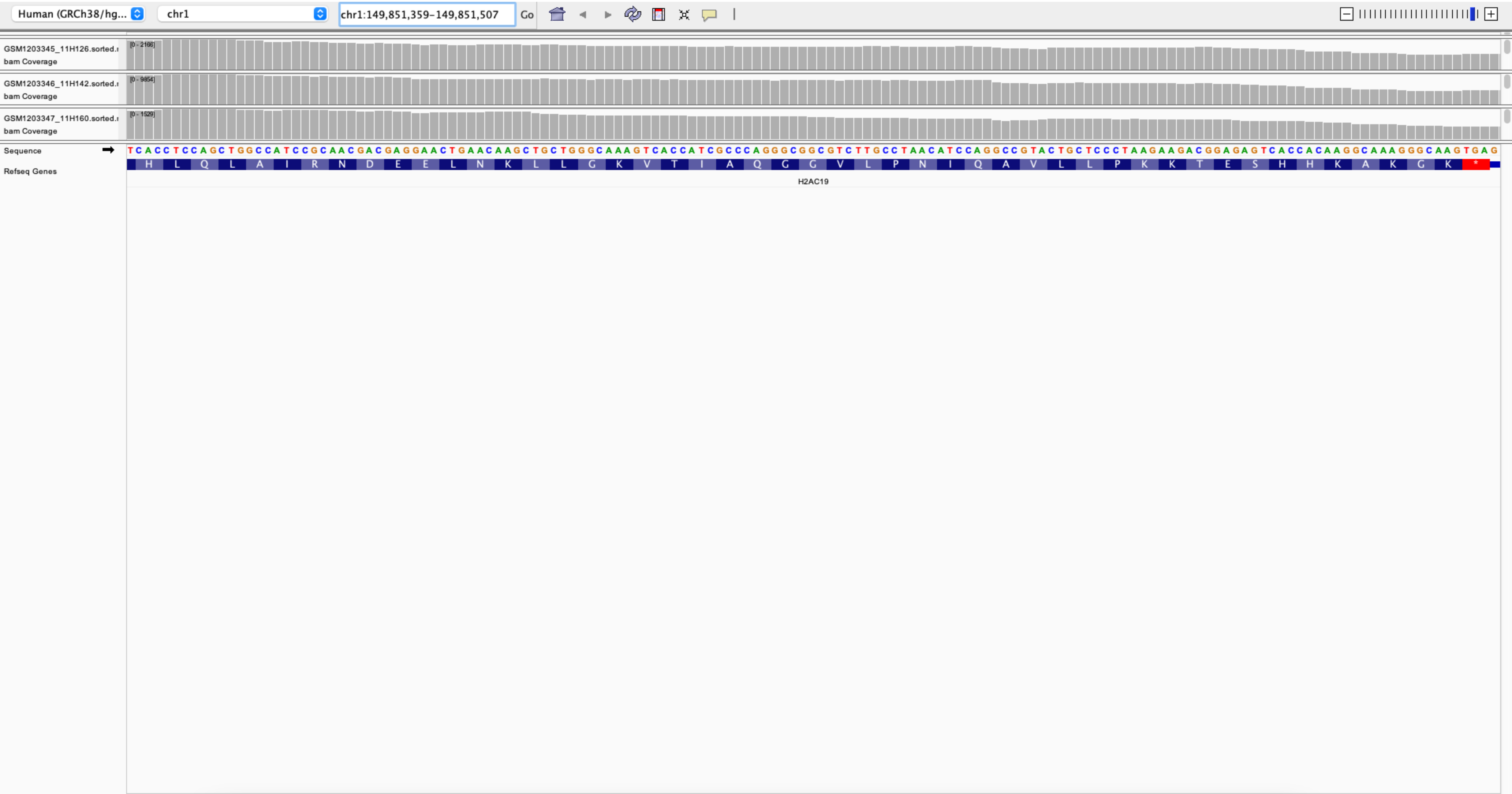

GSE52656
(27 samples)

4.GSM1273615-GSM1273634

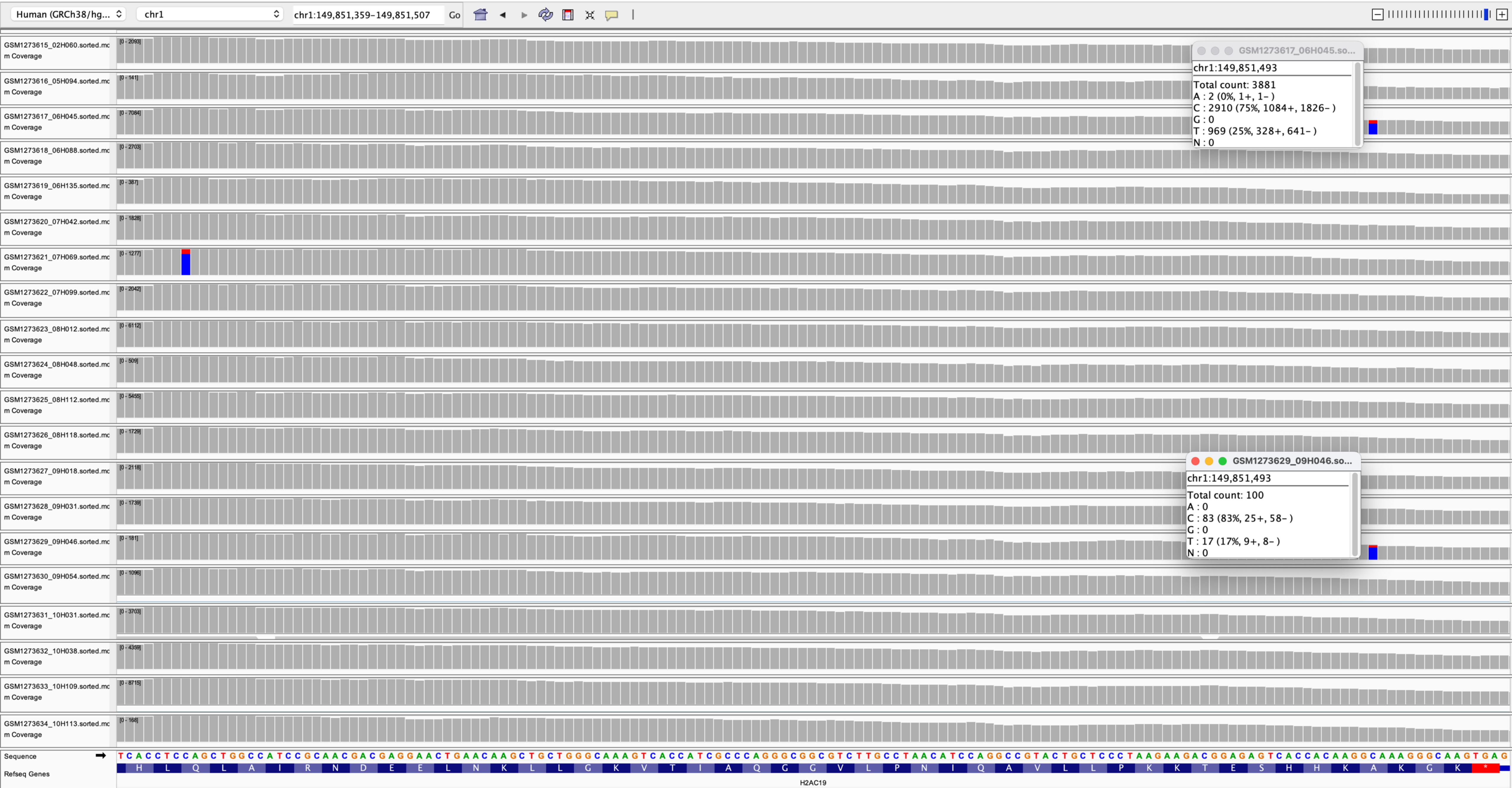

5.GSM1273635-GSM1273641

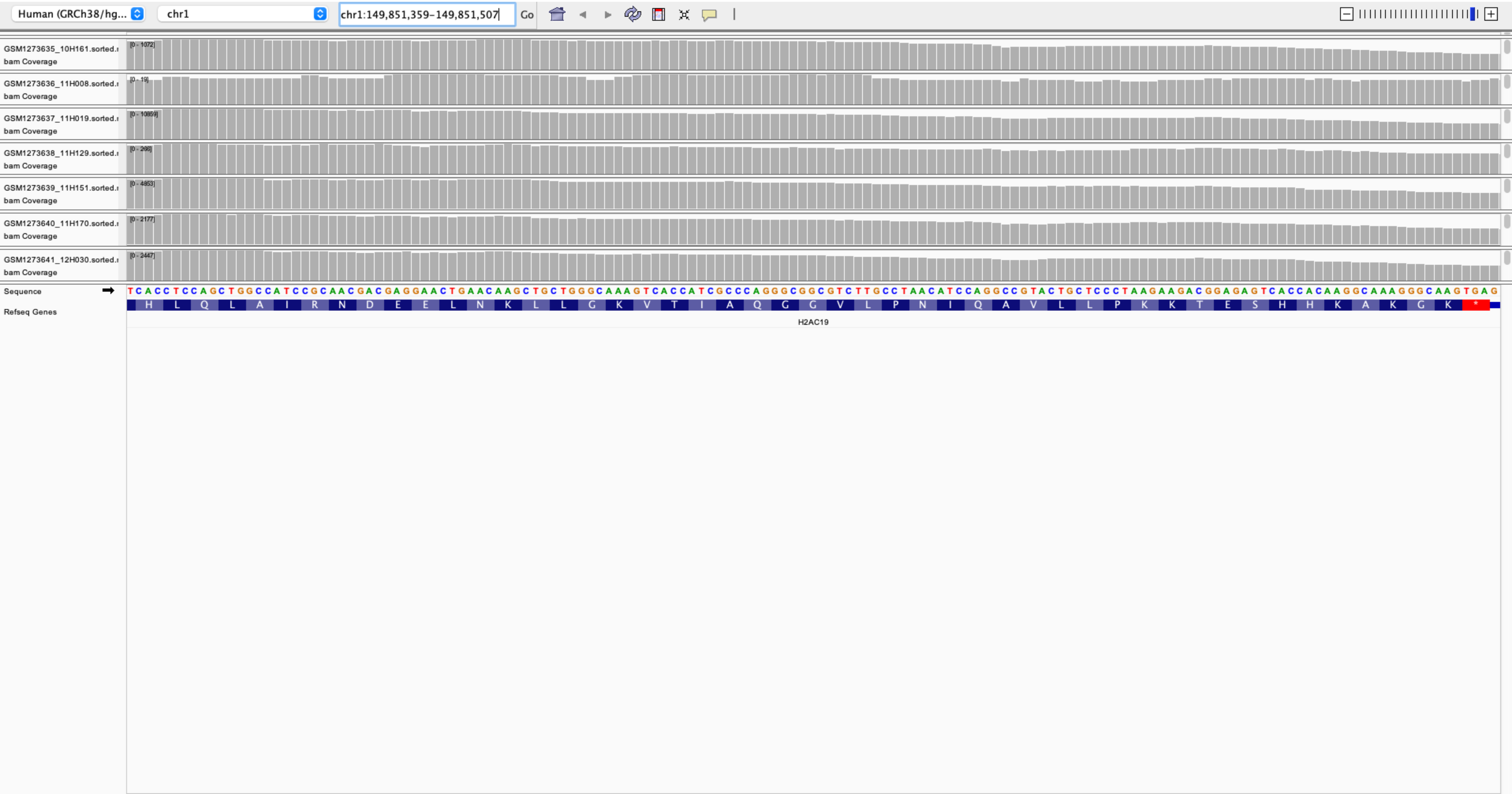

GSE62190
(82 samples)

6.GSM1521543-GSM1521562

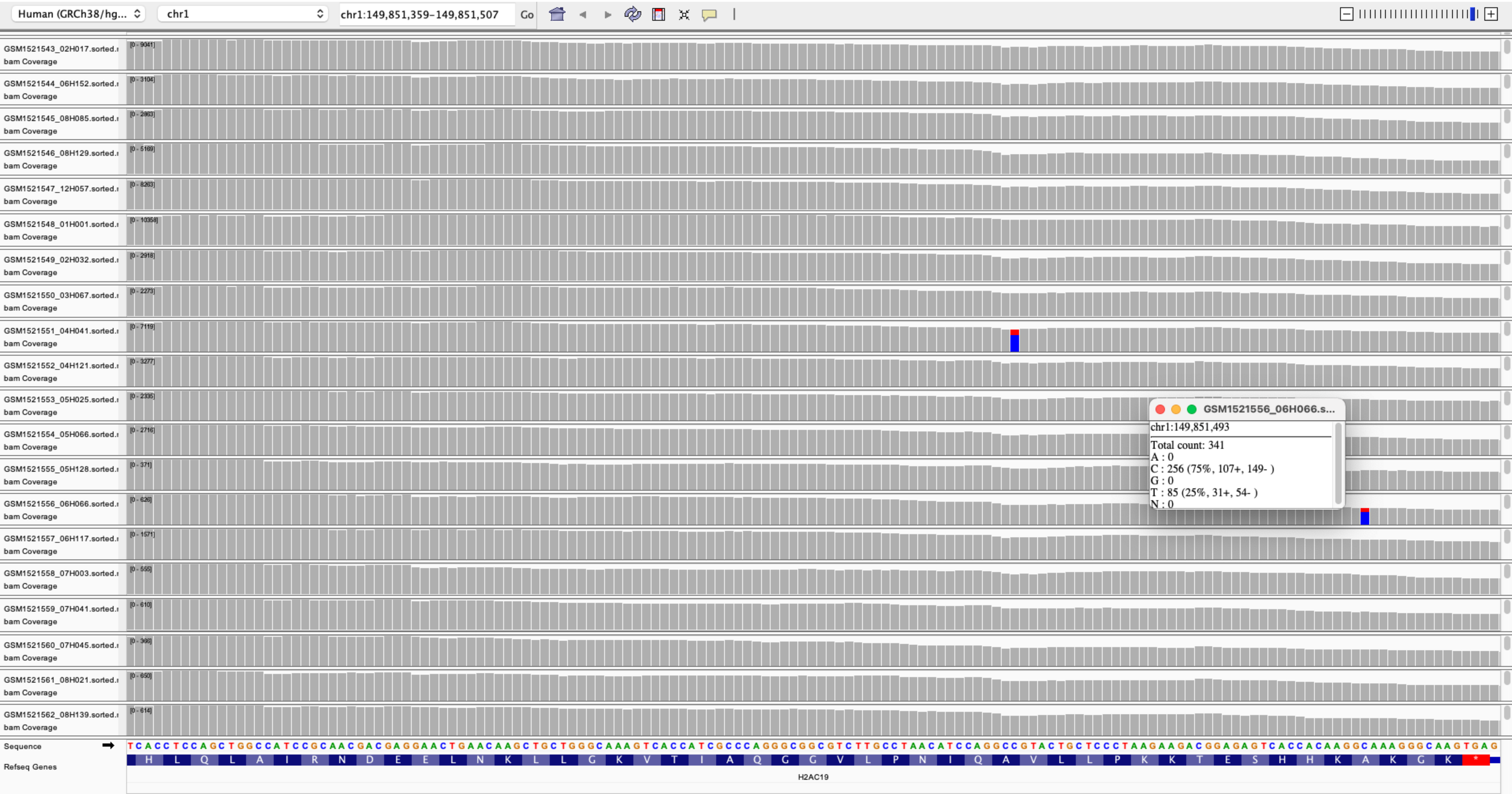

7.GSM1521563-GSM1521582

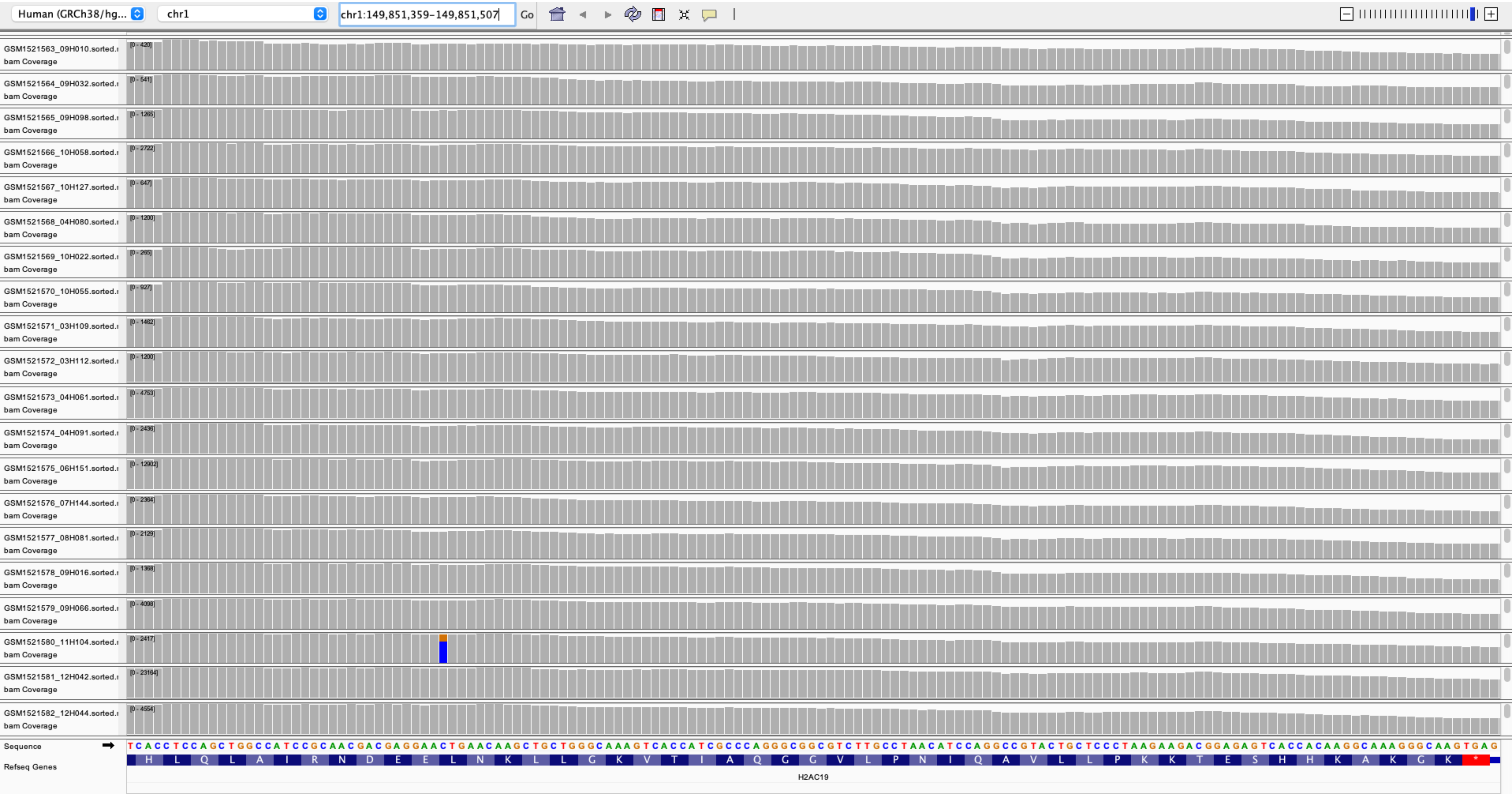

8.GSM1521583-GSM1521602

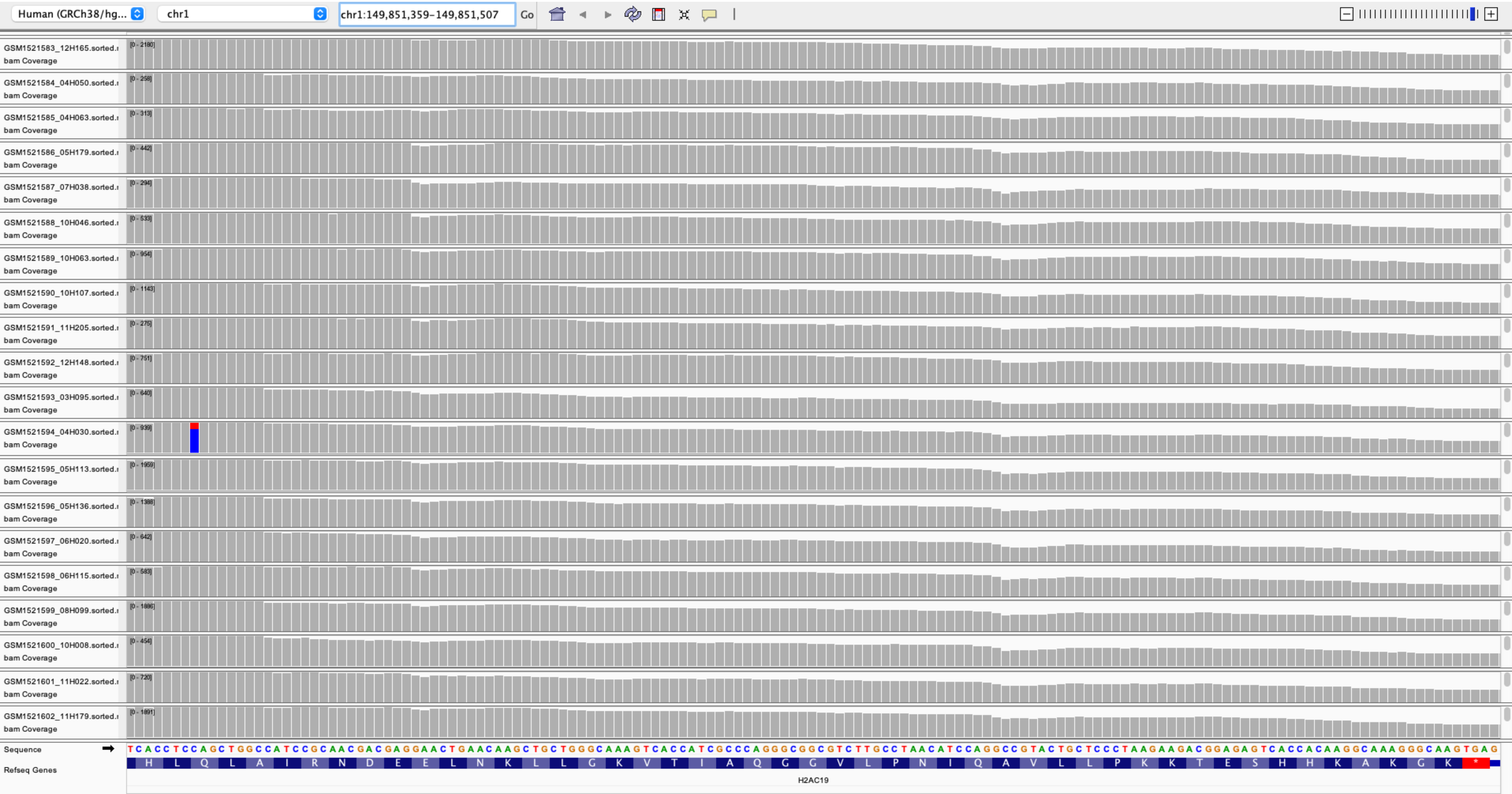

9.GSM1521603-GSM1521622

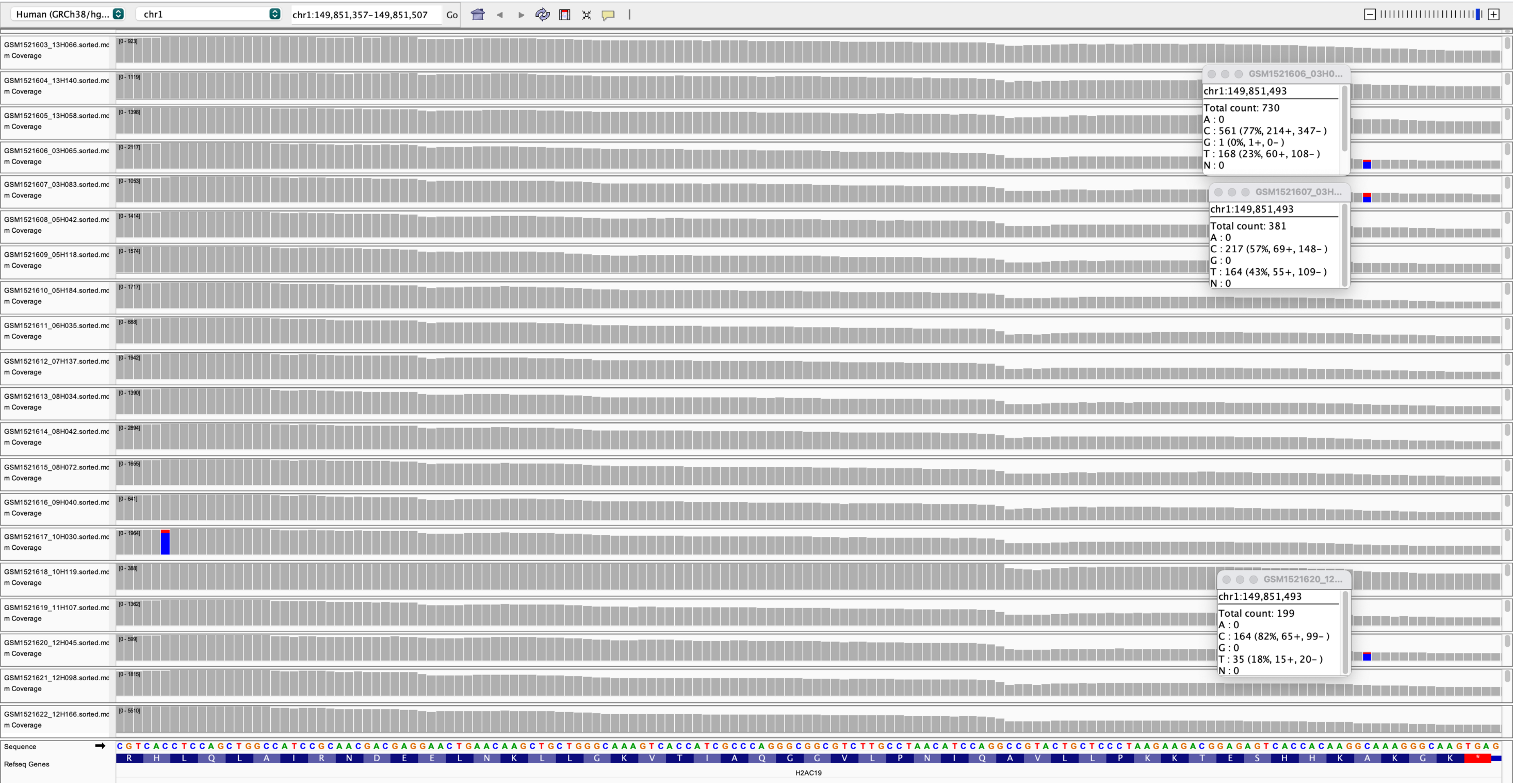

10.GSM1521623-GSM1521624

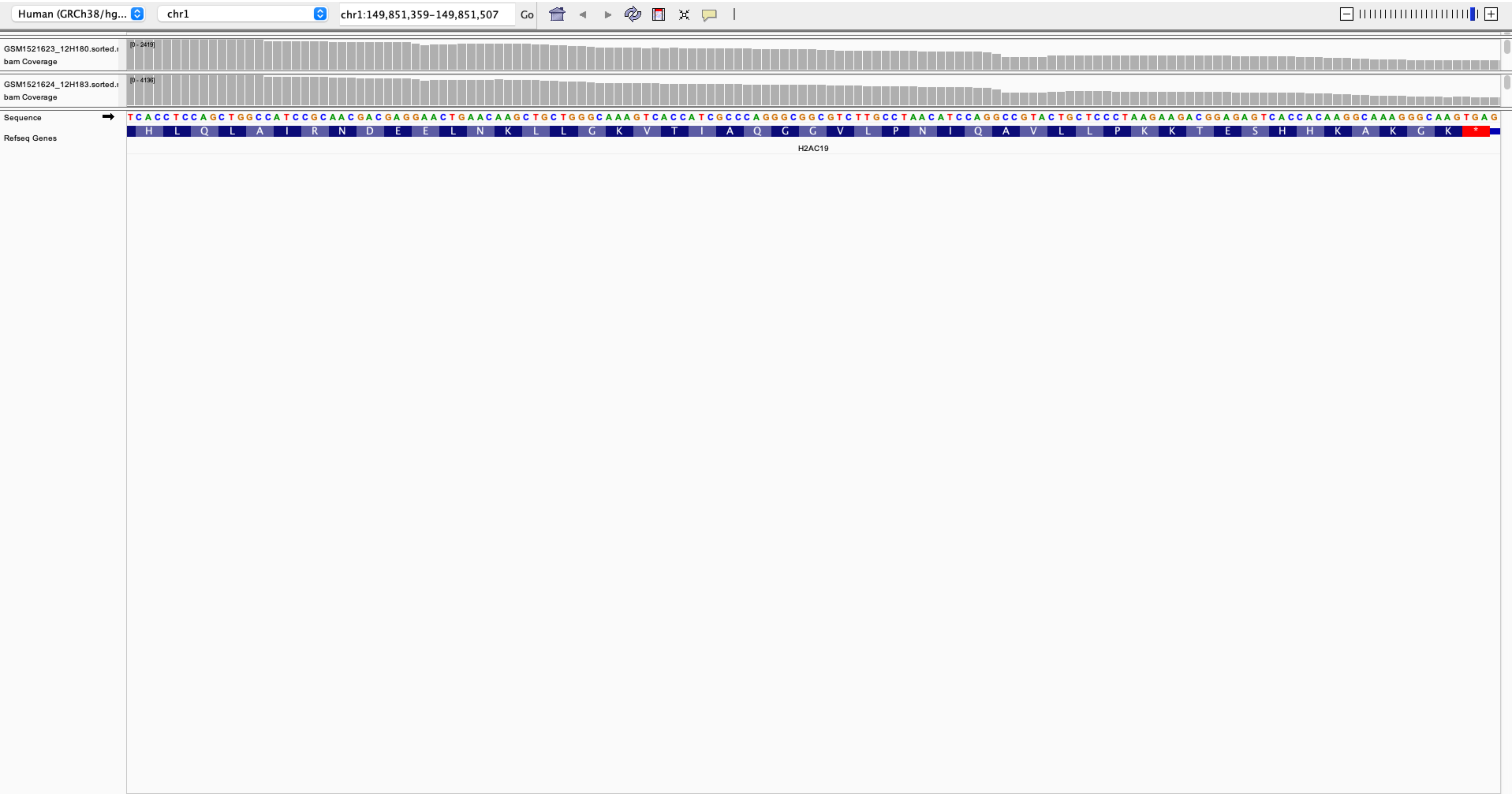

GSE66917
(22 samples)

11.GSM1634279-GSM1634298

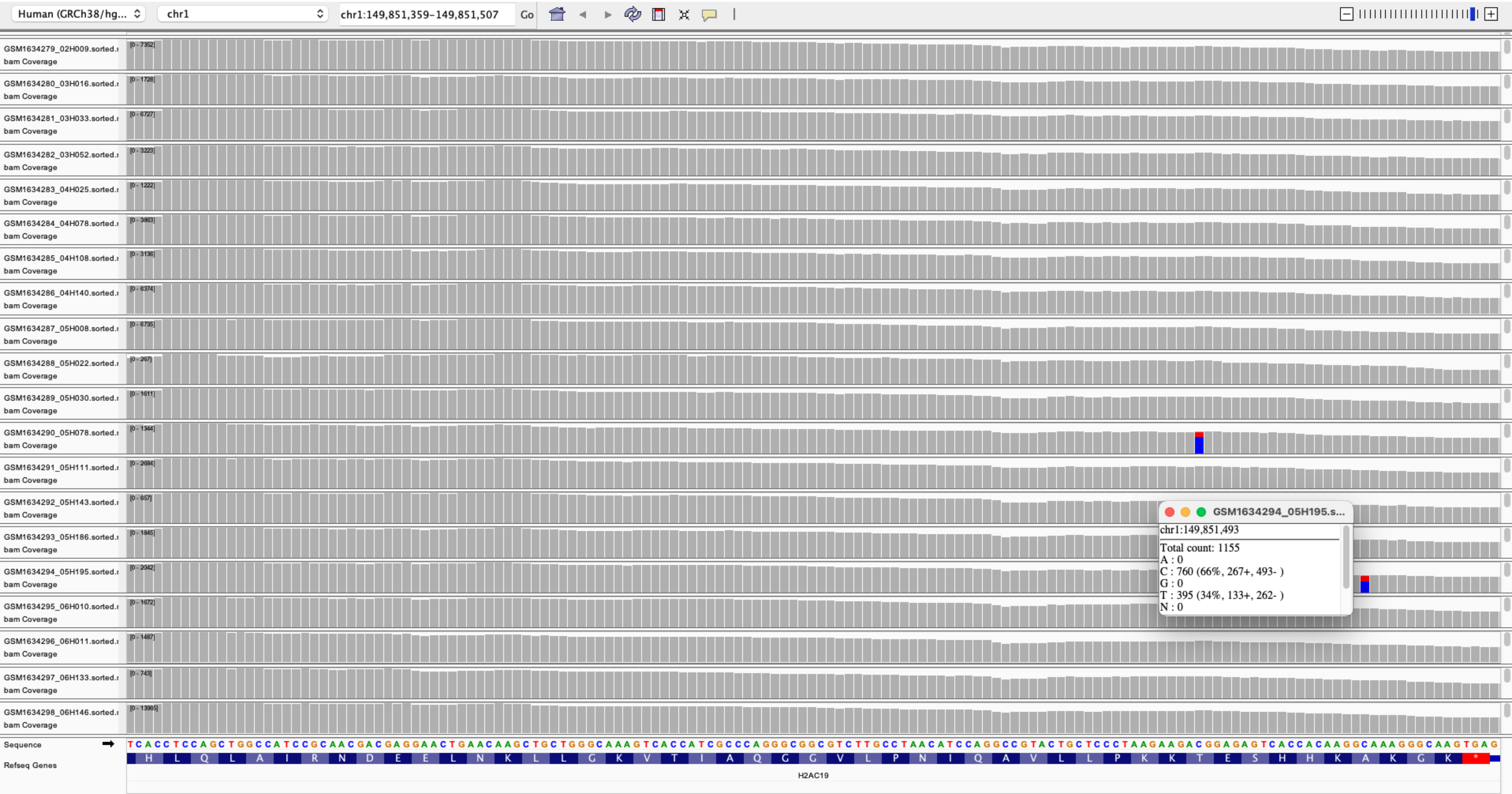

12.GSM1634299-GSM1634300

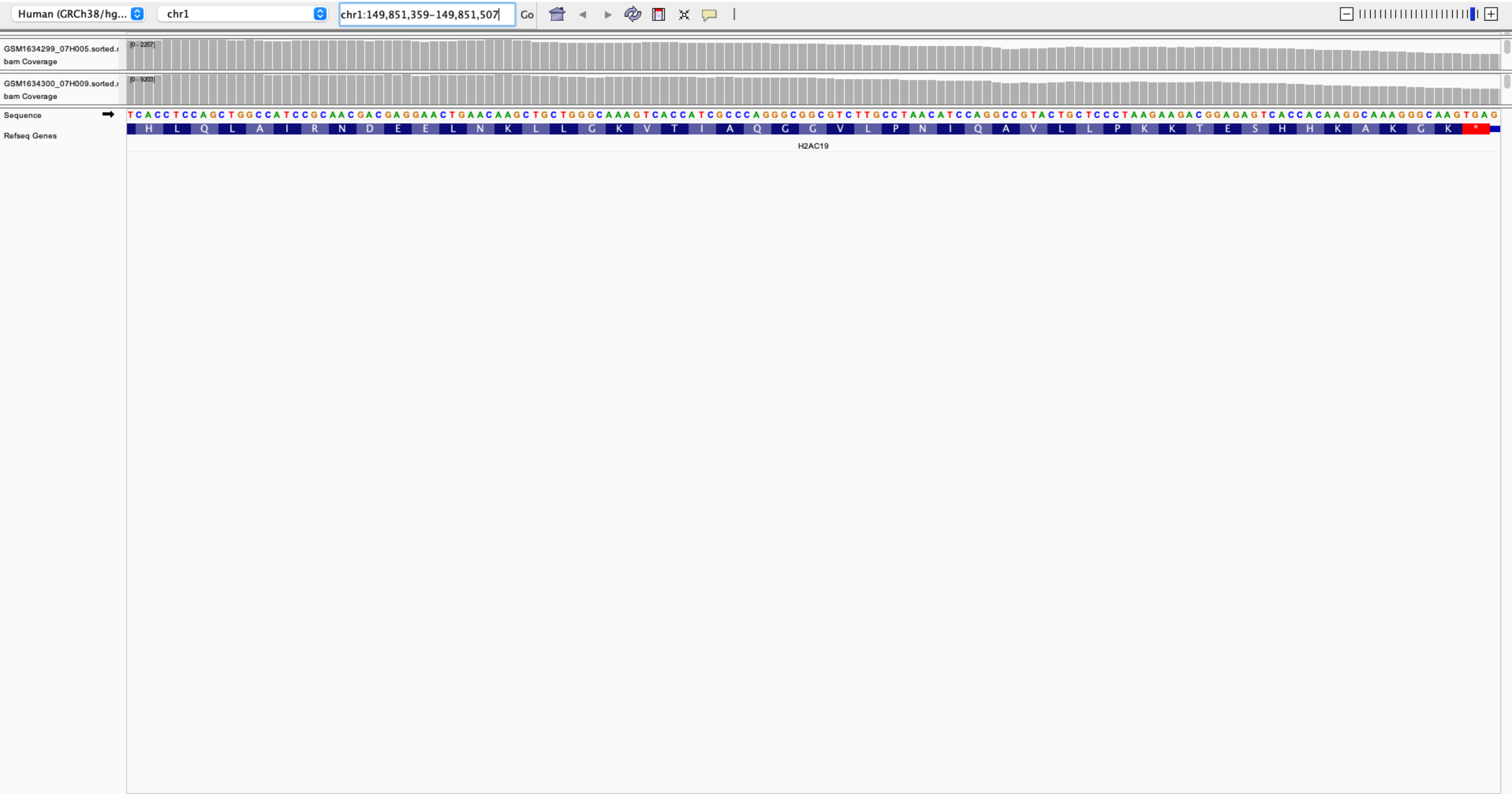

GSE67039
(263 samples)

13.GSM1636667-GSM1636686

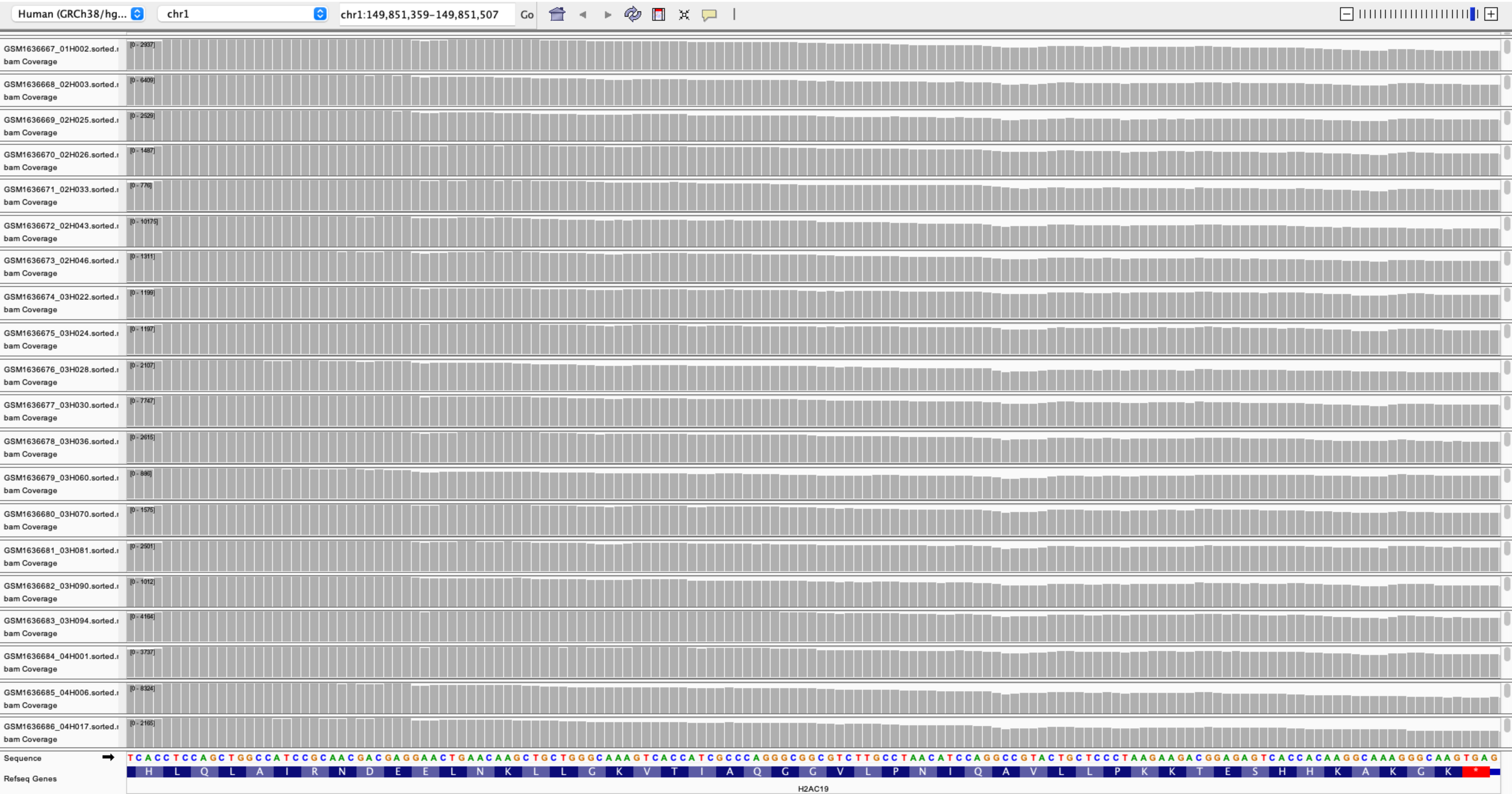

14.GSM1636687-GSM1636706

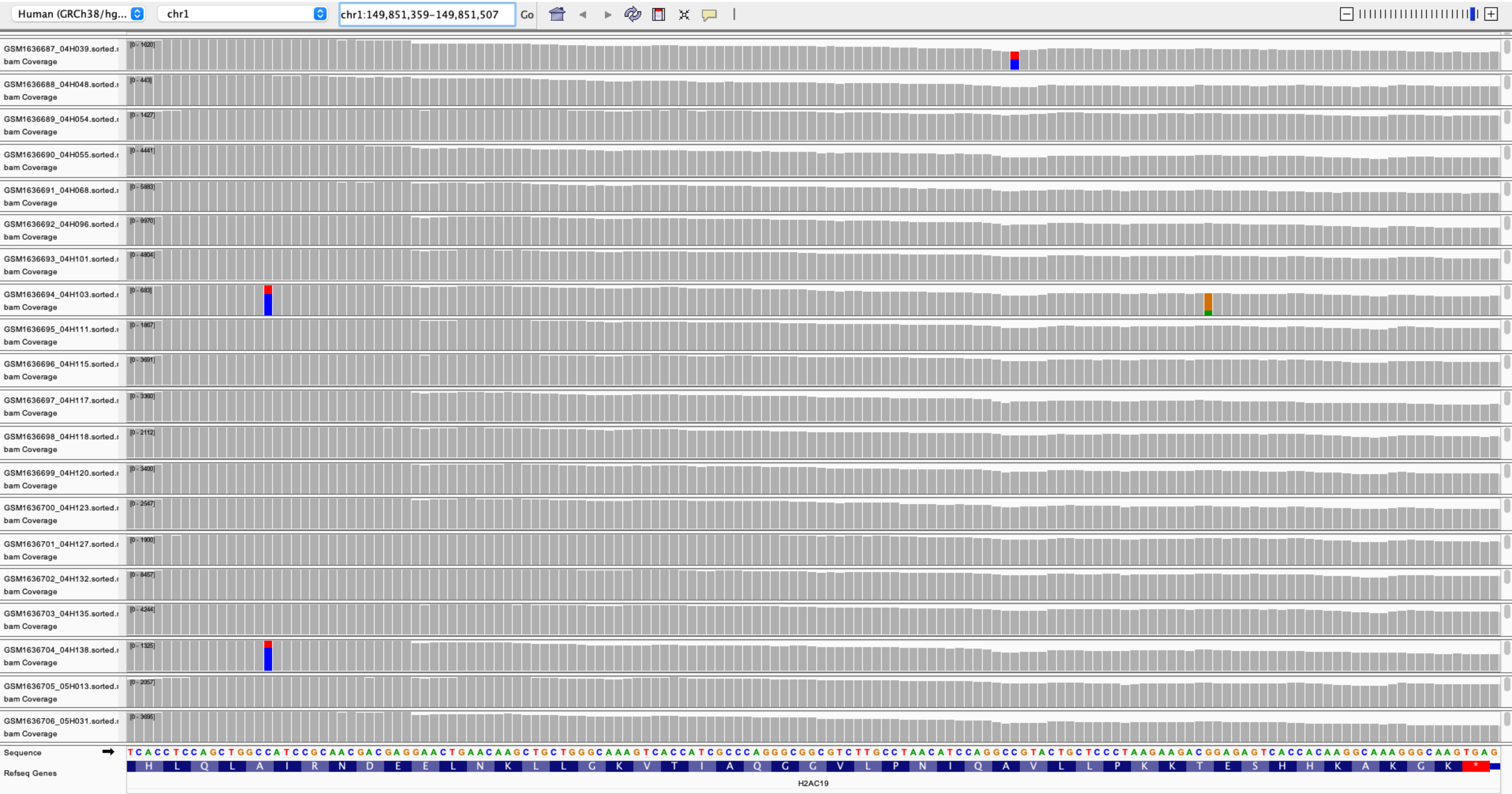

15.GSM1636707-GSM1636726

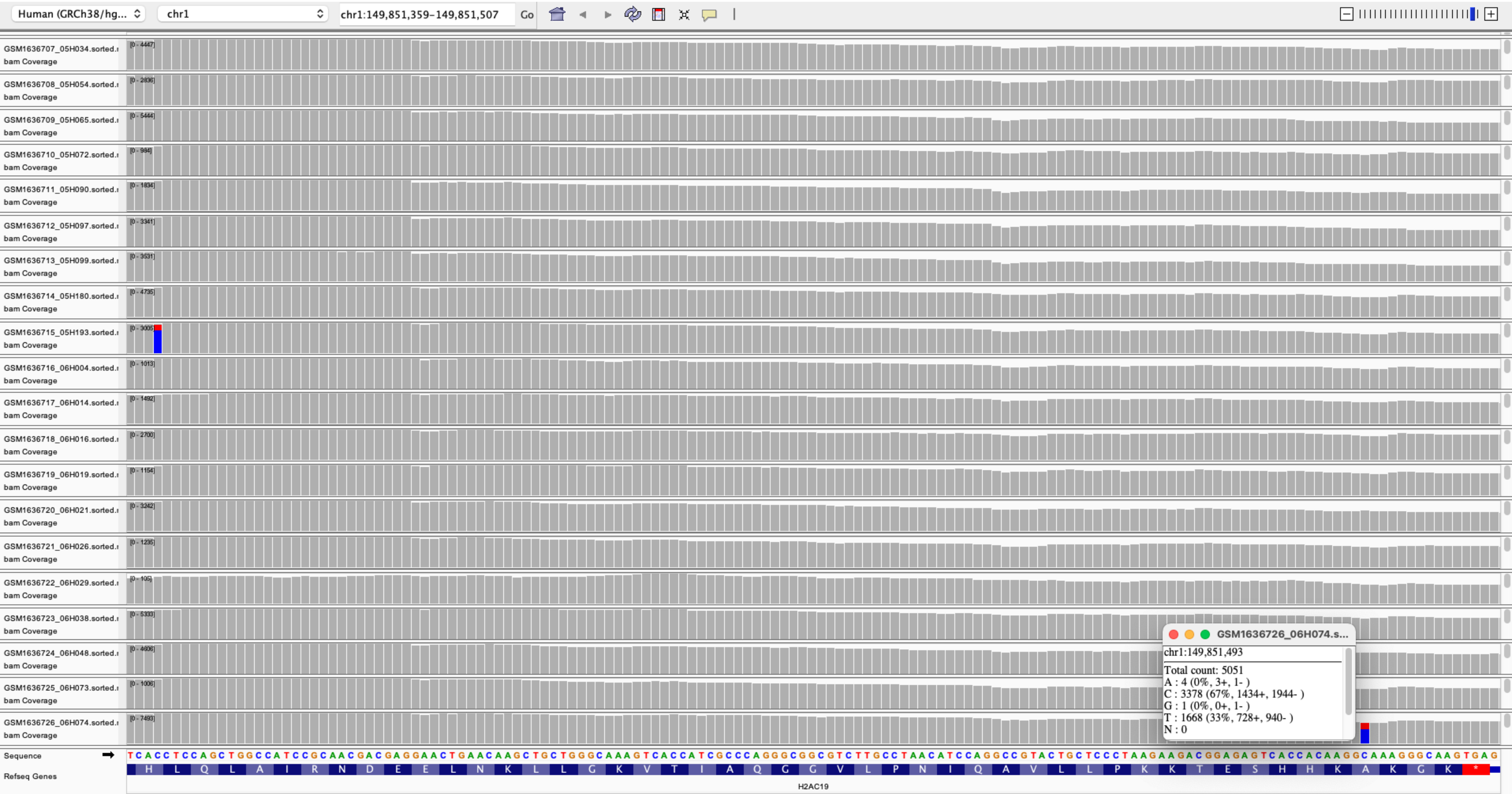

16.GSM1636727-GSM1636746

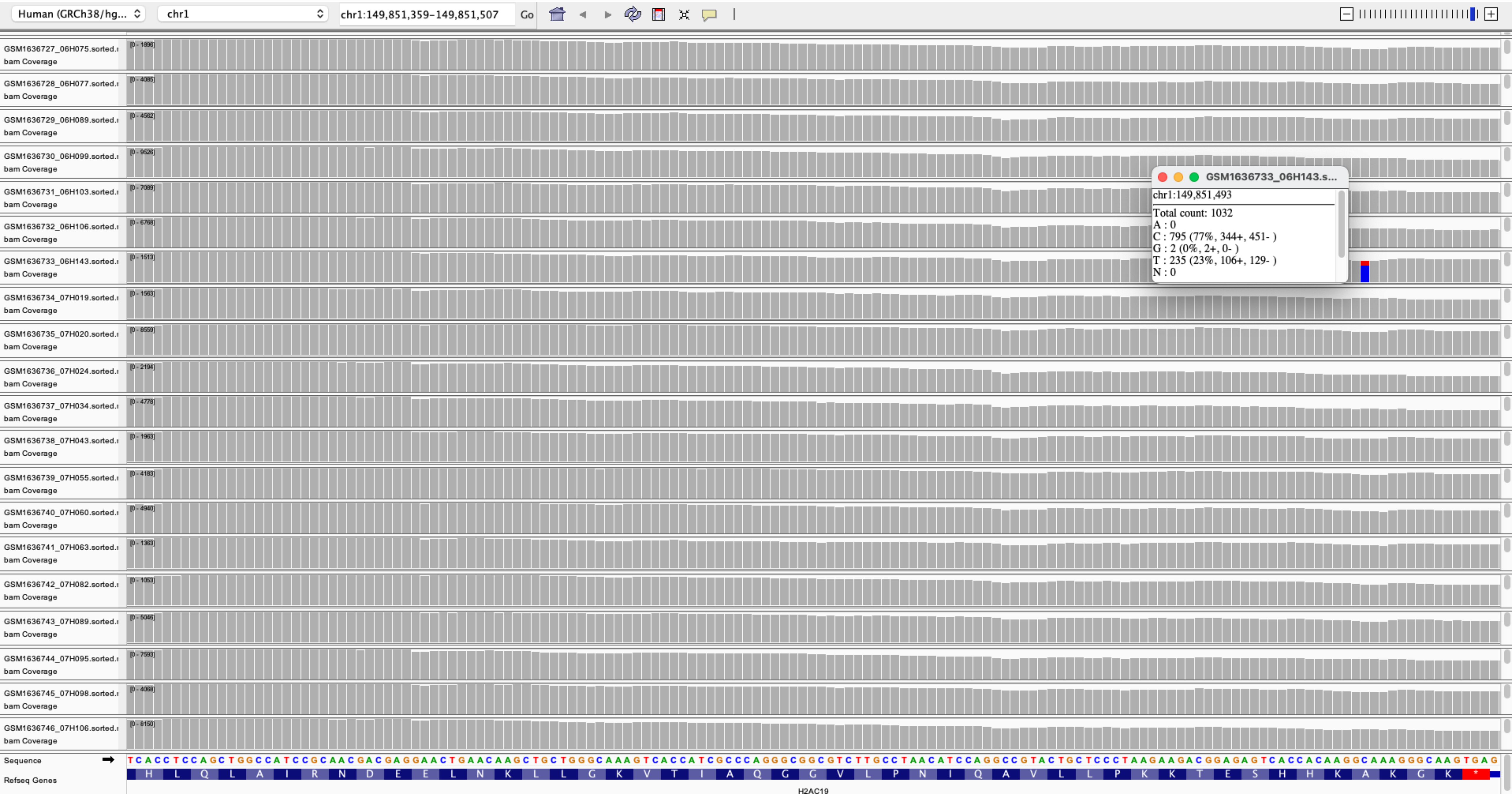

17.GSM1636747-GSM1636765

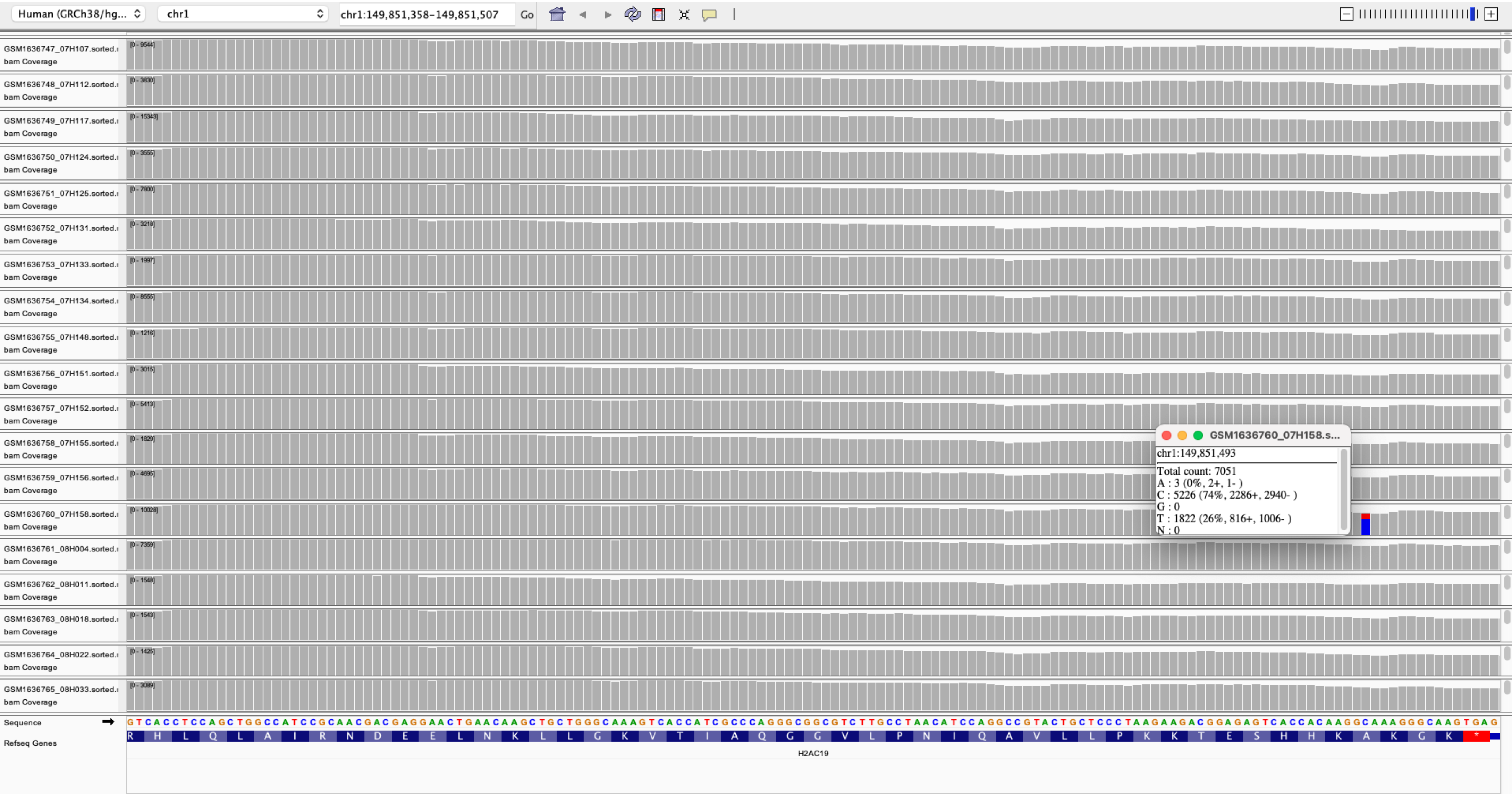

18.GSM1636766-GSM1636785

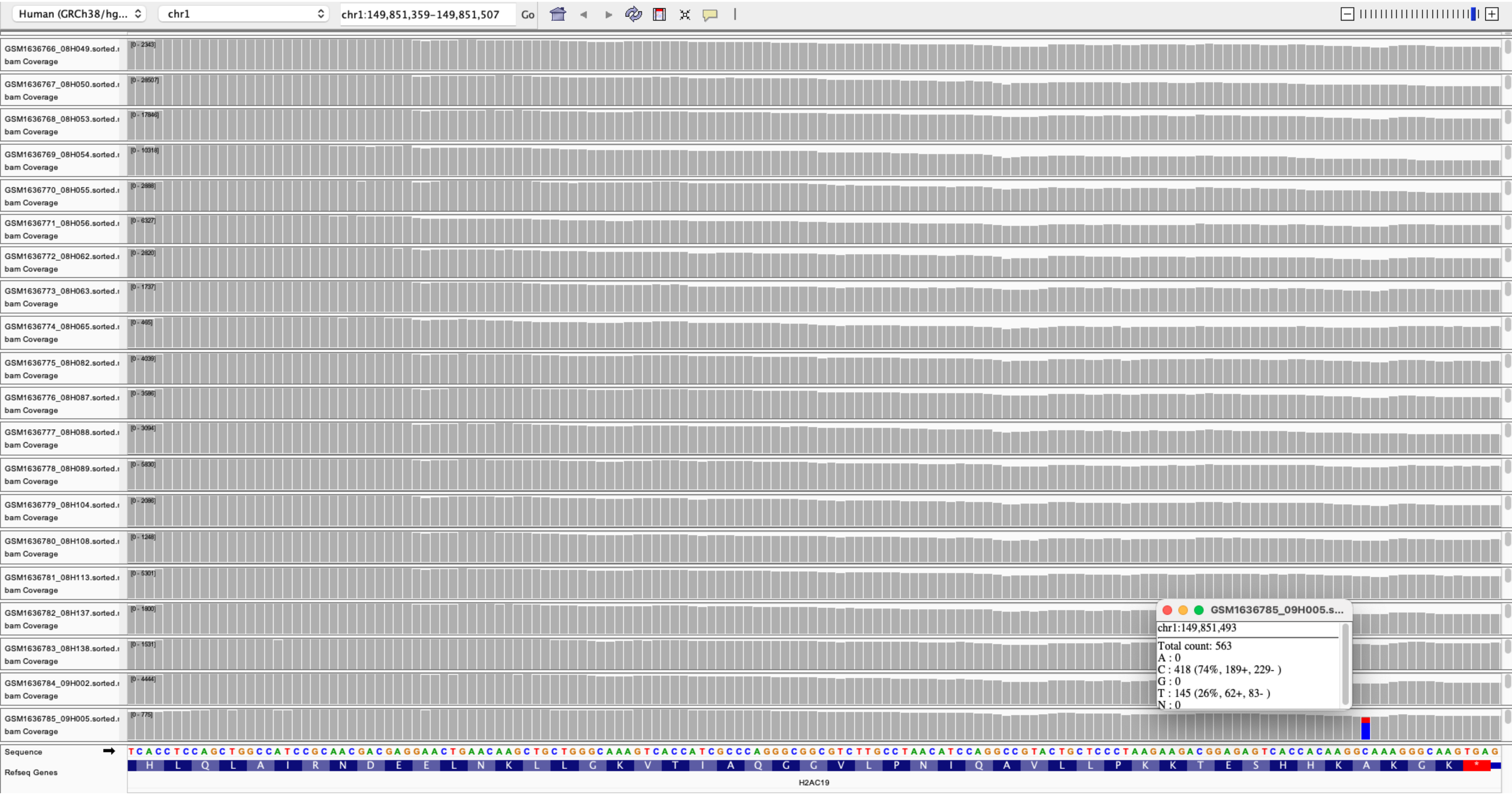

19.GSM1636786-GSM1636805

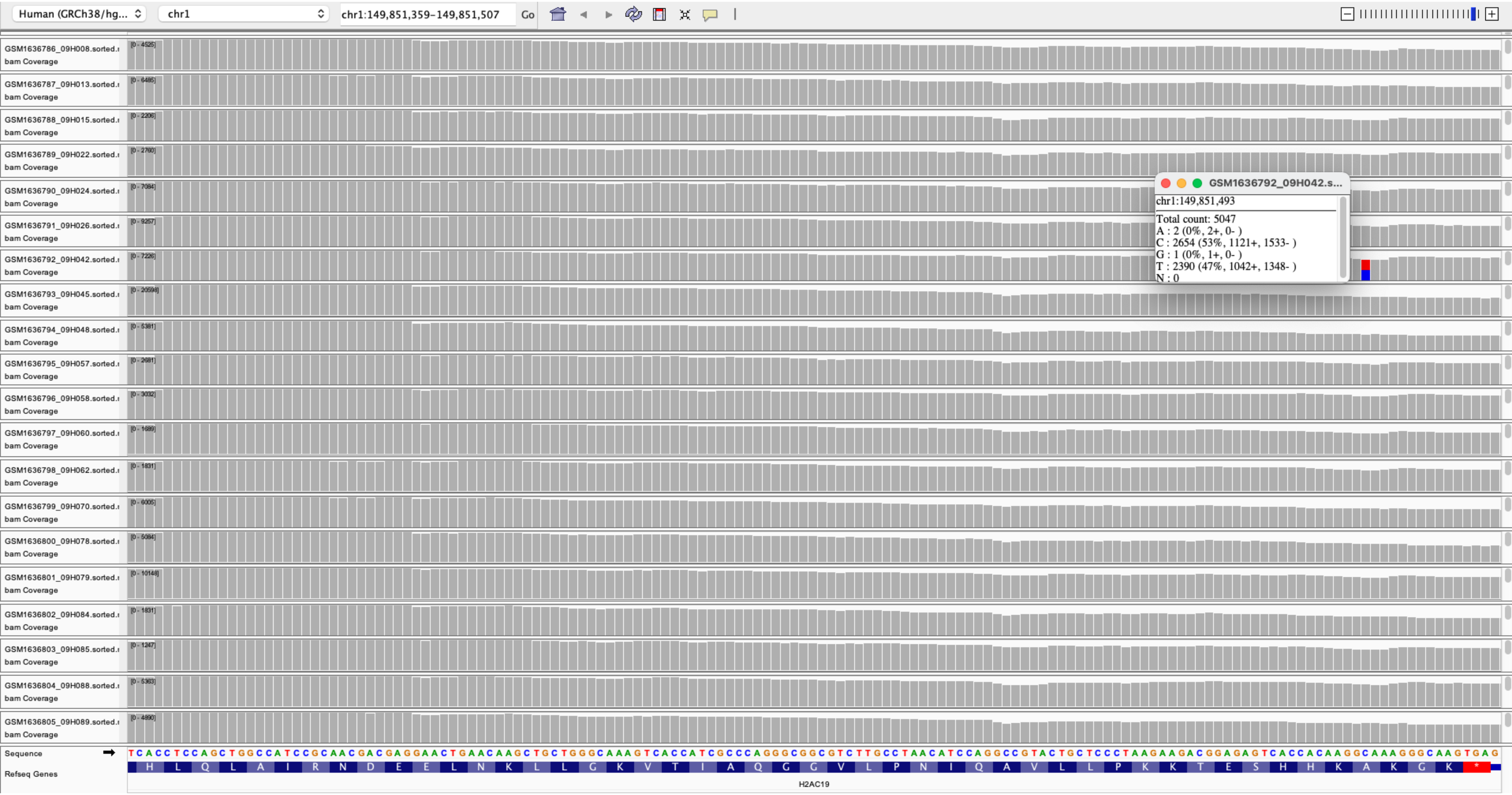

20.GSM1636806-GSM1636825

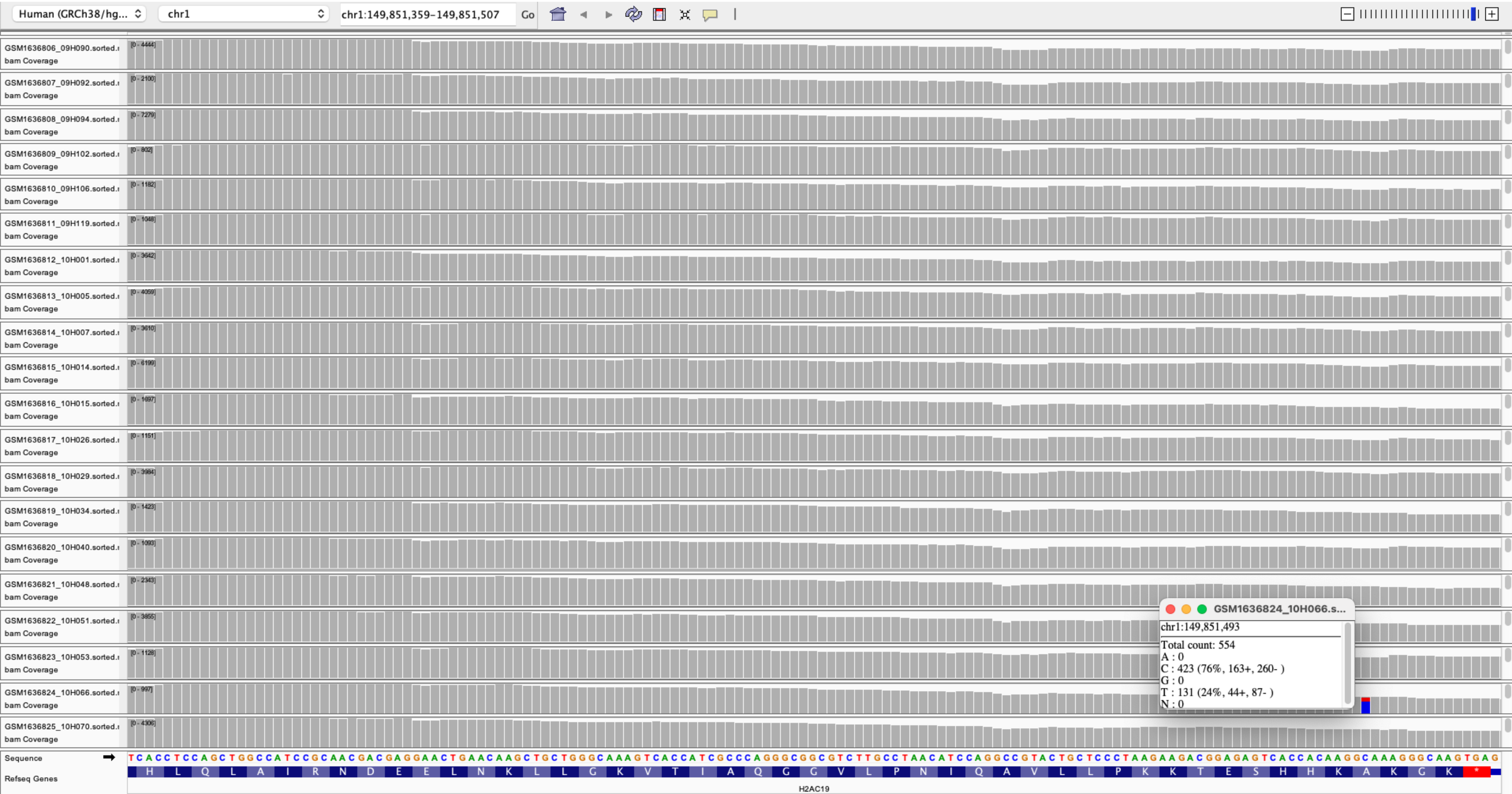

21.GSM1636826-GSM1636845

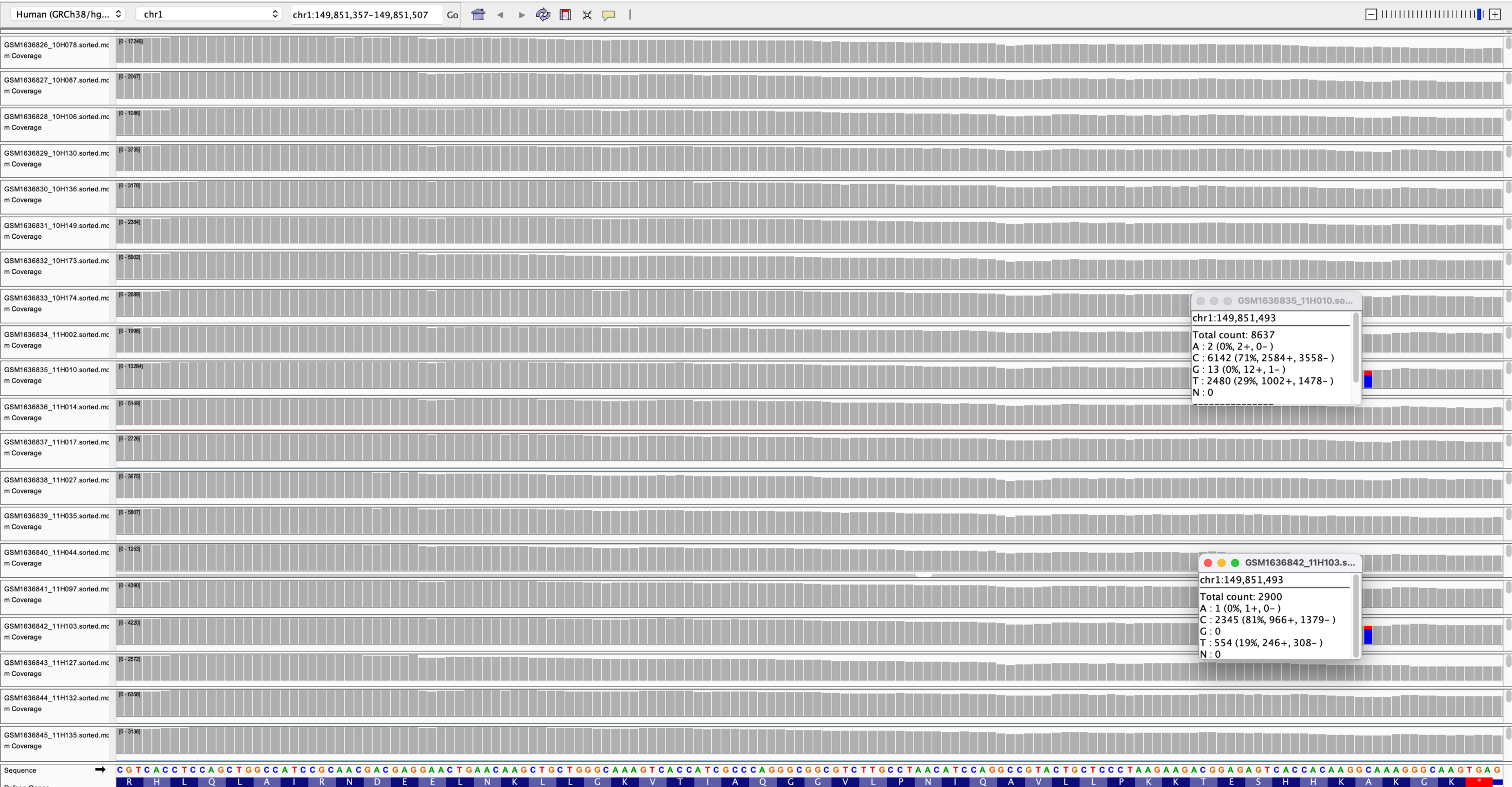

22.GSM1636846-GSM1636865

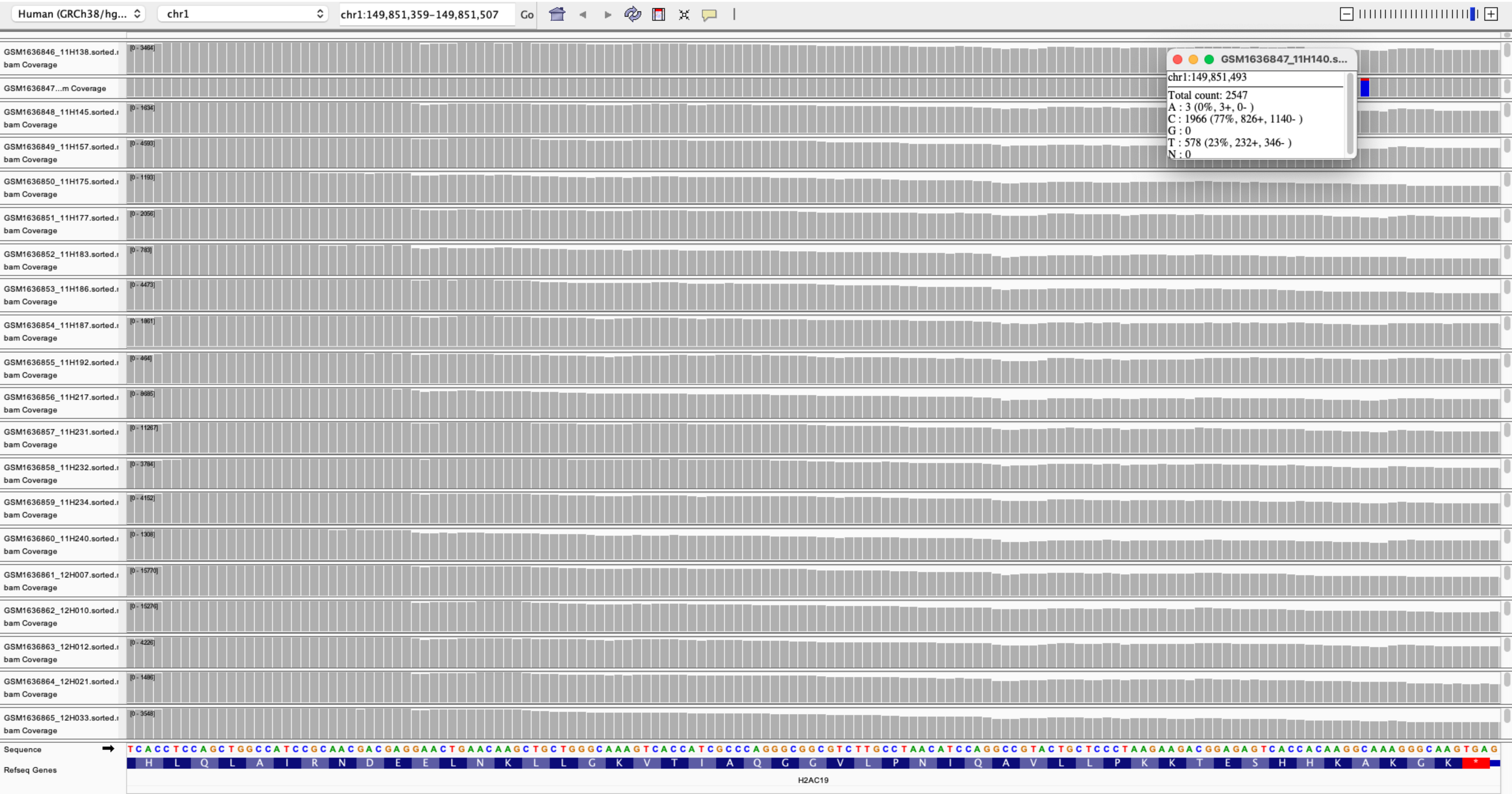

23.GSM1636866-GSM1636885

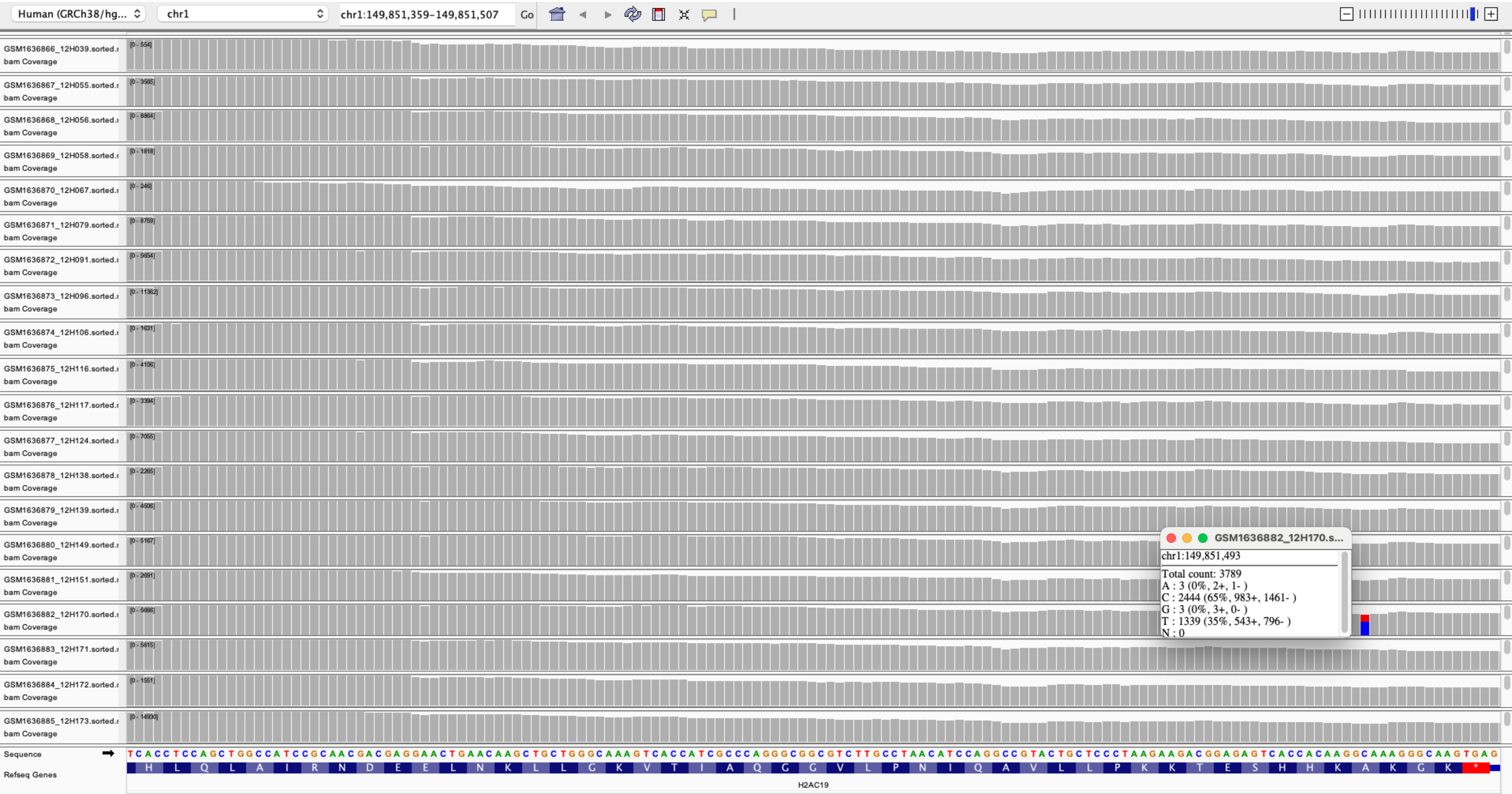

24.GSM1636886-GSM1636905

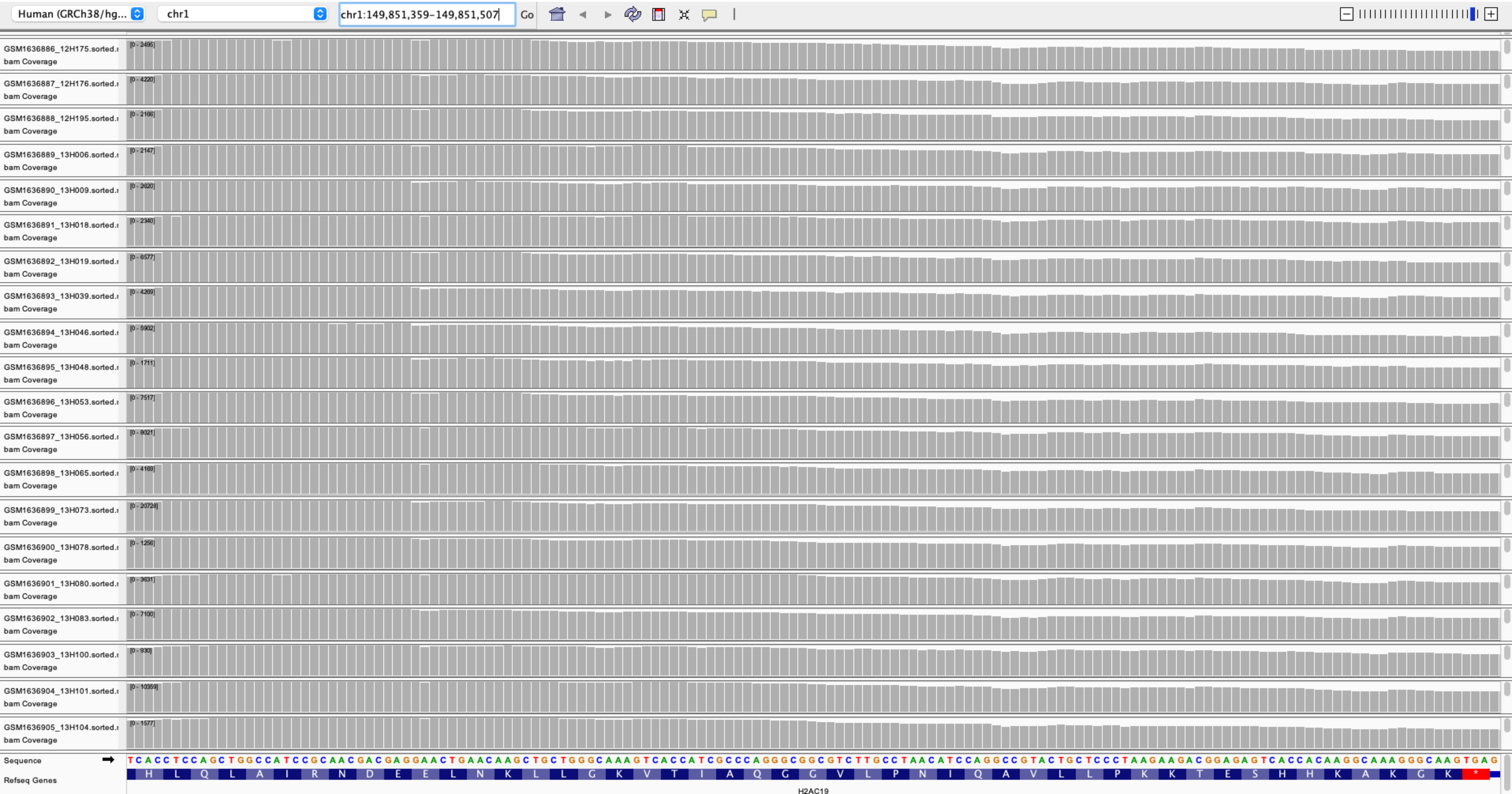

25.GSM1636906-GSM1636925

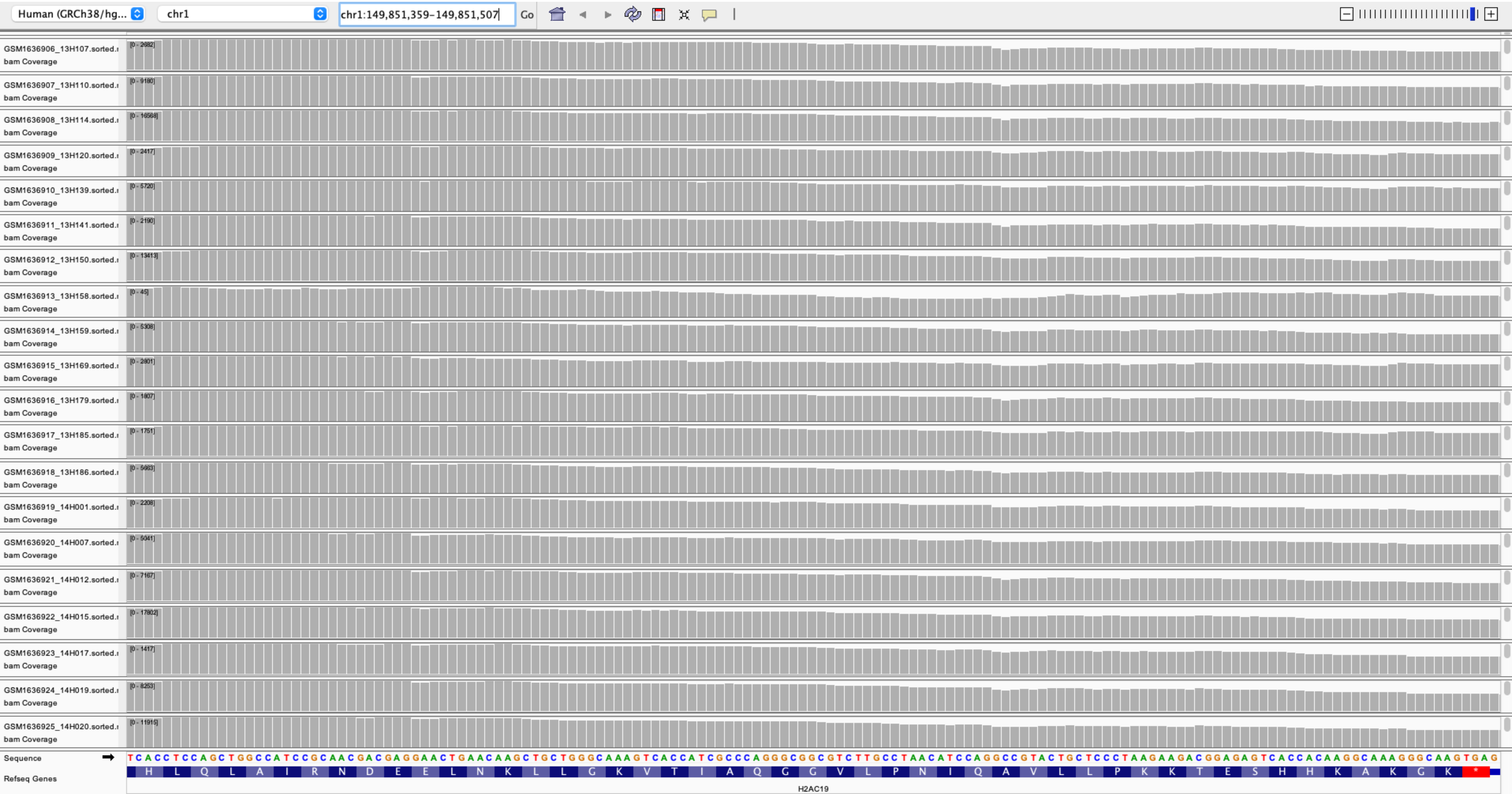

25.GSM1636926-GSM1636929

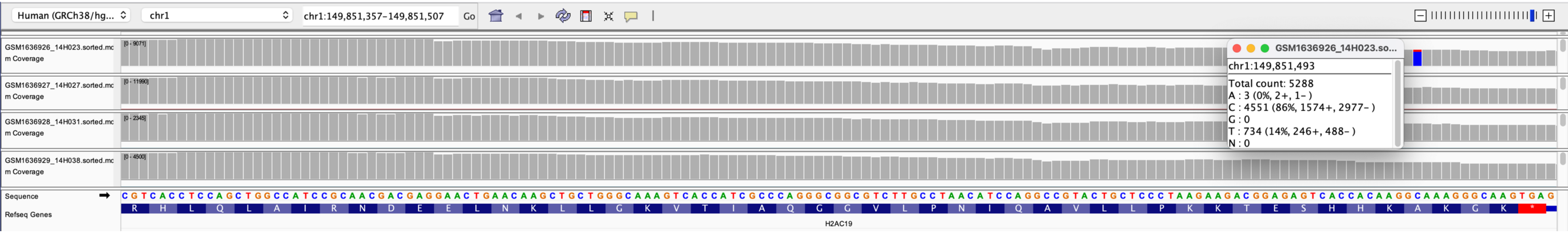

Supplement: Supplementary Figure 1 — Coverage tracks for the 5 AML samples carrying A127V in the validation cohort. [file Supplementaryfile1.pdf]
