# Supplementary material for: A novel H2A-A127 variant is associated with human cancer and enhances tumor-related phenotypes in Drosophila melanogaster models
Source: Front Oncol. 2026 Jul 13;16:1814908. doi: 10.3389/fonc.2026.1814908 (PMC13402181; doi:10.3389/fonc.2026.1814908)

ACB: African Caribbean in Barbados
(96 samples)

ACB: HG01879-HG01985

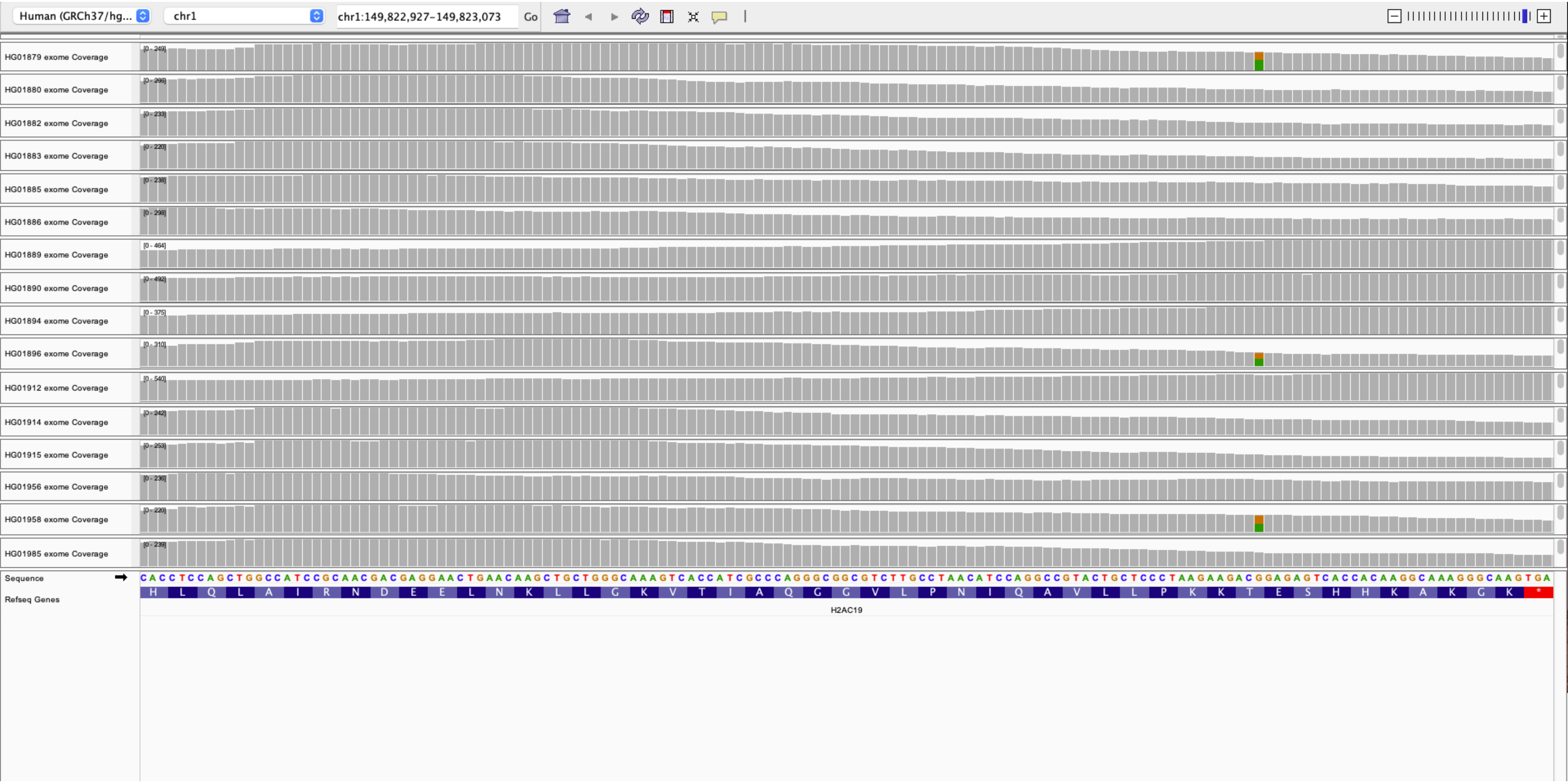

ACB: HG01986-HG02255

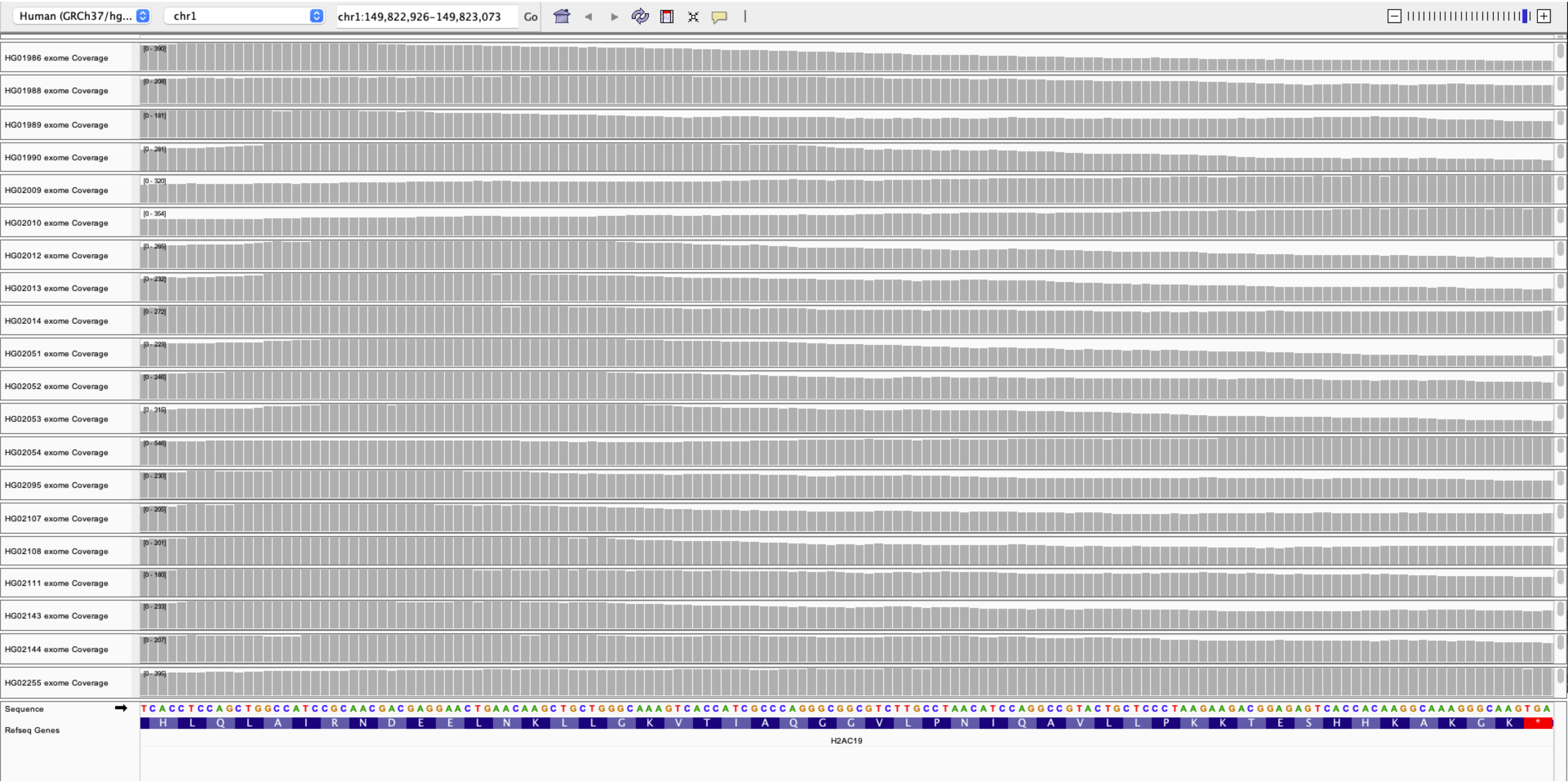

ACB: HG02256-HG02330

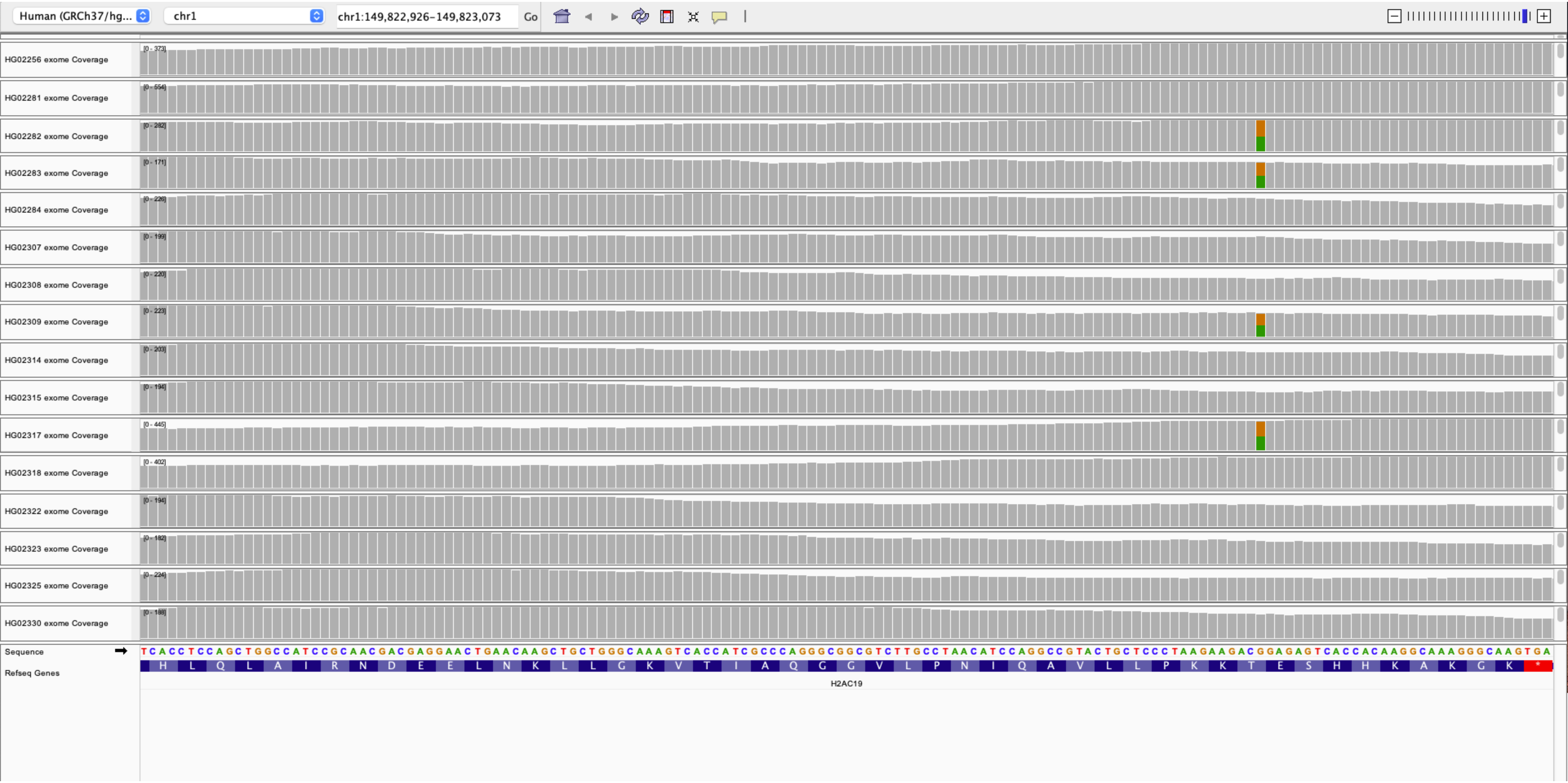

ACB: HG02332-HG02455

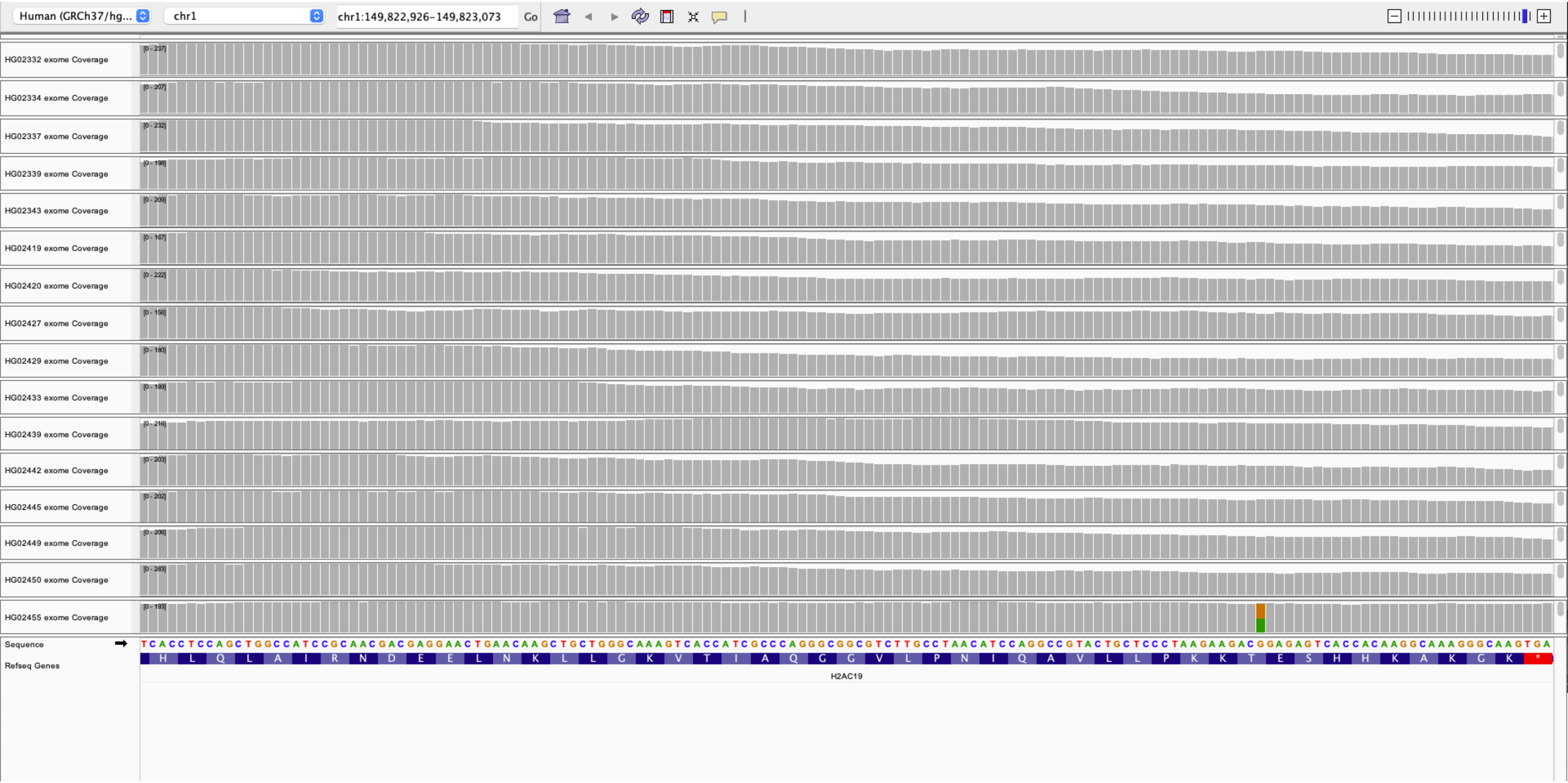

ACB: HG02470-HG02511

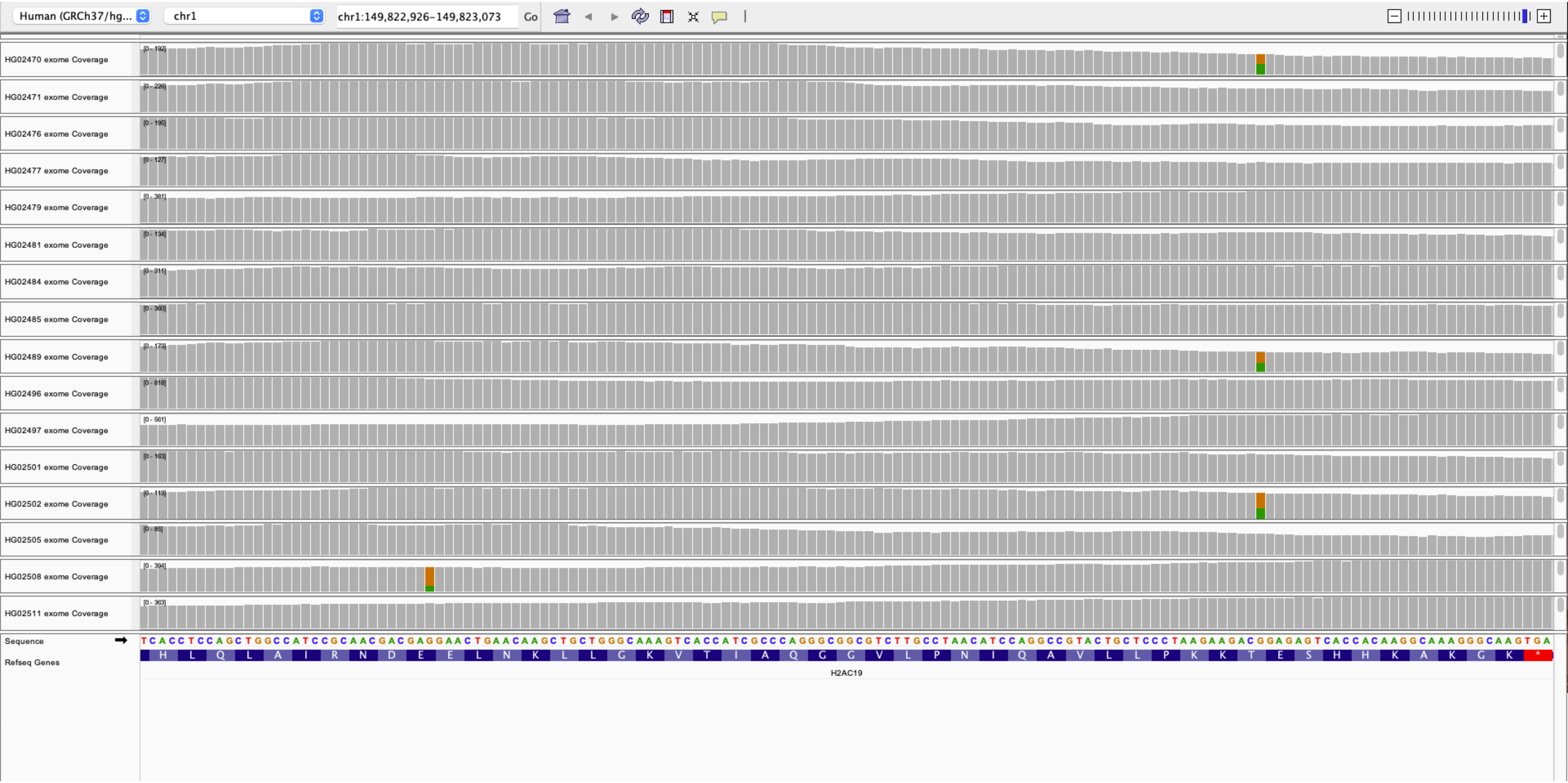

ACB: HG02536-HG02580

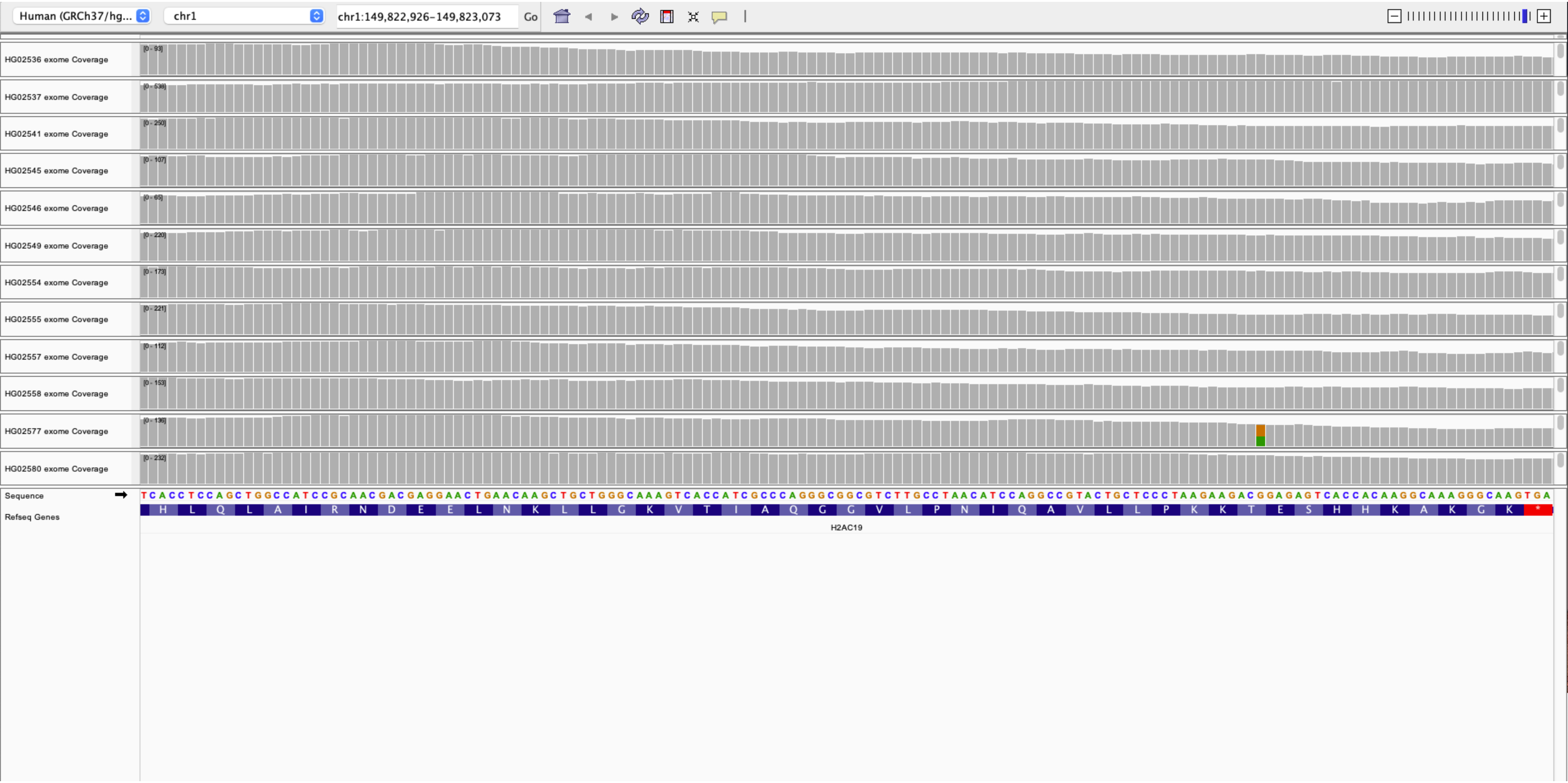

ASW: People with African Ancestry in Southwest USA (66 samples)

ASW: NA19625-NA19904

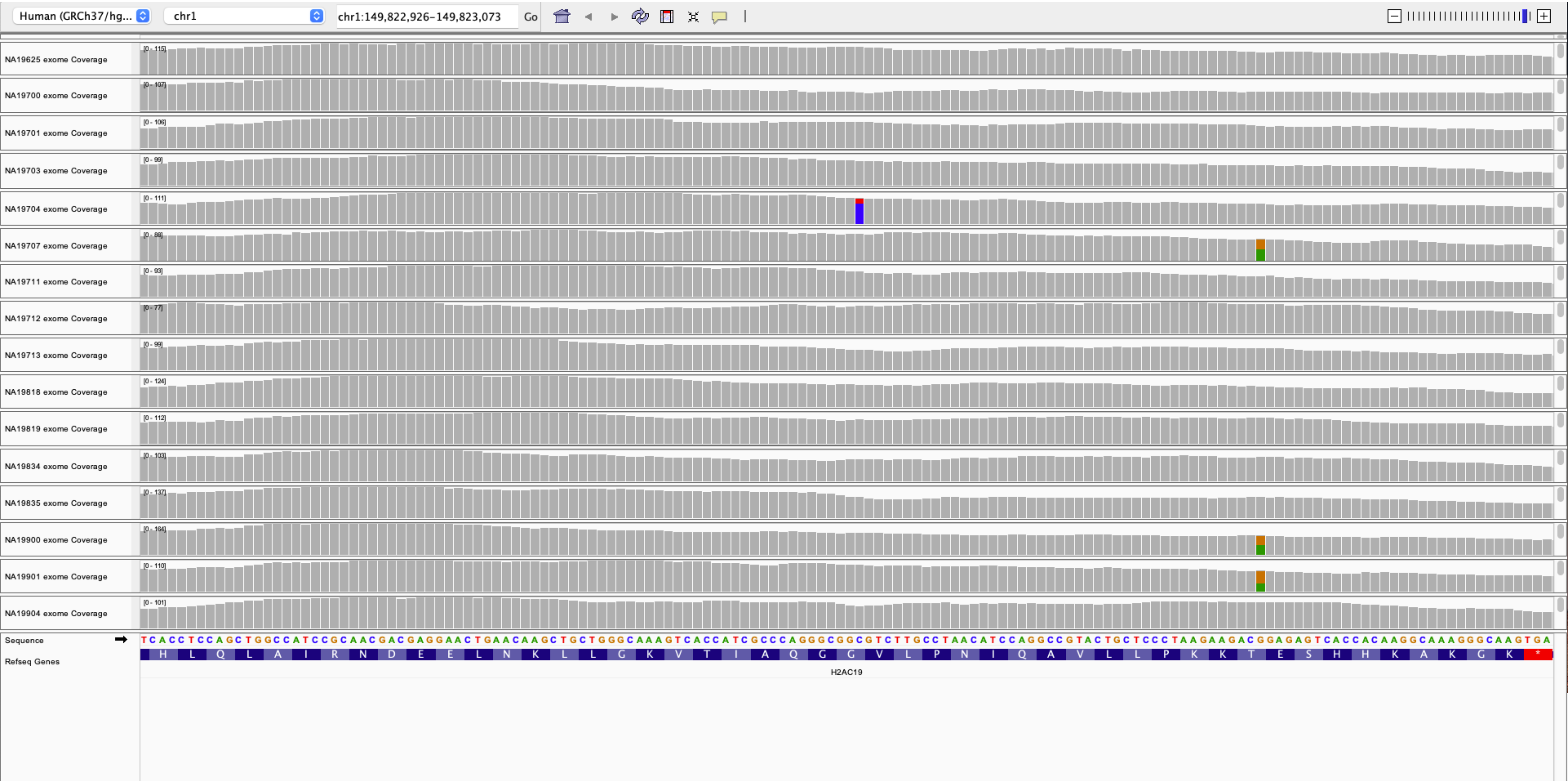

ASW: NA19908-NA20274

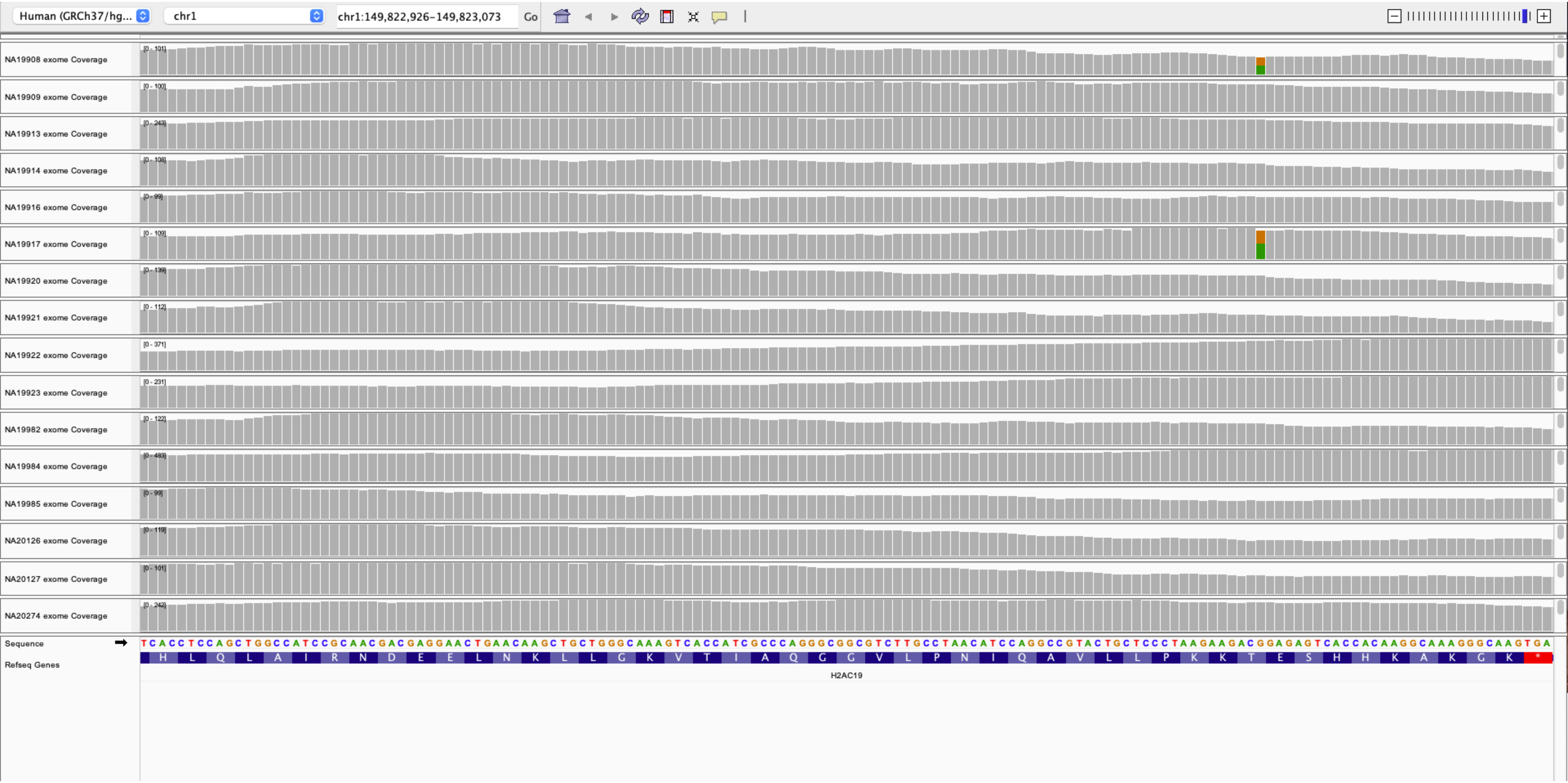

ASW: NA20276-NA20321

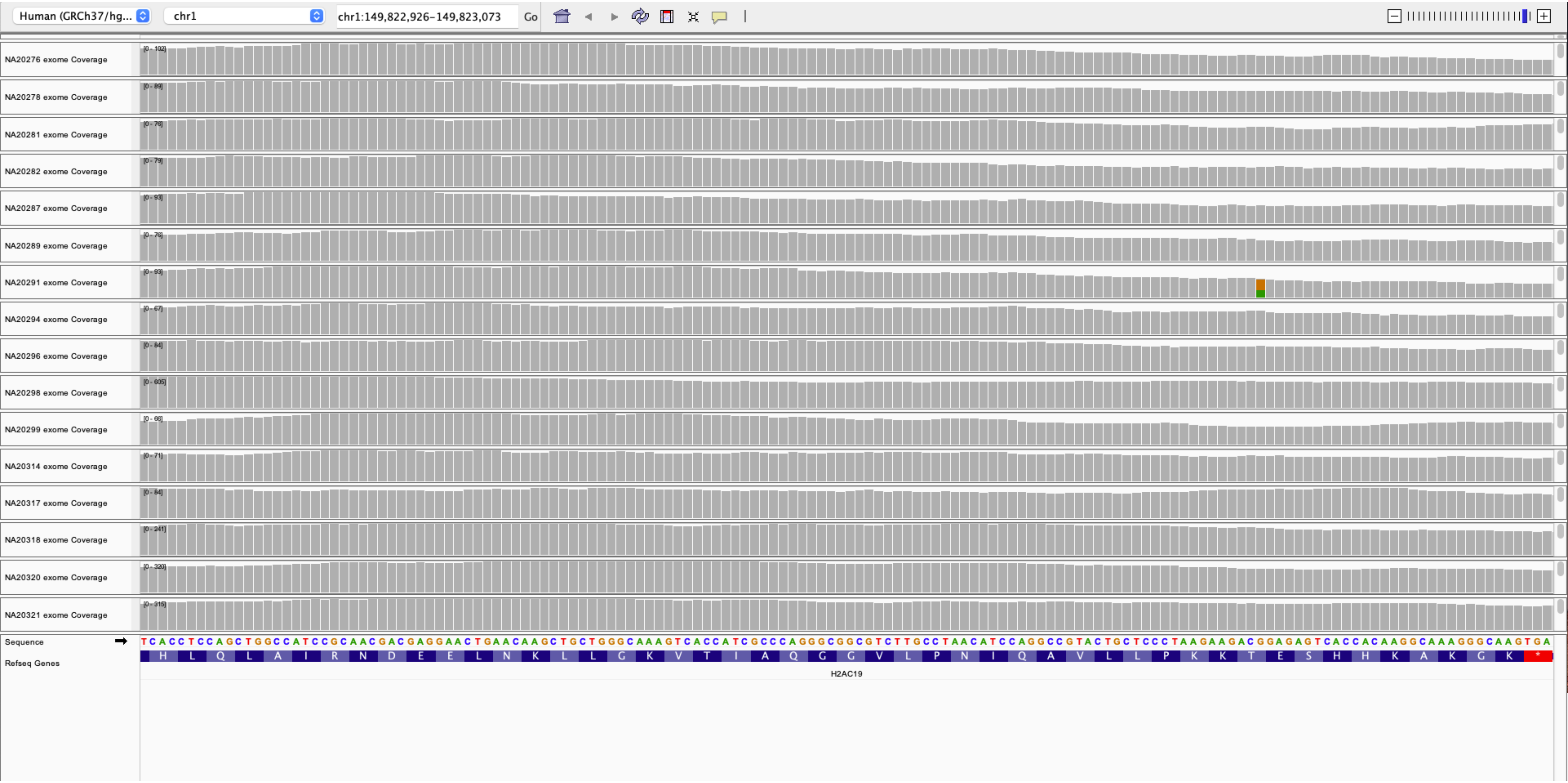

ASW: NA20322-NA20412

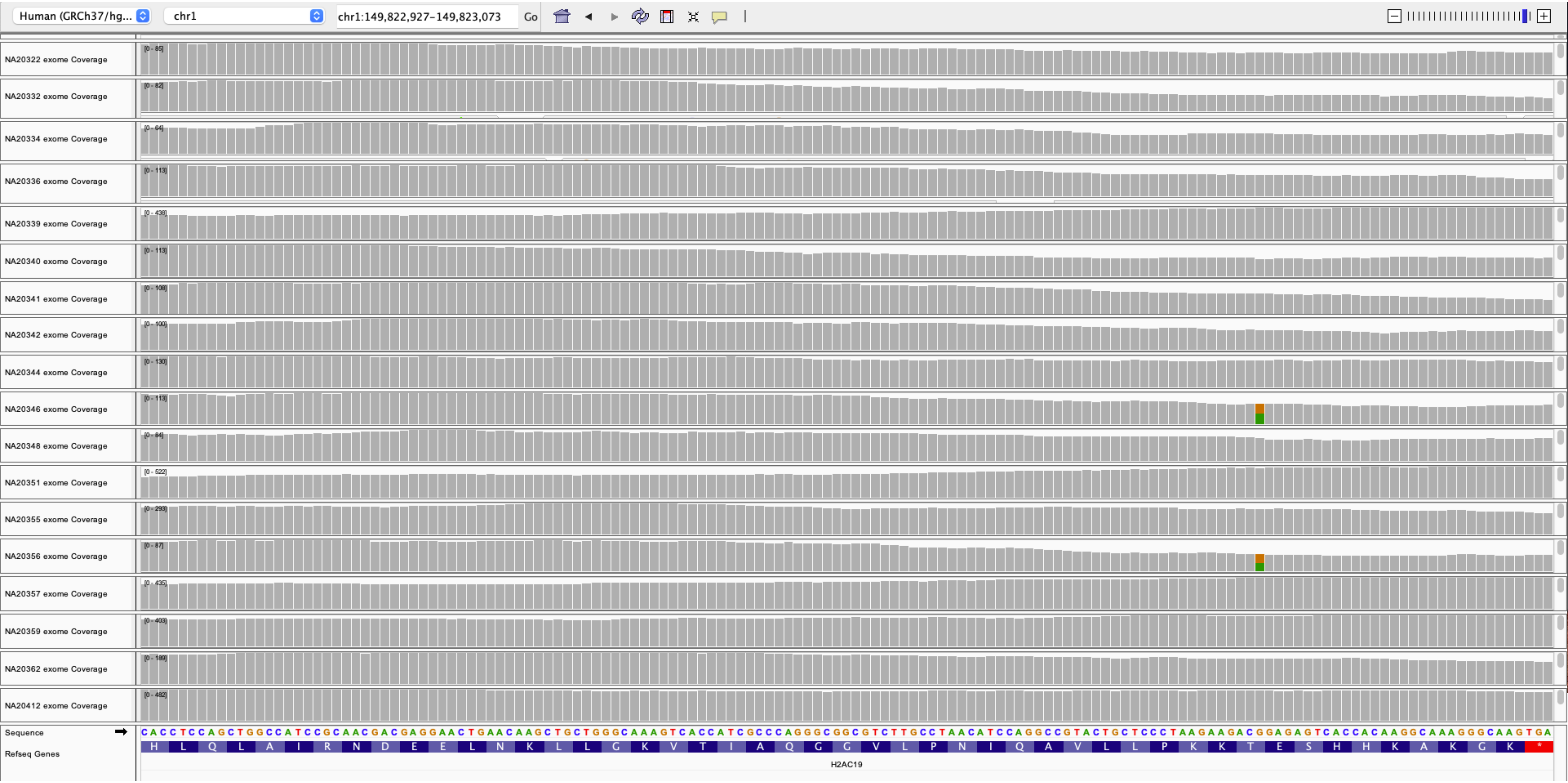

BEB: Bengali in Bangladesh (86 samples)

BEB: HG03006-HG03793

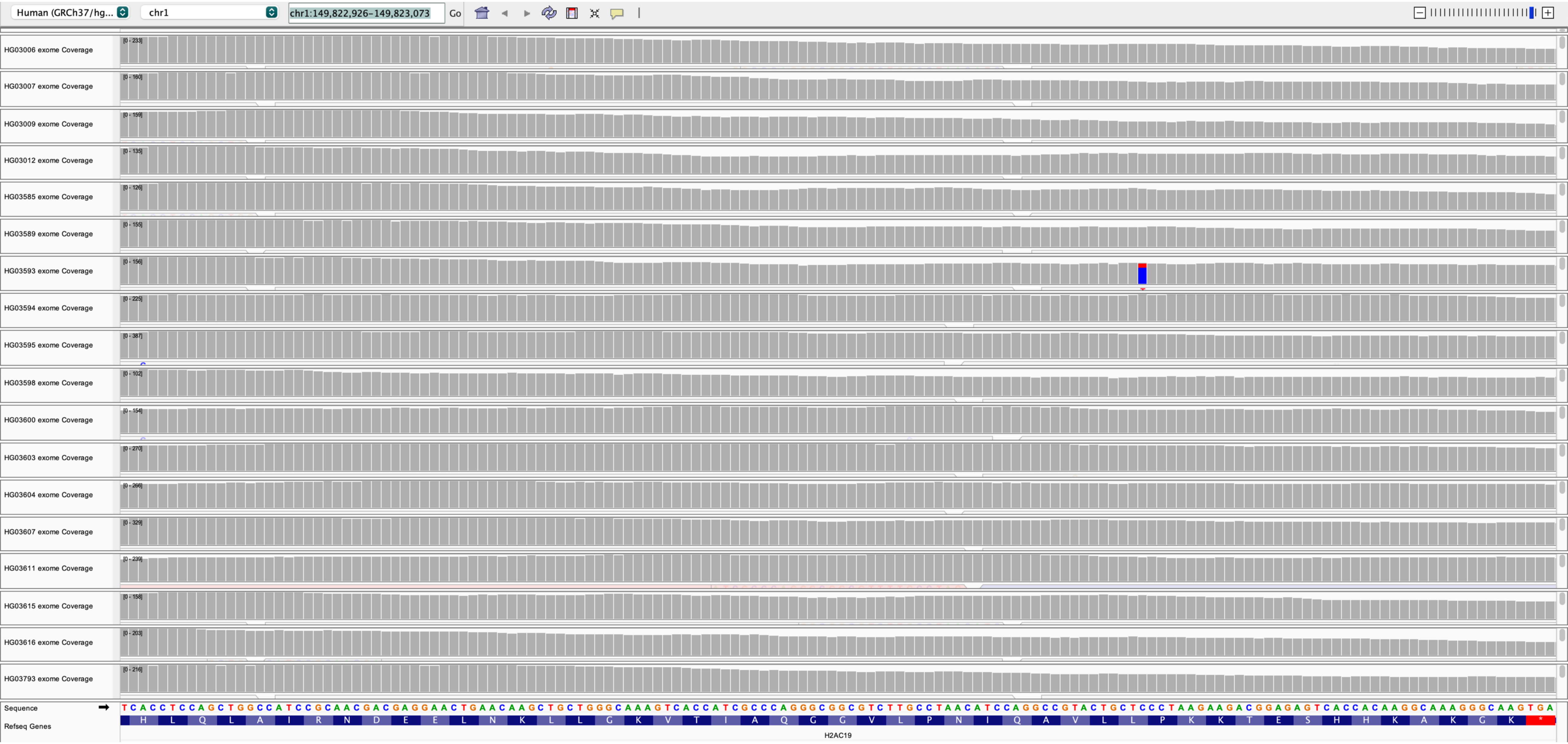

BEB: HG03796-HG03832

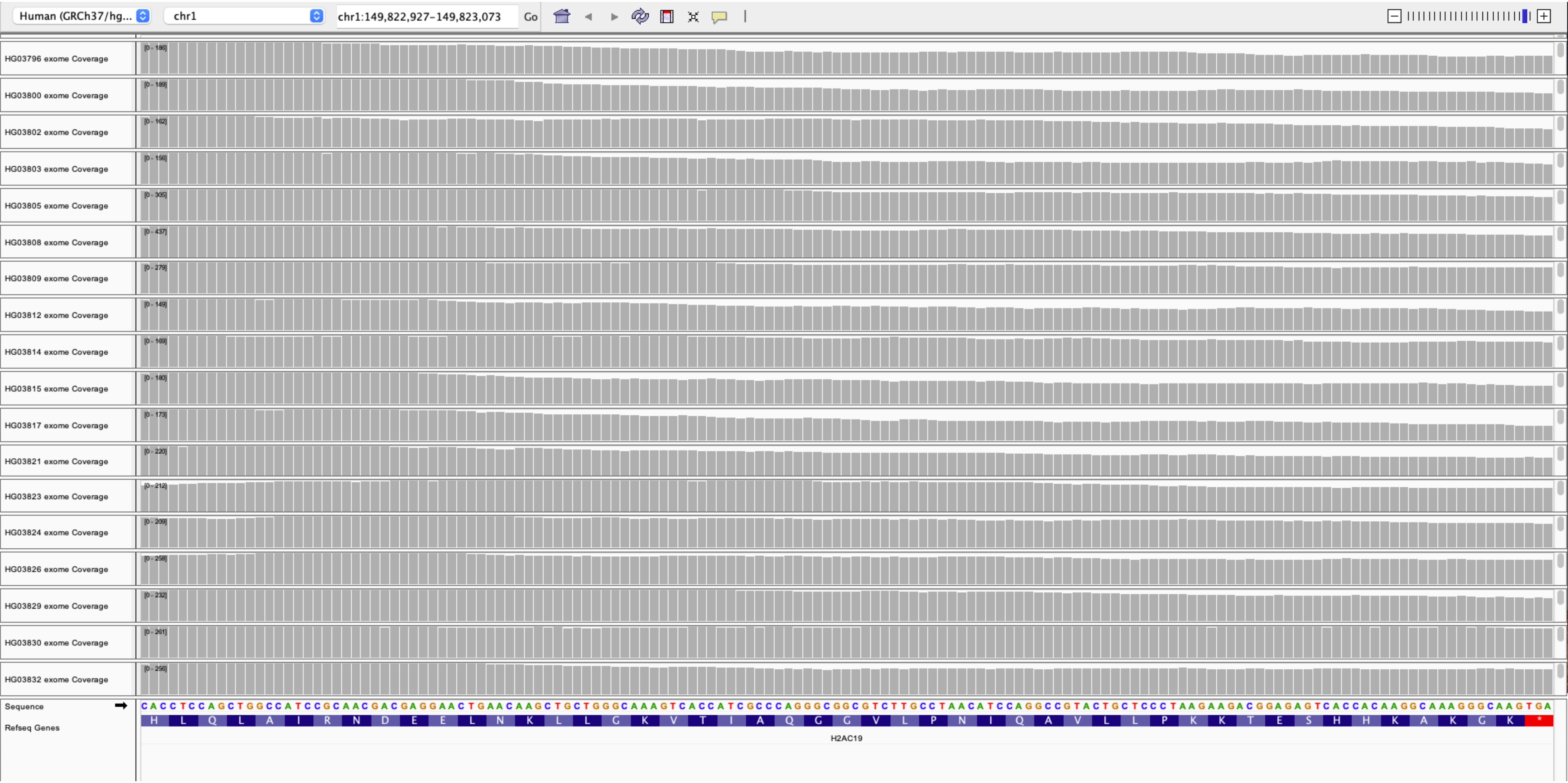

BEB: HG03833-HG03931

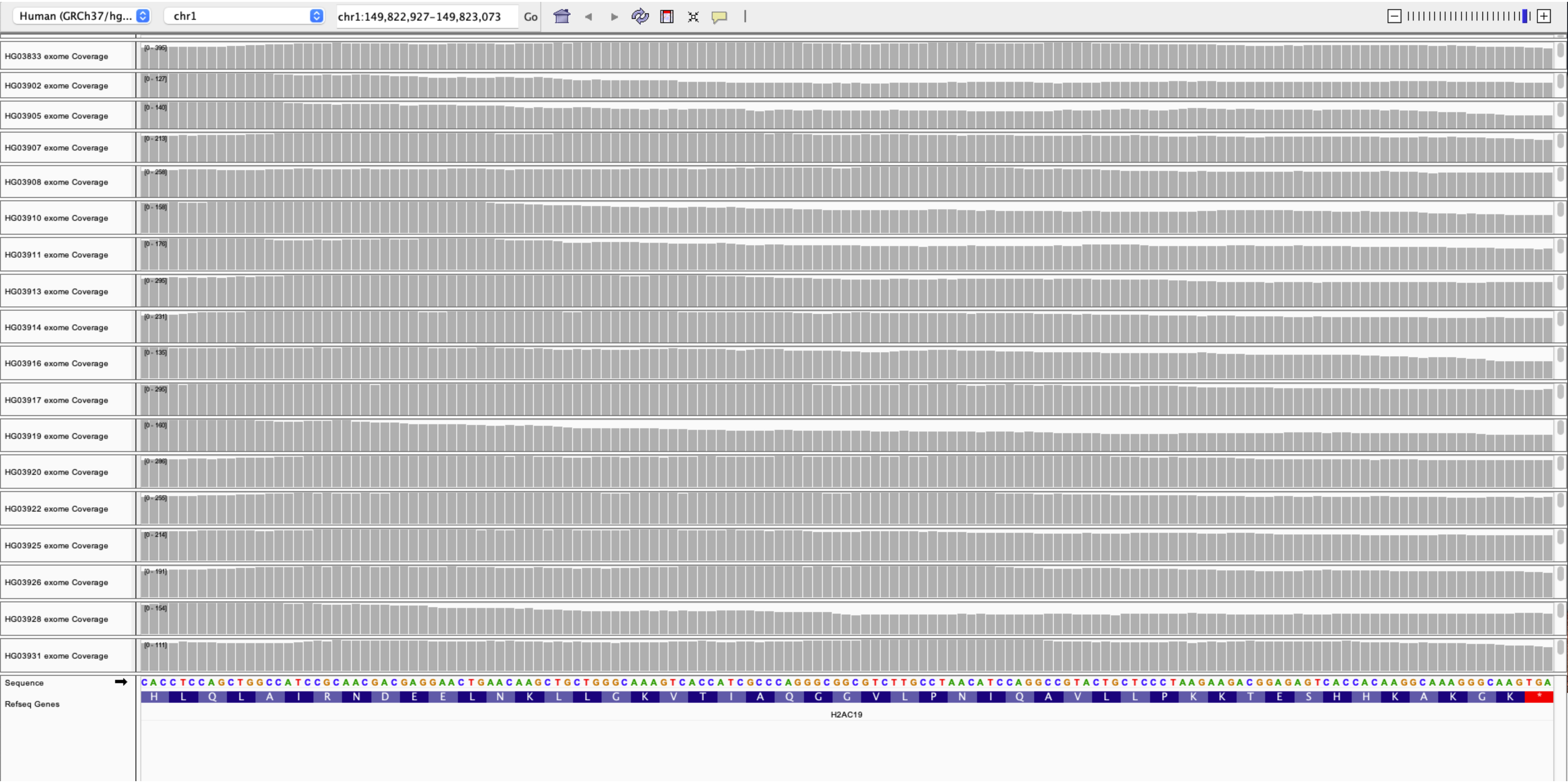

BEB: HG03934-HG04162

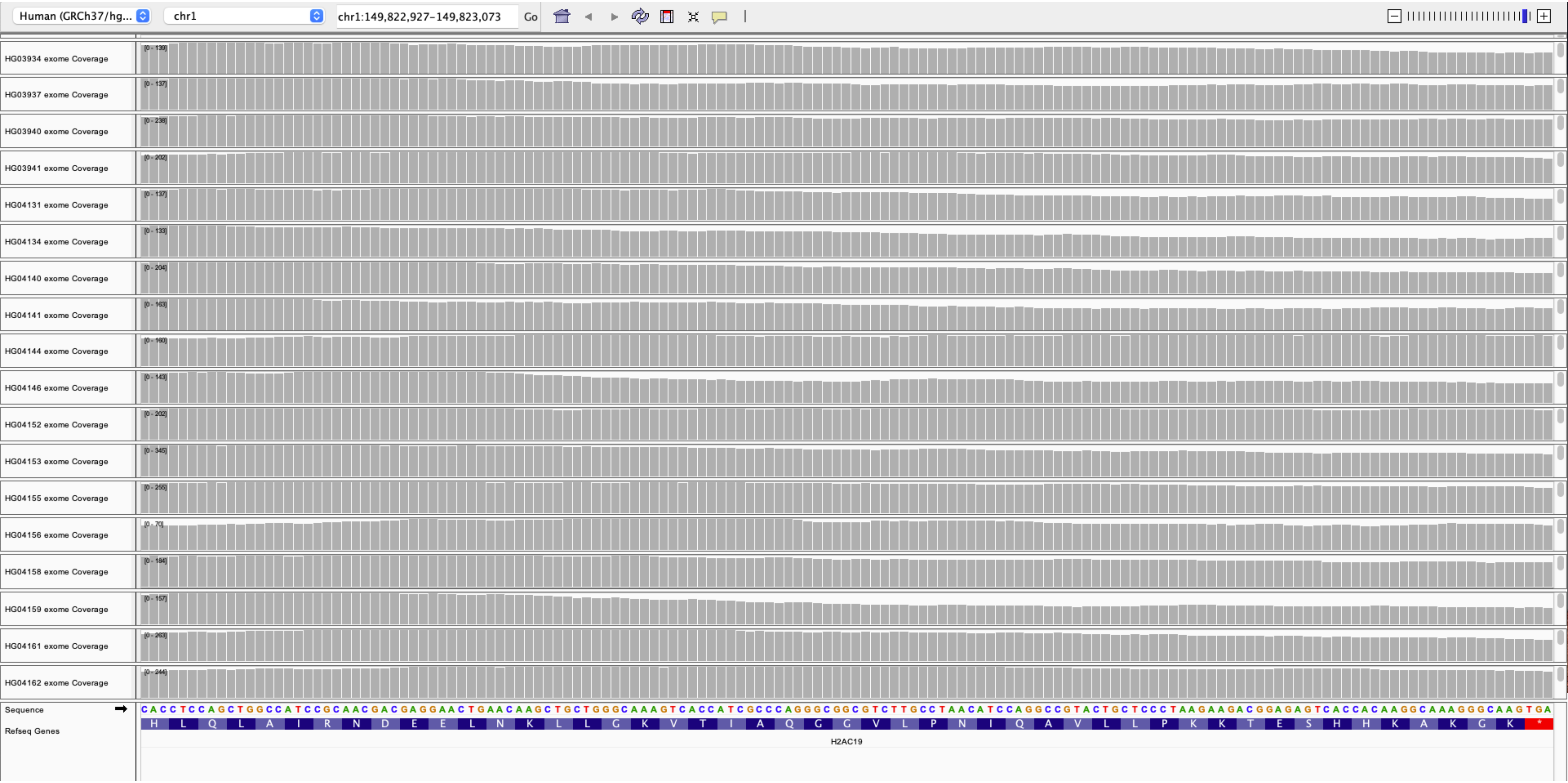

BEB: HG04164-HG04195

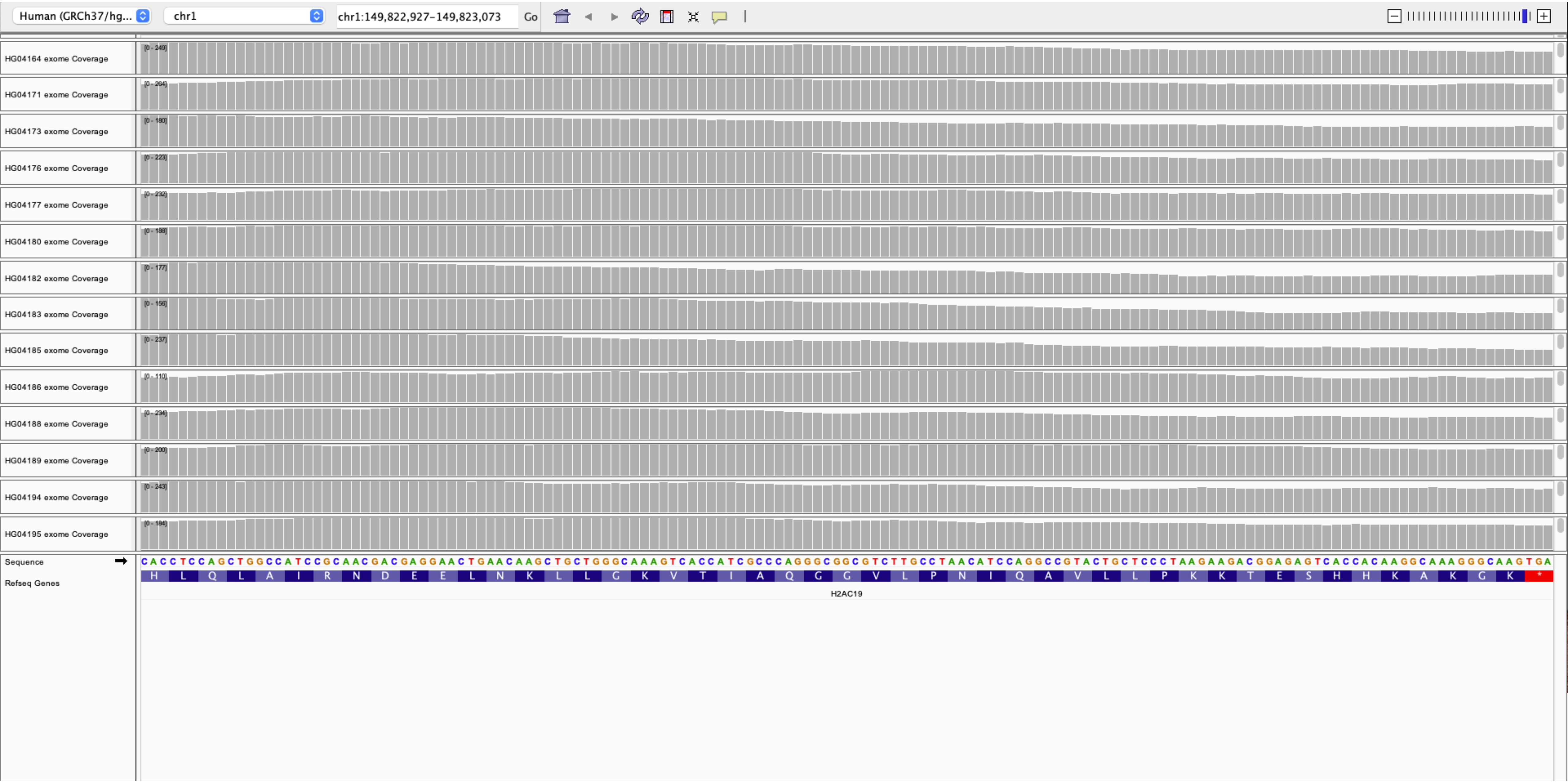

CDX: Chinese Dai in Xishuangbanna, China (99 samples)

CDX: HG00759-HG01796

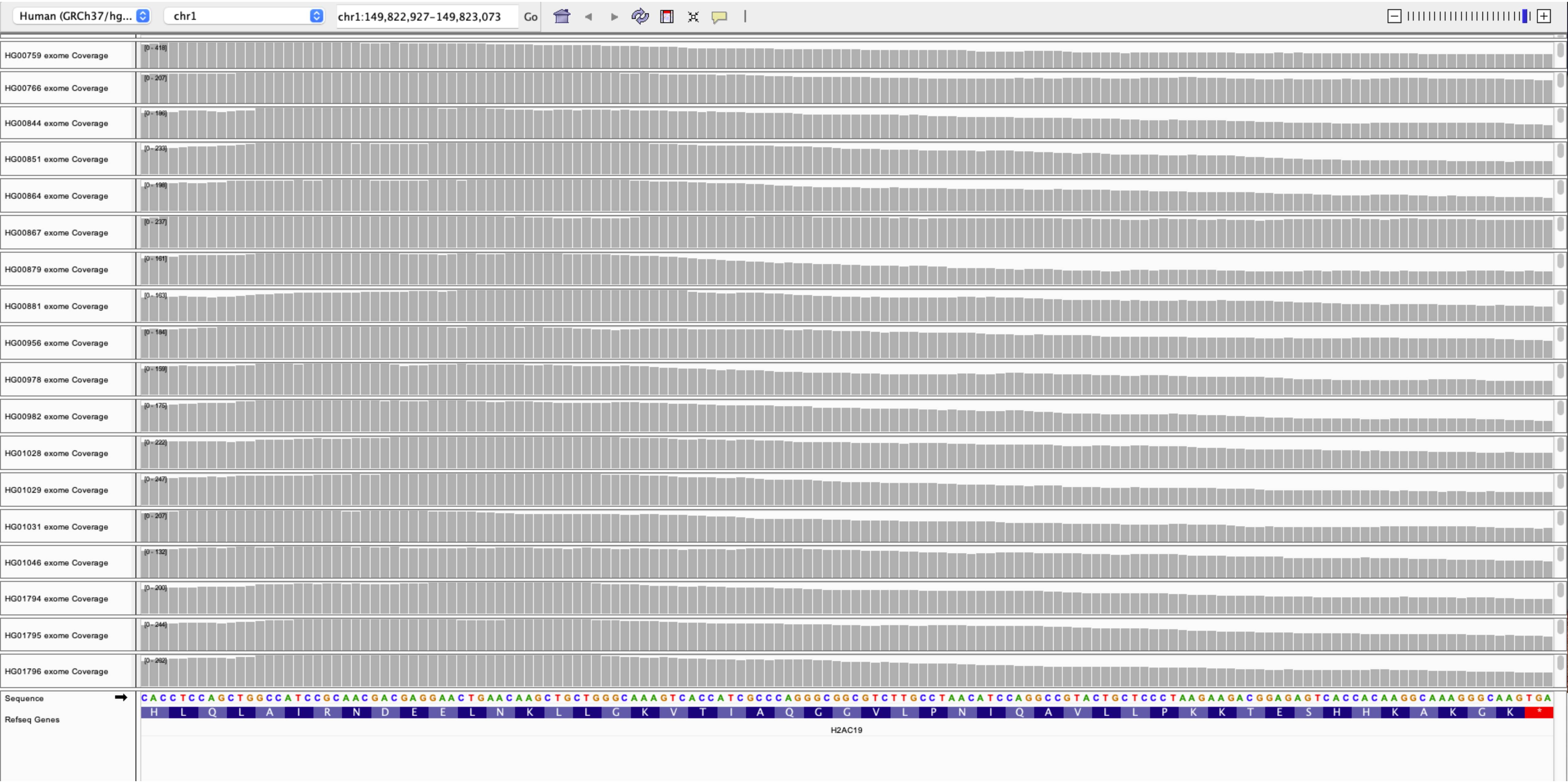

CDX: HG01797-HG01816

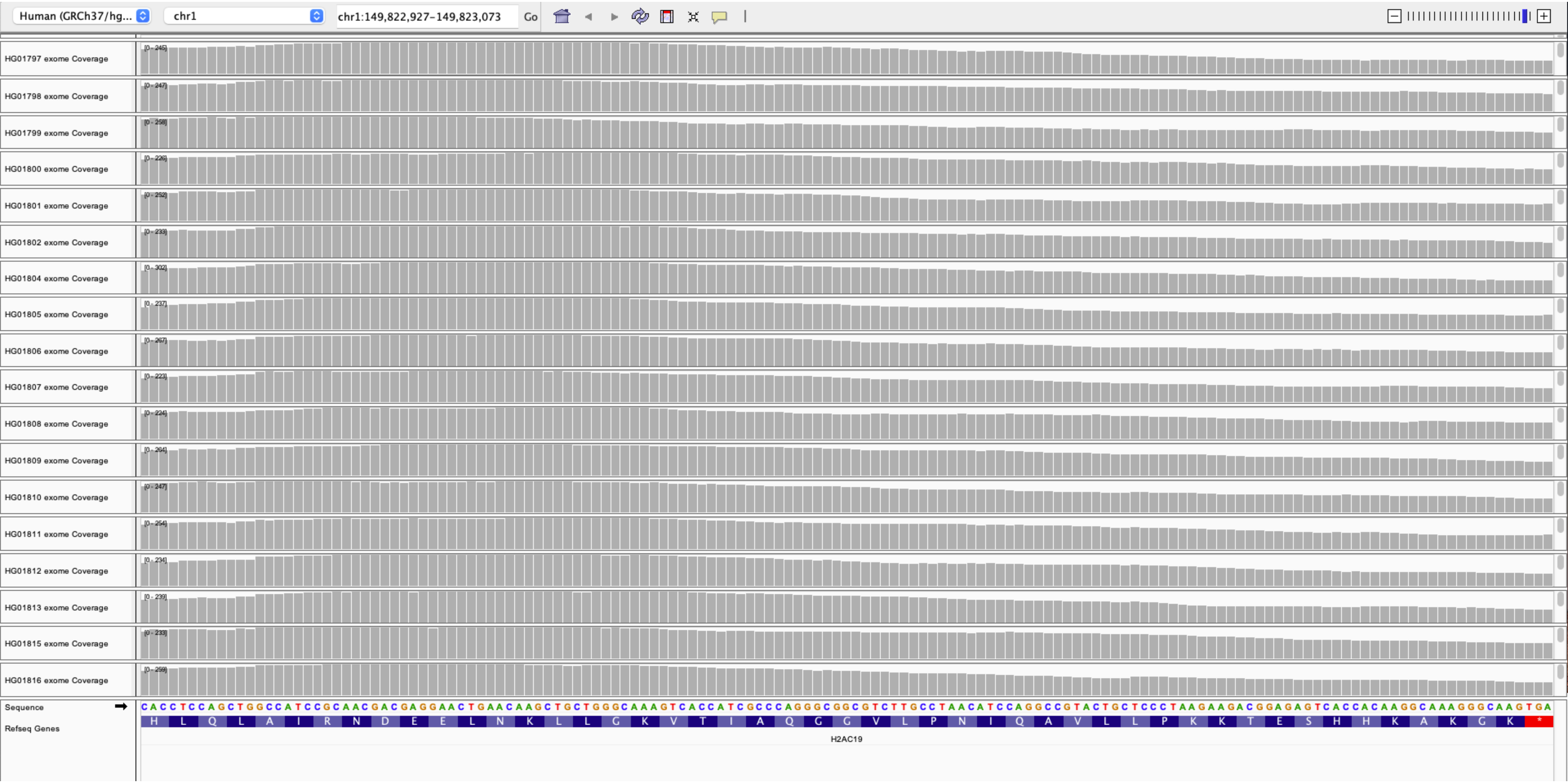

CDX: HG01817-HG02186

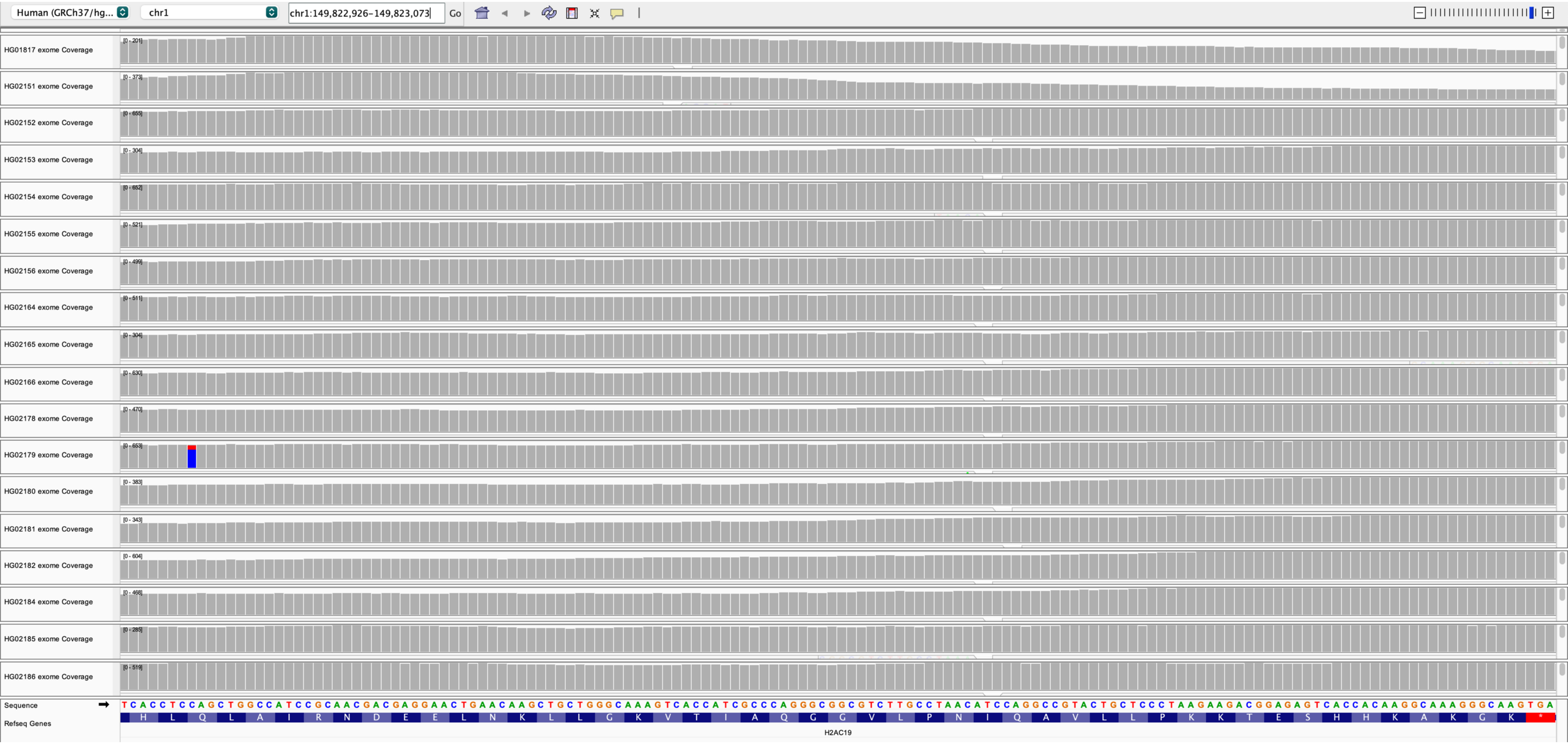

CDX: HG02187-HG02377

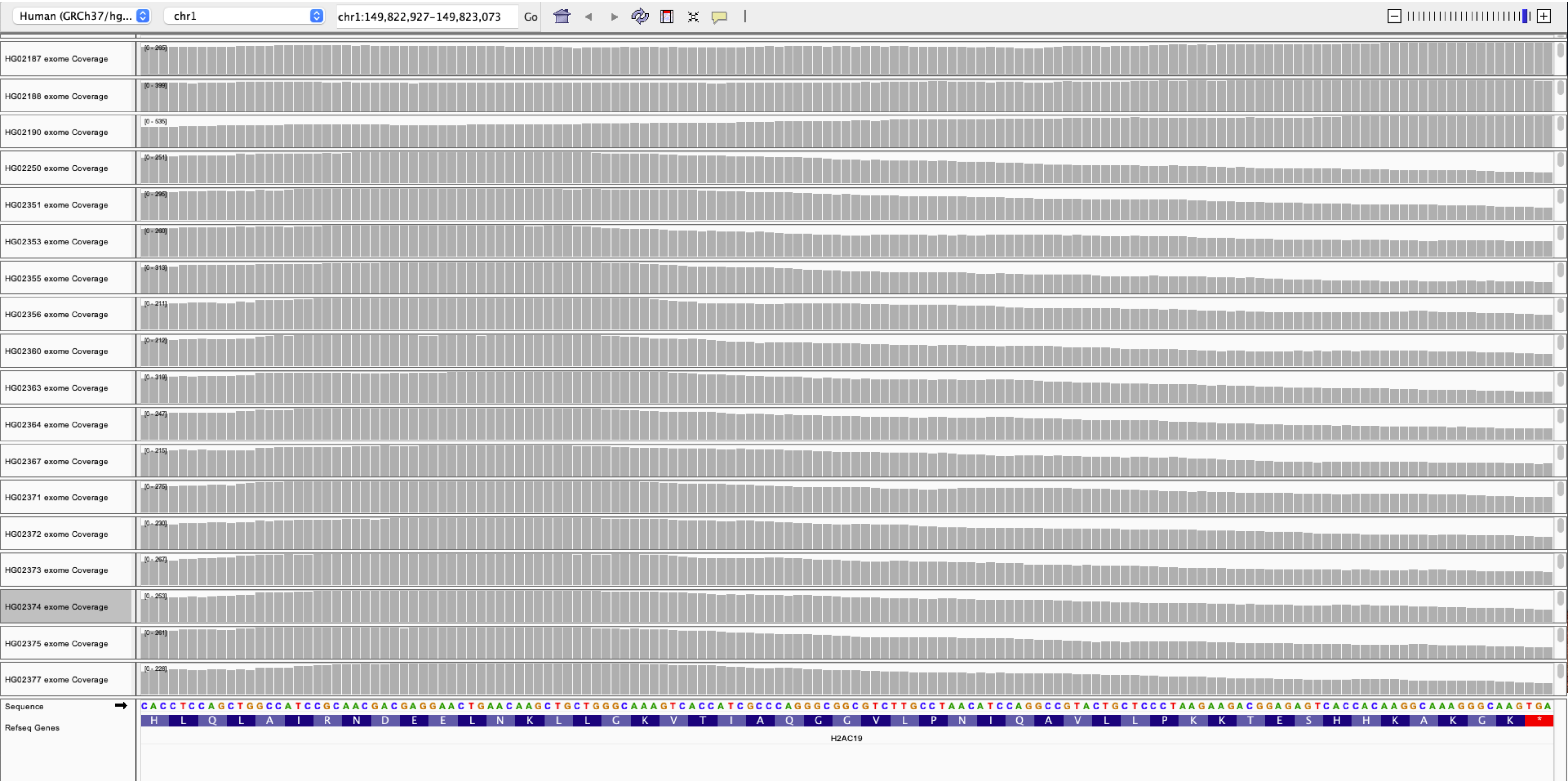

CDX: HG02379-HG02397

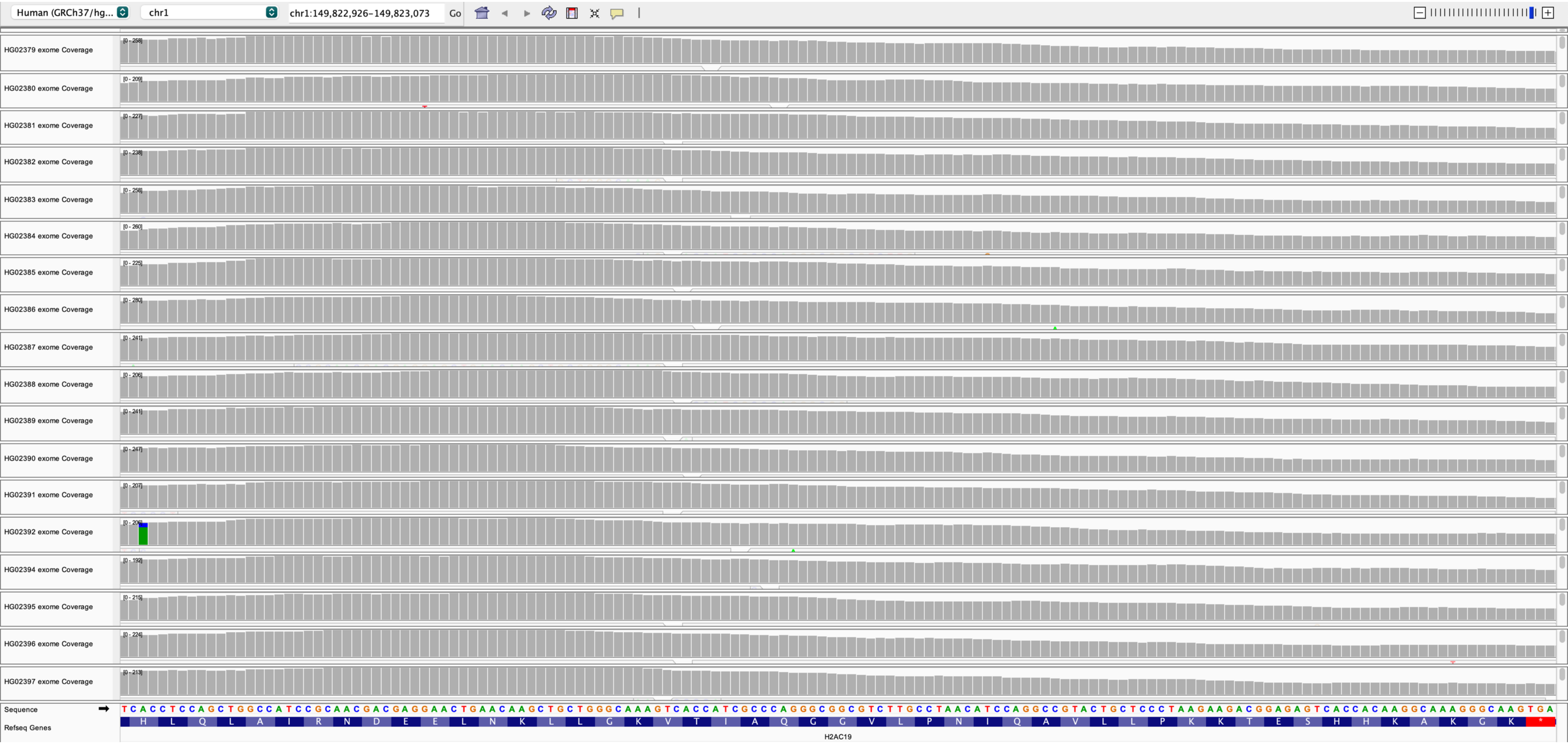

CDX: HG02398-HG02410

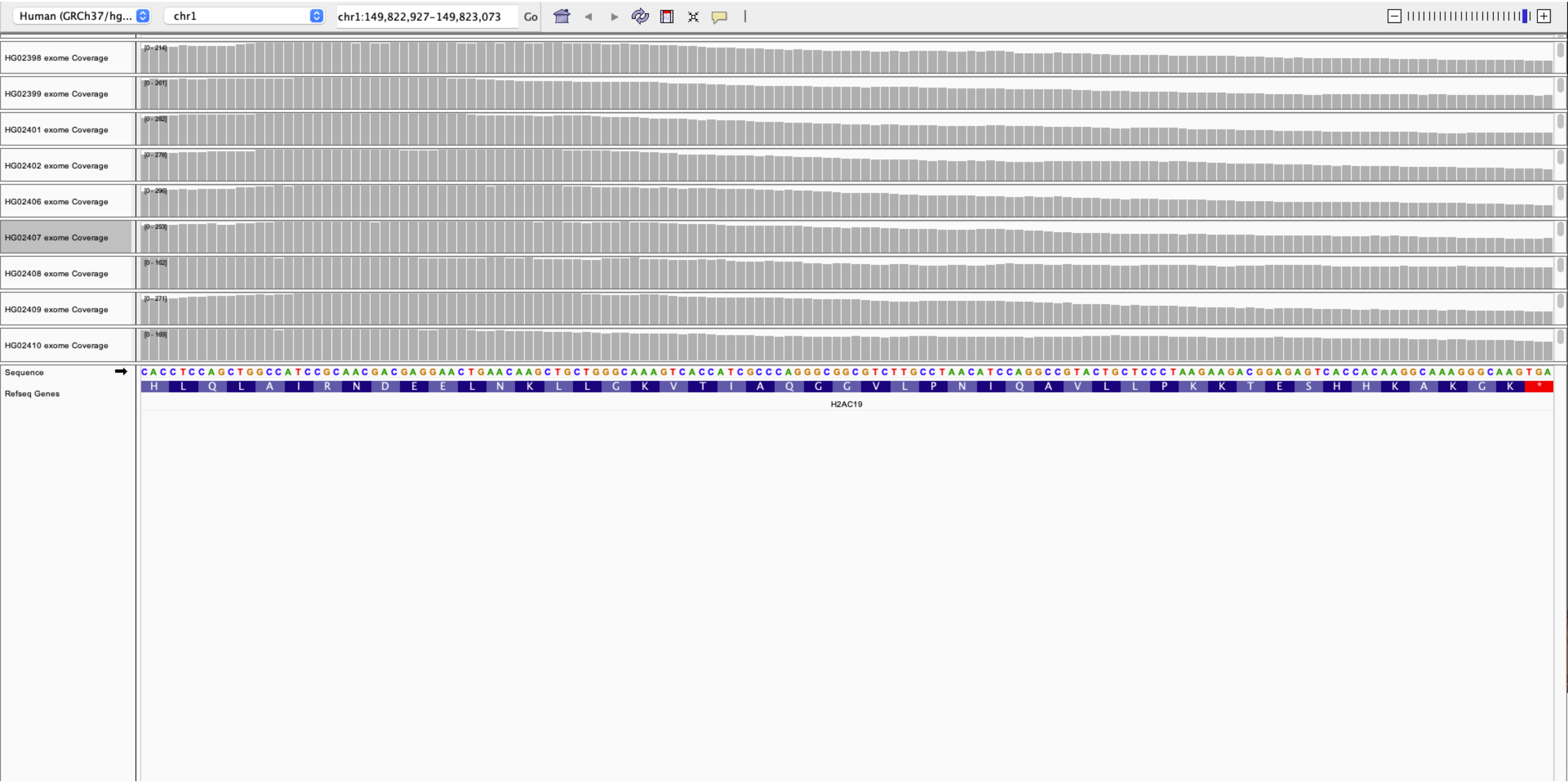

CEU: Utah residents (CEPH) with Northern and Western European ancestry (99 samples)

CEU: NA06984-NA11832

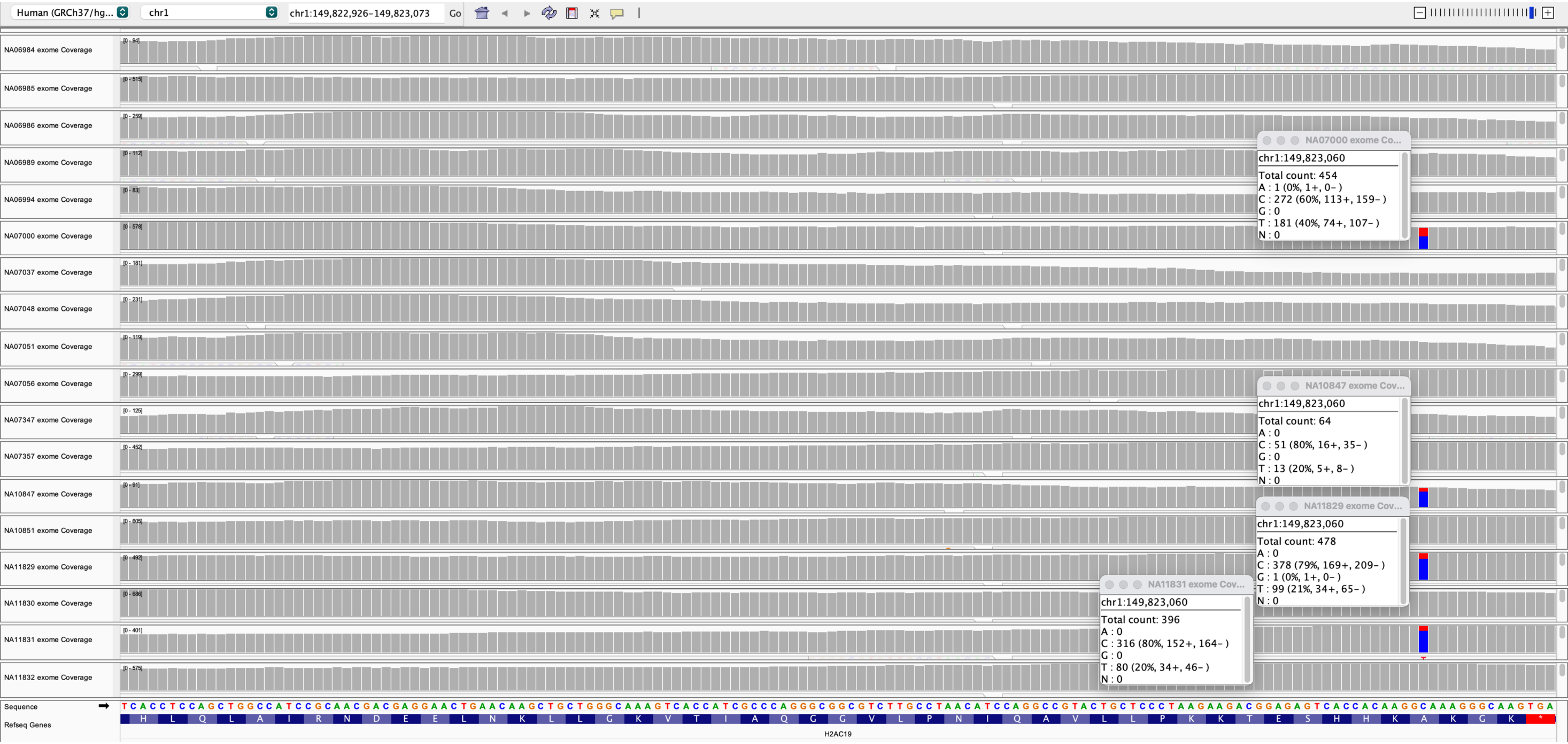

CEU: NA11840-NA12004

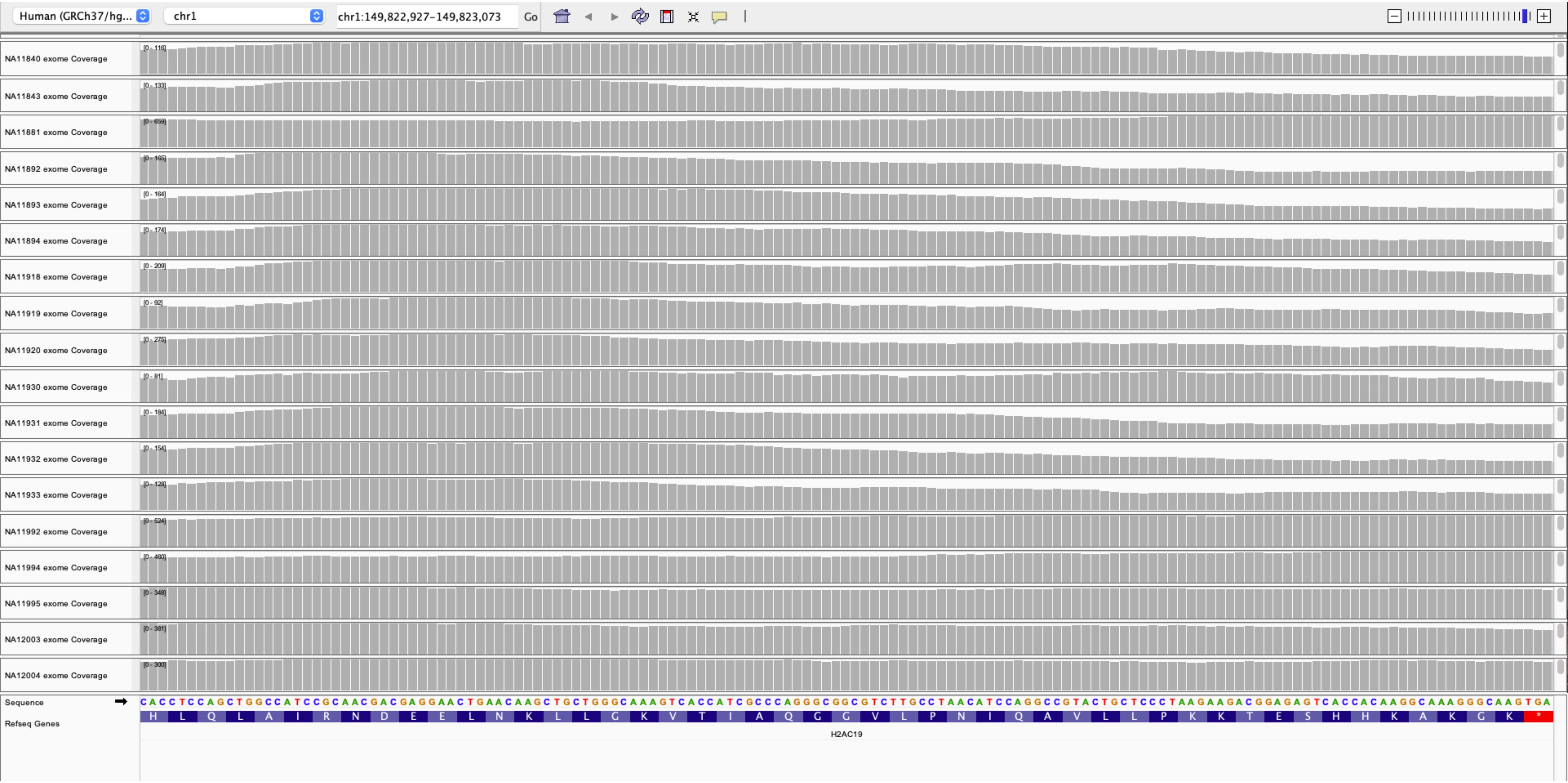

CEU: NA12005-NA12283

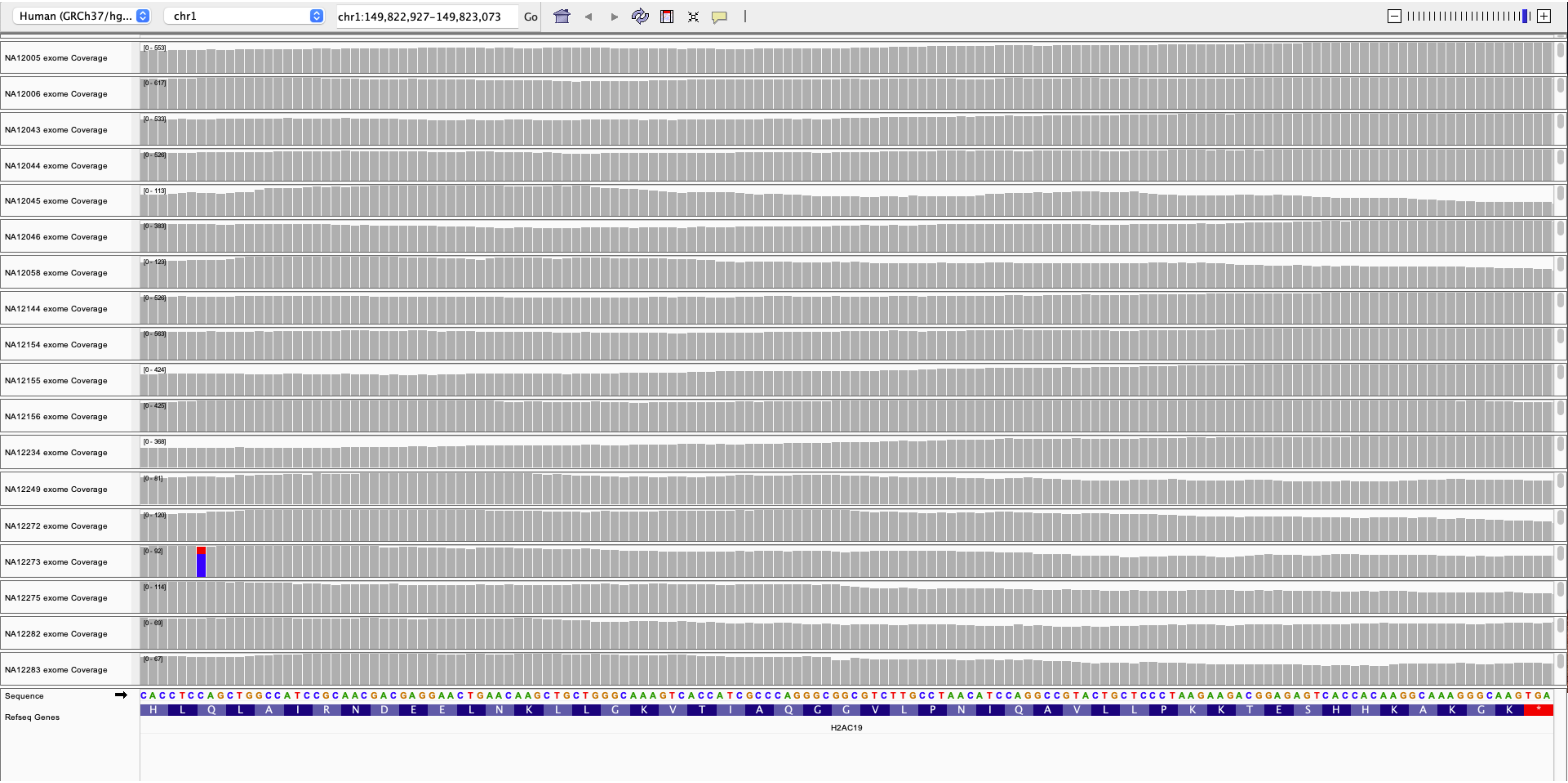

CEU: NA12286-NA12748

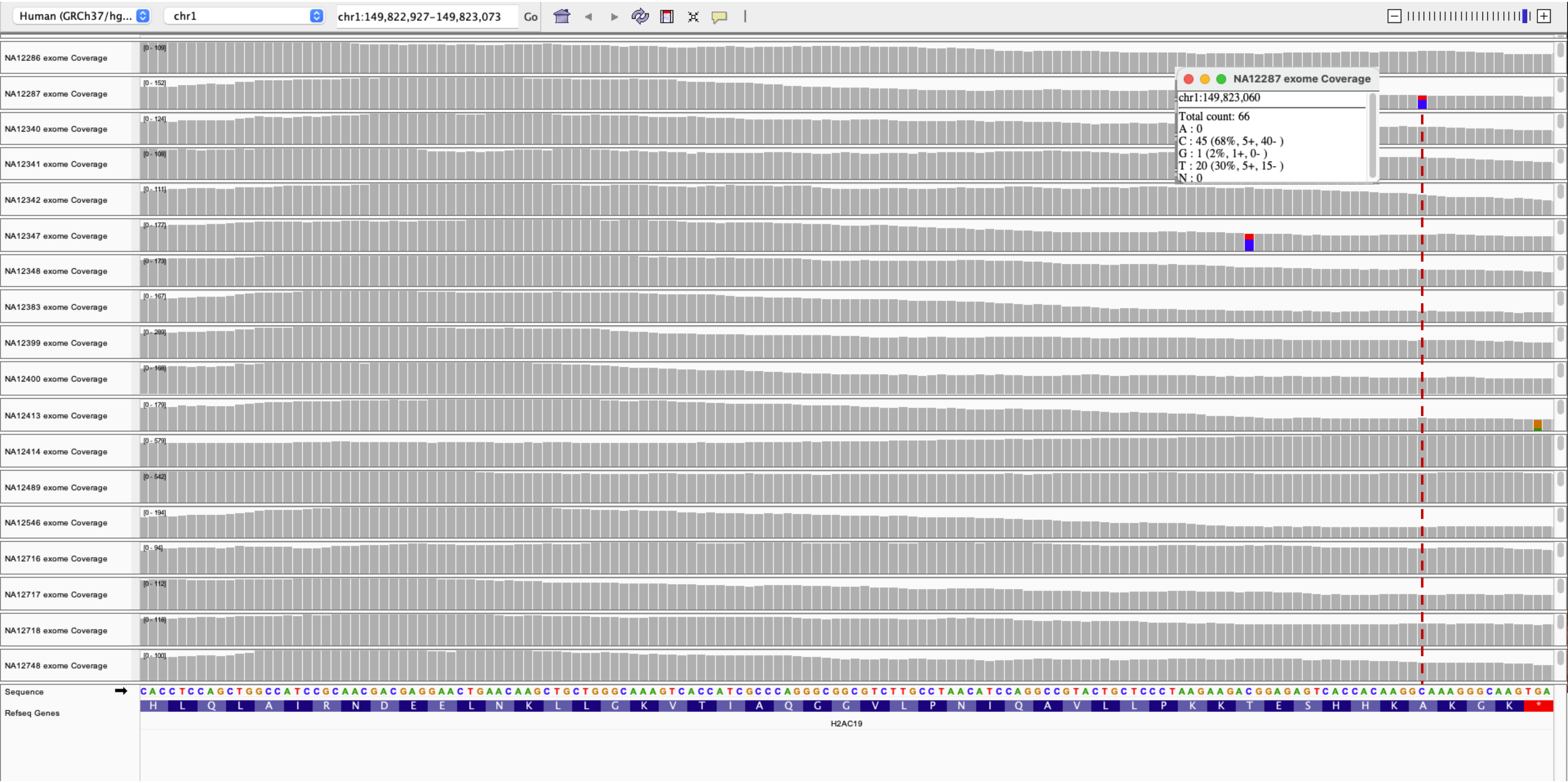

CEU: NA12749-NA12829

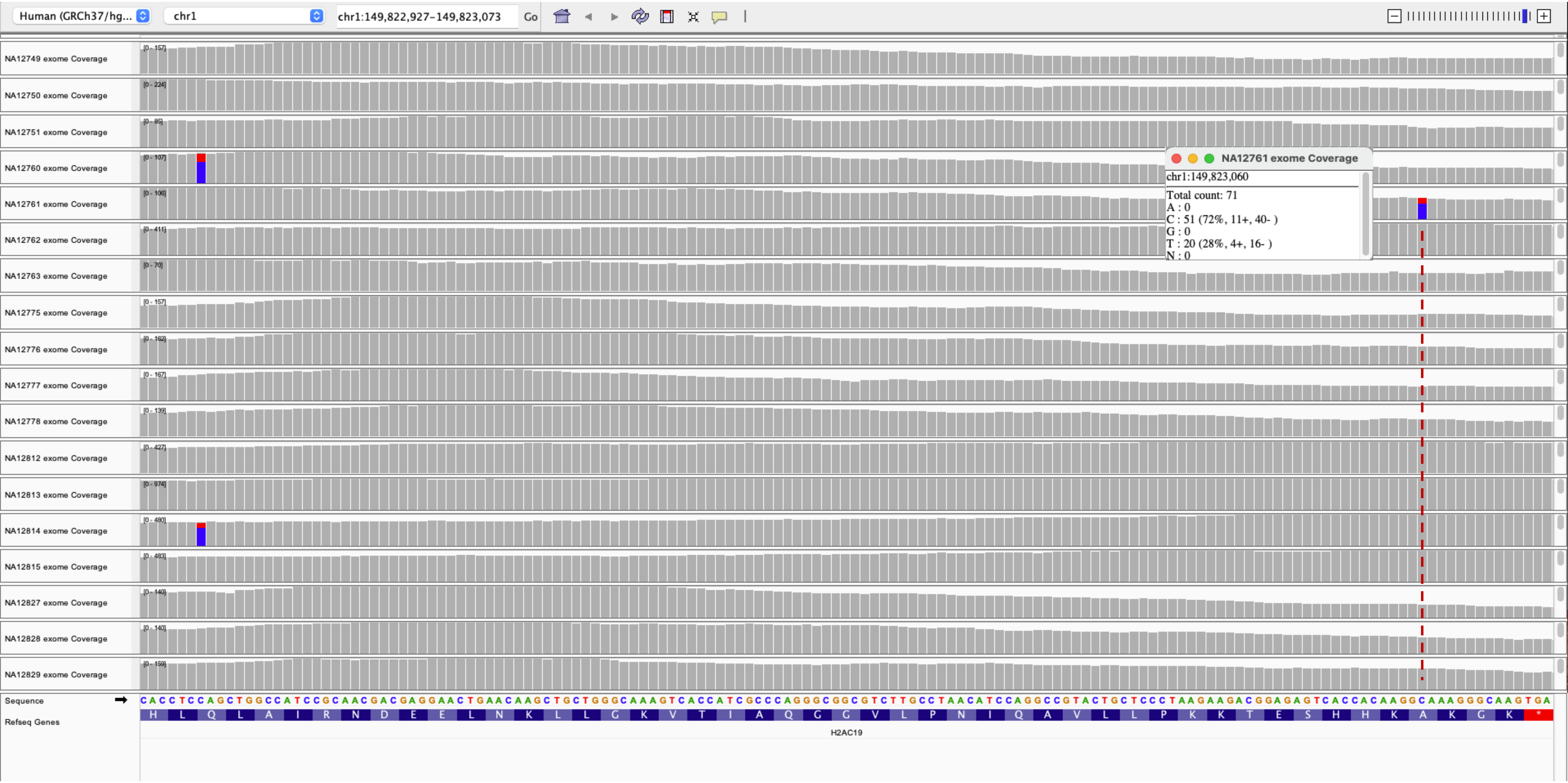

CEU: NA12830-NA12890

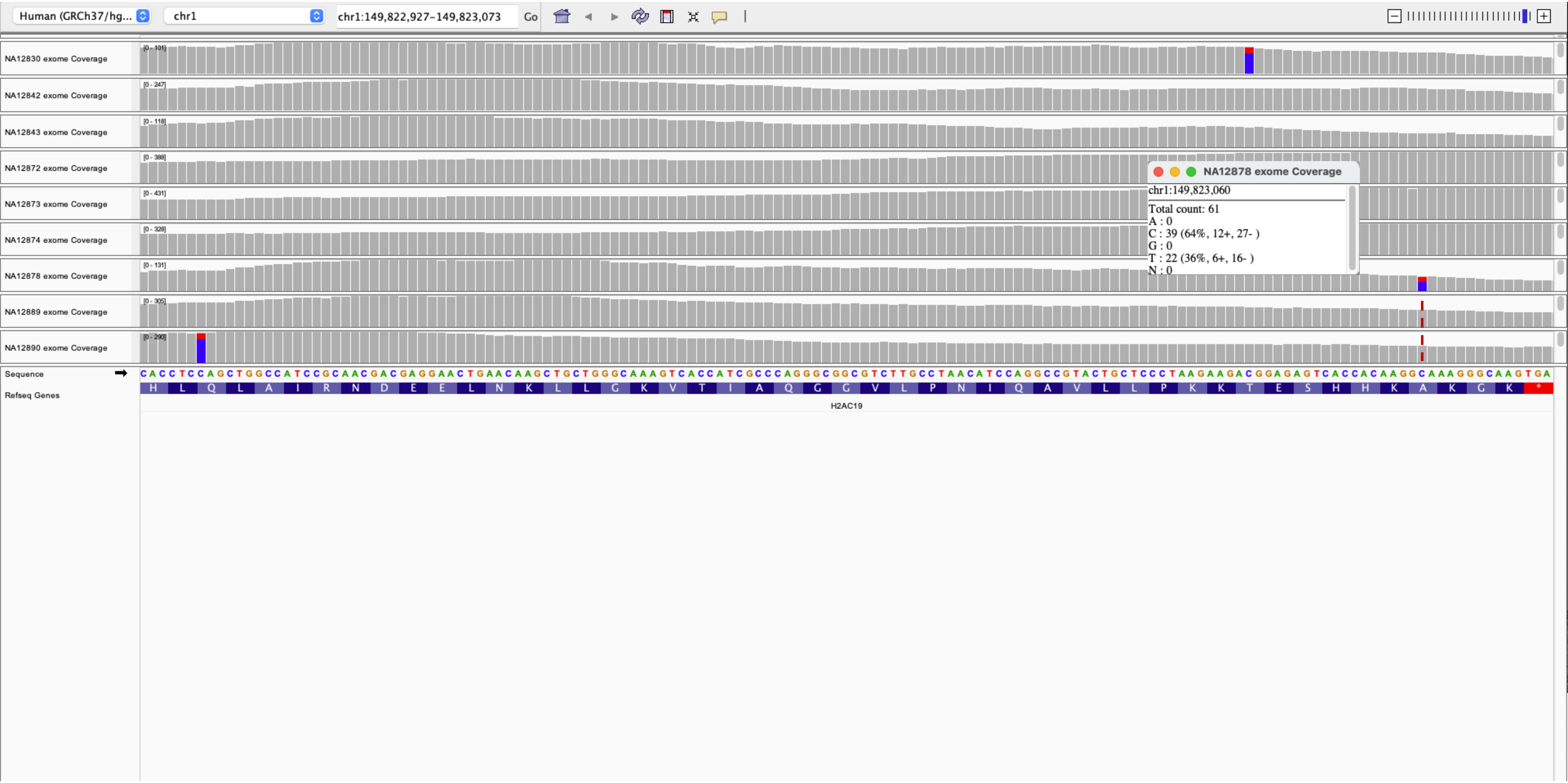

GBR: British in England and Scotland (92 samples)

GBR: HG00096-HG00115

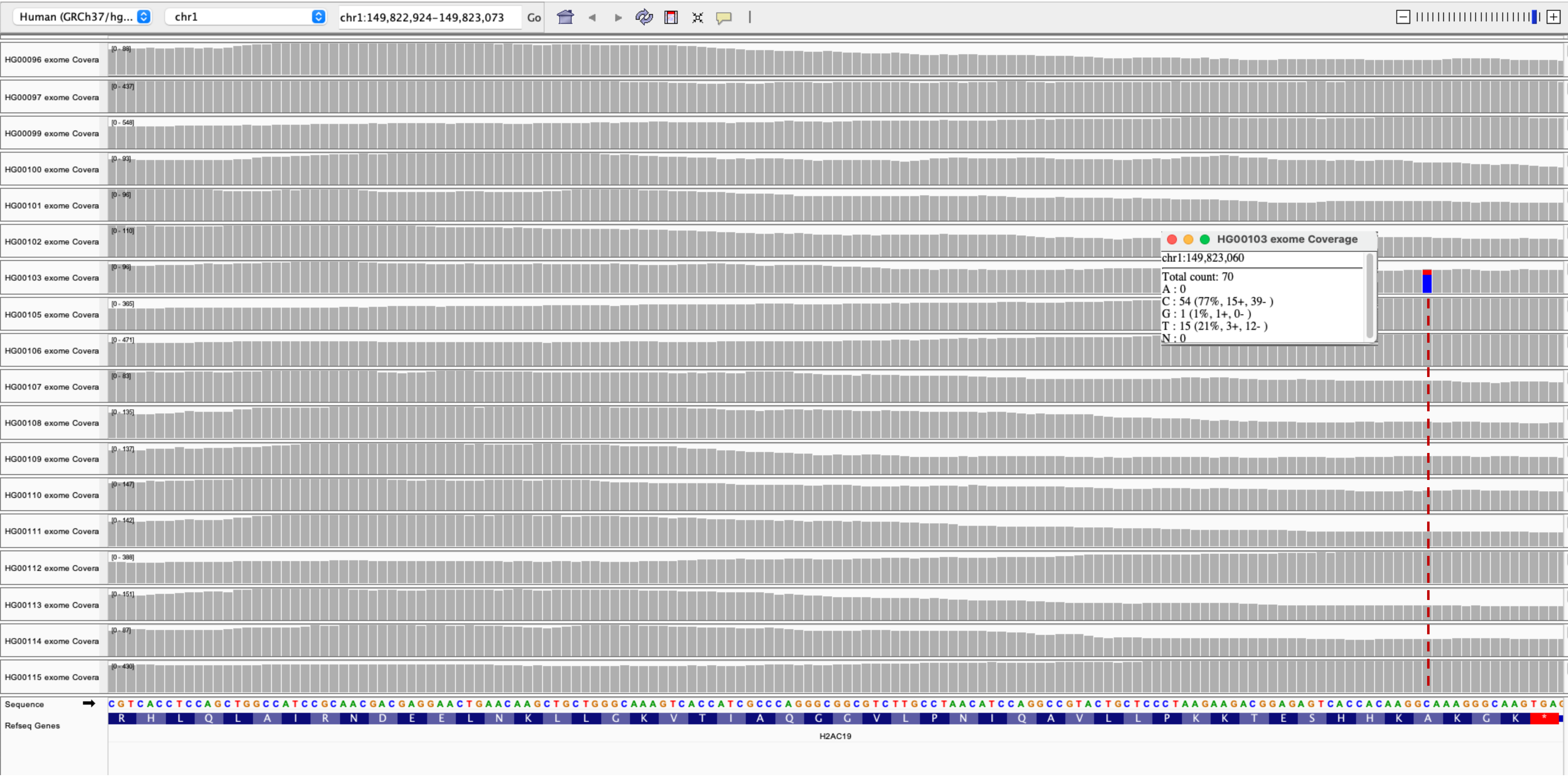

GBR.HG00116-HG00133

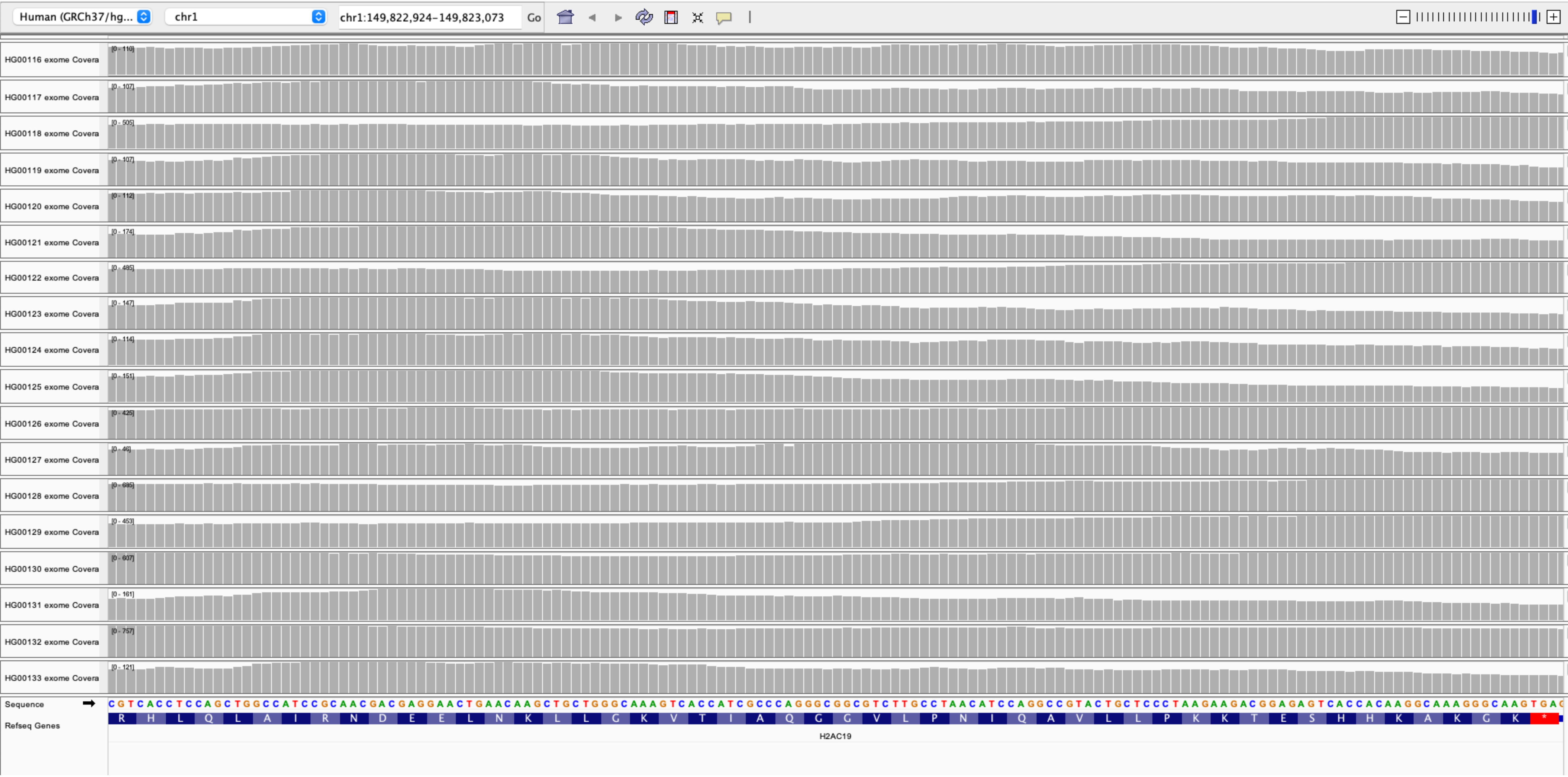

GBR: HG00136-HG00158

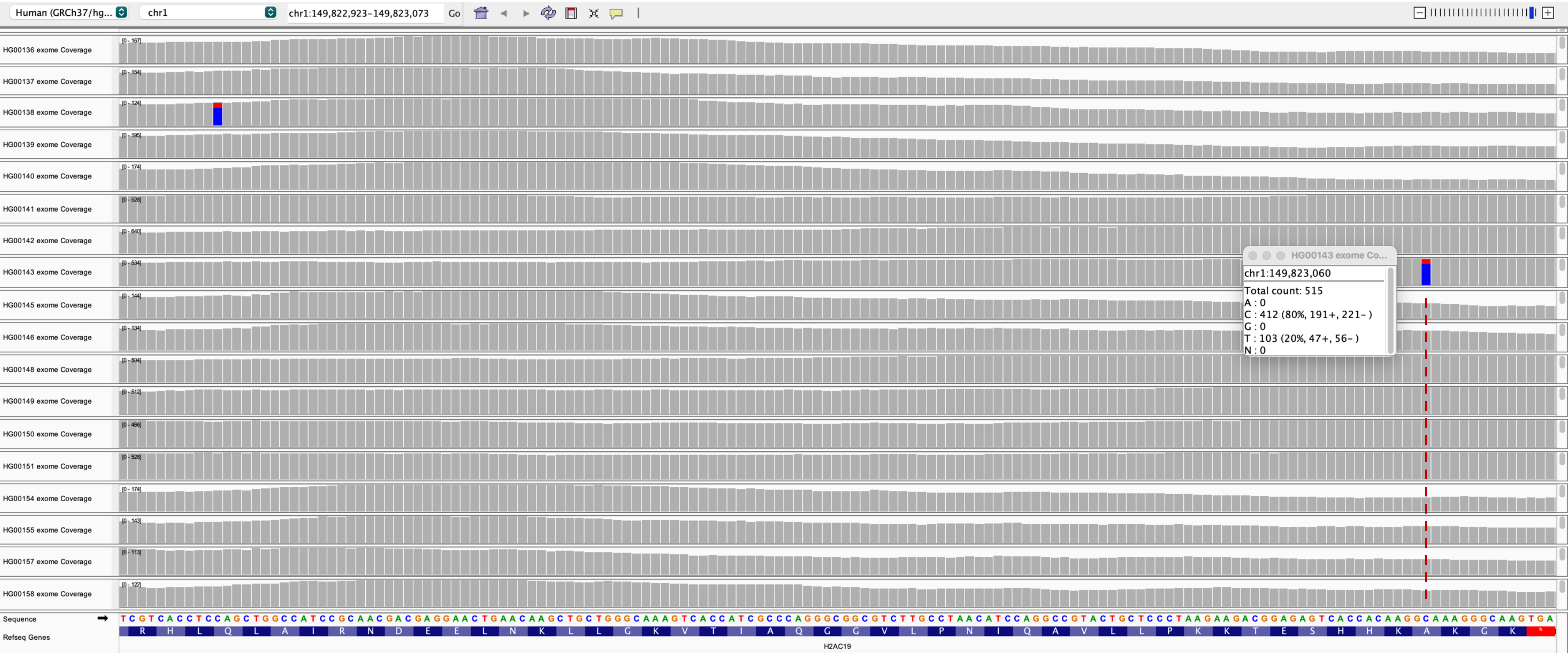

GBR: HG00159-HG00250

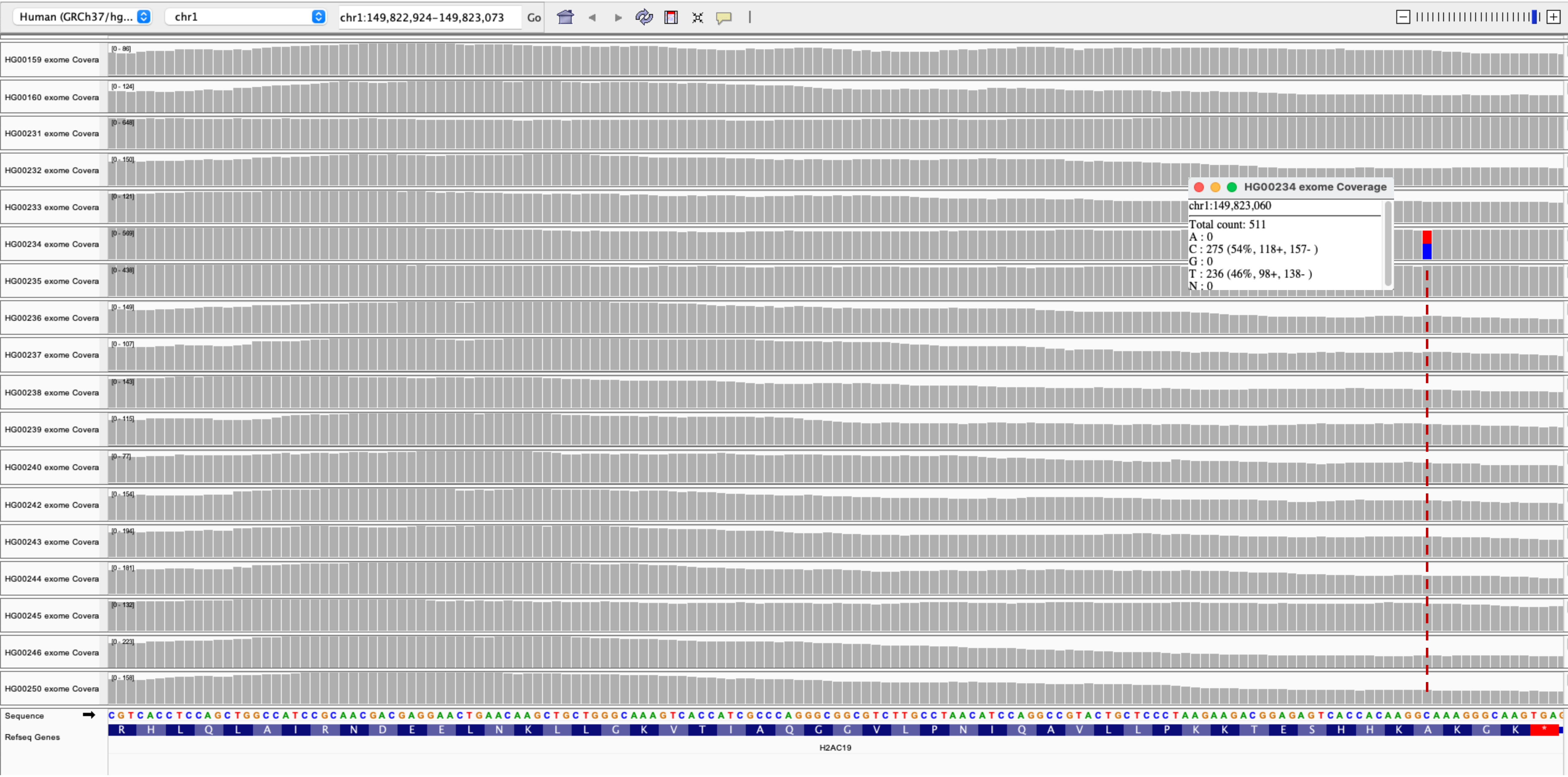

GBR: HG00251-HG02215

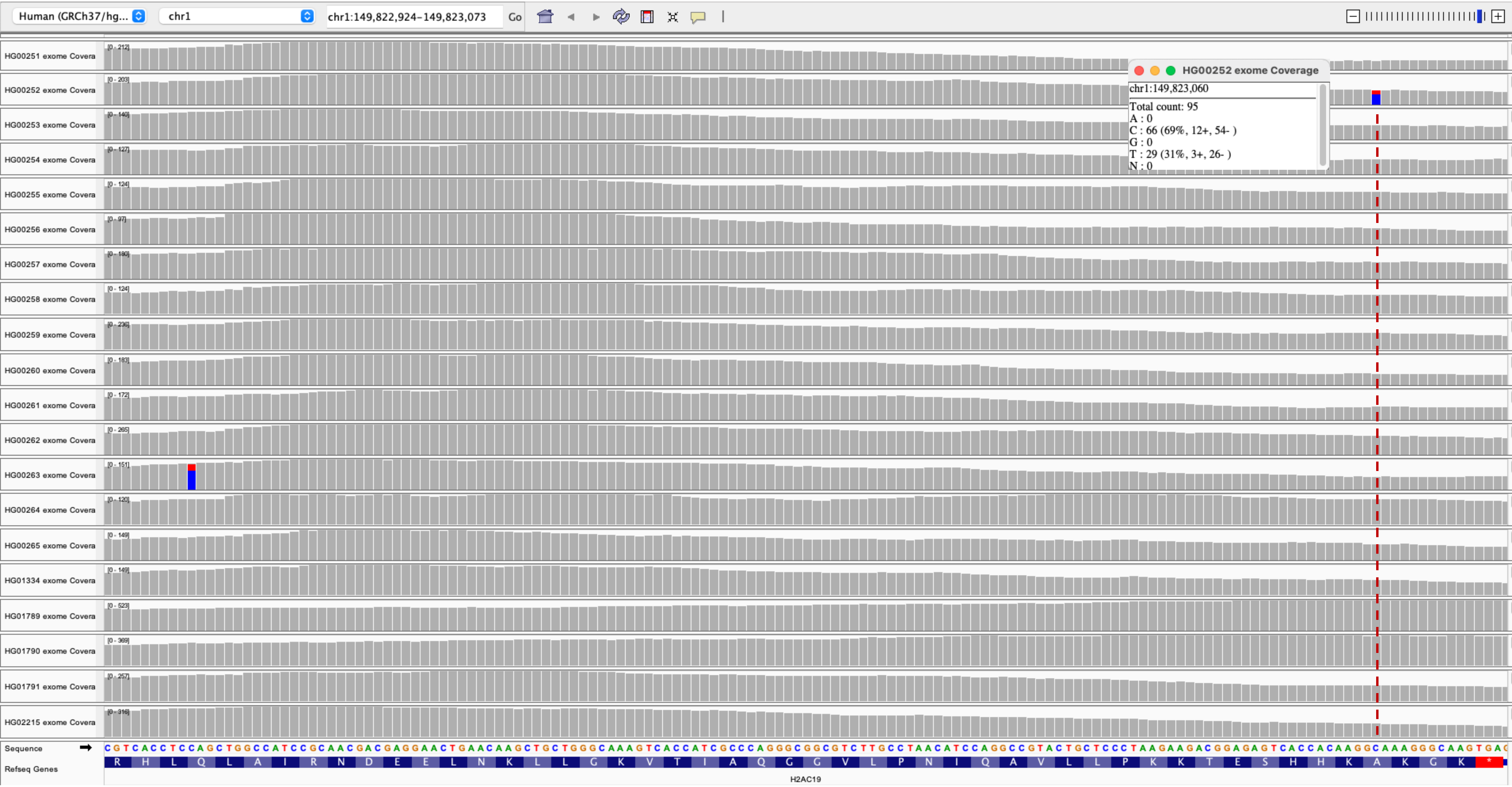

FIN: Finnish in Finland
(99 samples)

FIN: HG00171-HG00268

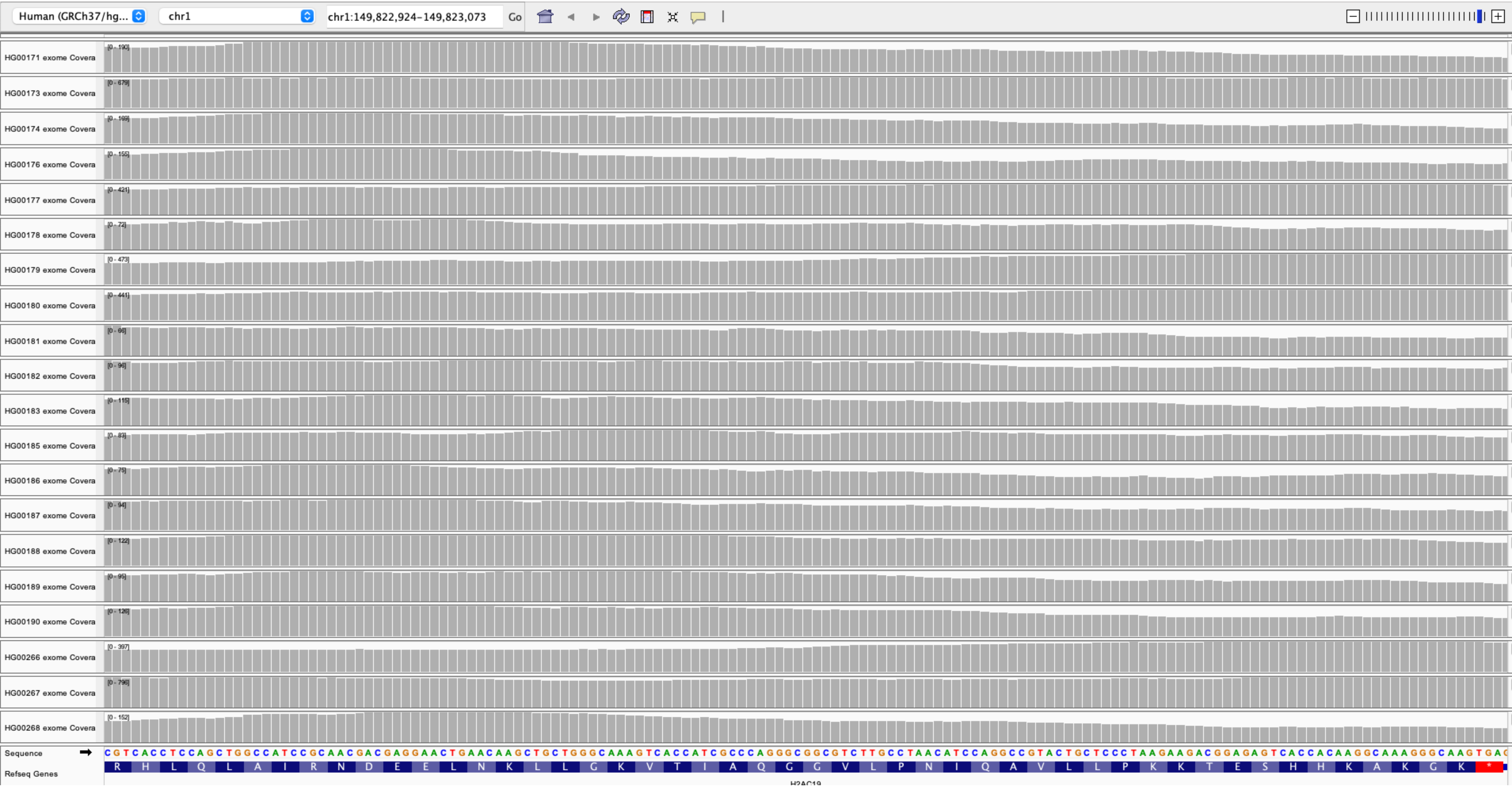

FIN: HG00269-HG00309

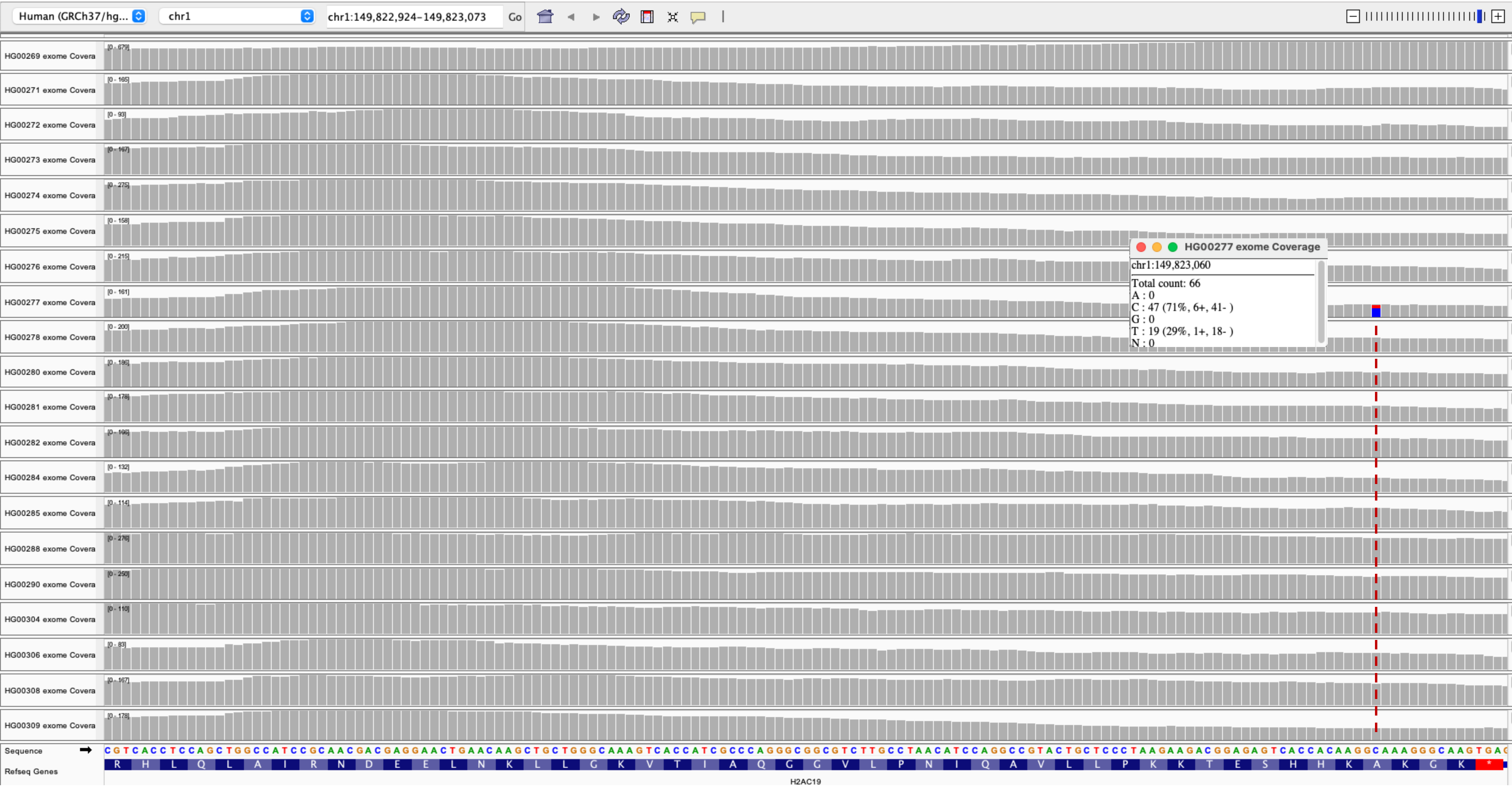

FIN: HG0310-HG00335

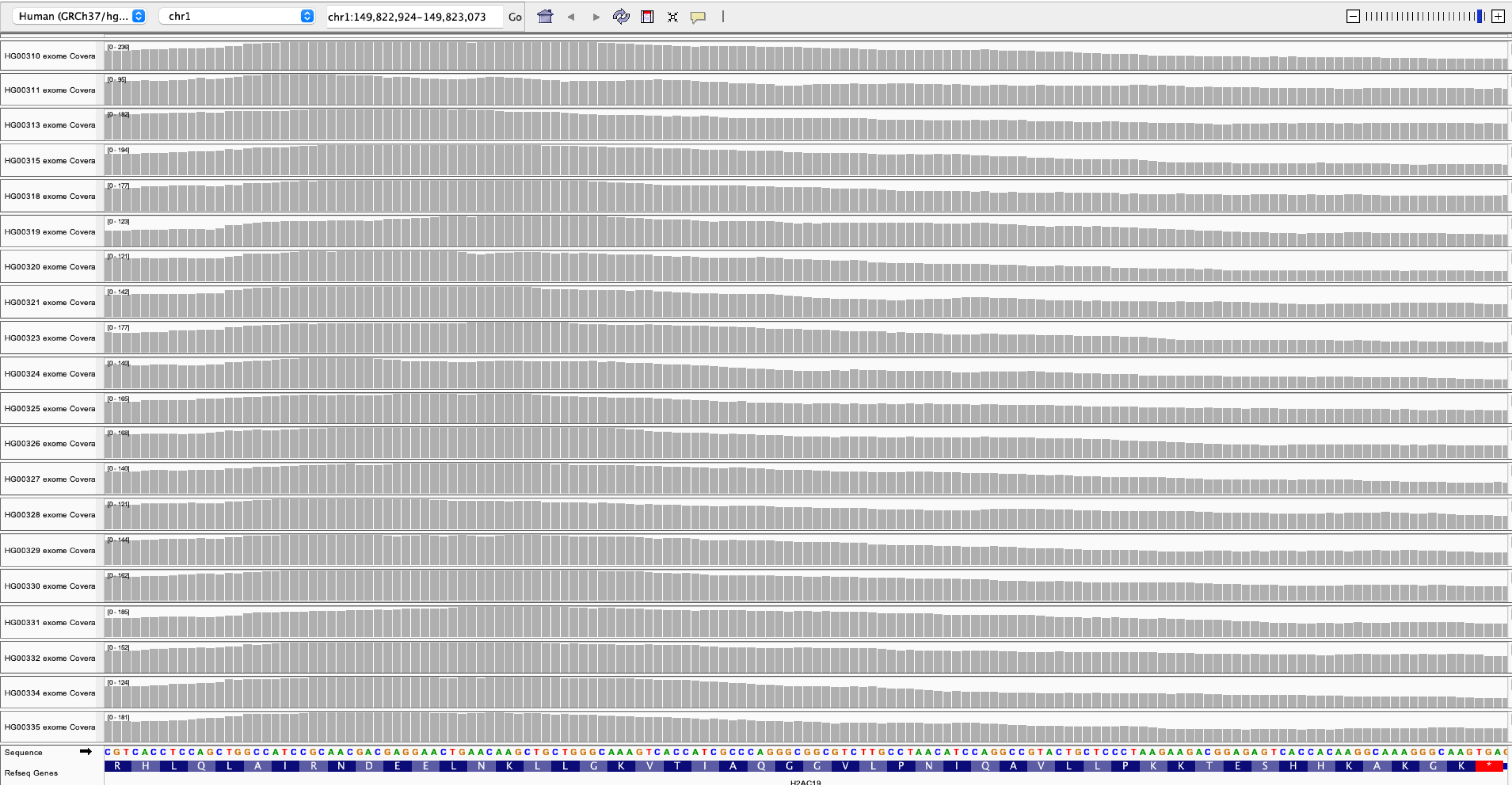

FIN: HG00336-HG00361

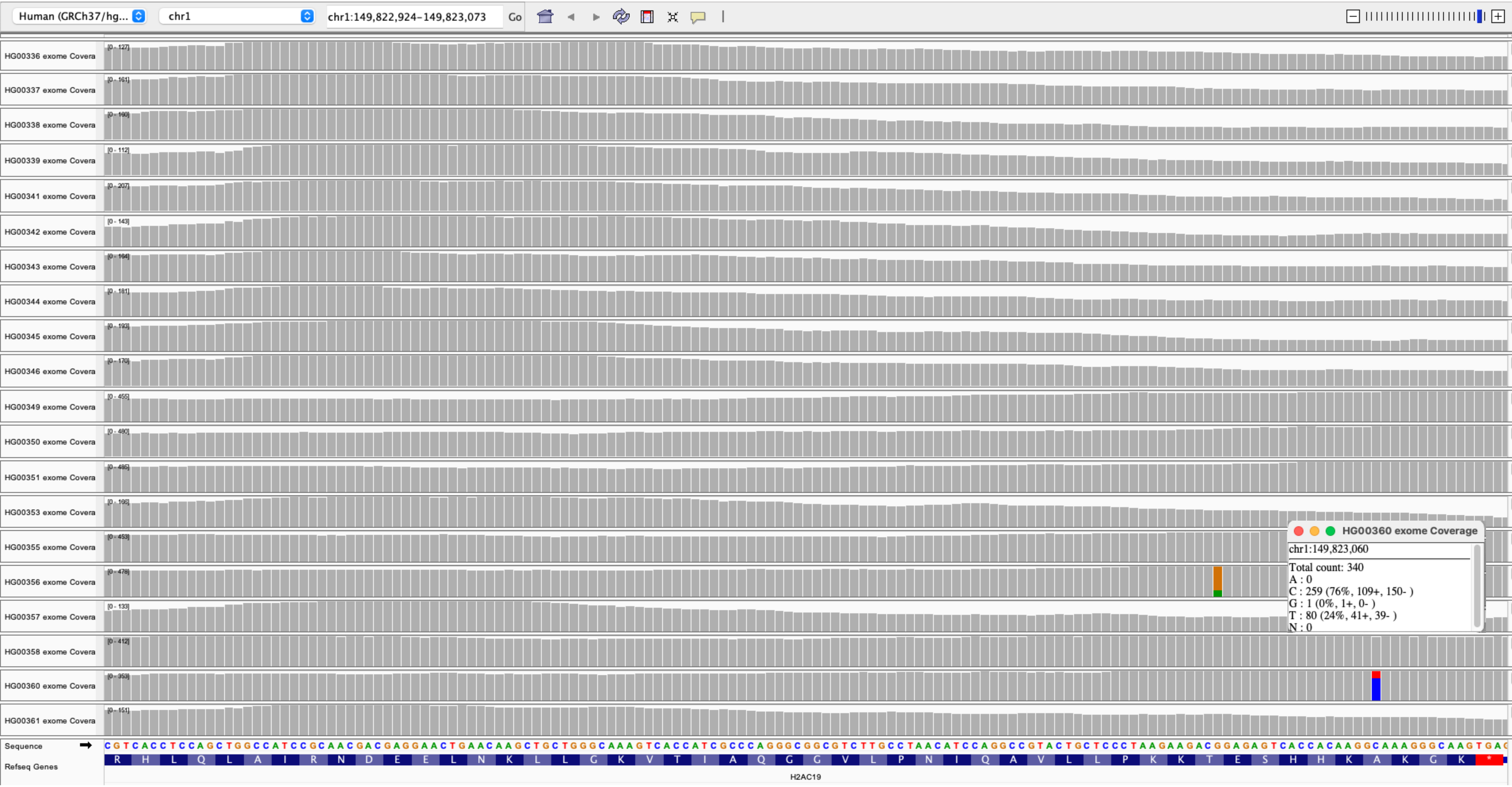

FIN: HG00362-HG00384

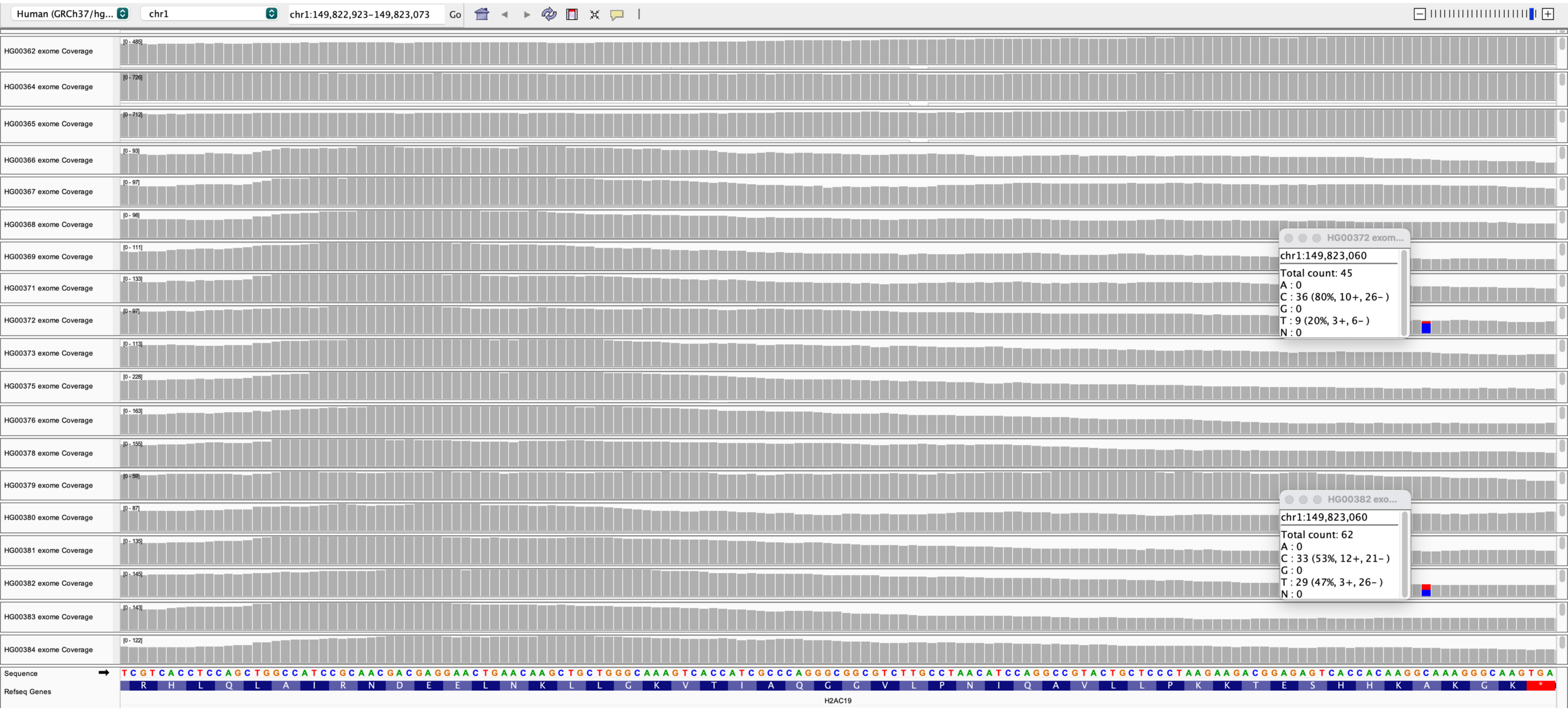

IBS: Iberian Populations in Spain (107 samples)

IBS: HG01500-HG01528

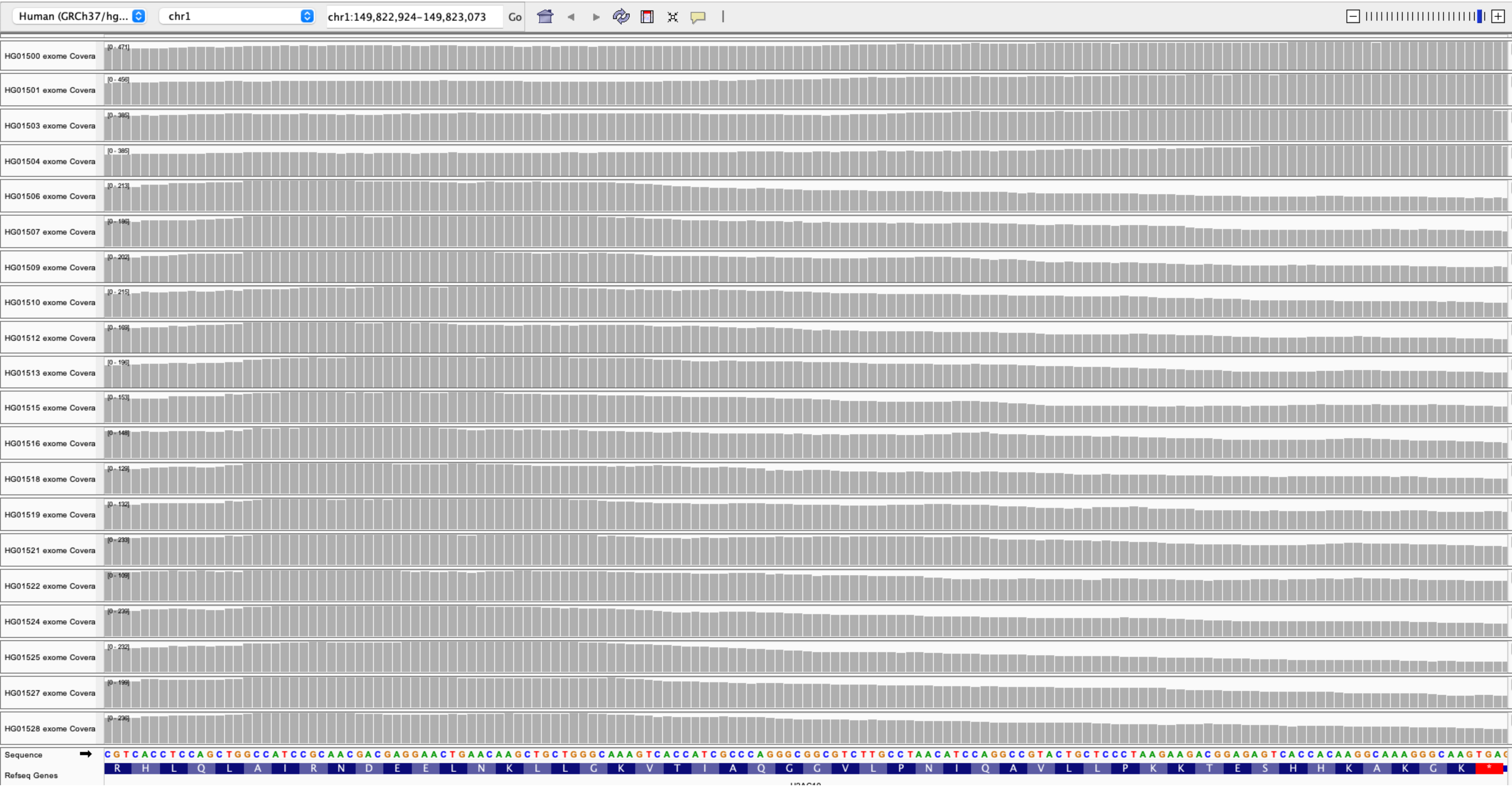

IBS: HG01530-HG01624

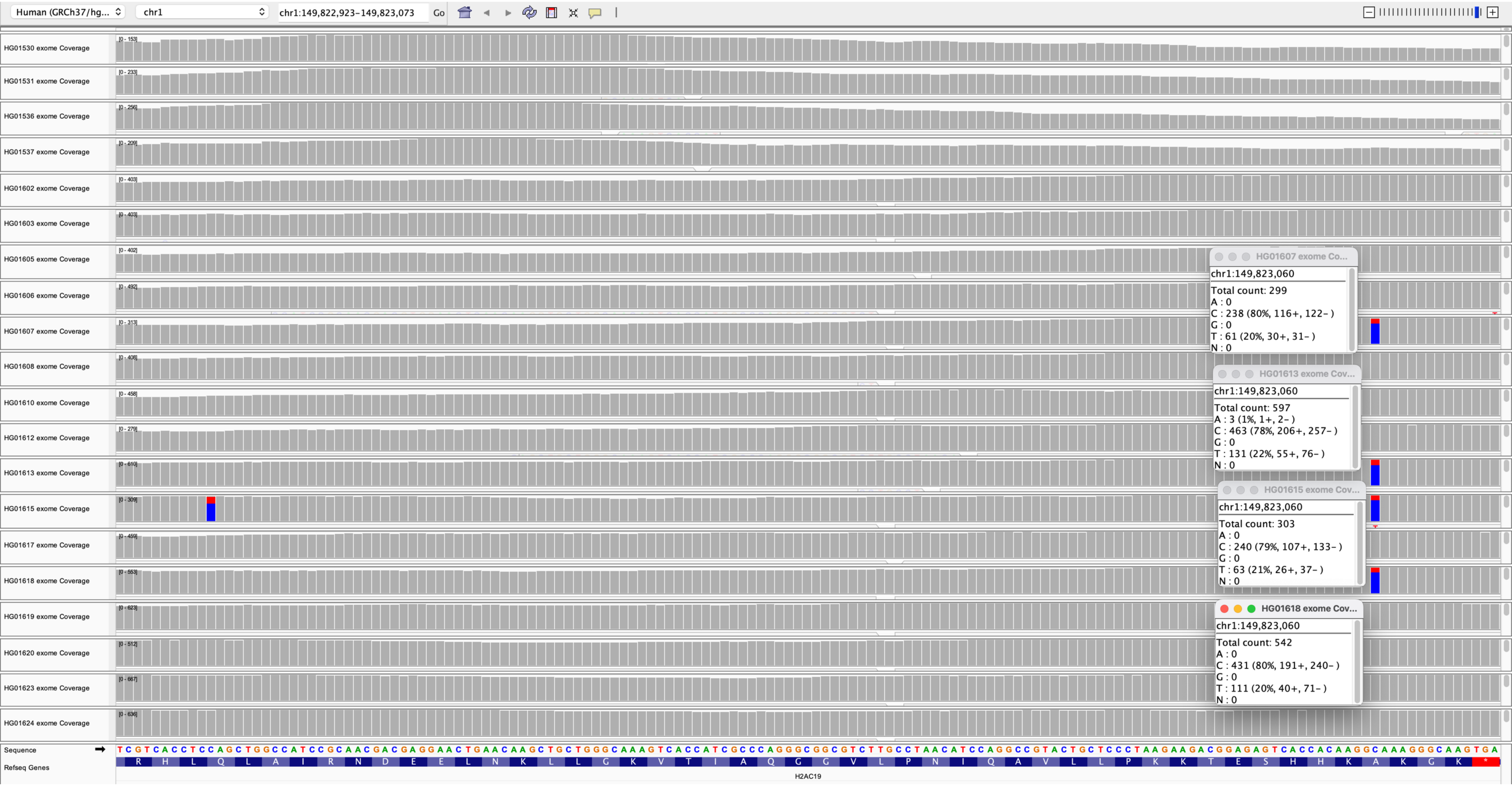

IBS: HG01625-HG01686

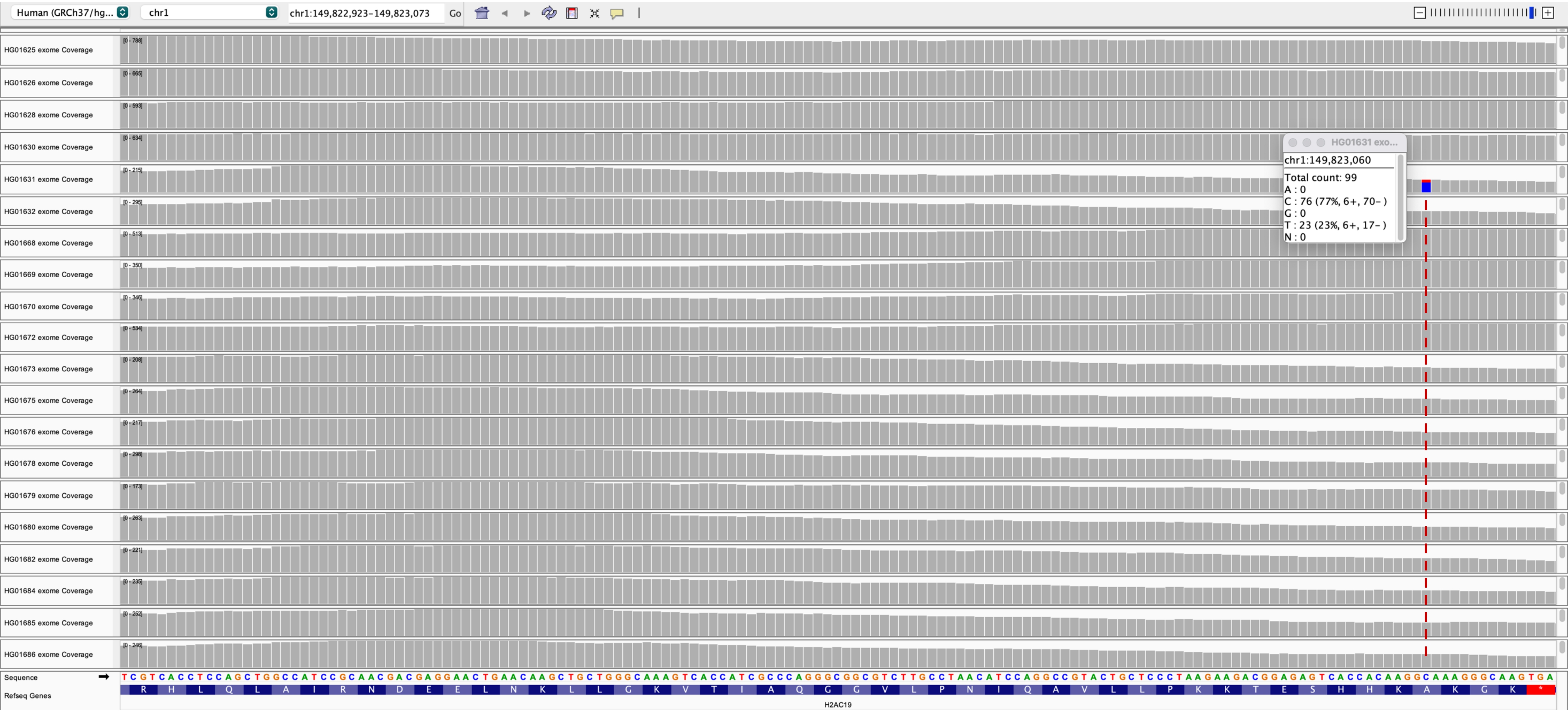

IBS: HG01694-HG01766

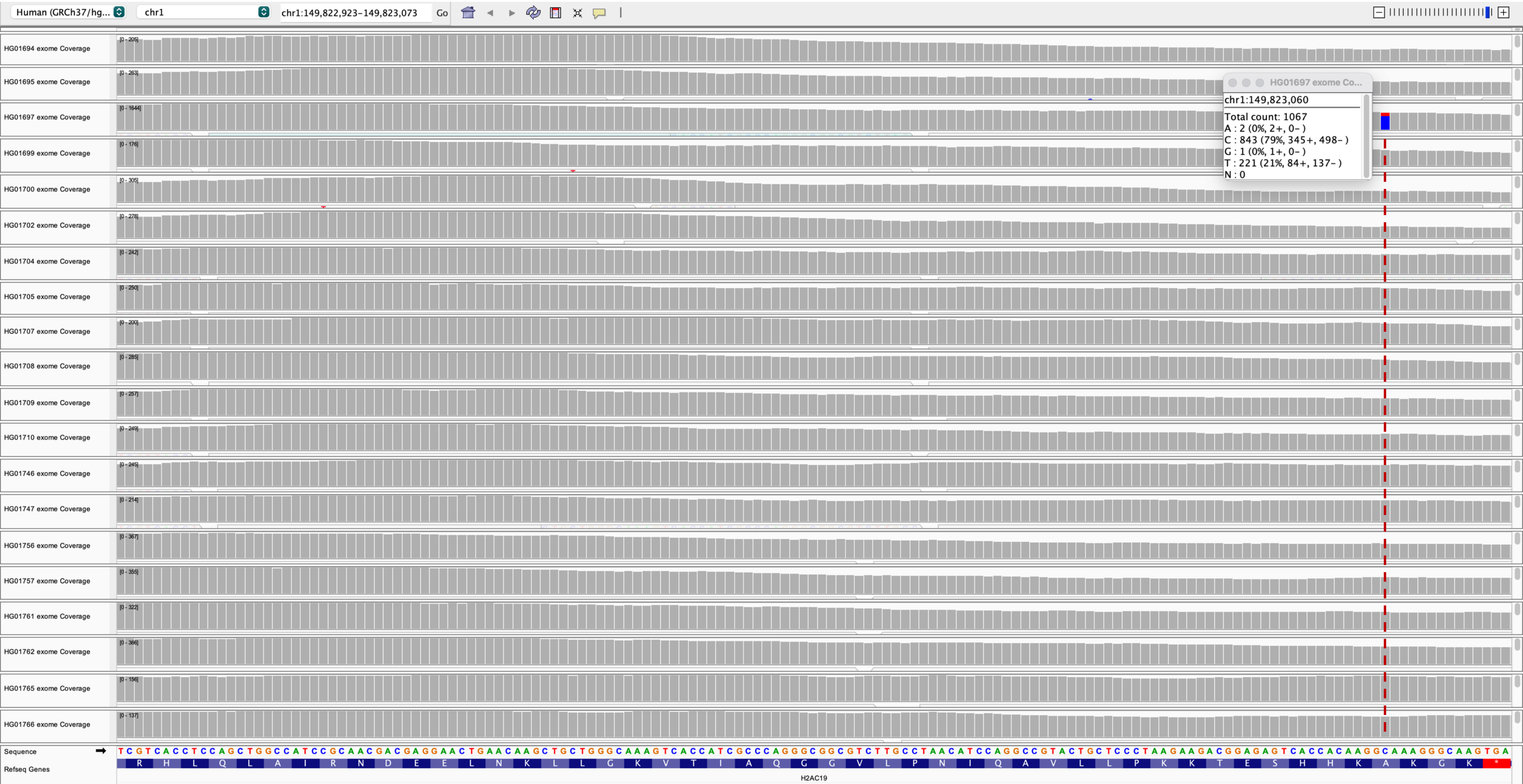

IBS: HG01767-HG02230

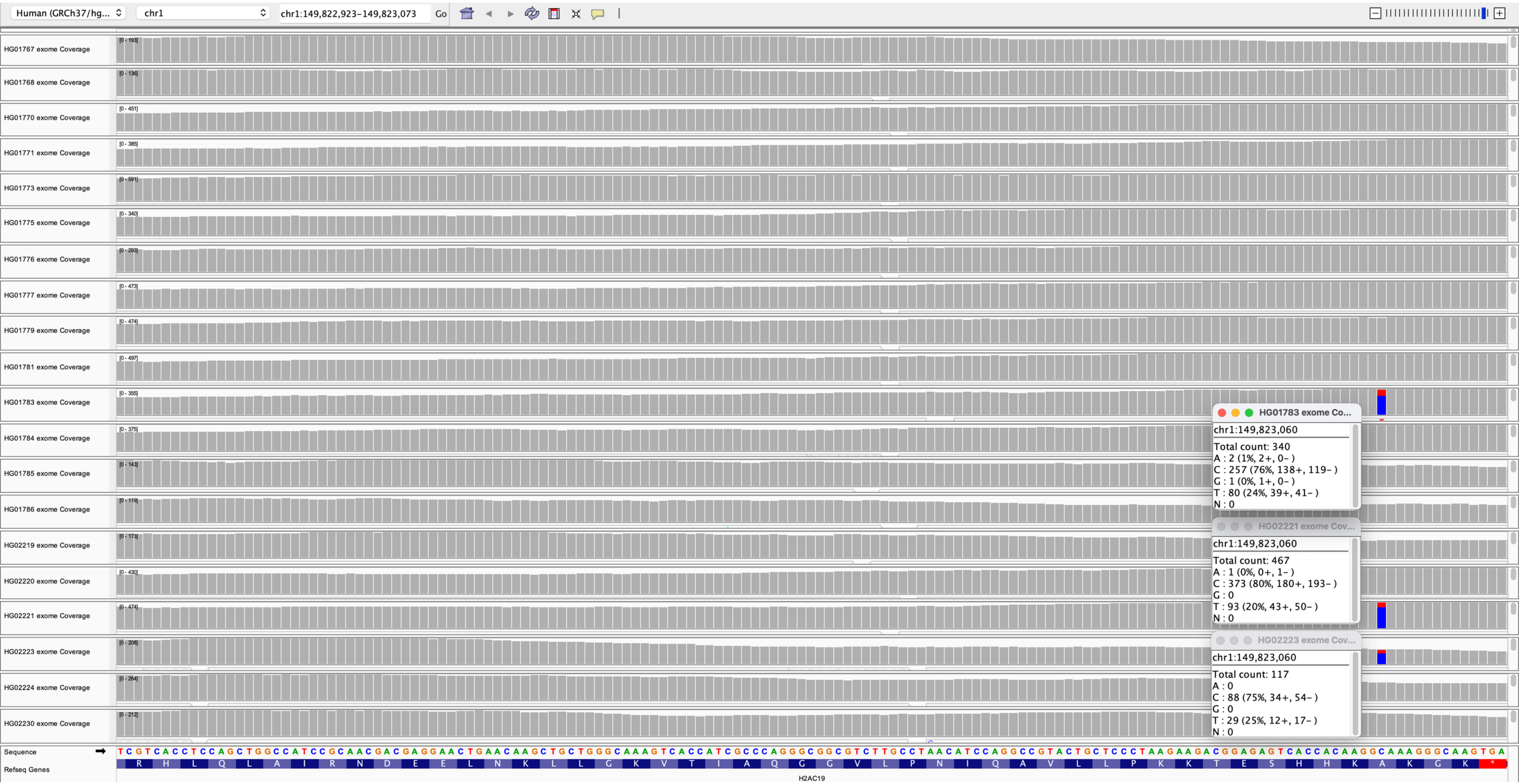

IBS: HG02231-HG02239

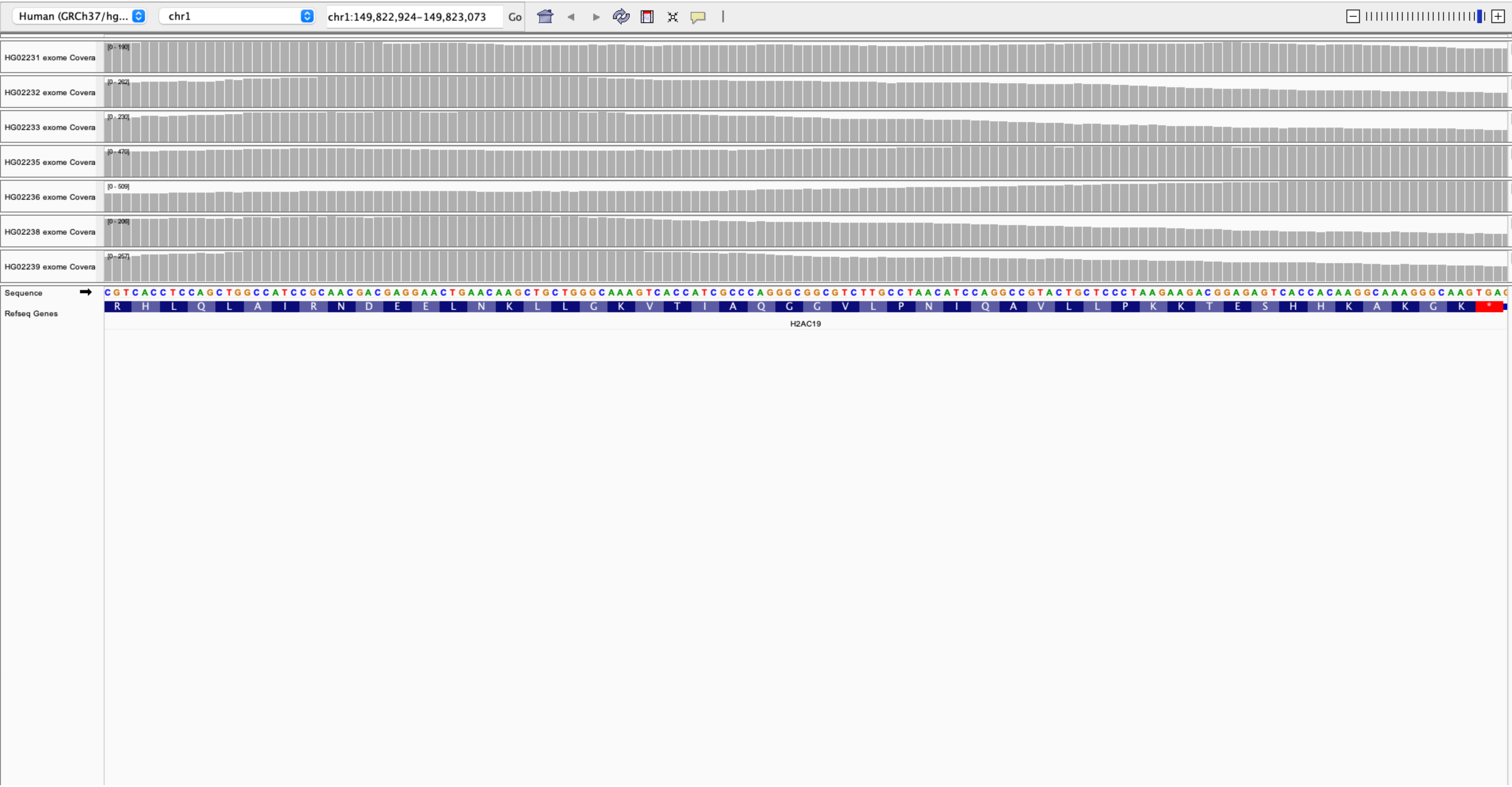

TSI: Toscani in Italia
(108 samples)

TSI: NA20502-NA20521

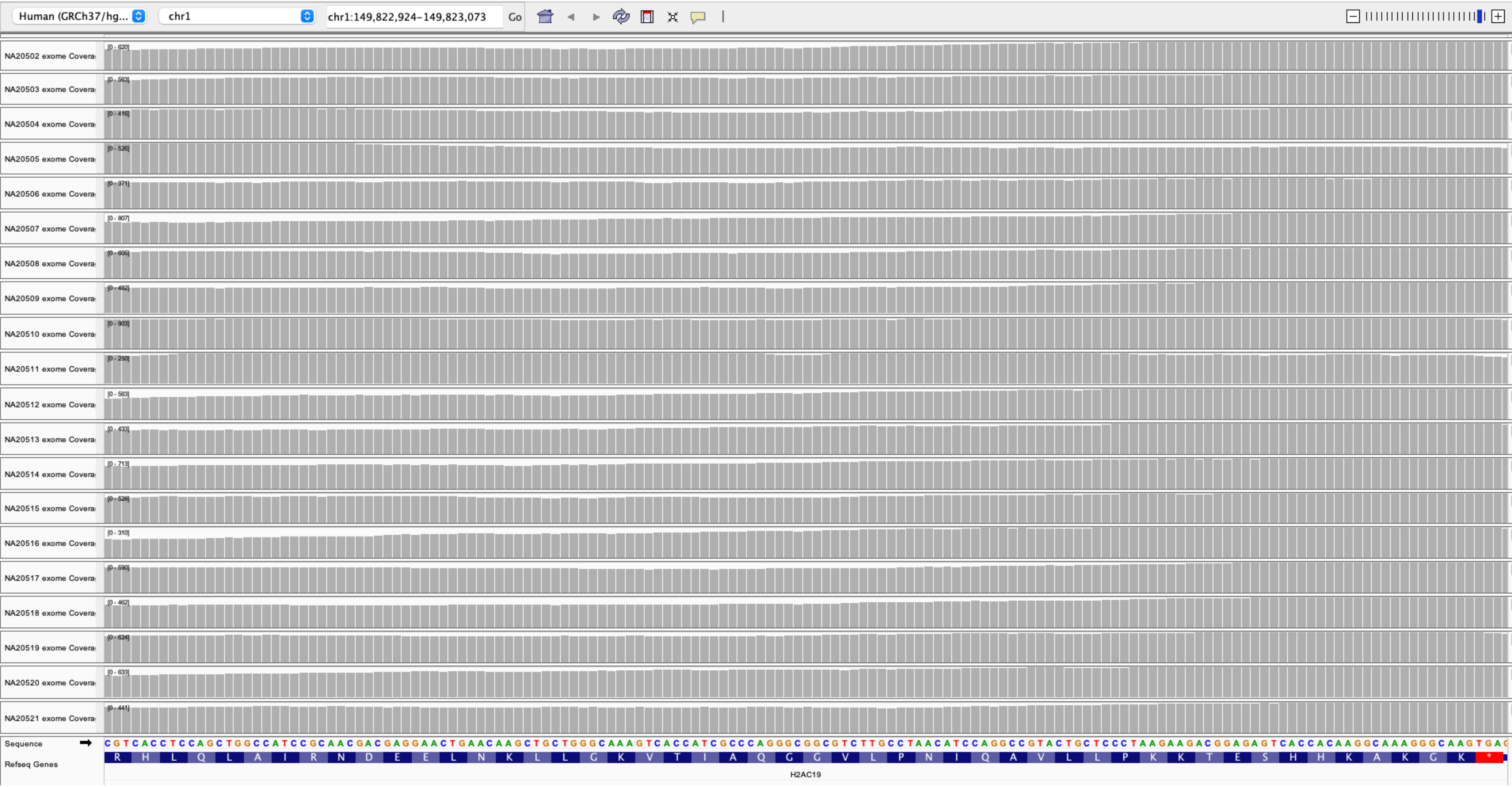

TSI: NA20522-NA20543

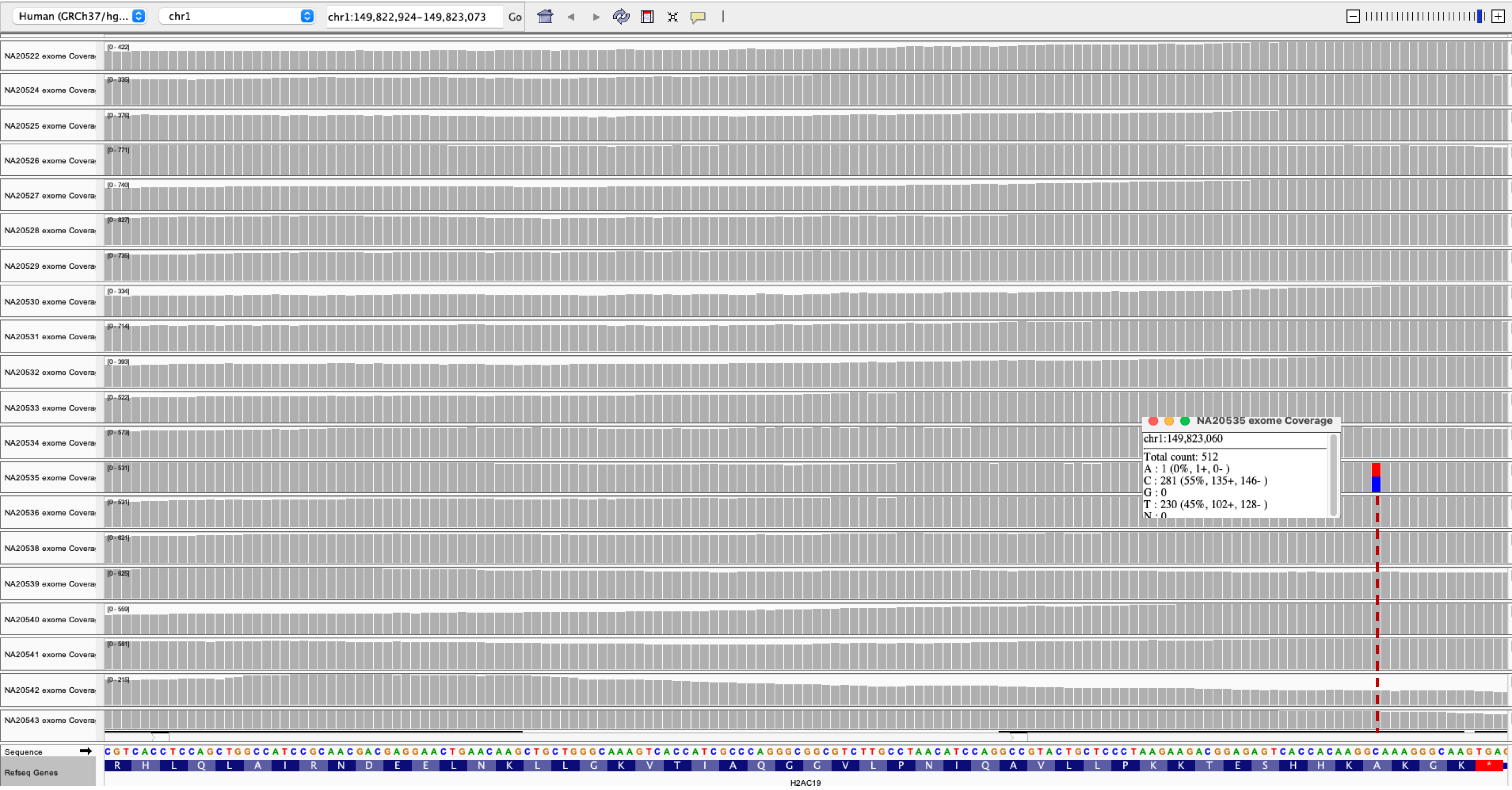

TSI: NA20544-NA20763

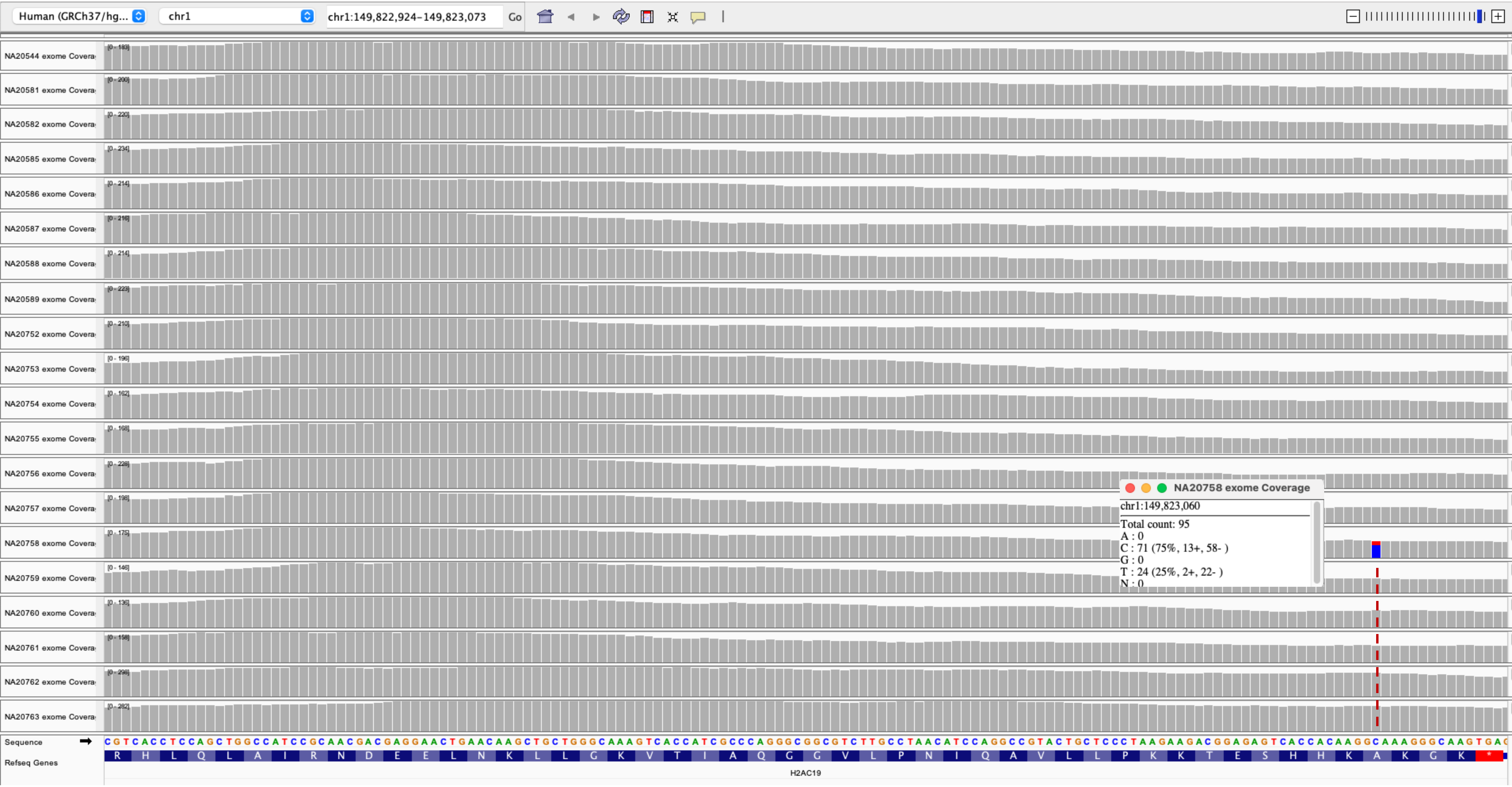

TSI: NA20764-NA20795

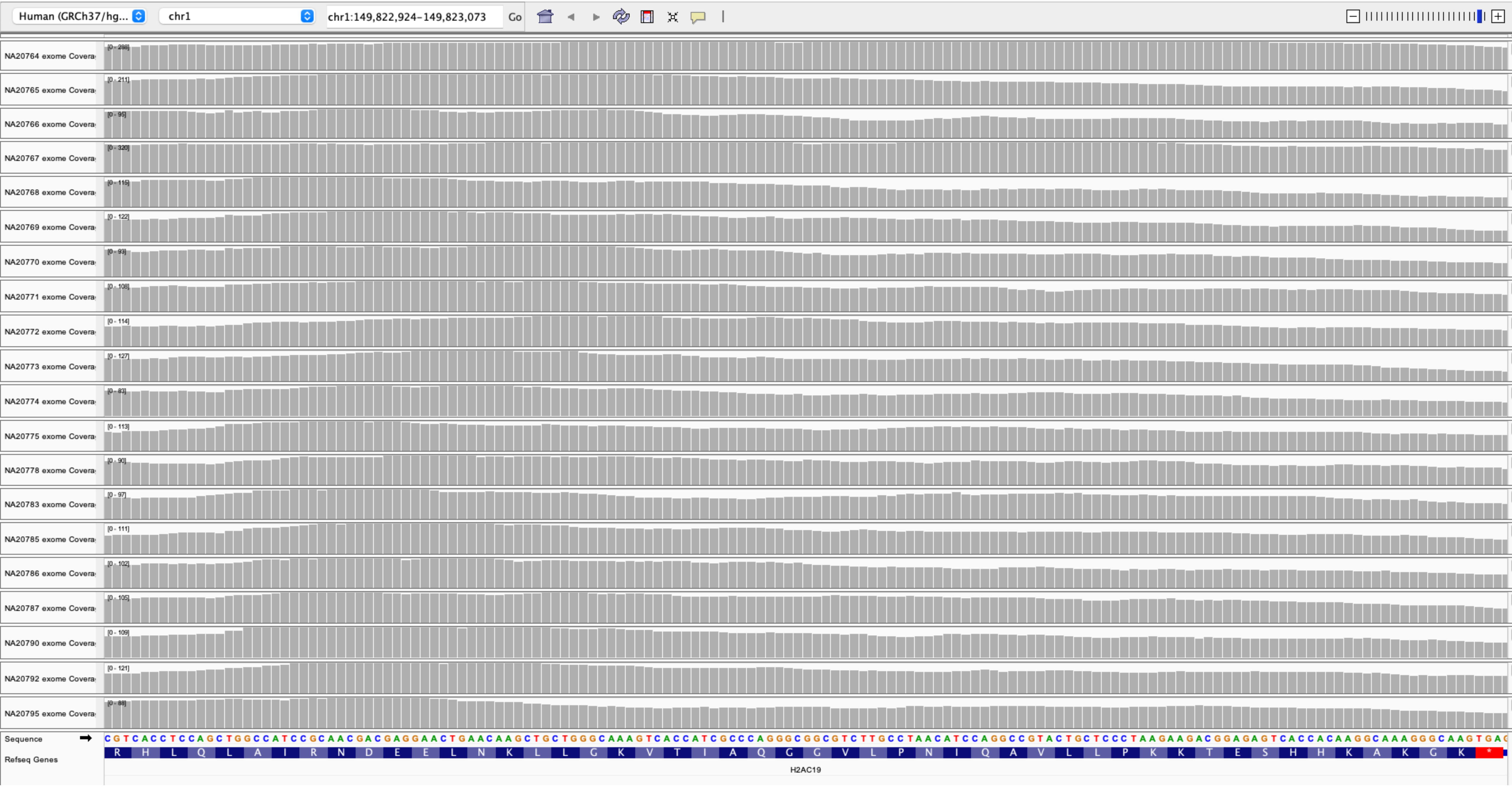

TSI: NA20796-NA20815

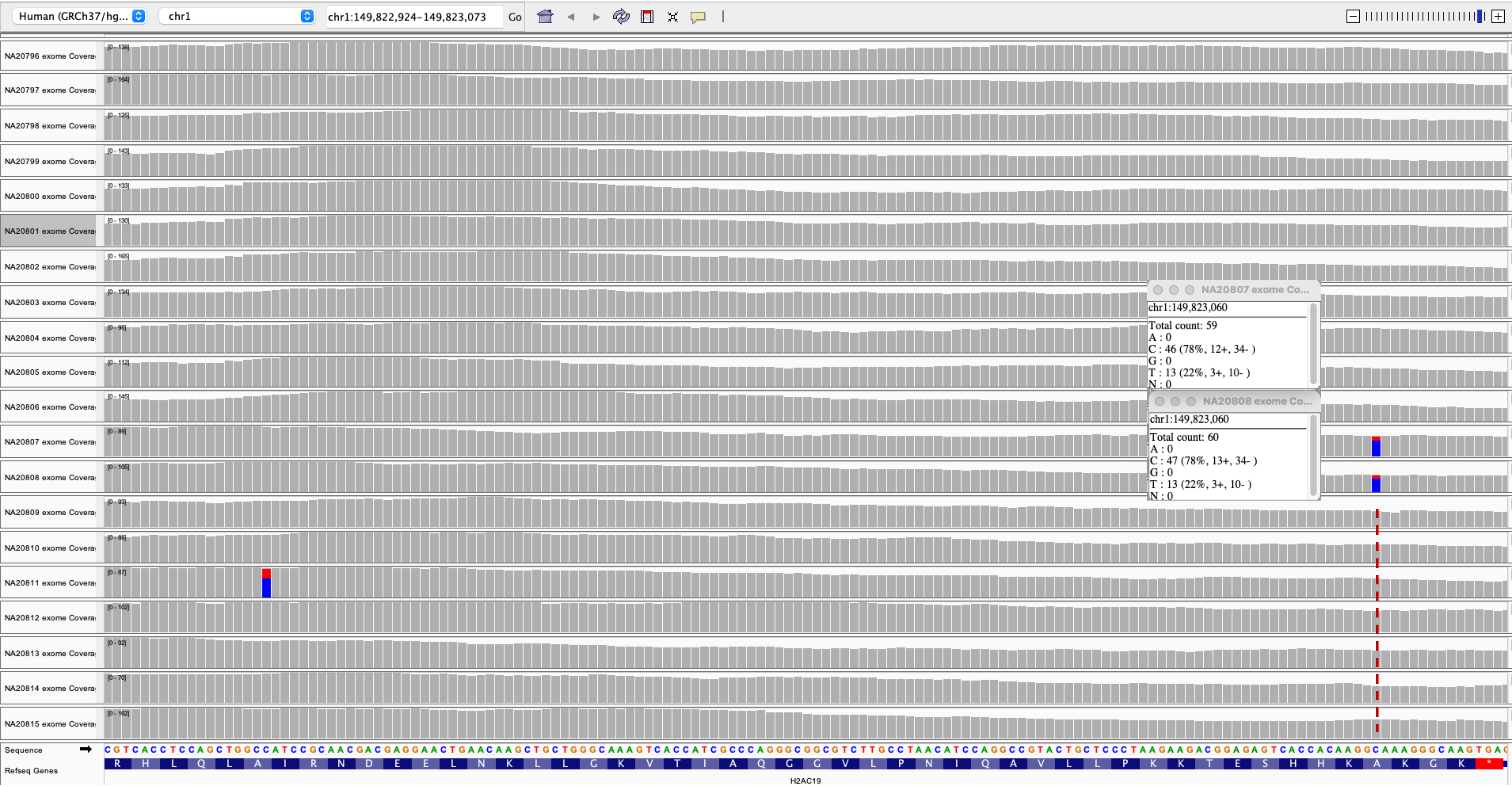

TSI: NA20818-NA20832

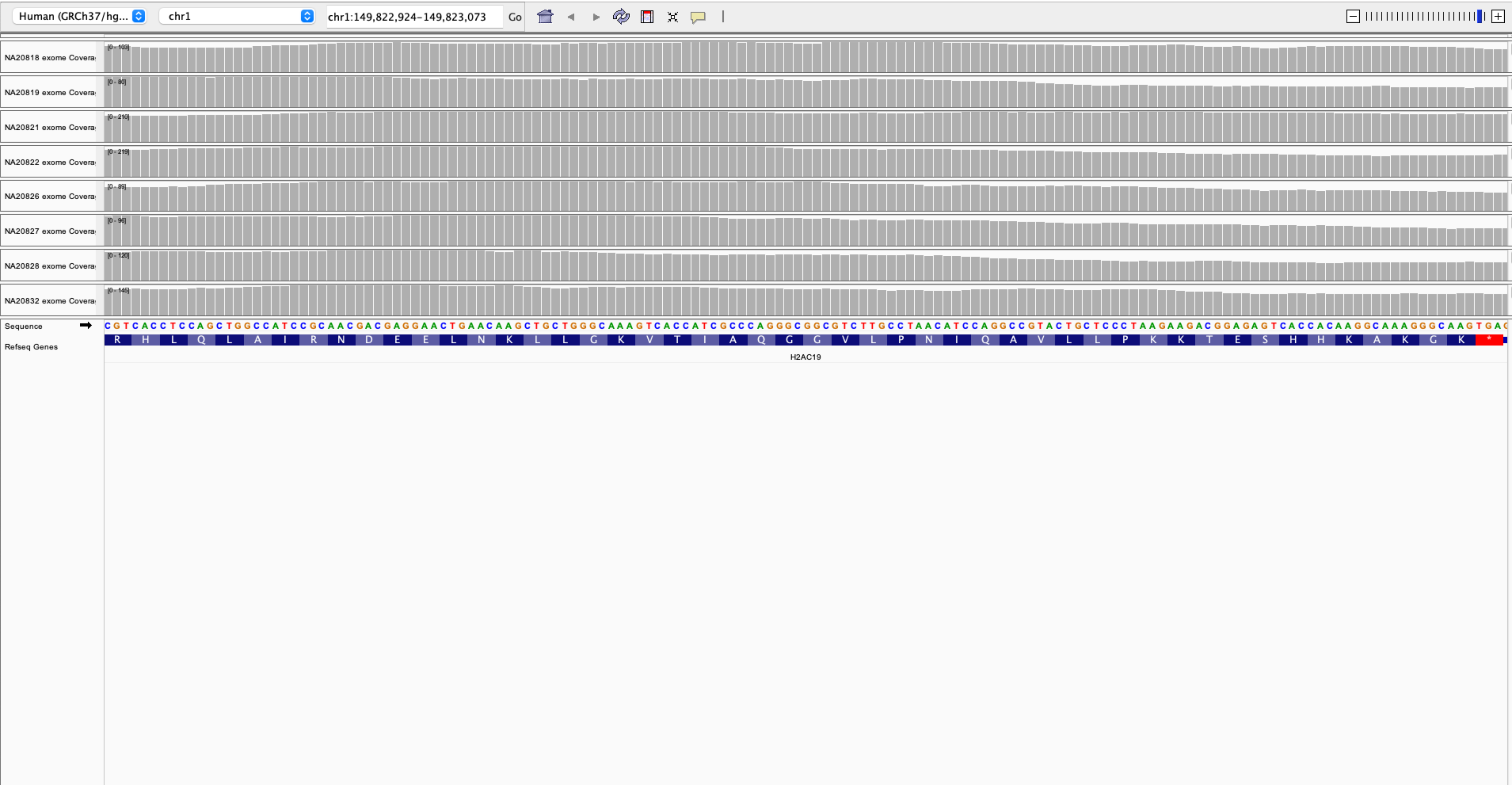

ESN: Esan in Nigeria
(99 samples)

ESN: HG02922-HG03099

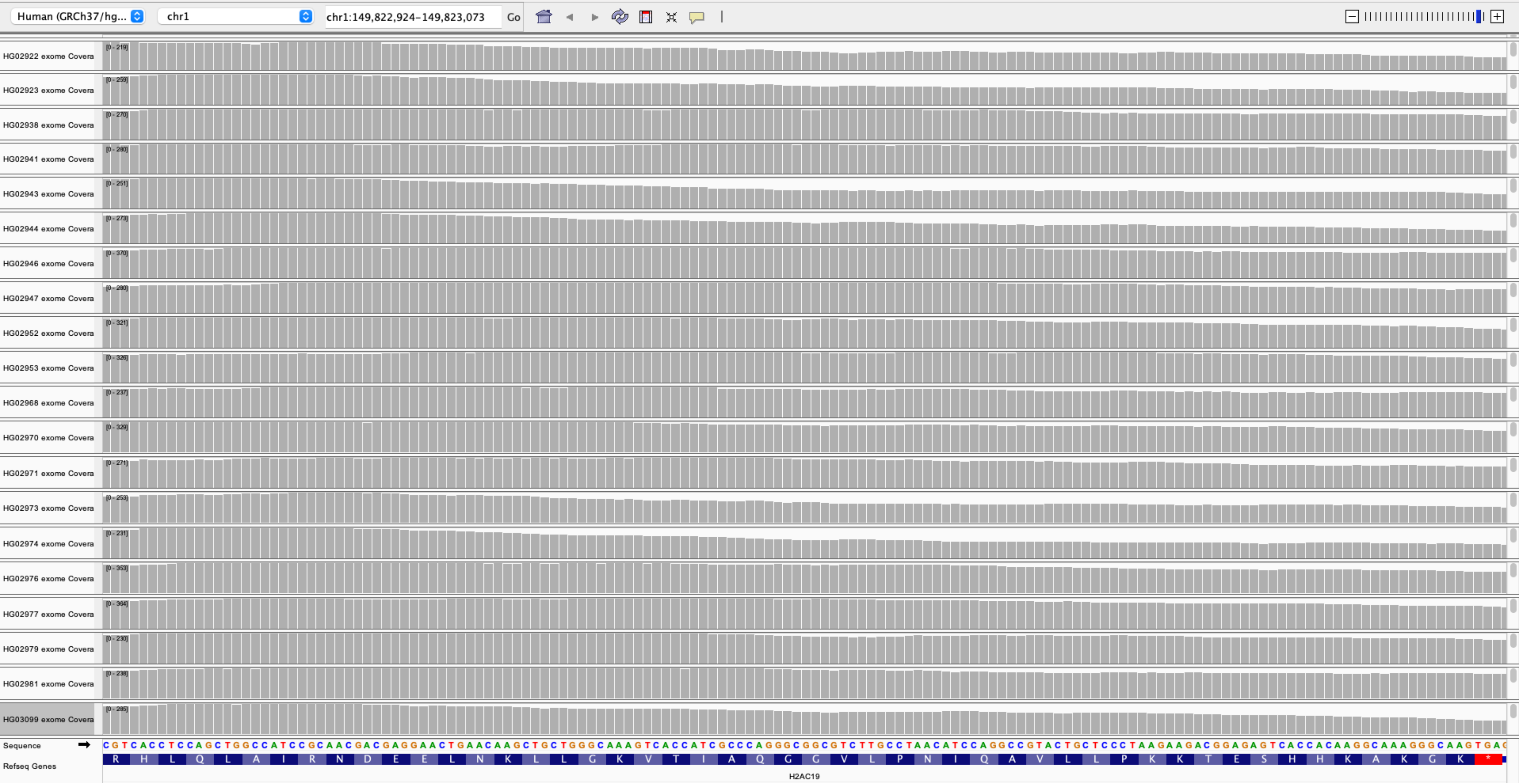

ESN: HG03100-HG03132

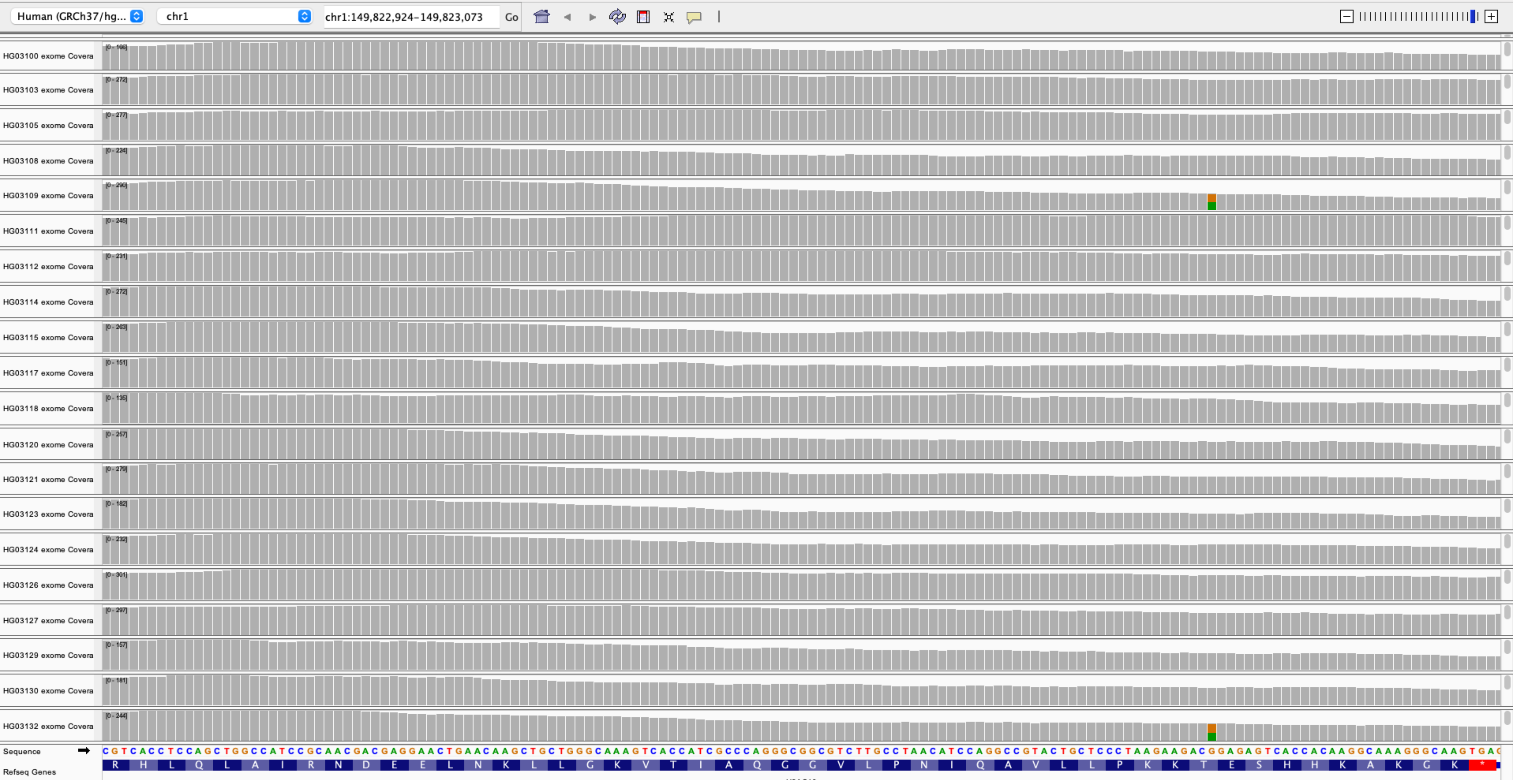

ESN: HG03133-HG03198

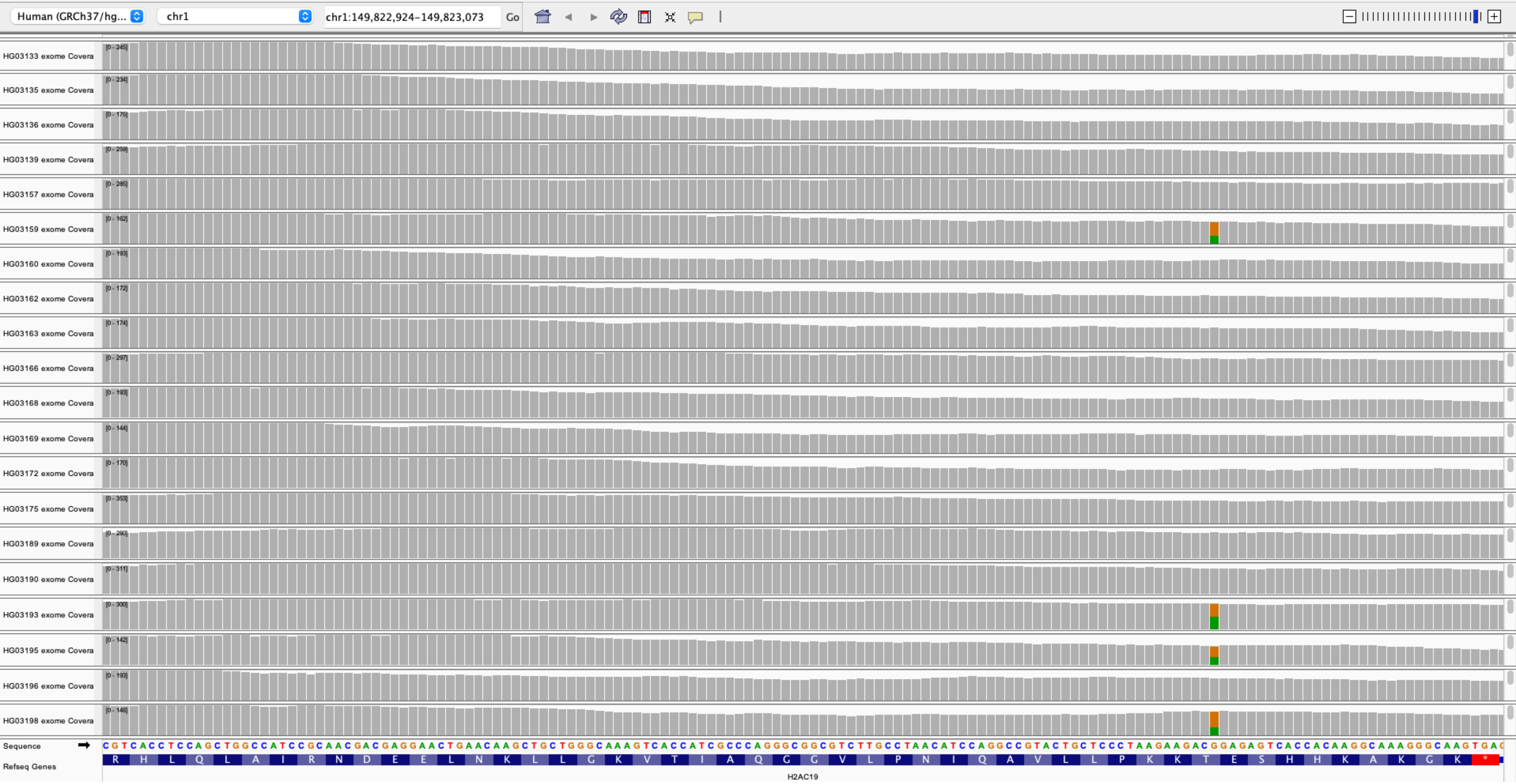

ESN: HG03199-HG03313

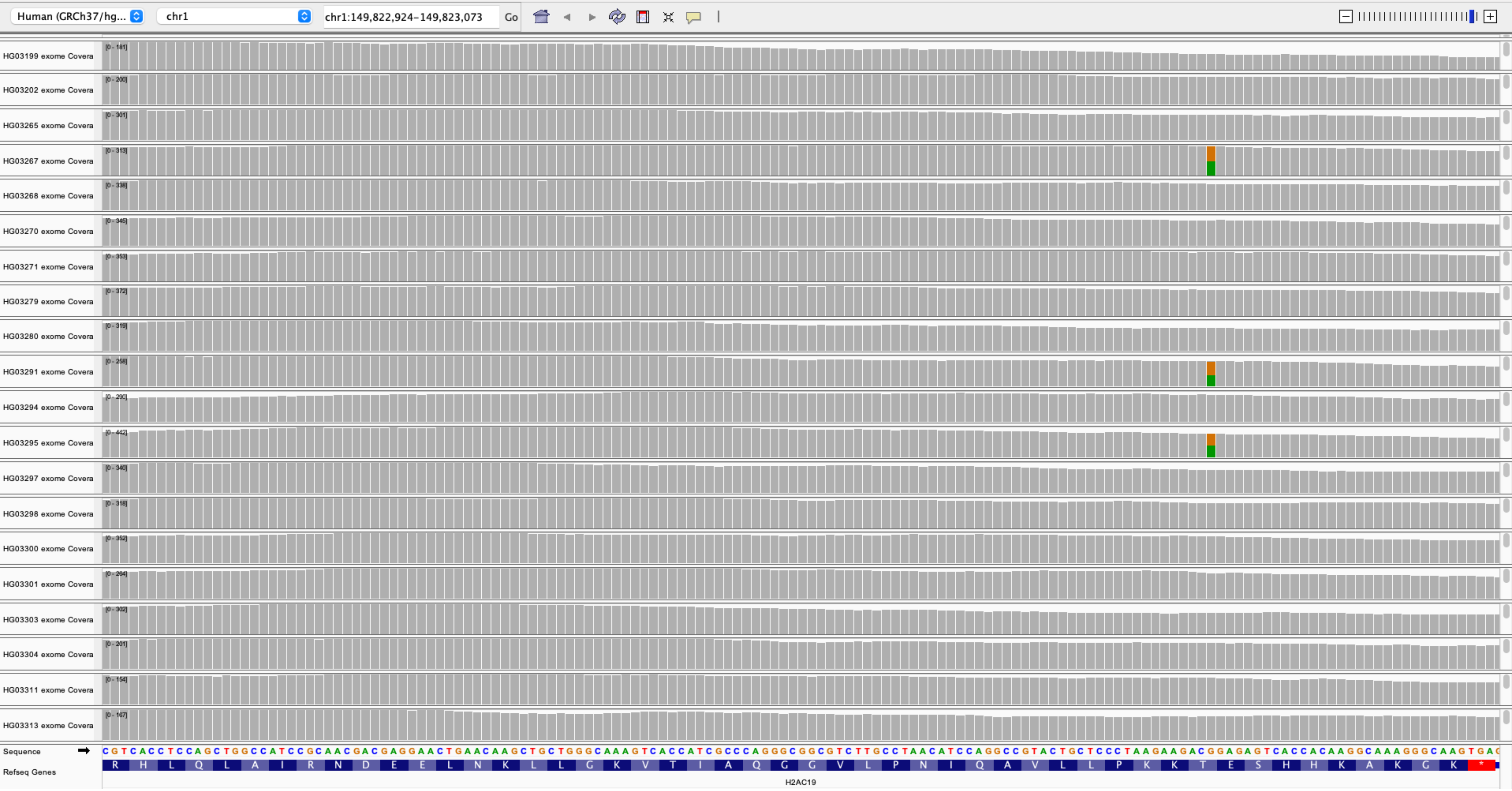

ESN: HG03342-HG03521

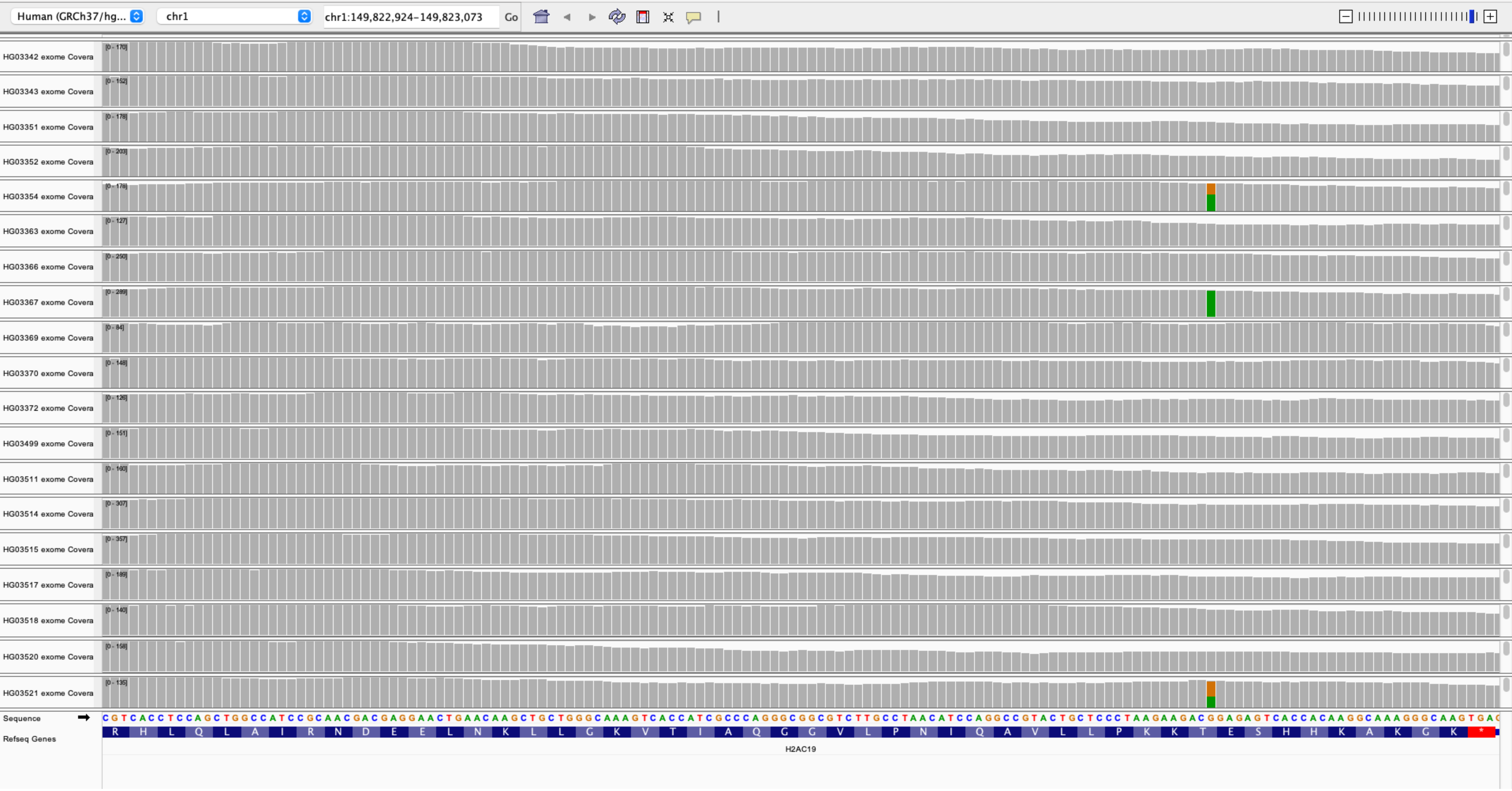

GWD: Gambian in Western Division, Mandinka (113 samples)

GWD: HG02461-HG02610

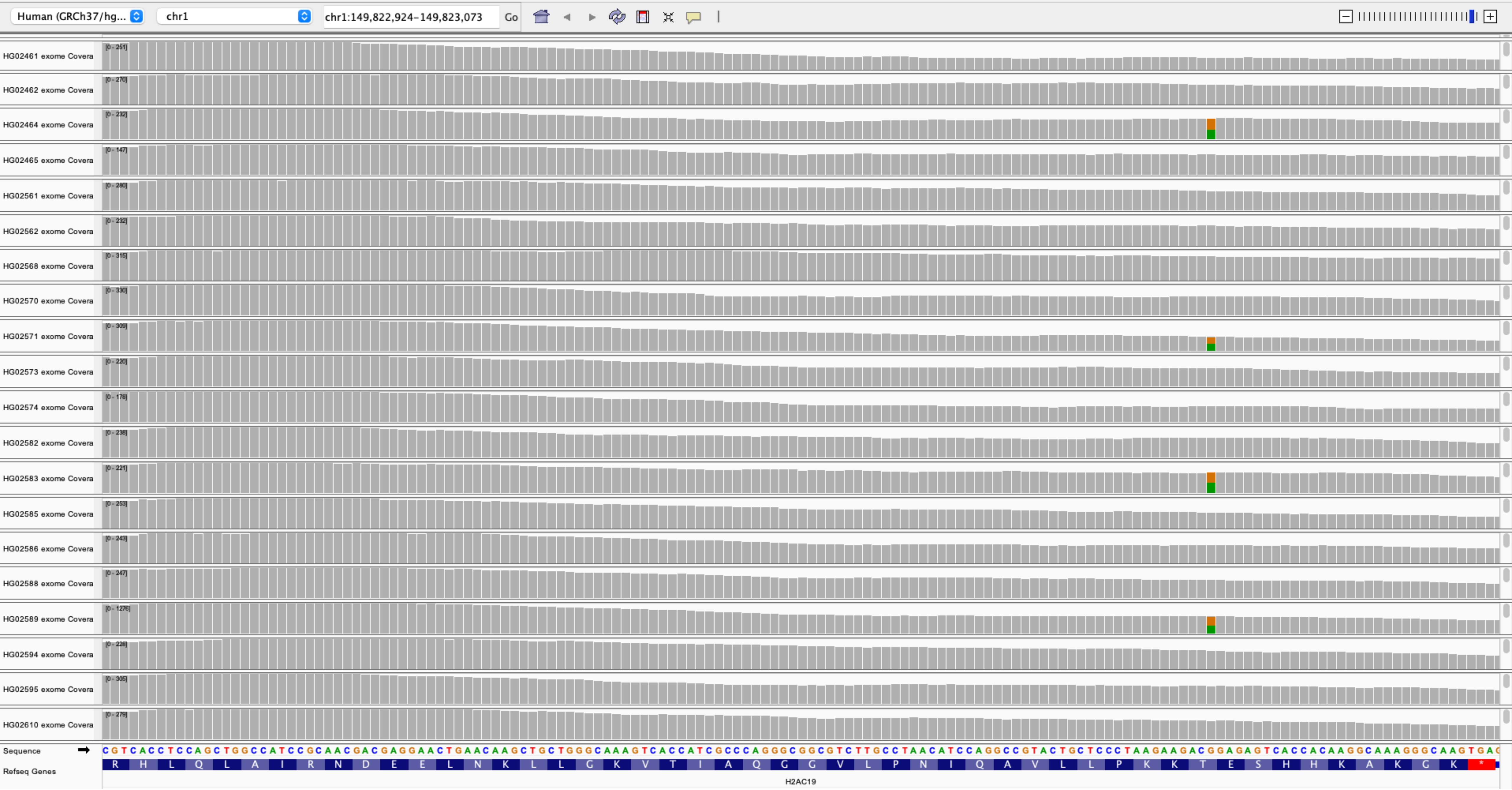

GWD: HG02611-HG02678

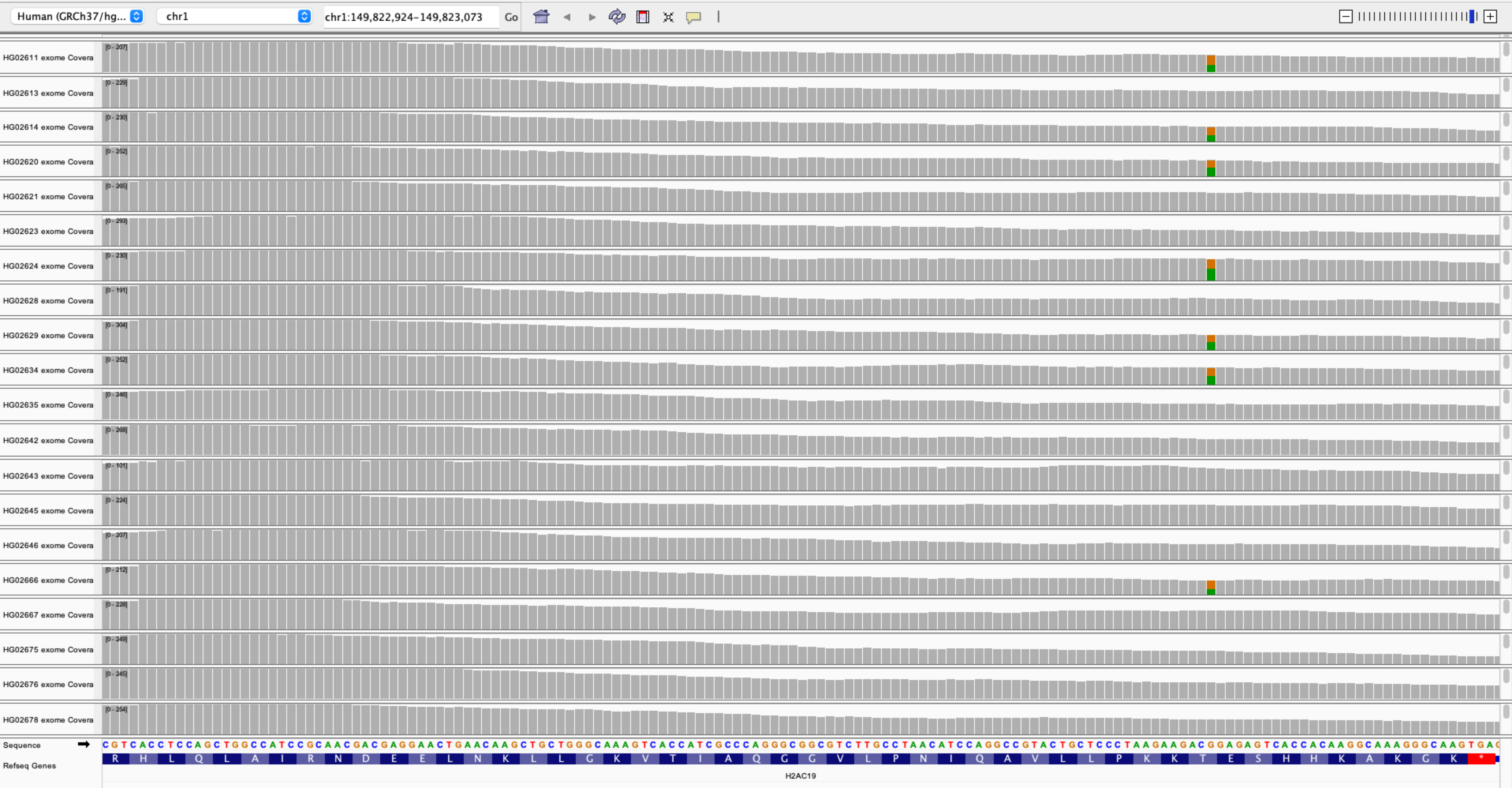

GWD: HG02679-HG02805

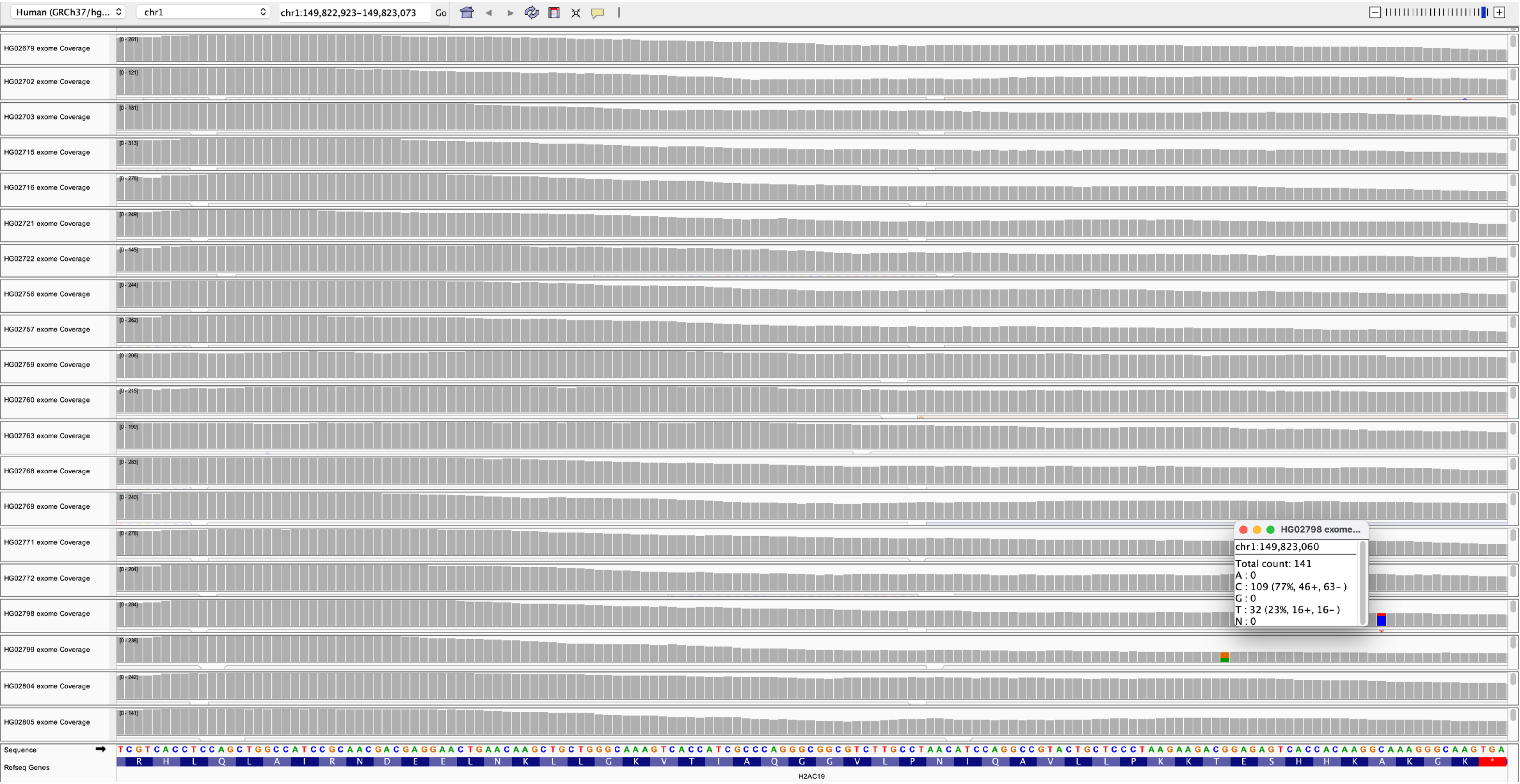

GWD: HG02807-HG02861

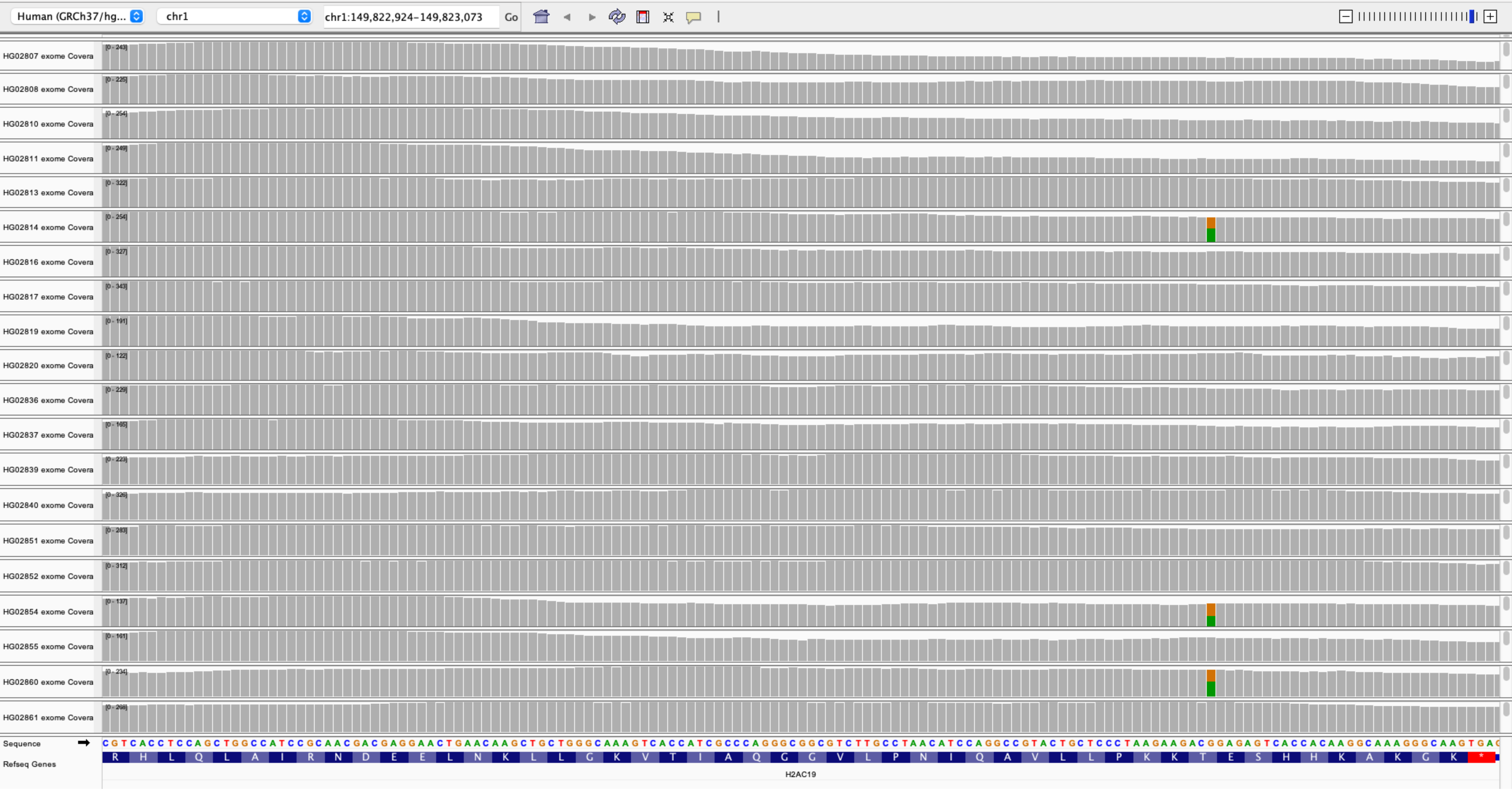

GWD: HG02870-HG03039

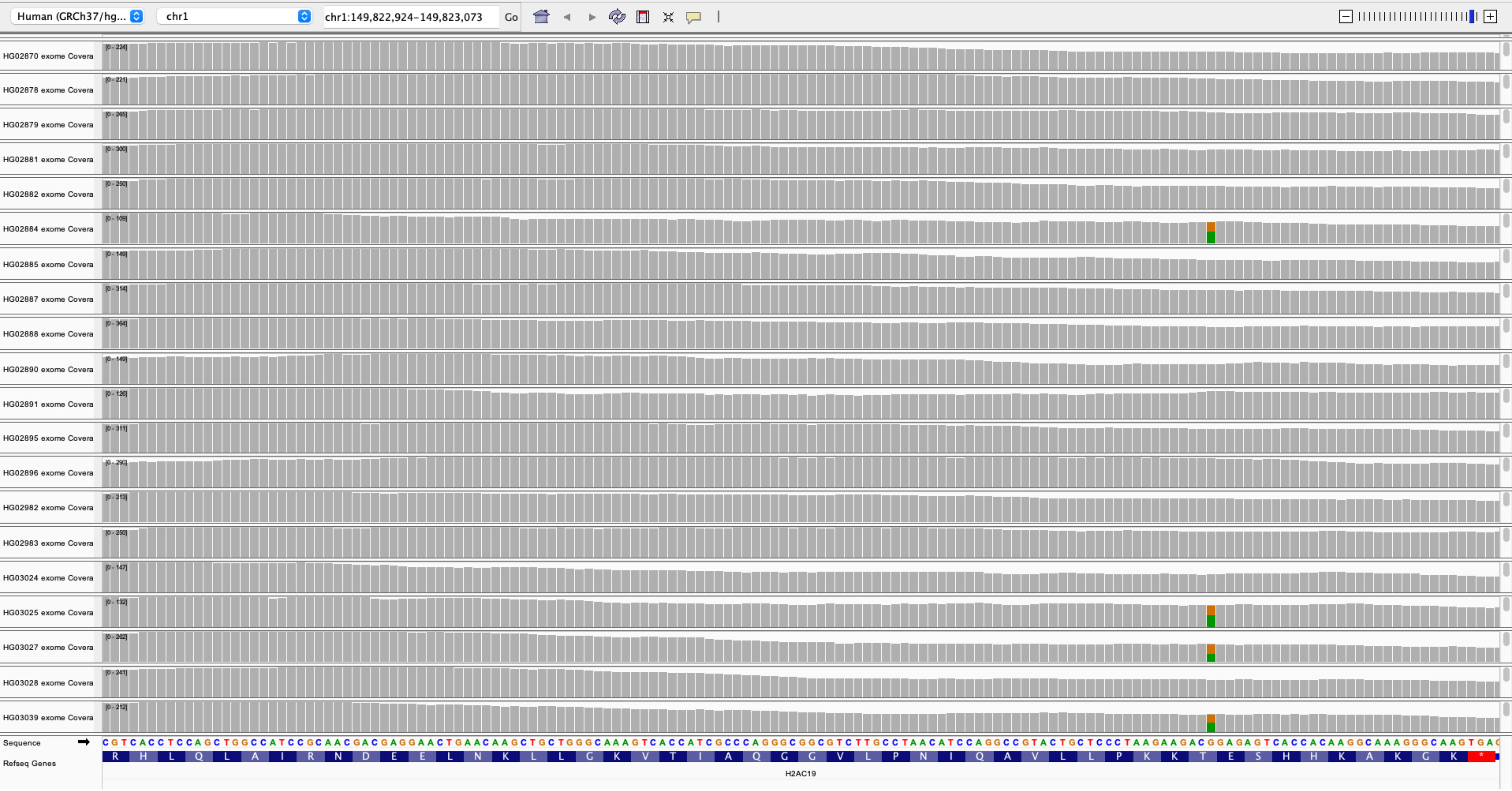

GWD.HG03040-HG03539

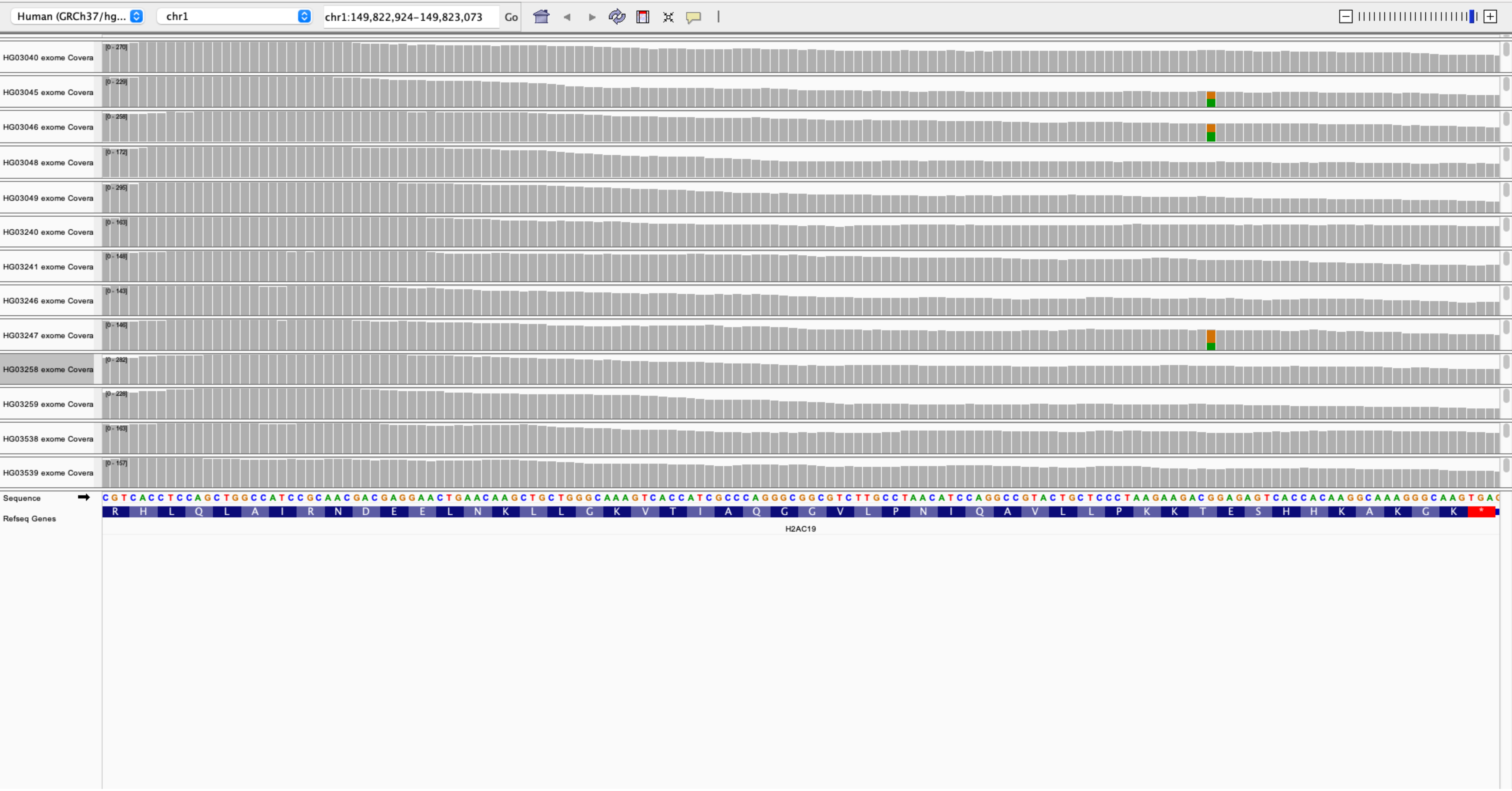

LWK: Luhya in Webuye, Kenya
(101 samples)

LWK: NA19017-NA19308

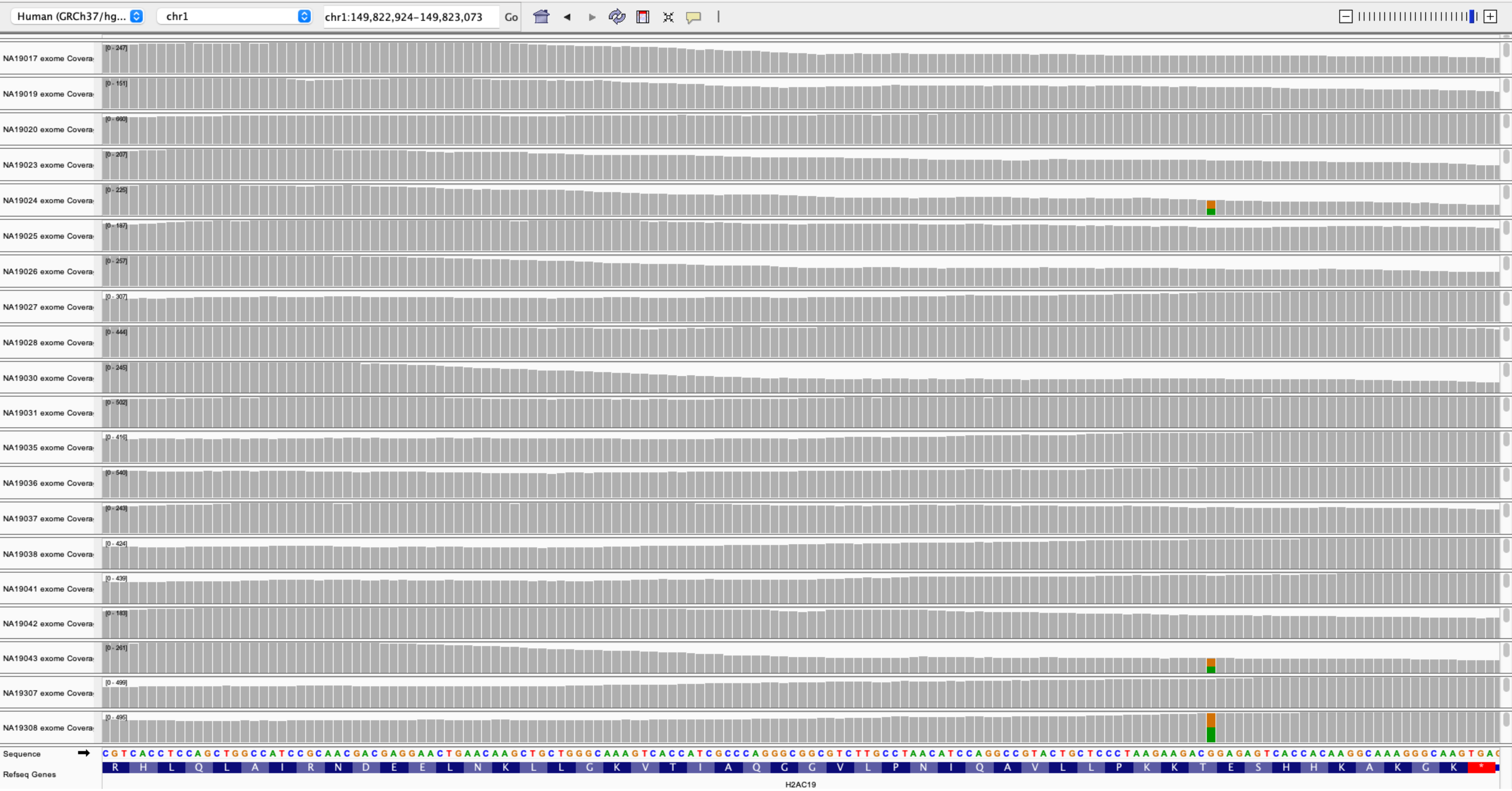

LWK: NA19309-NA19334

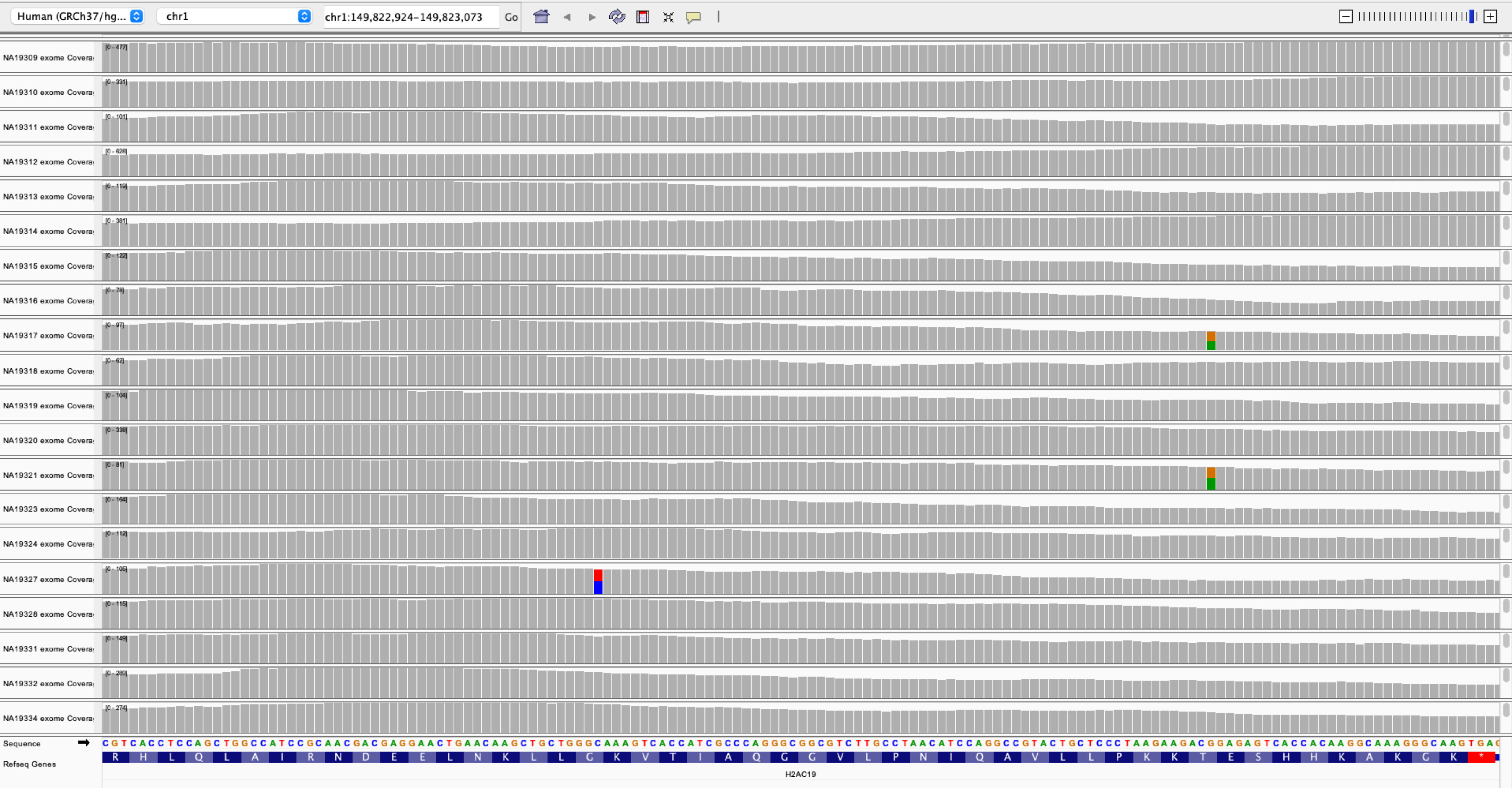

LWK: NA19338-NA19391

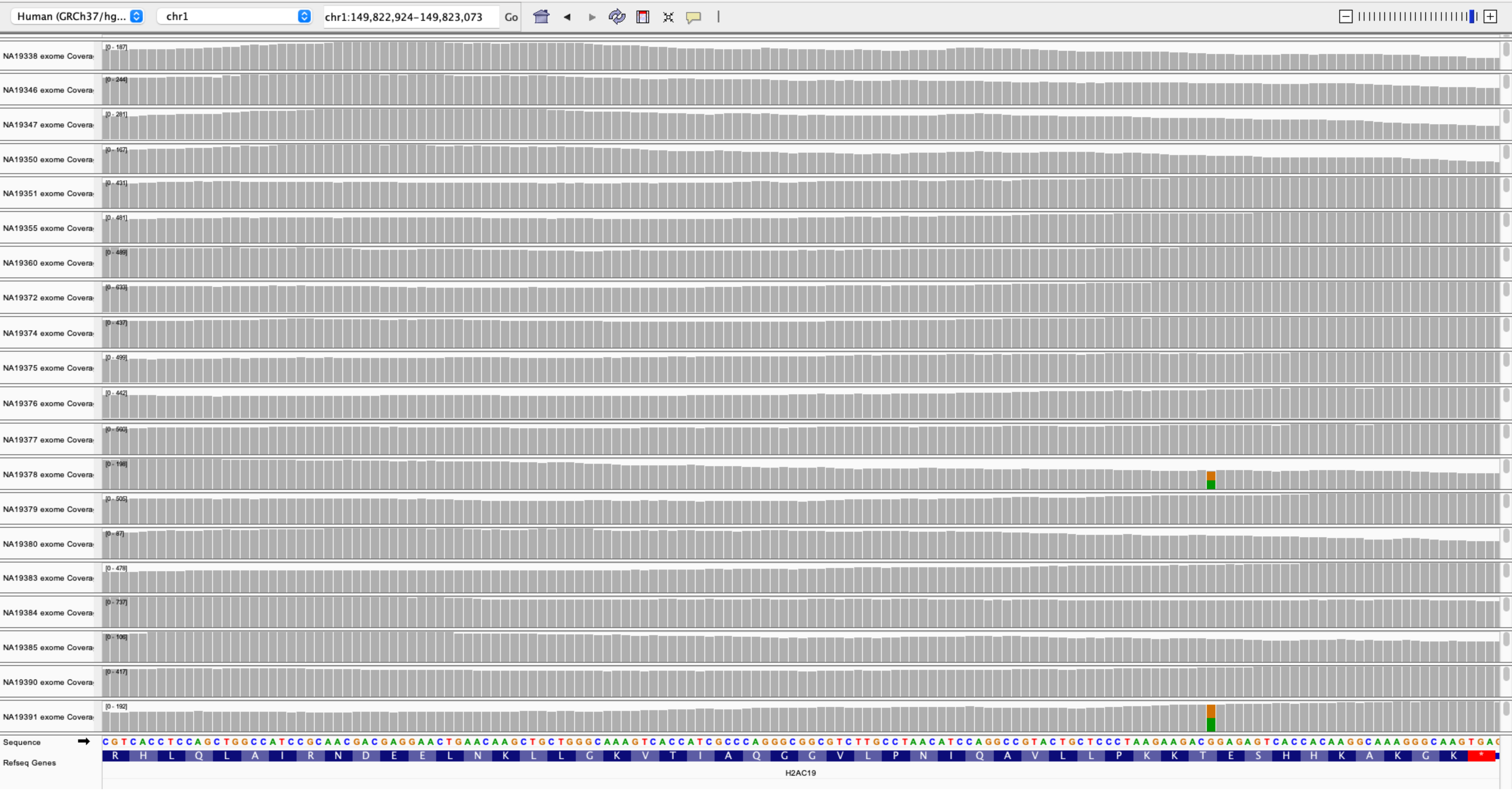

LWK: NA19393-NA19443

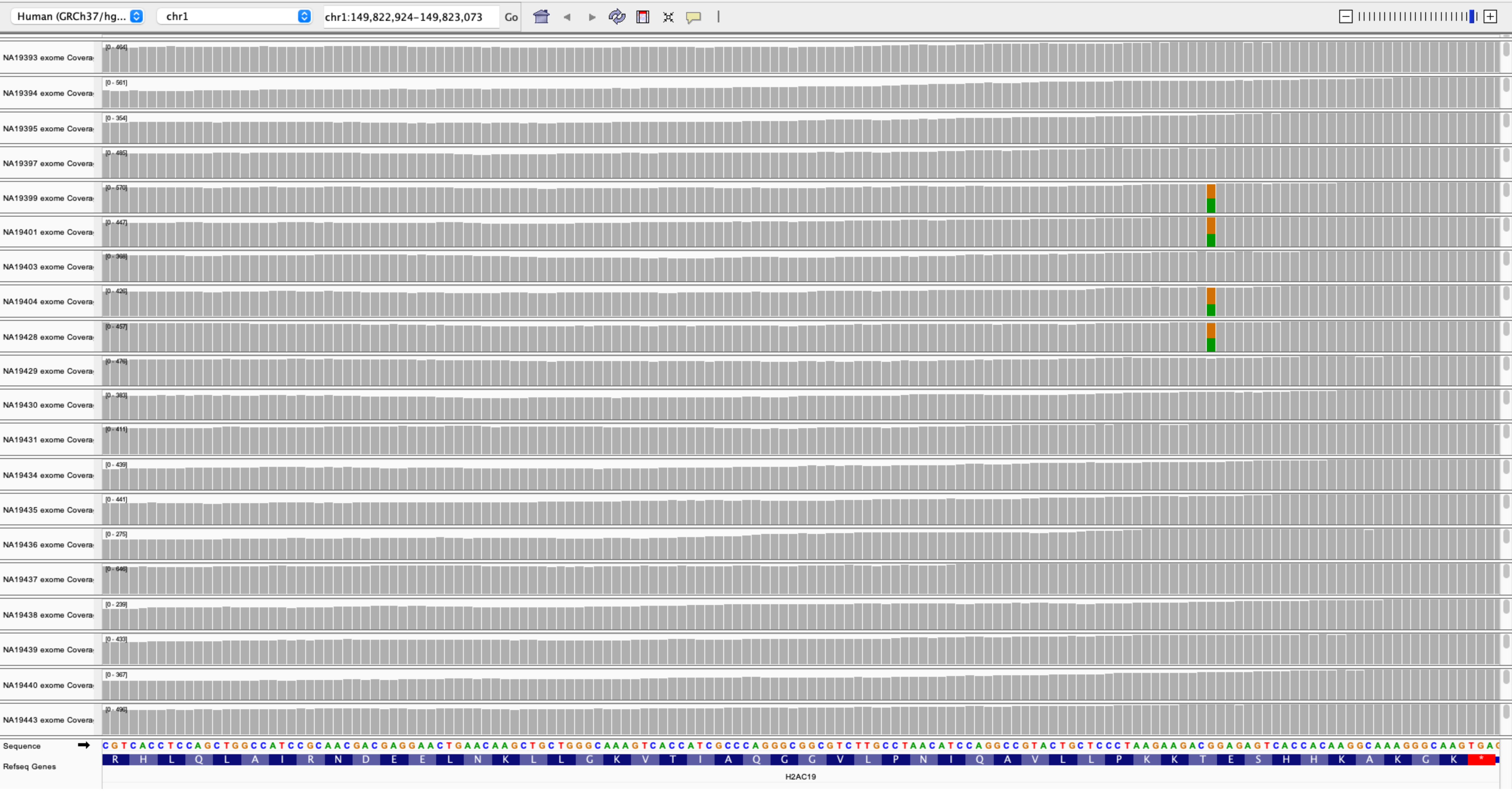

LWK: NA19445-NA19474

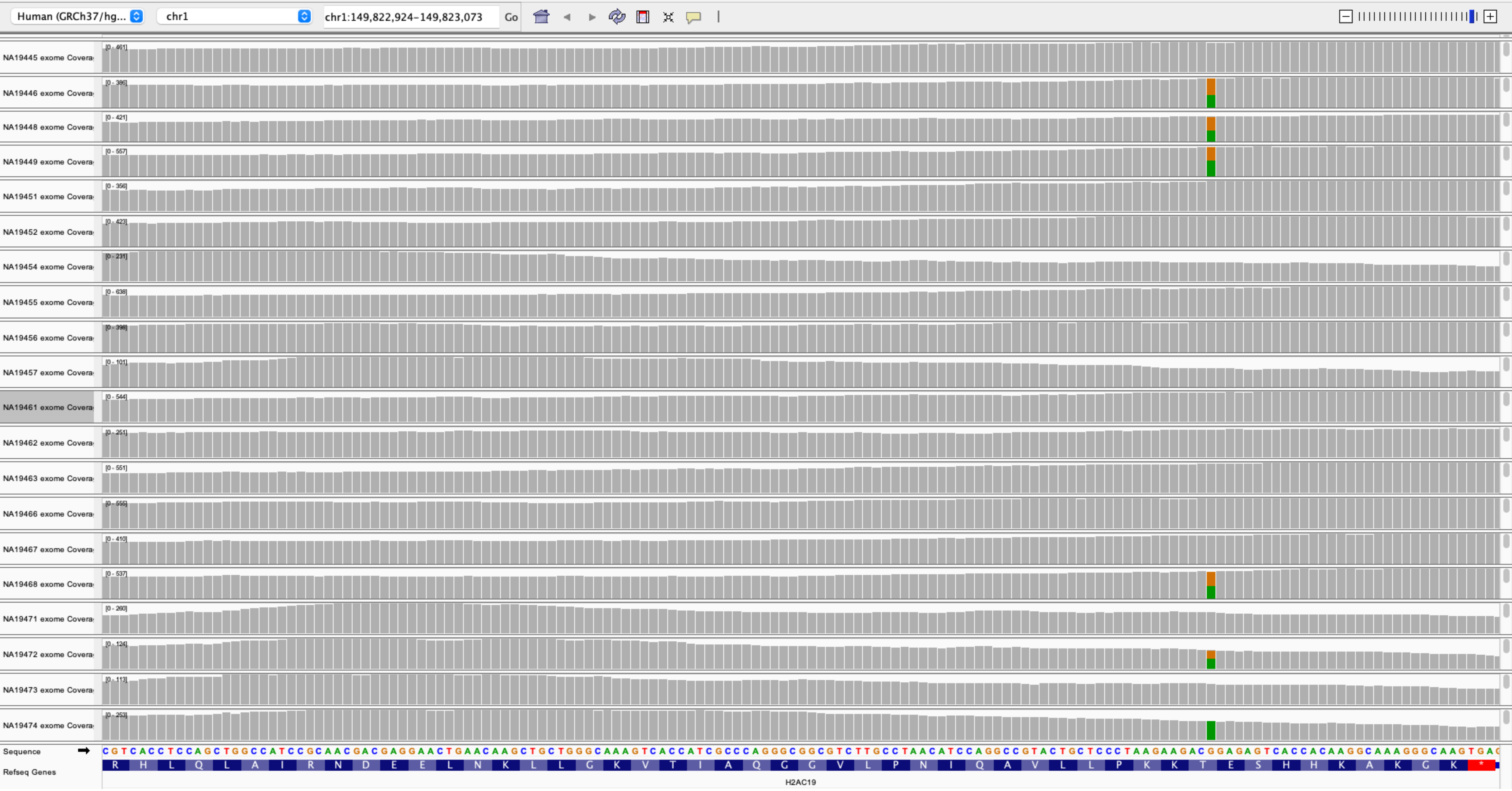

LWK: NA19475

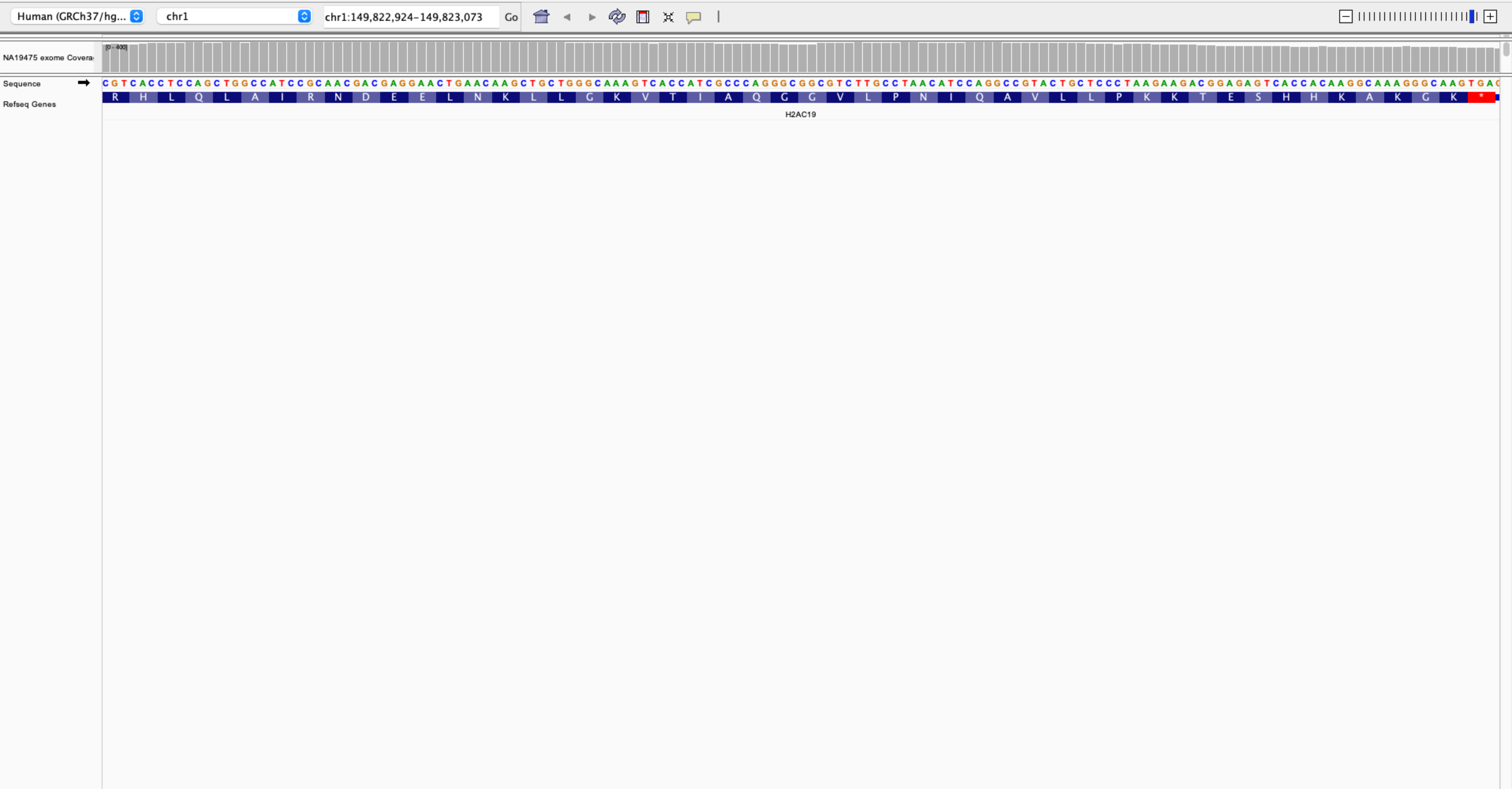
Human (GRCh37/hg...
chr1
chr1:149,822,924-149,823,073
Go
NA19475 exome Covera
[0 - 400]
Sequence
CGTCACCTCCAGCTGGCCATCCGCAACGACGAGGAACTGAACAAGCTGCTGGGCAAAGTCACCATCGCCCAGGGCGGCGTCTTGCCTAACATCCAGGCCGTACTGCTCCCTAAGAAGACGGAGAGTCACCACAAGGCAAAGGGCAAGTGAG
Refseq Genes
R H L Q L A I R N D E E L N K L L G K V T I A Q G G V L P N I Q A V L L P K K T E S H H K A K G K *
H2AC19

YRI: Yoruba in Ibadan, Nigeria
(109 samples)

YRI: NA18486-NA18853

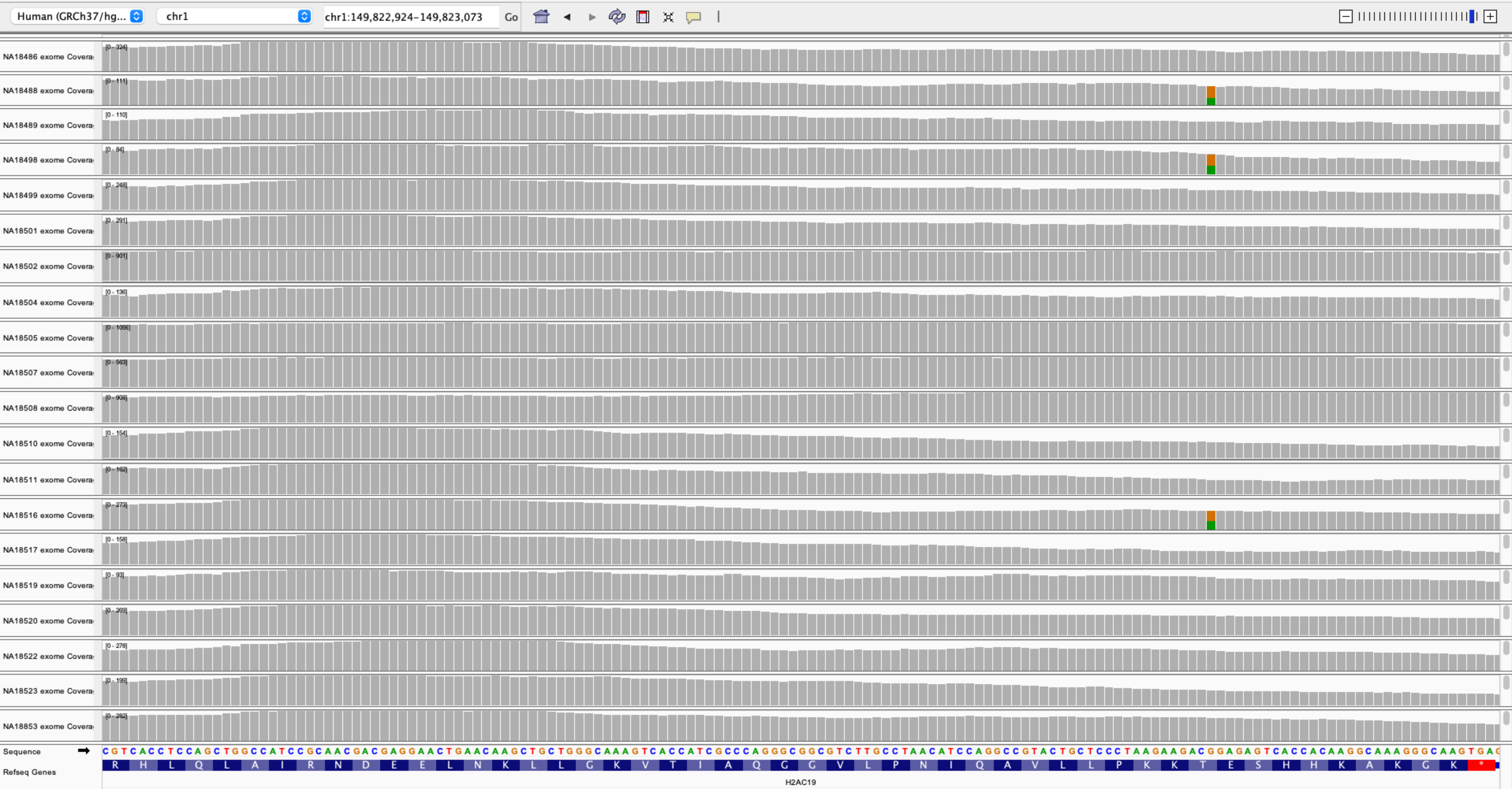

YRI: NA18856-NA18910

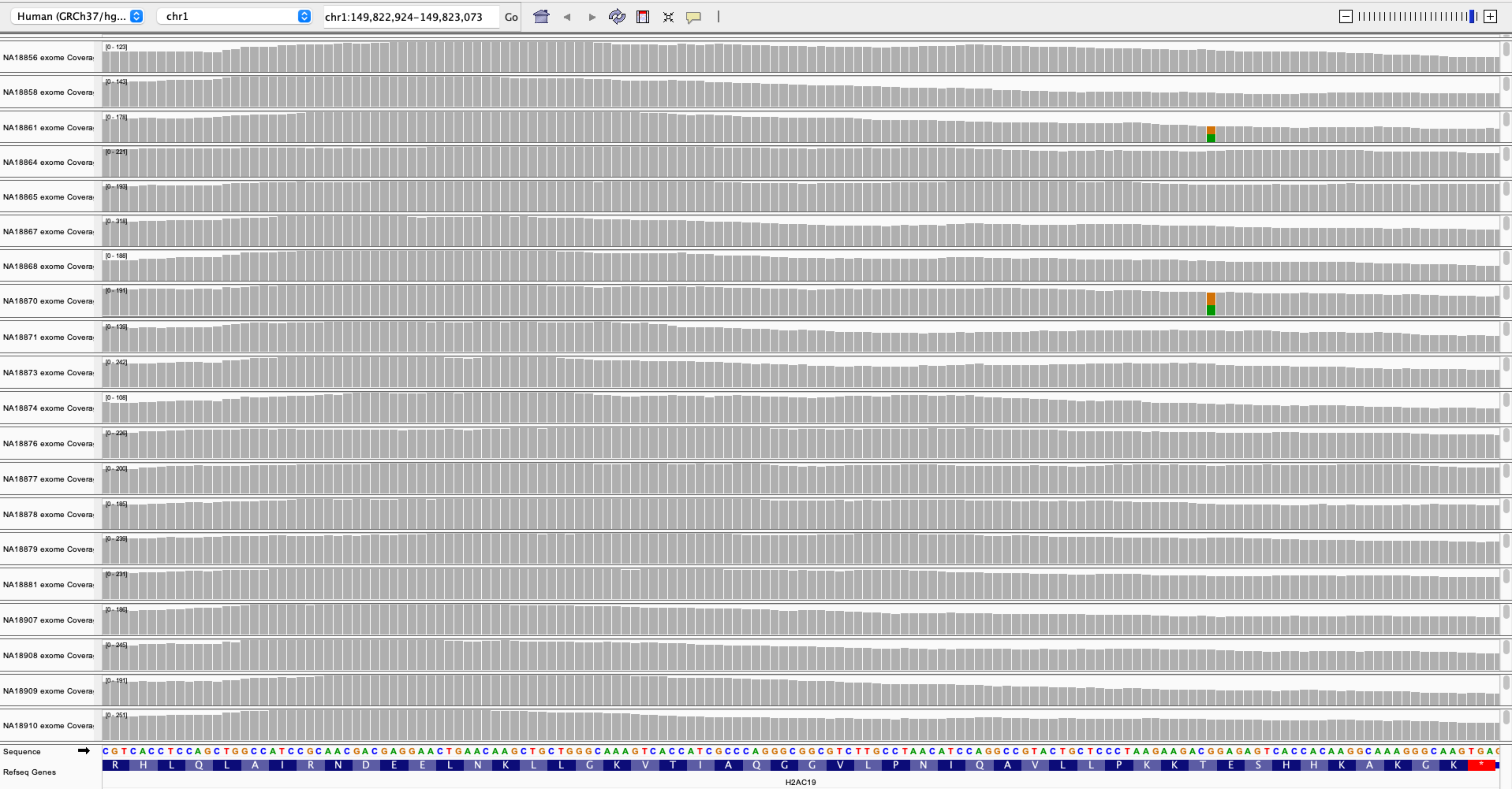

YRI: NA18912-NA19116

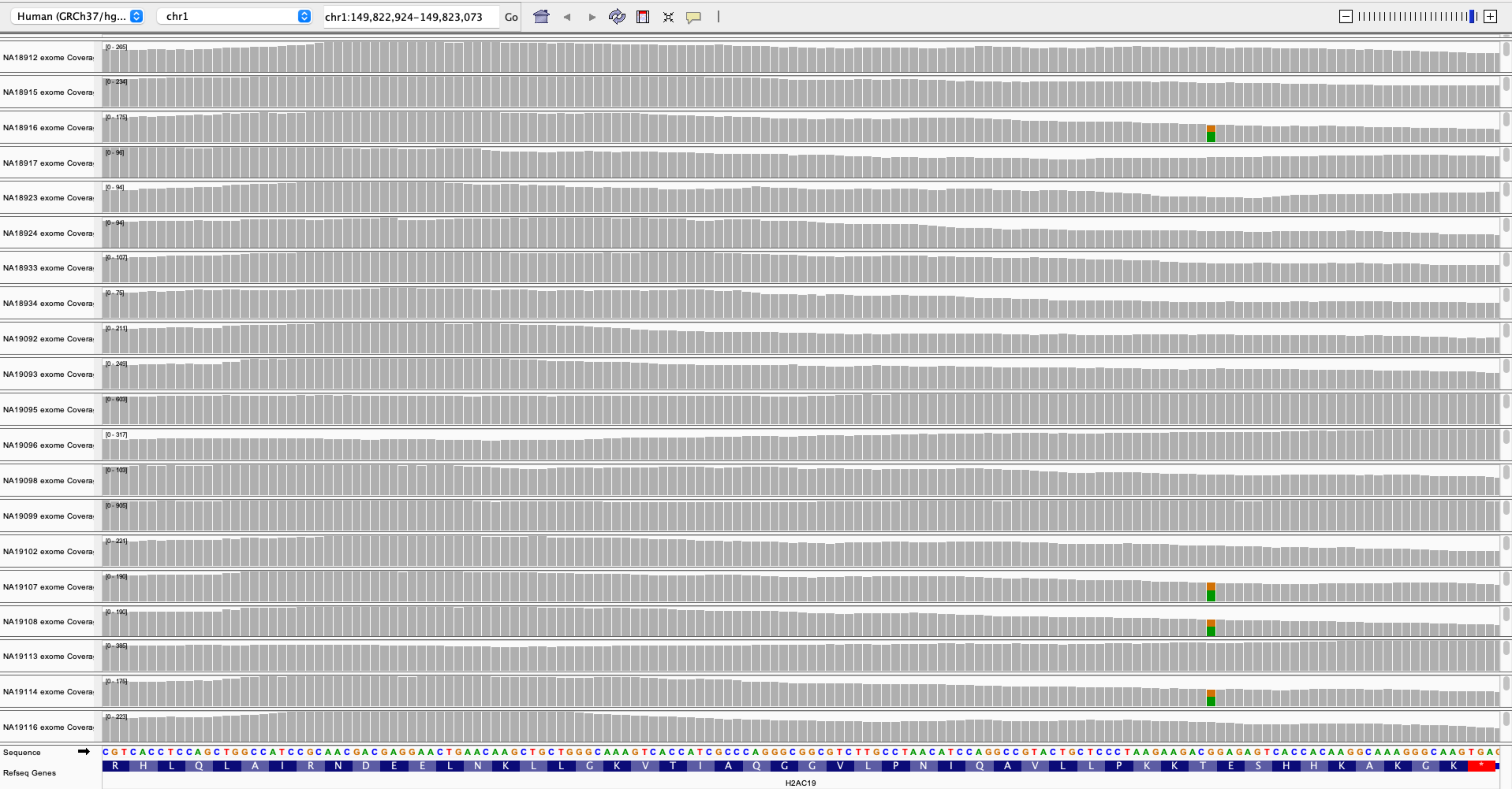

YRI: NA19117-NA19171

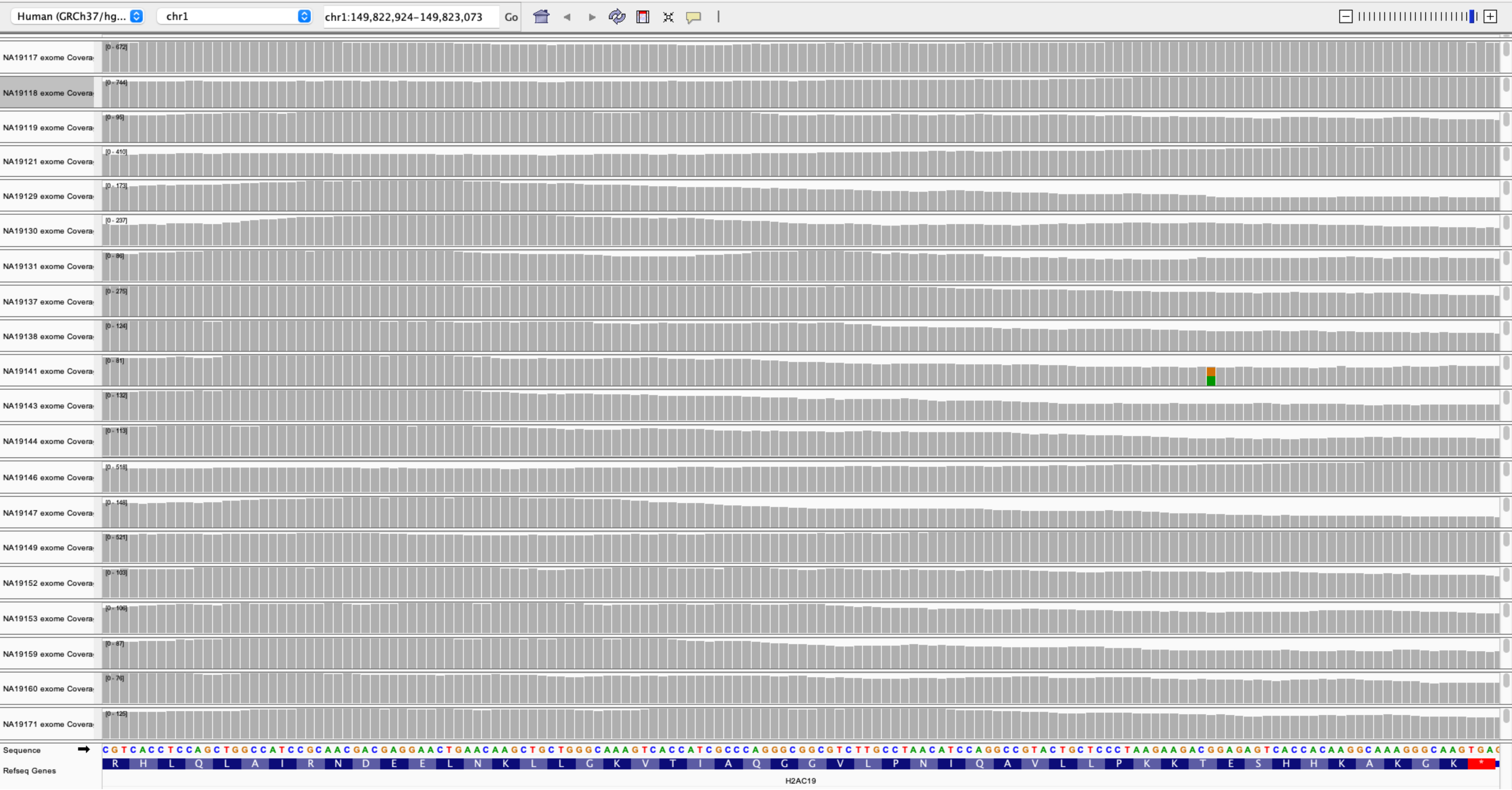

YRI: NA19172-NA19225

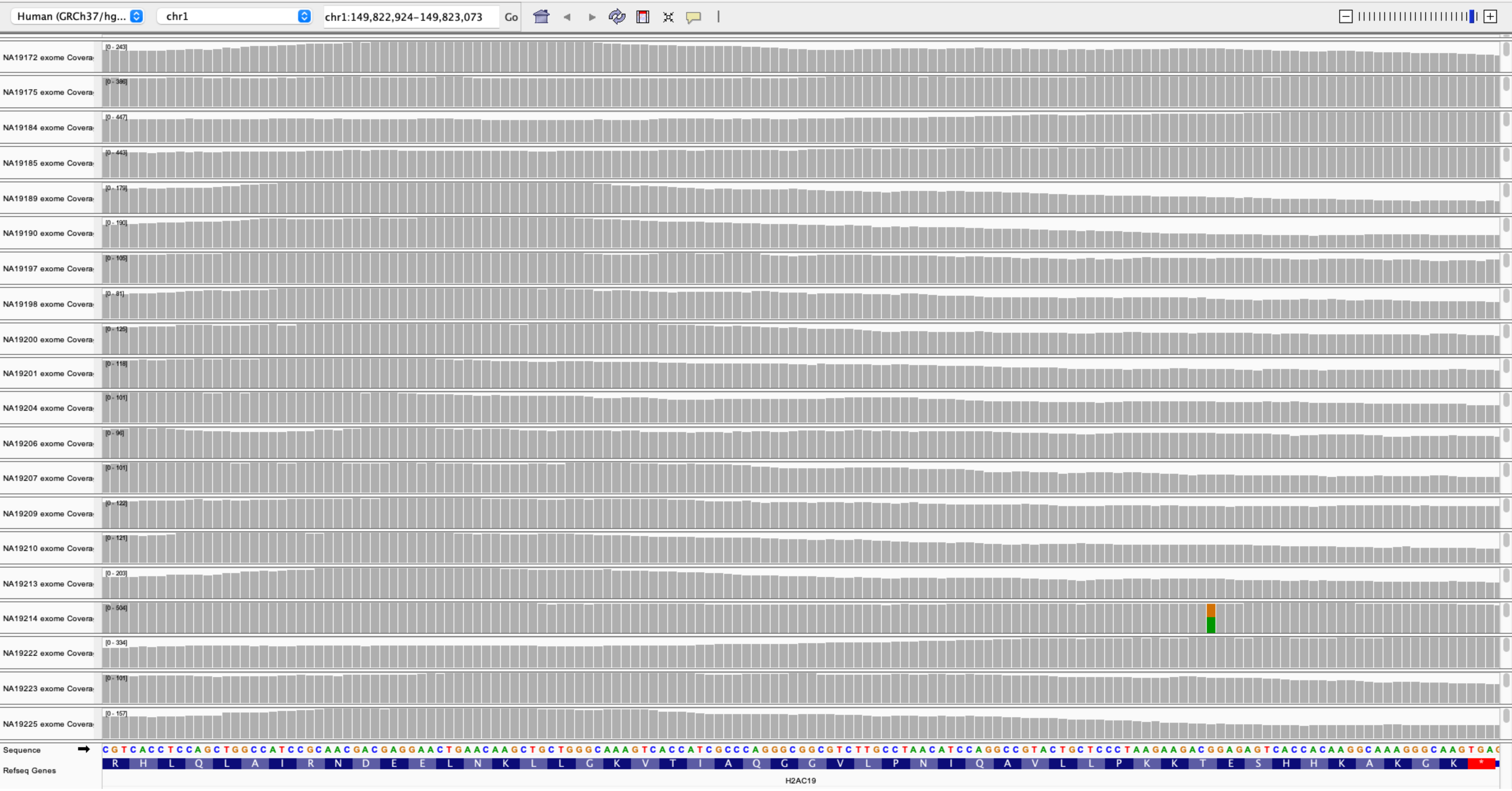

YRI: NA19235-NA19257

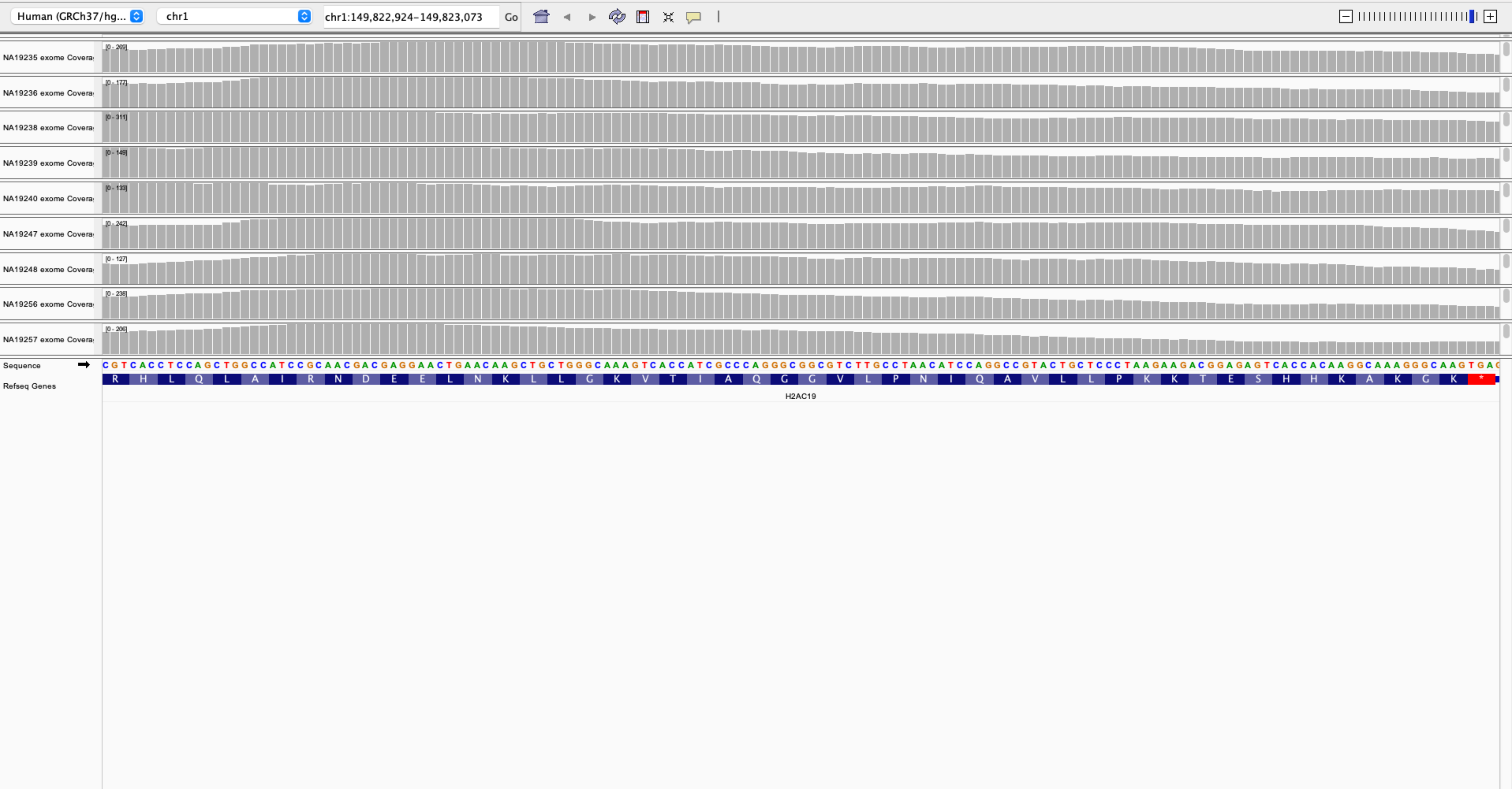

MSL: Mende in Sierra Leone
(85 samples)

MSL: HG03052-HG03084

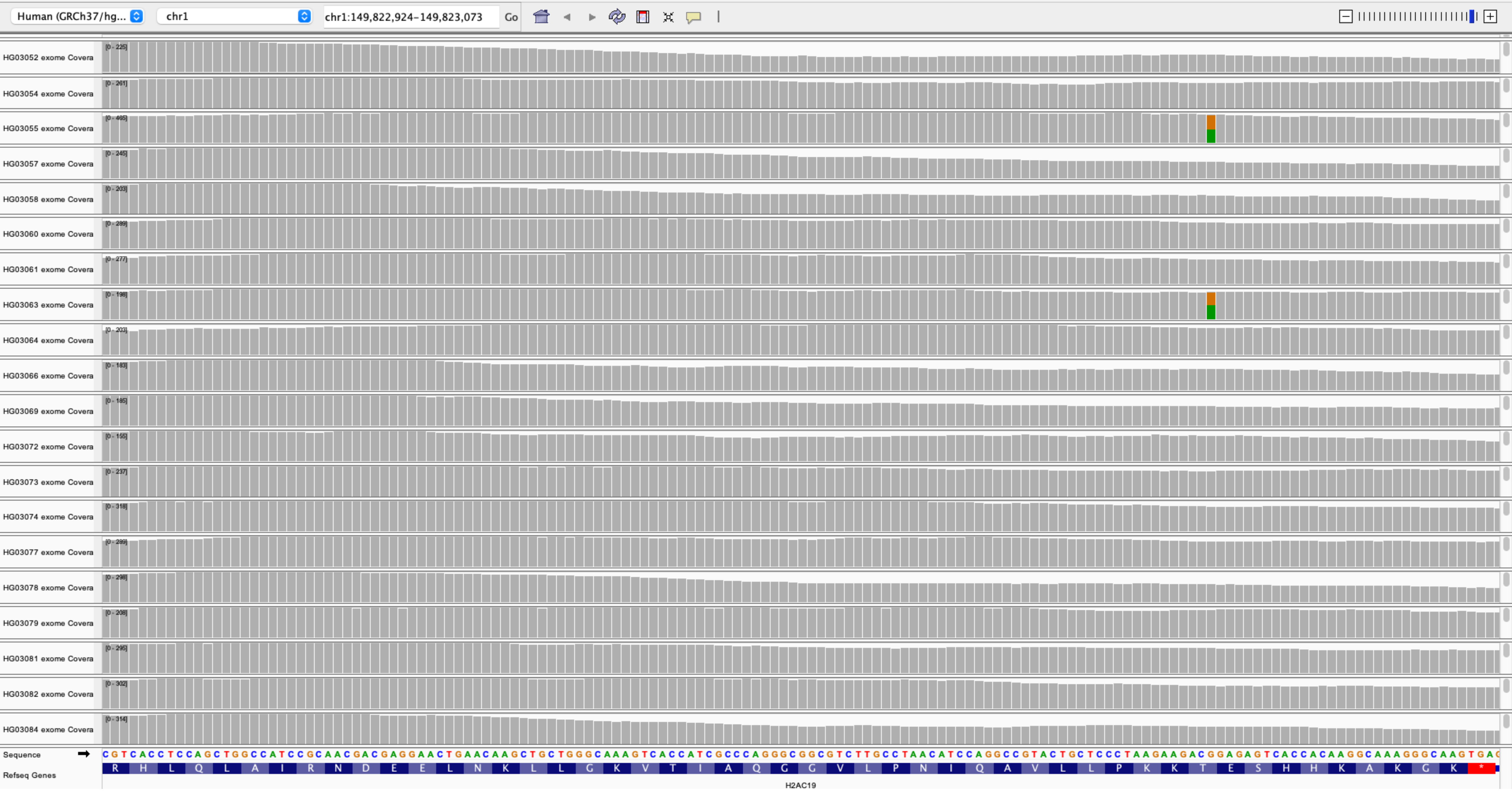

MSL: HG03085-HG03397

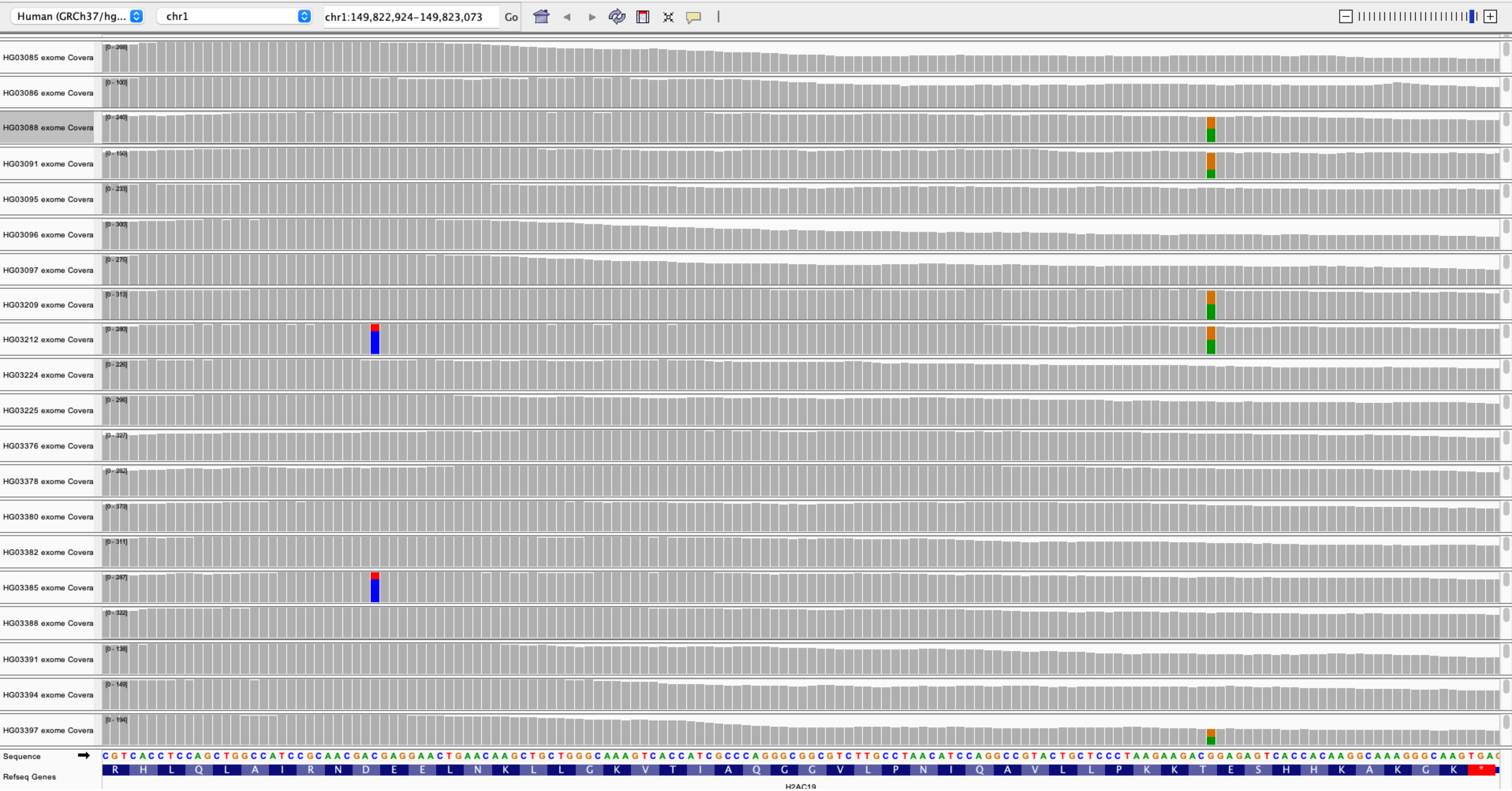

MSL: HG03401-HG03461

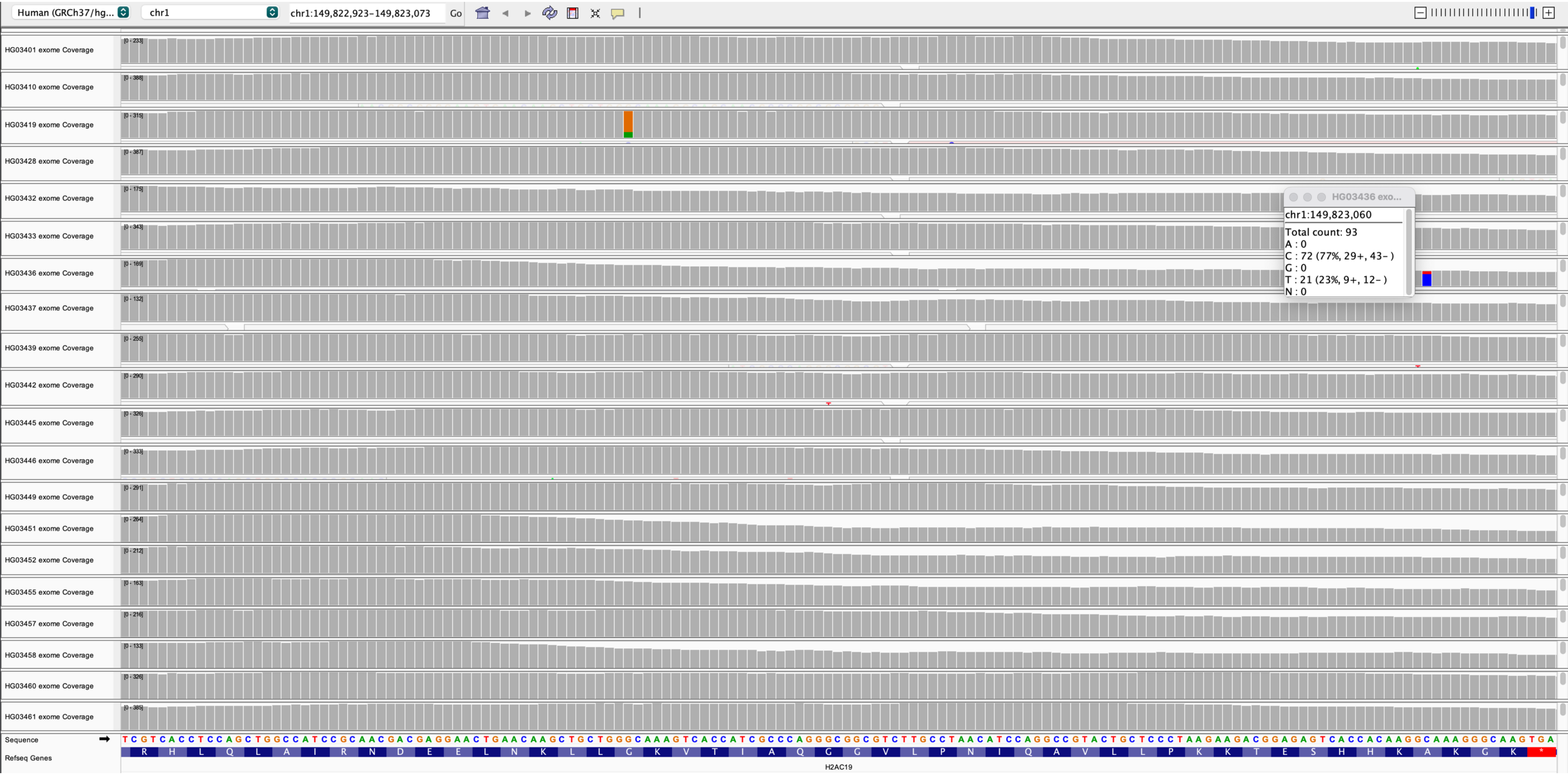

MSL: HG03464-HG03571

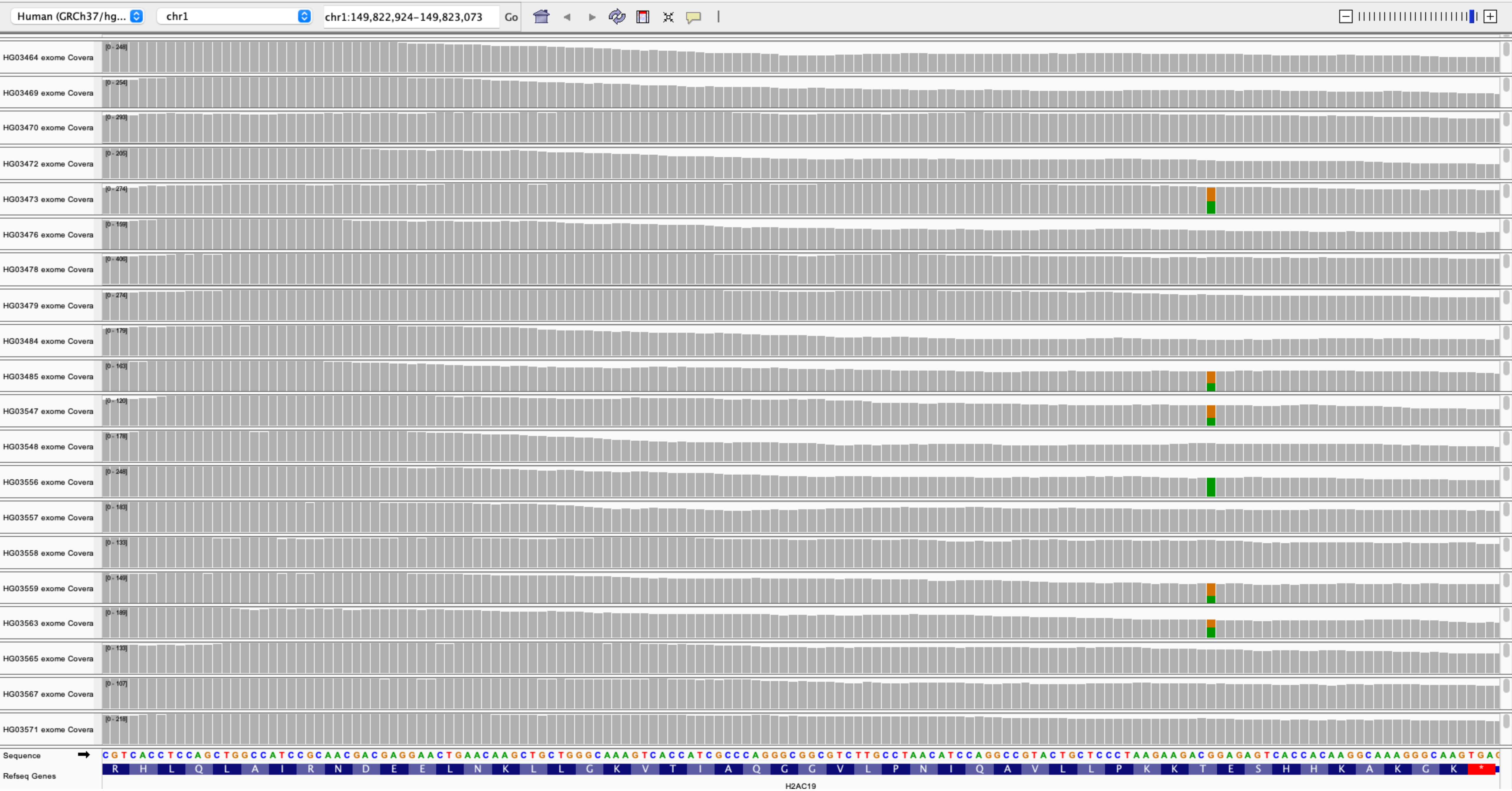

MSL: HG03572-HG03583

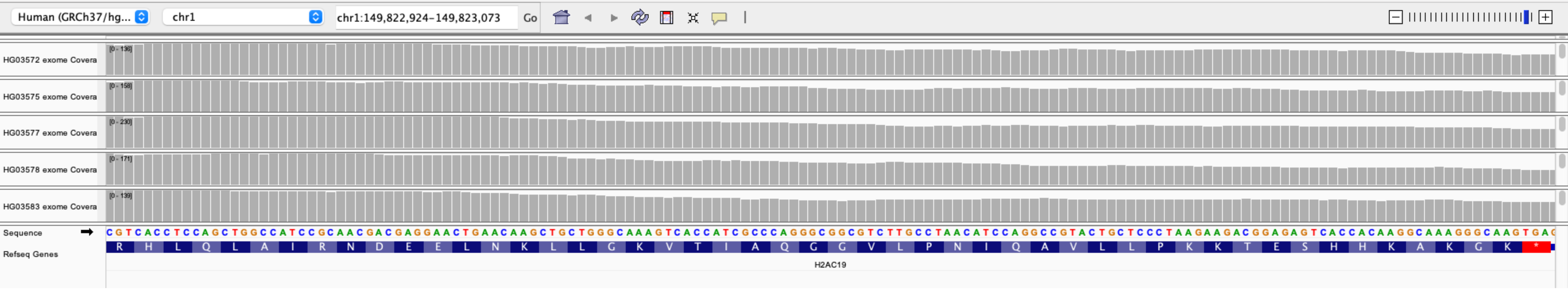

CLM: Colombians in Medellin, Colombia (94 samples)

CLM: HG01112-HG01251

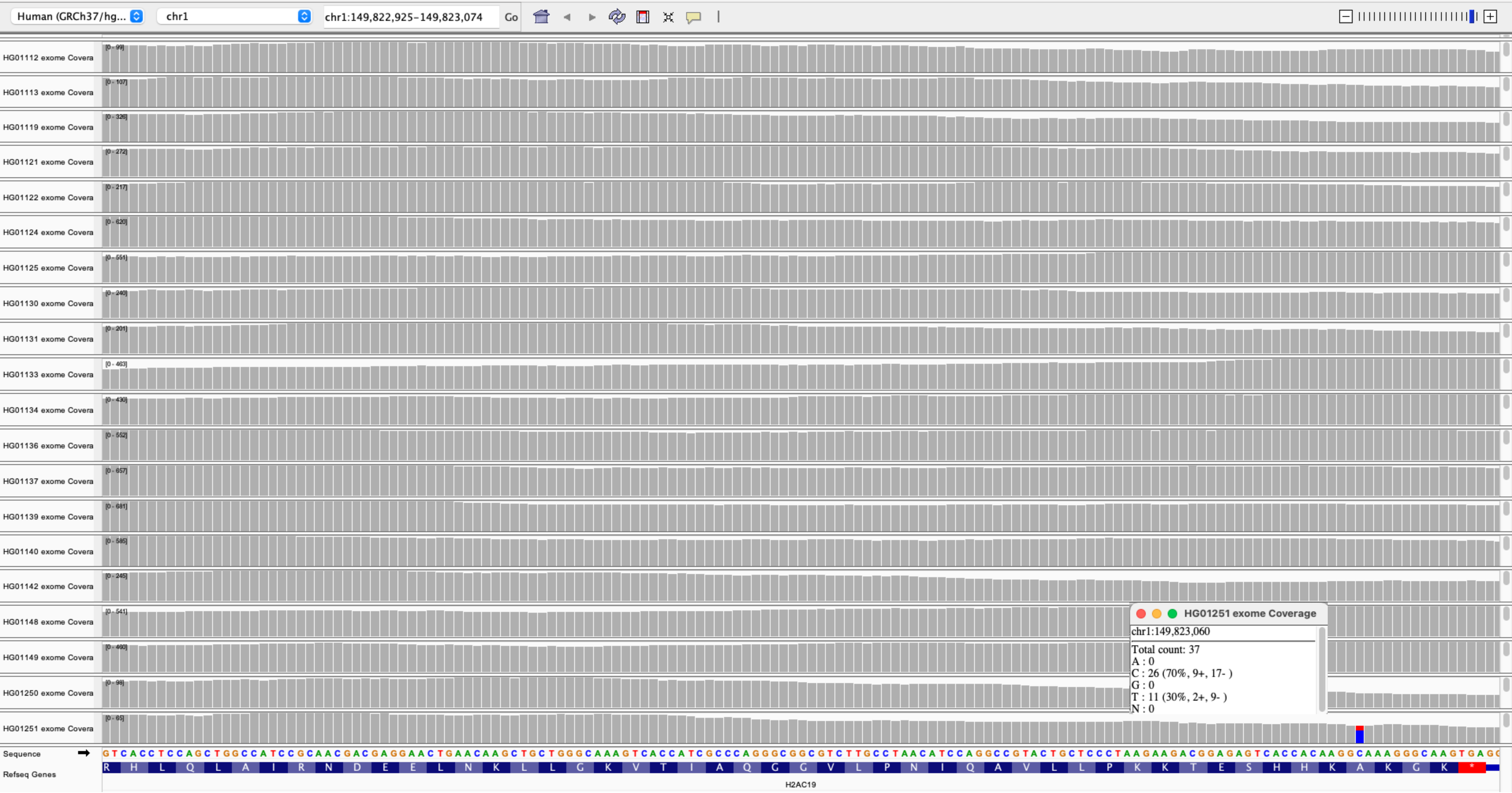

CLM: HG01253-HG01350

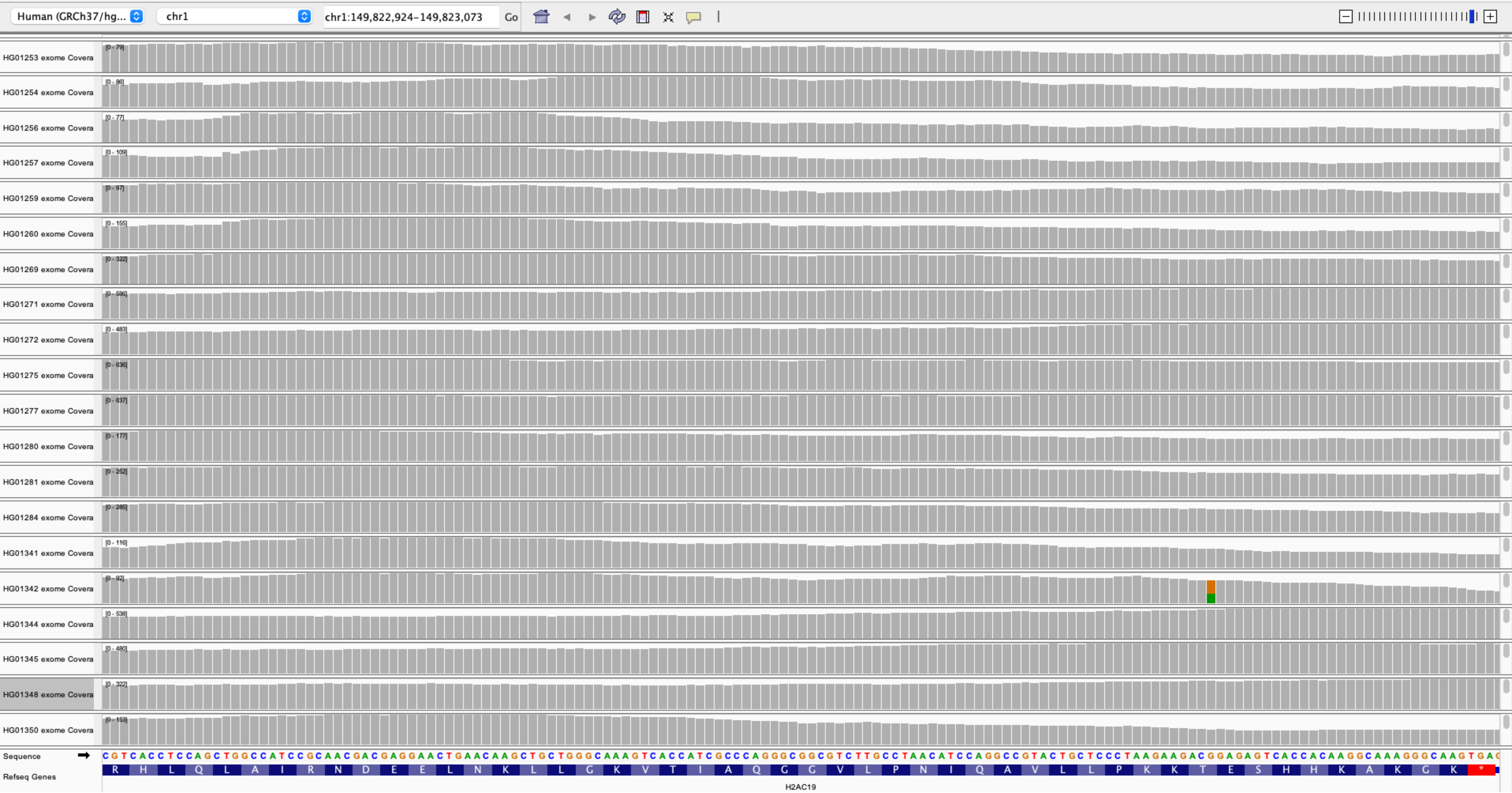

CLM: HG01351-HG01389

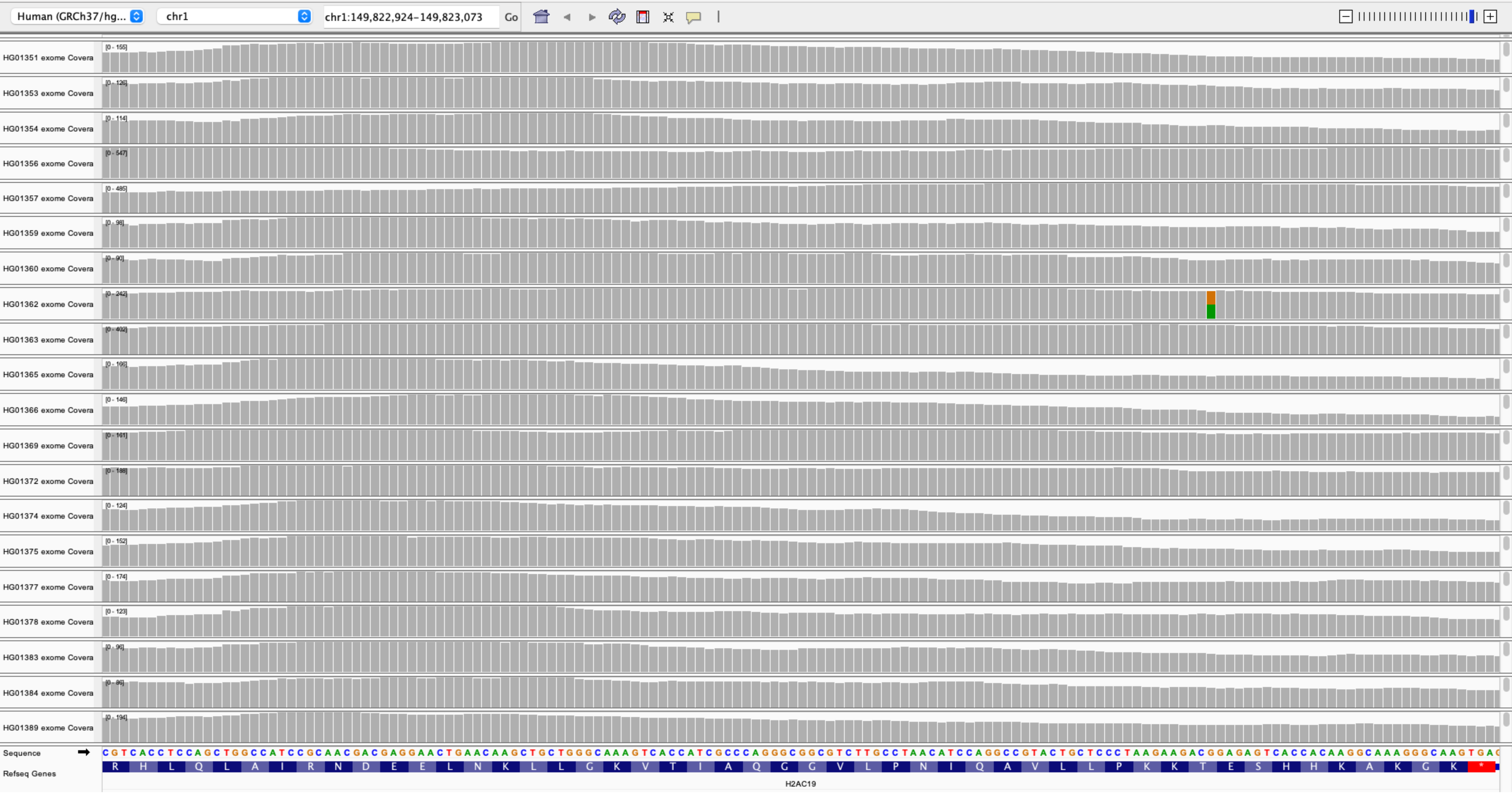

CLM: HG01390-HG01474

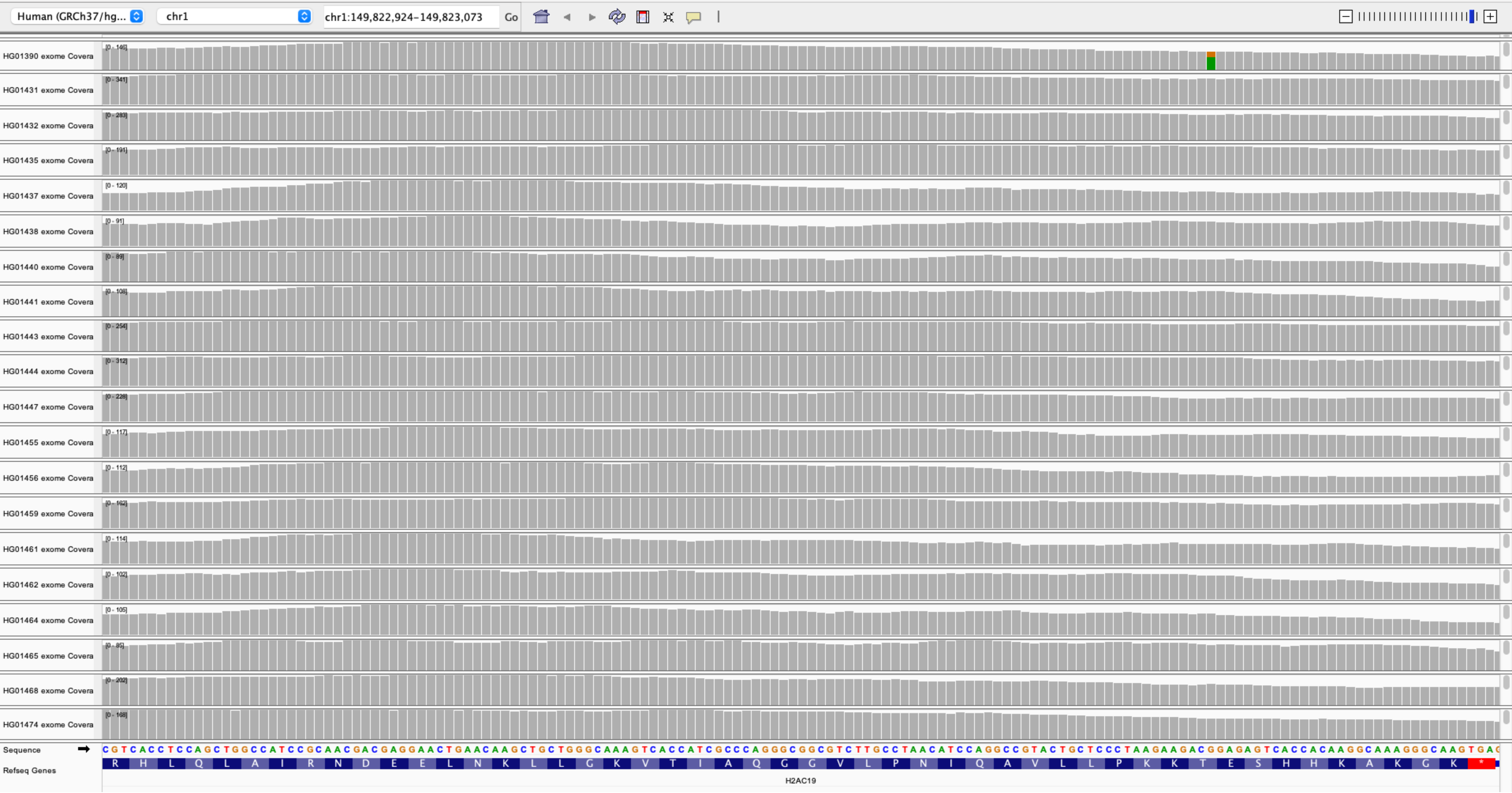

CLM: HG01479-HG01556

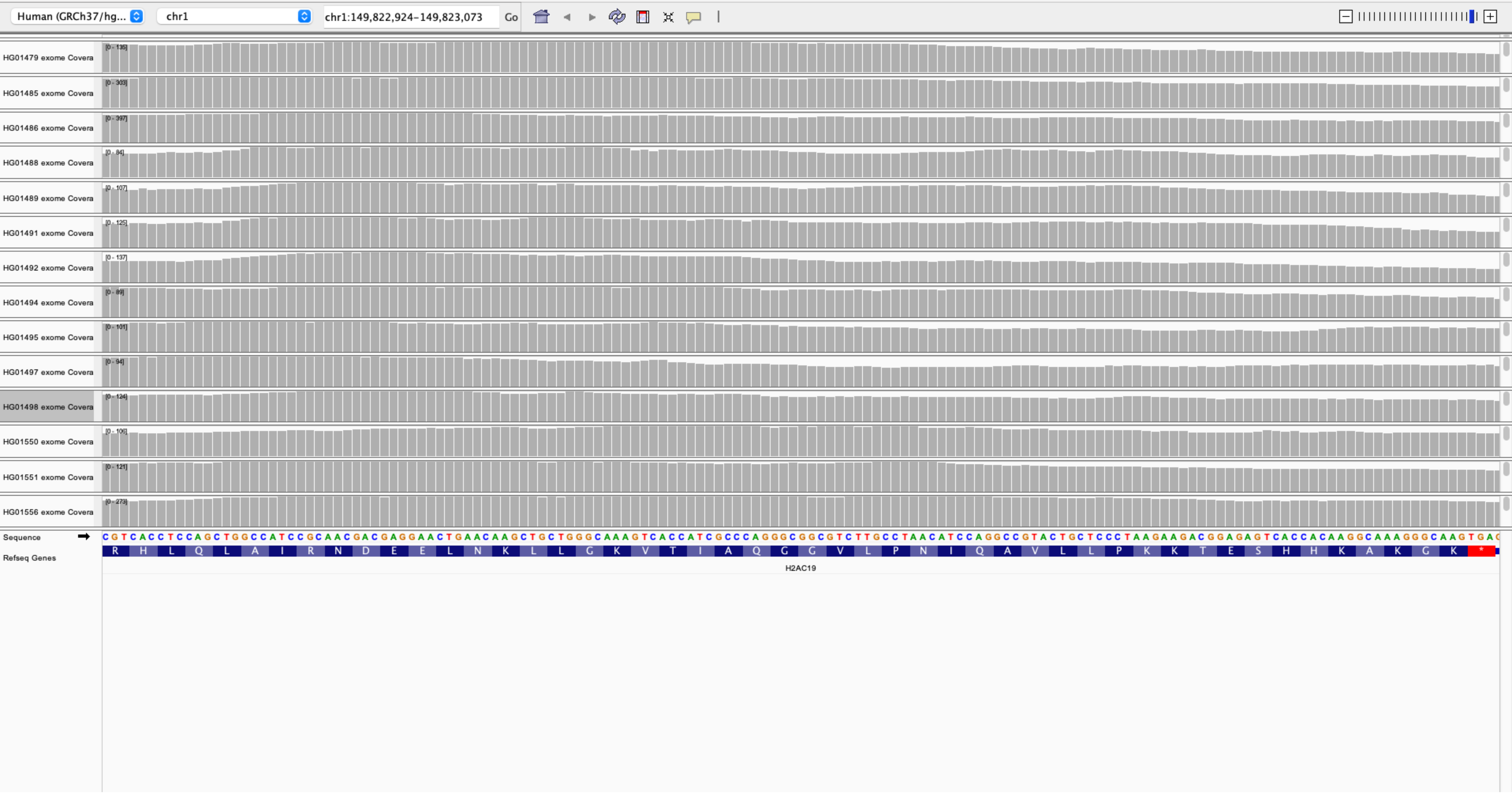

MXL: People with Mexican Ancestry in Los Angeles (67 samples)

MXL: NA19648-NA19682

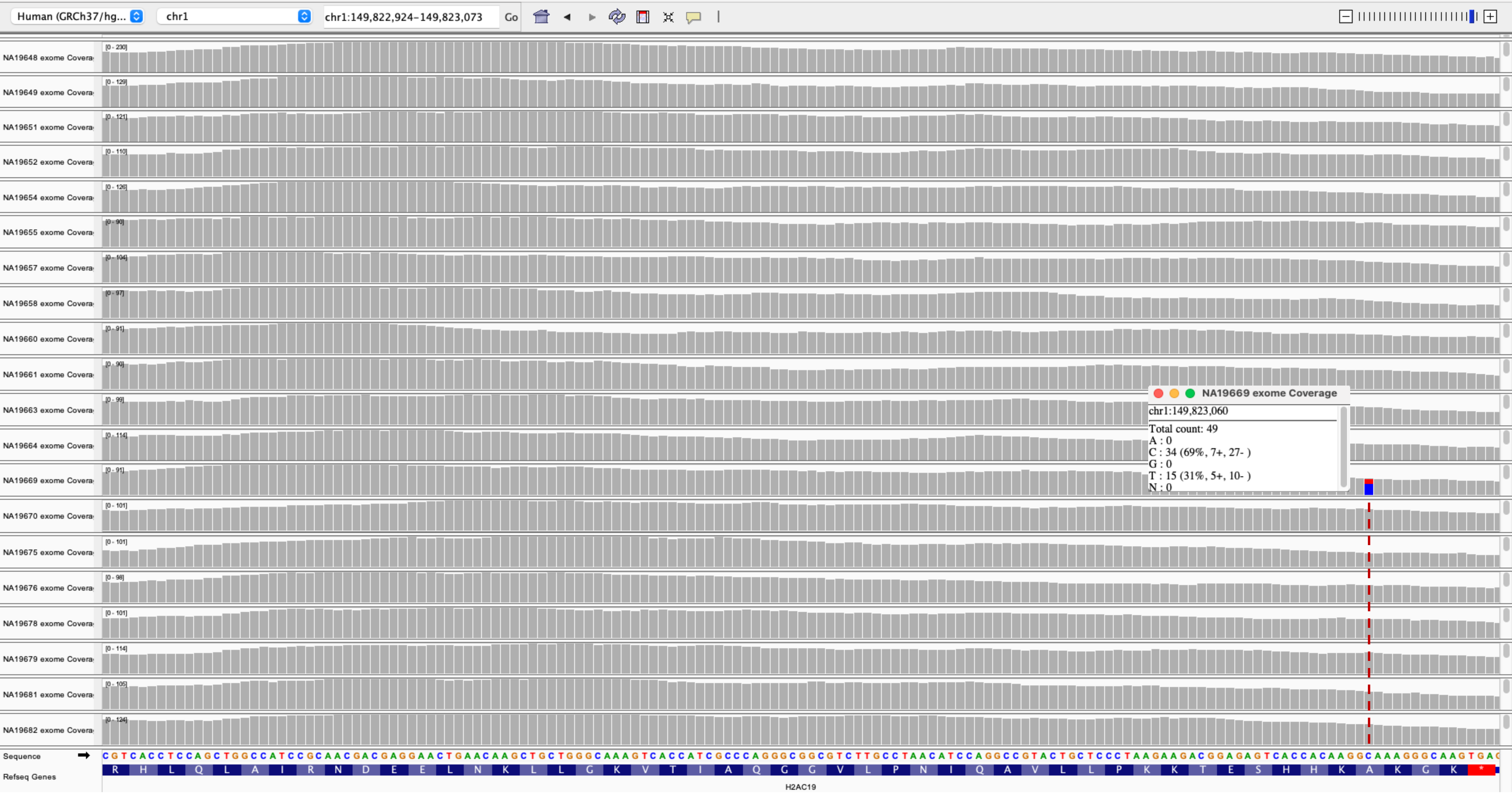

MXL: NA19684-NA19747

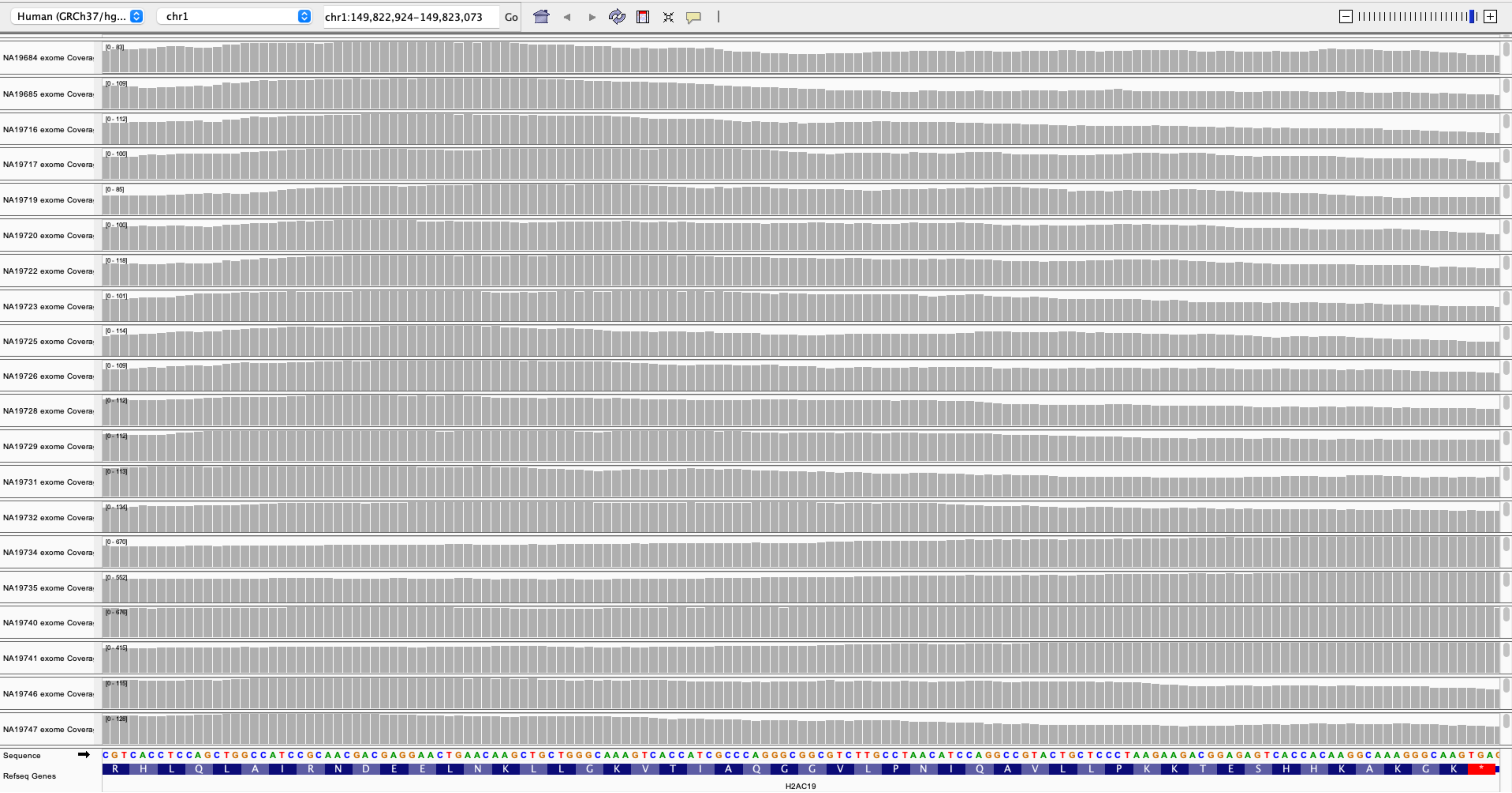

MXL: NA19749-NA19783

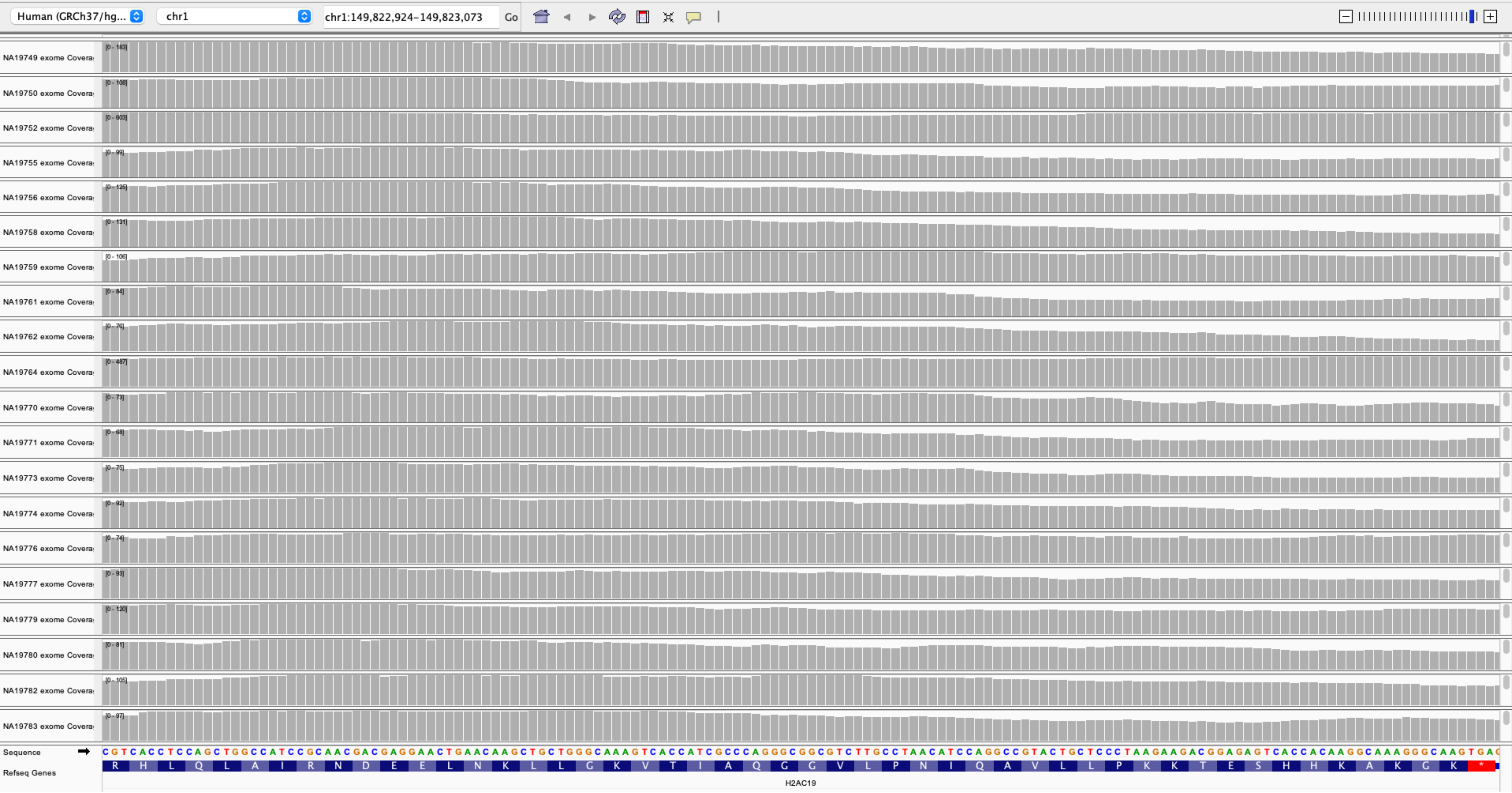

MXL: NA19785-NA19795

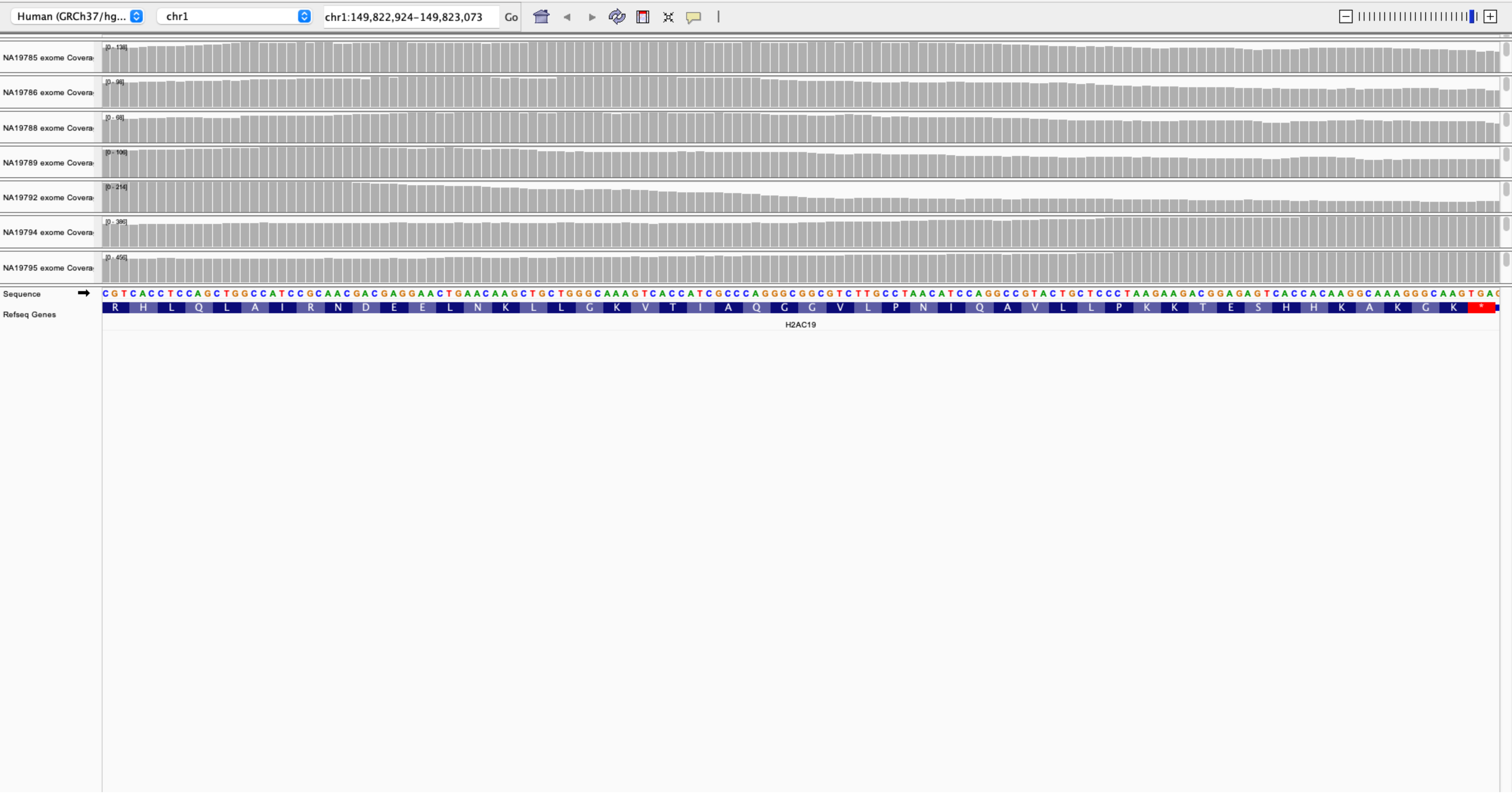

PEL: Peruvians in Lima, Peru (86 samples)

PEL: HG01565-HG01936

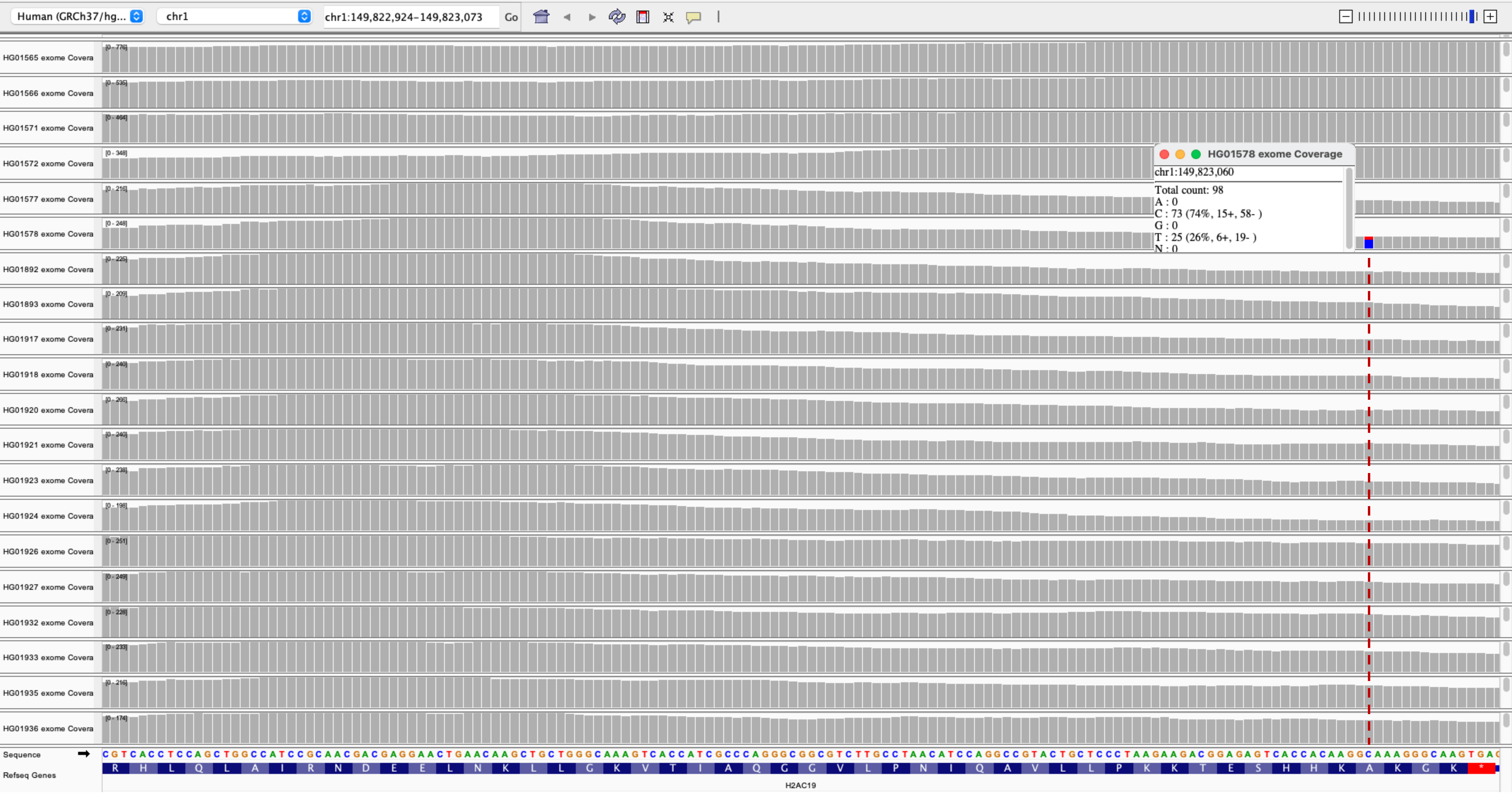

PEL: HG01938-HG01974

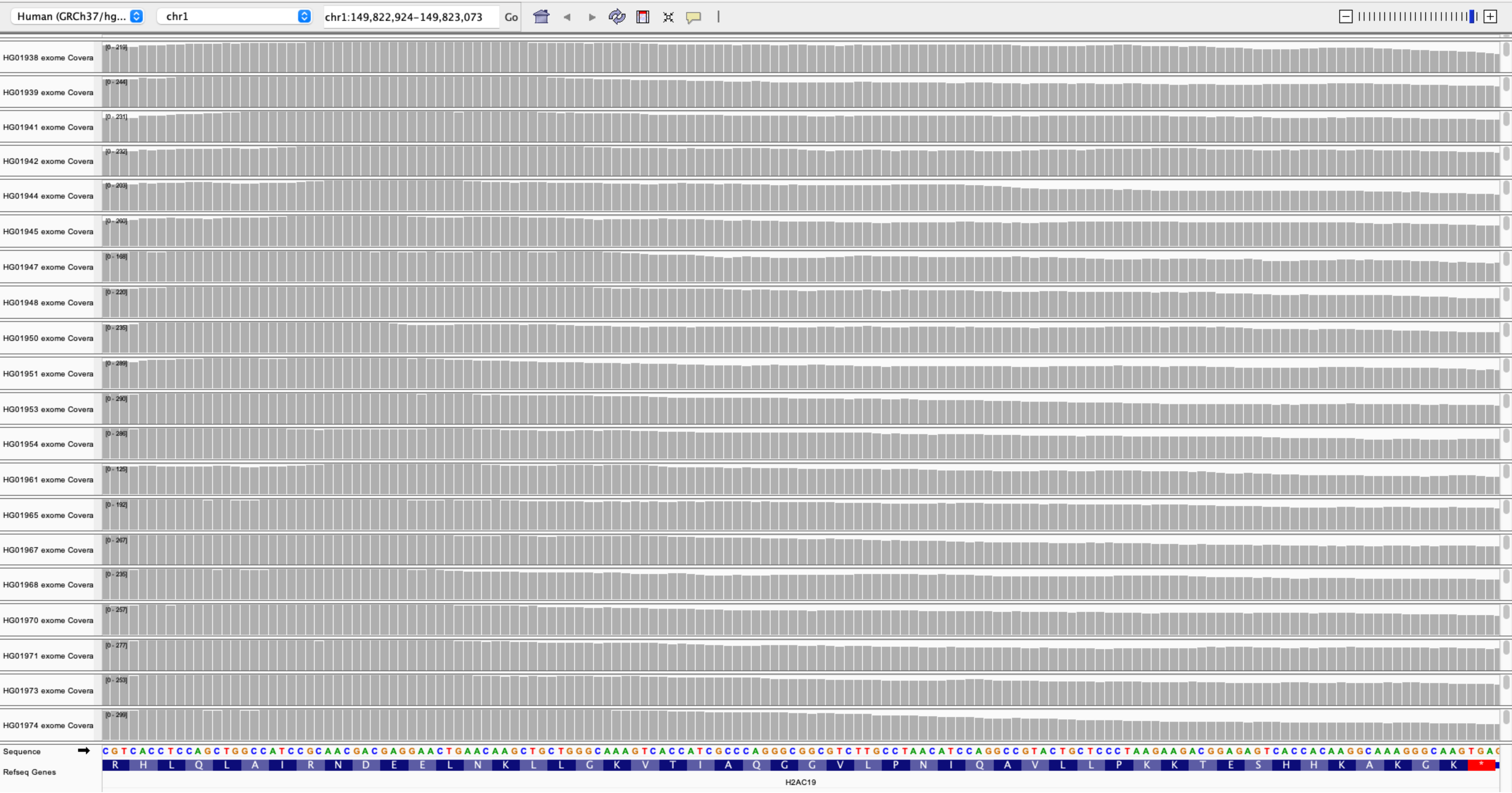

PEL: HG01976-HG02147

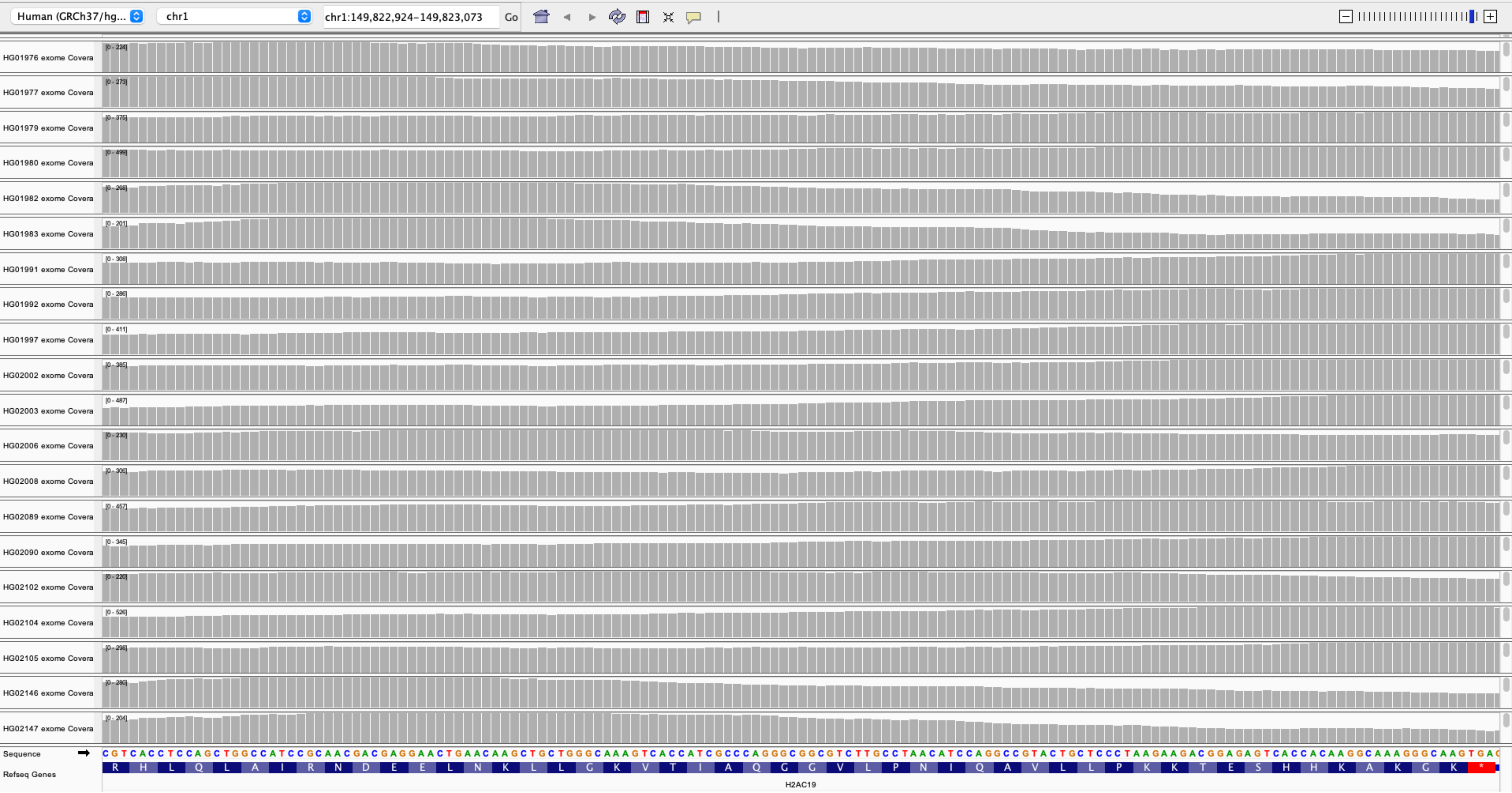

PEL: HG02150-HG02299

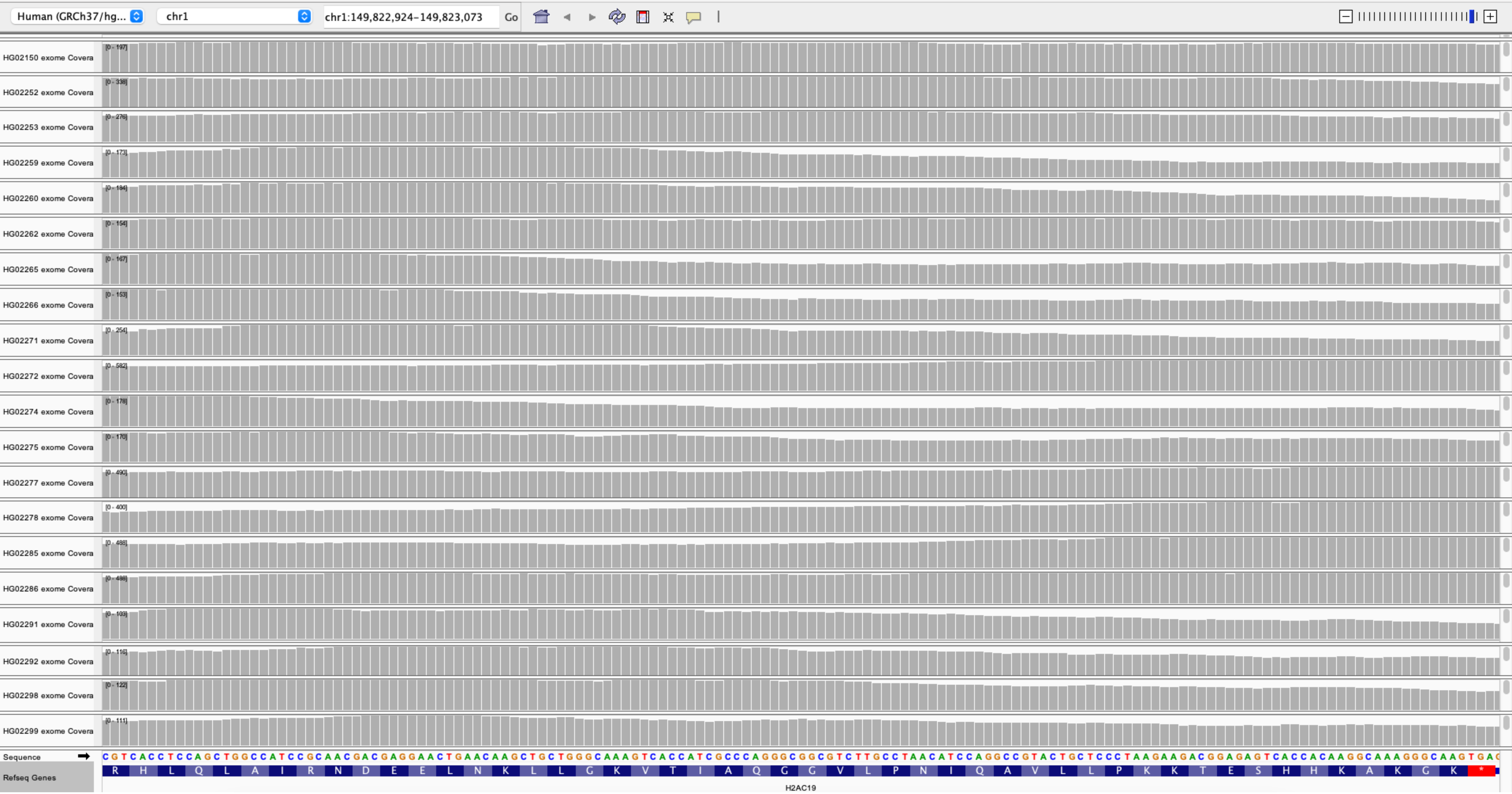

PEL: HG02301-HG02425

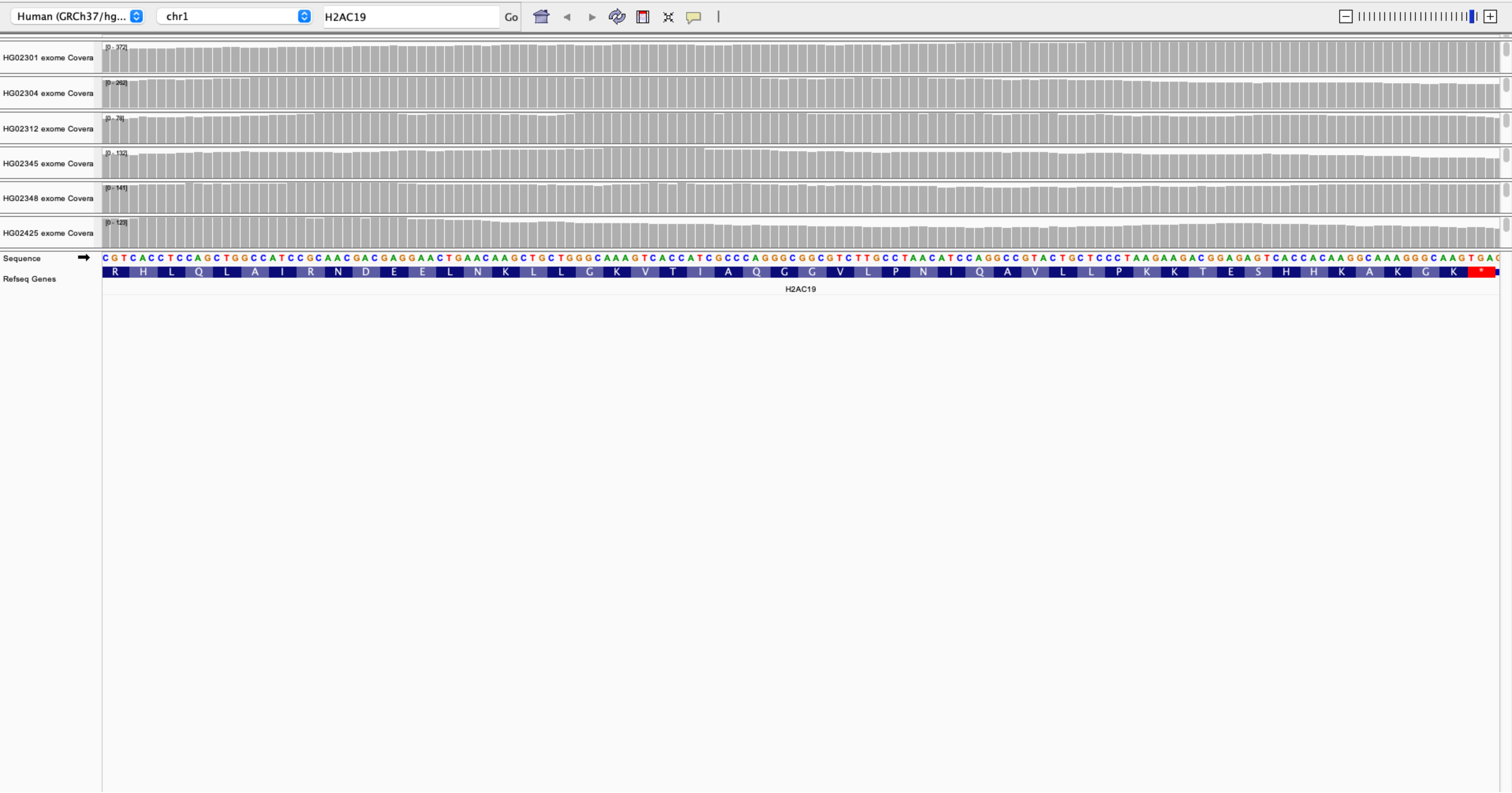

PUR:
(105 samples)

PUR: HG00551-HG01049

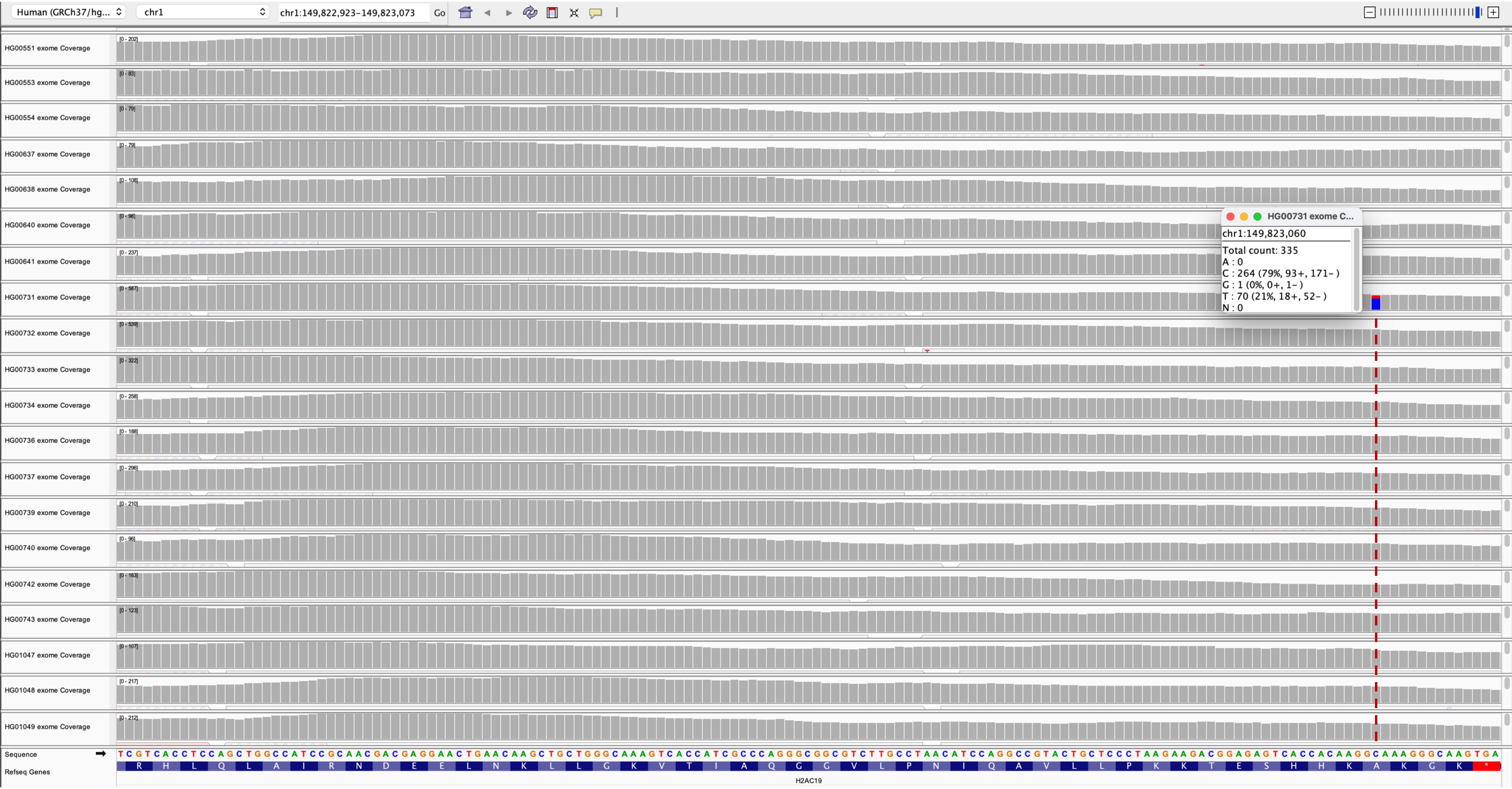

PUR: HG01051-HG01082

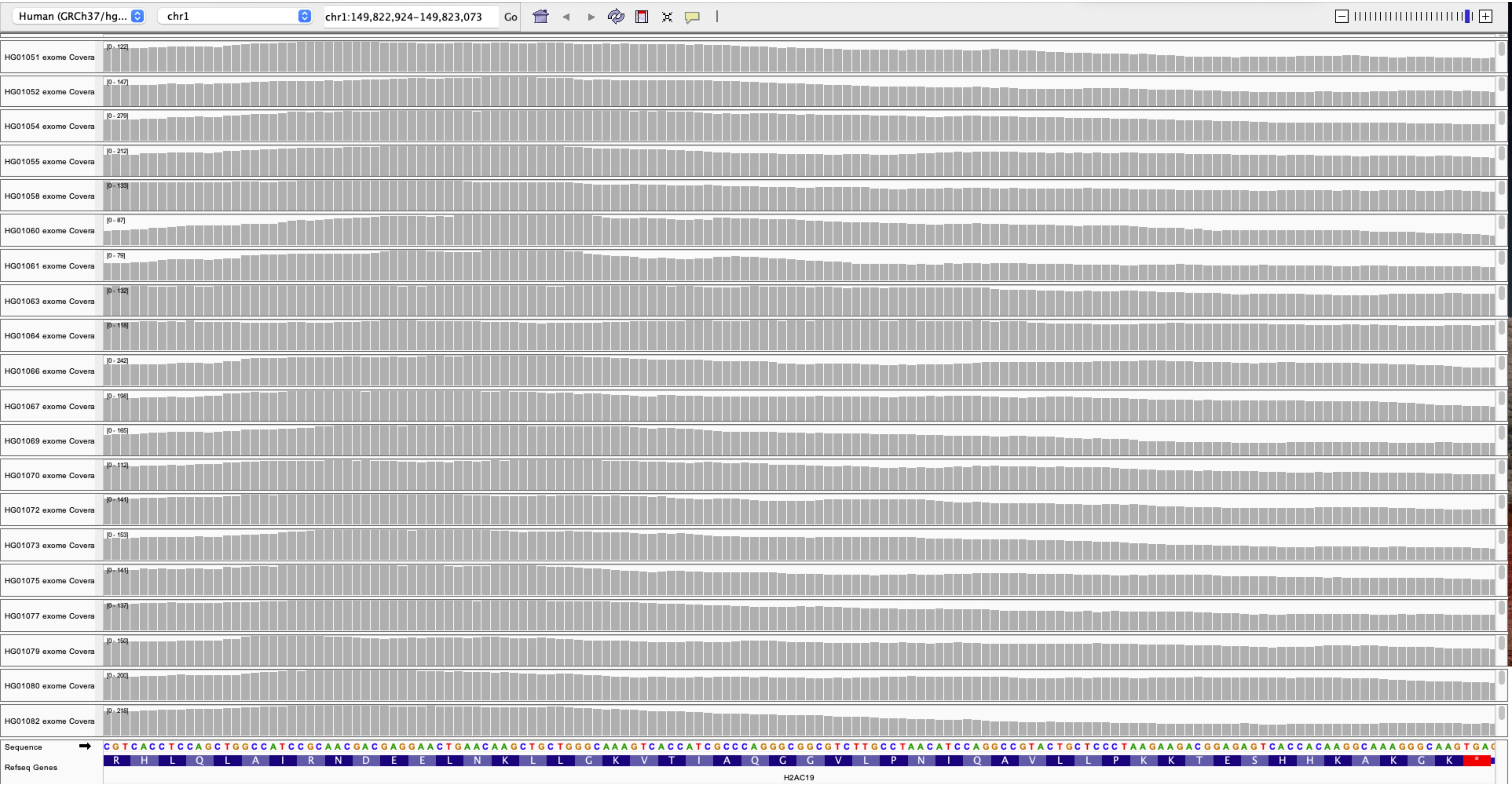

PUR: HG01083-HG01162

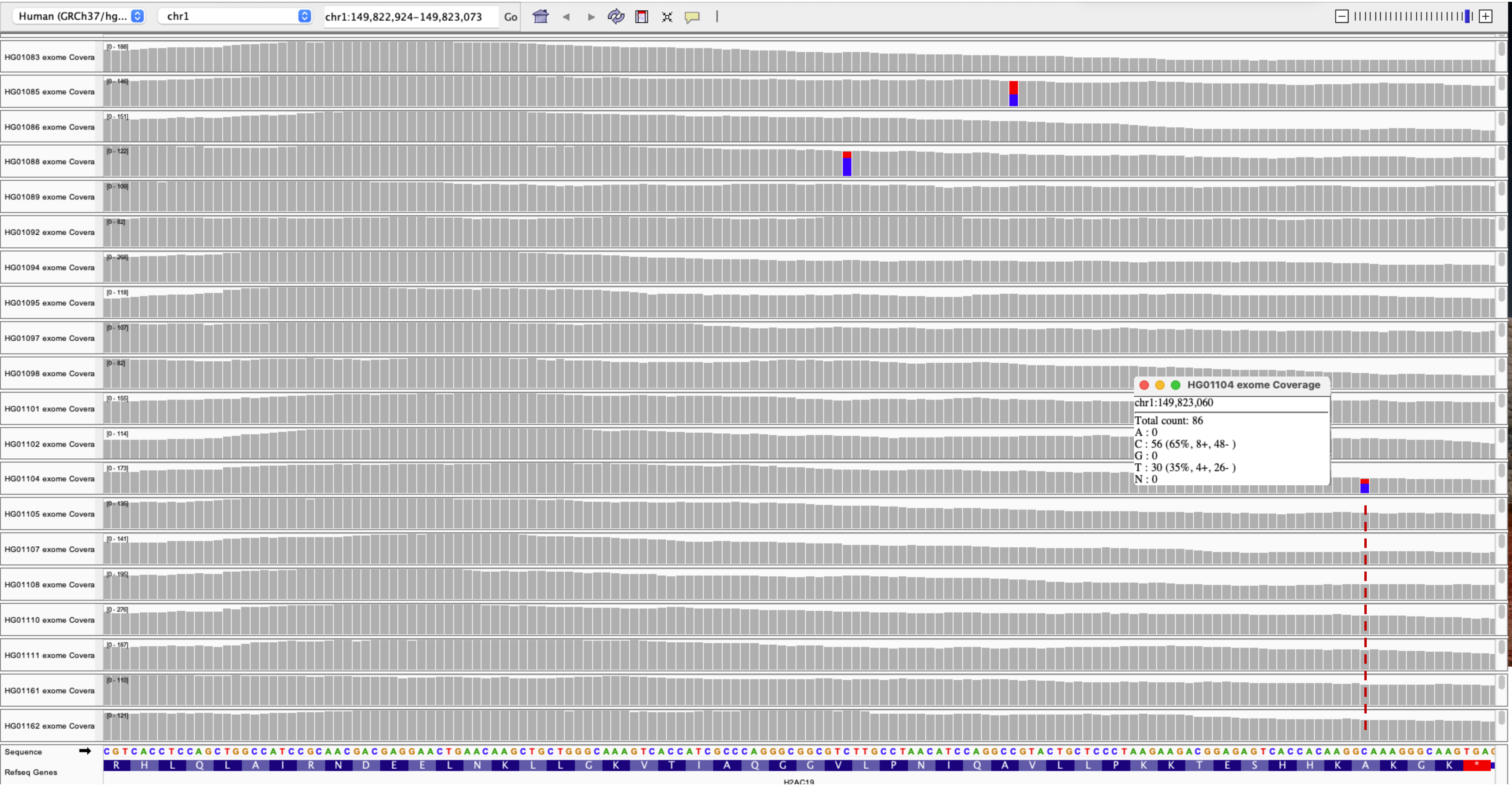

PUR: HG01164-HG01205

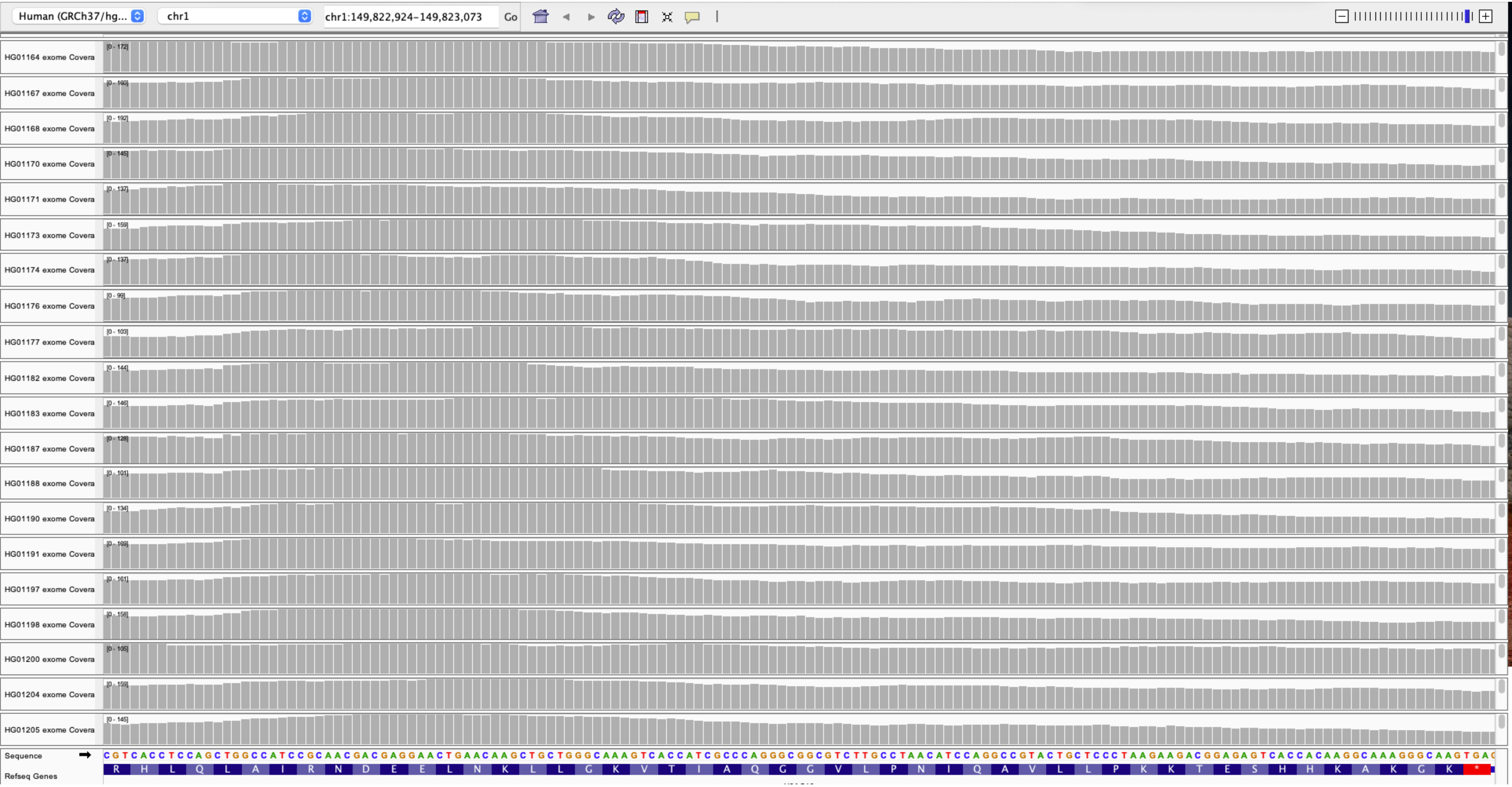

PUR: HG01241-HG01402

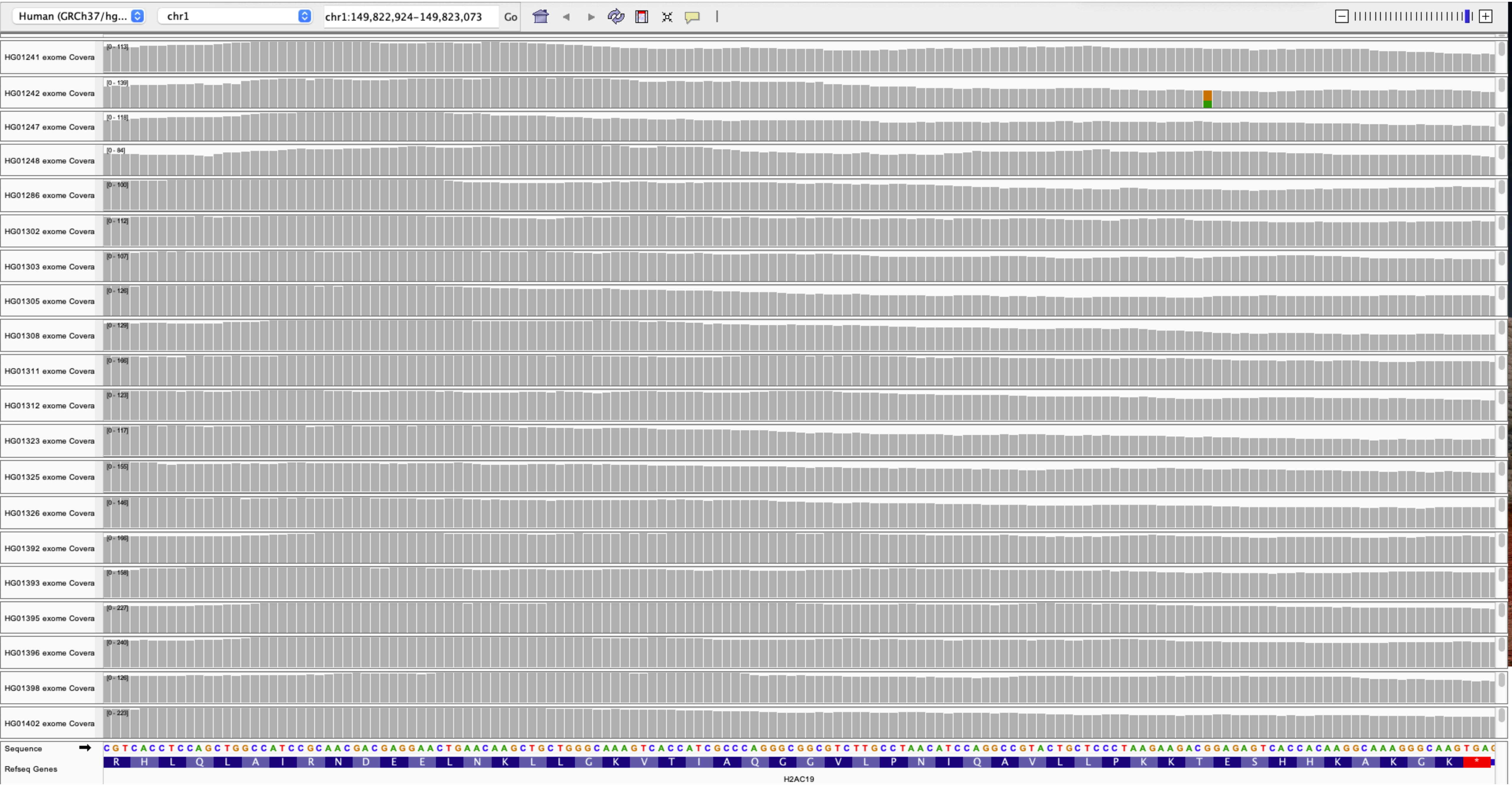

PUR: HG01403-HG01414

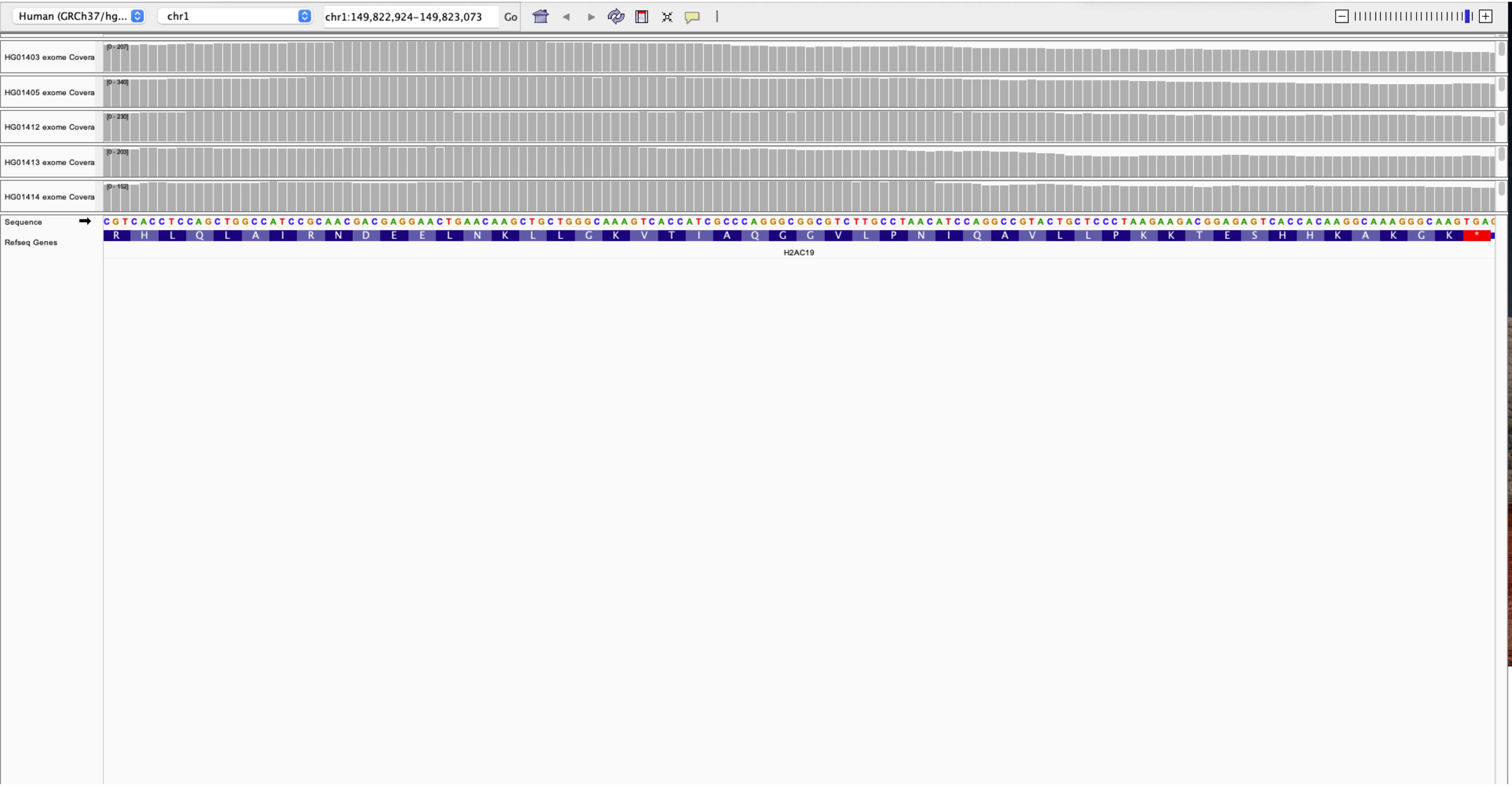

CHB:
(103 samples)

CHB: NA18525-NA18547

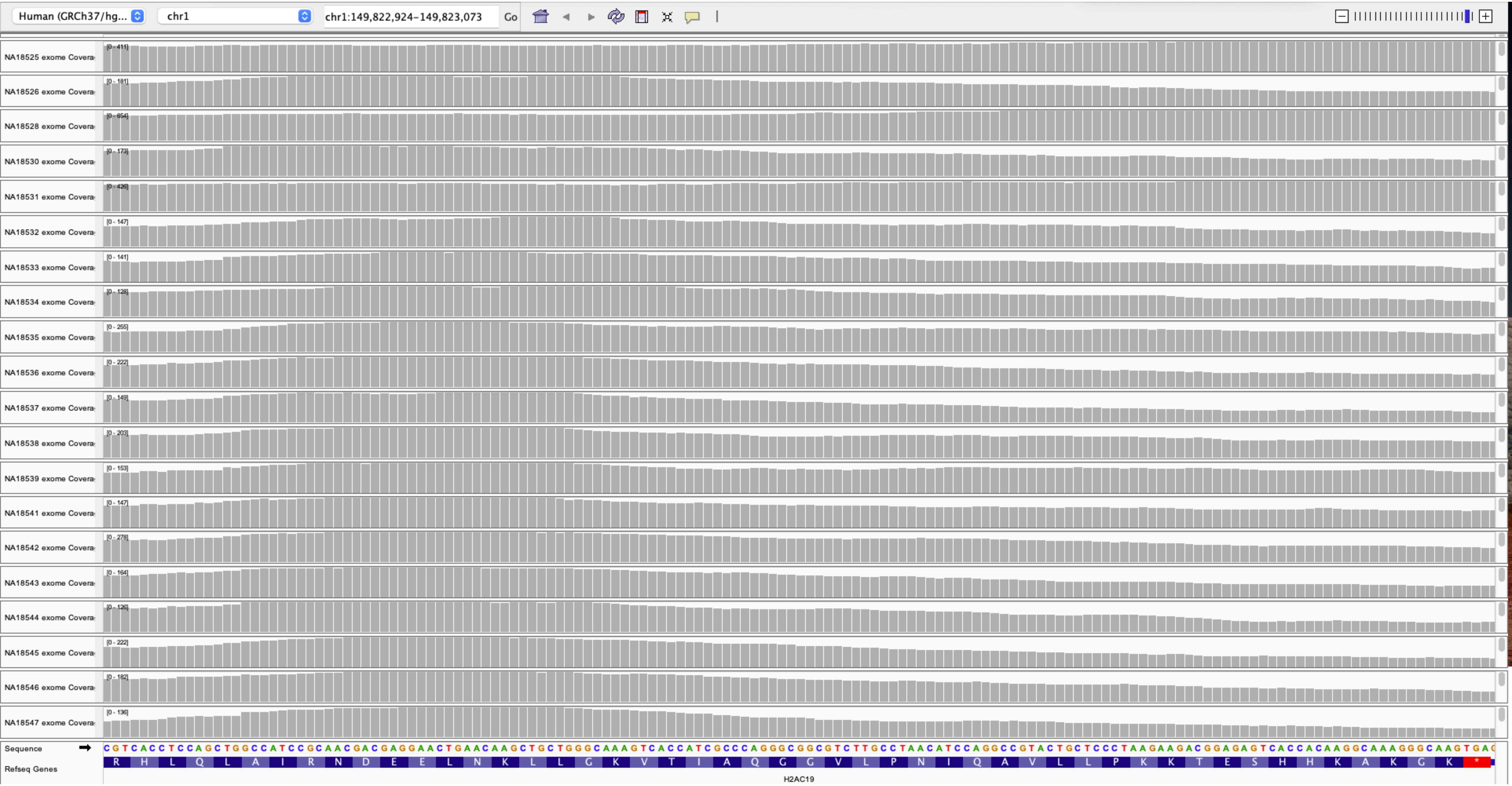

CHB: NA18548-NA18572

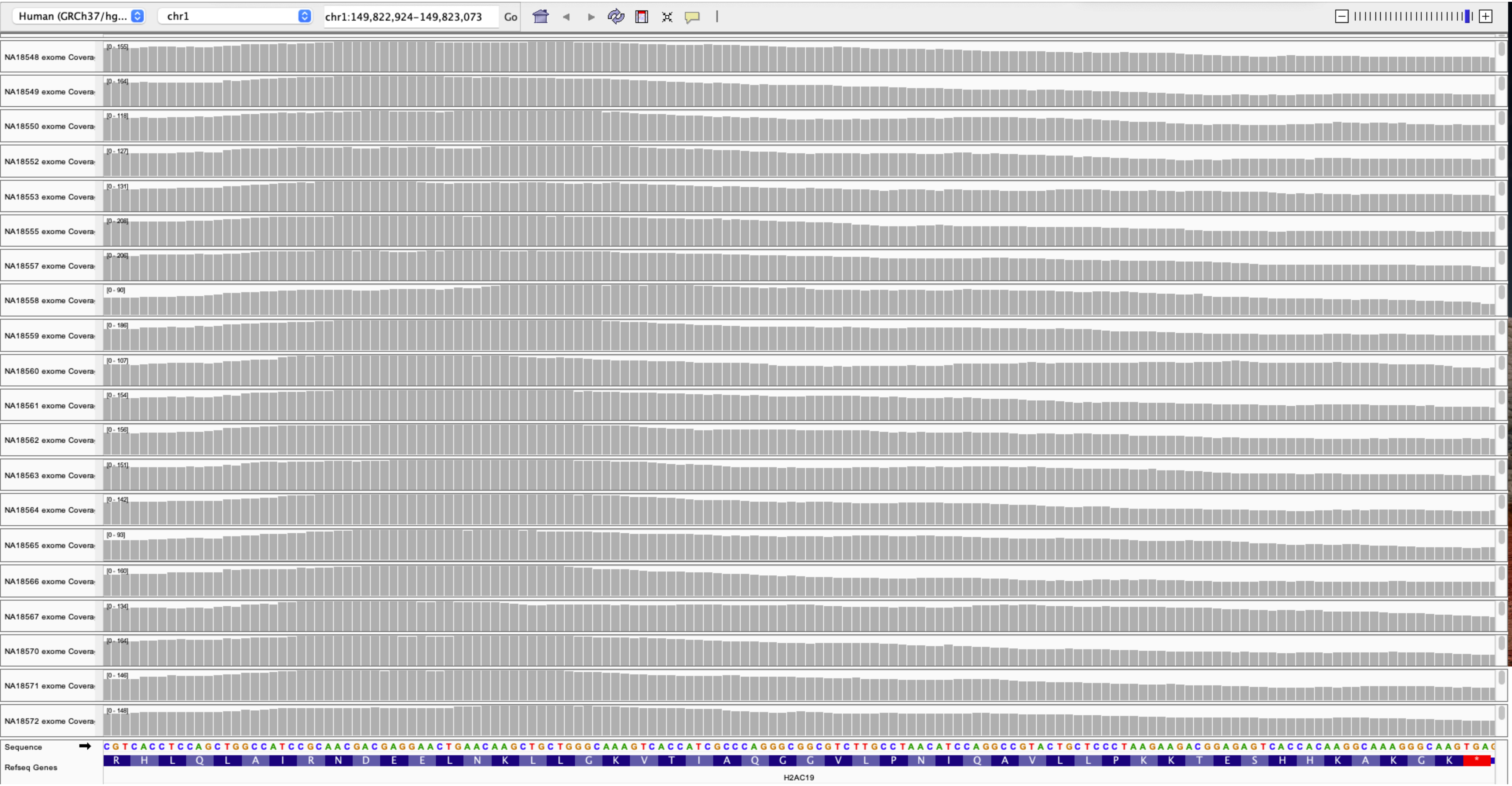

CHB: NA18573-NA18611

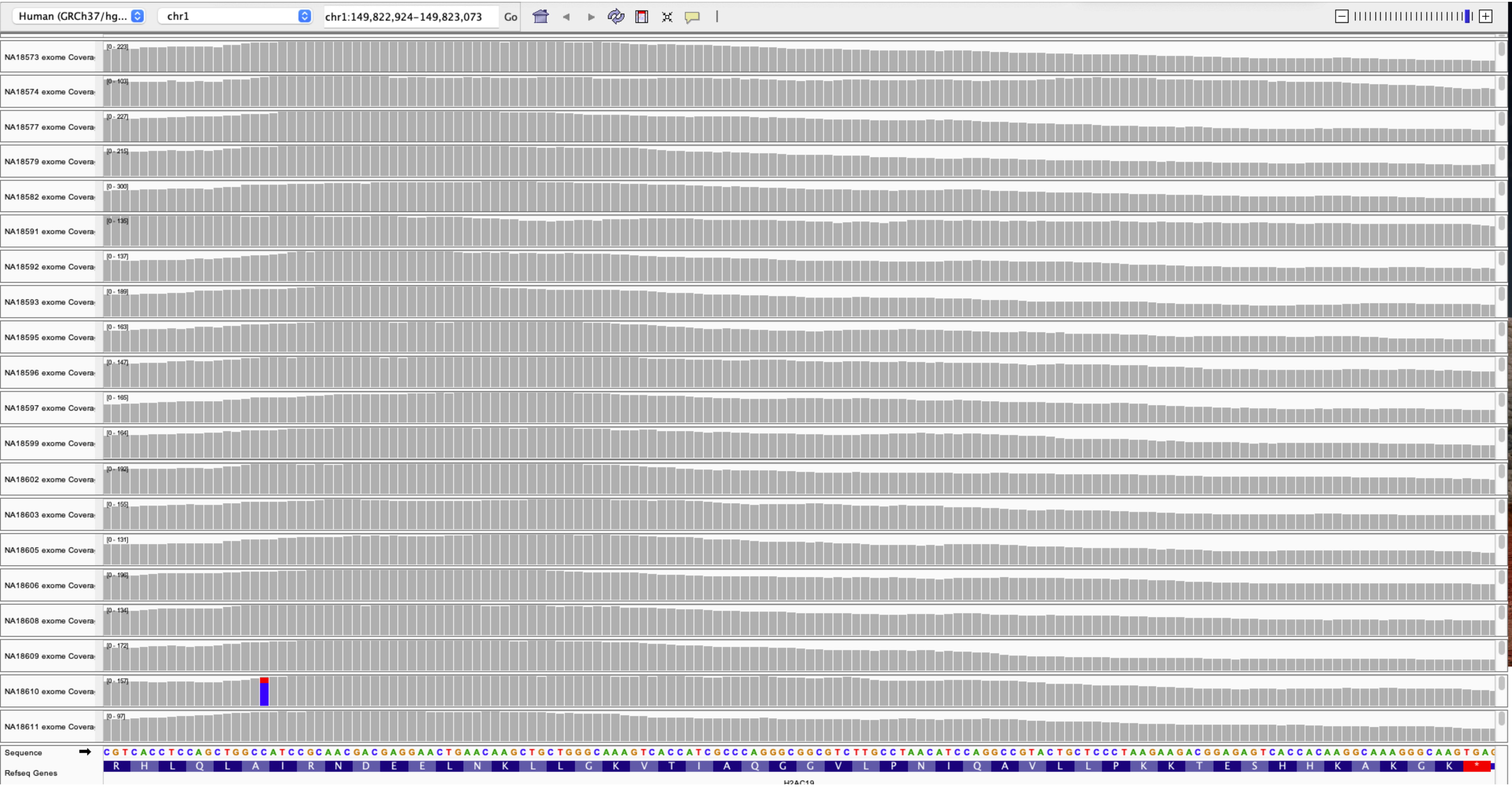

CHB: NA18612-NA18631

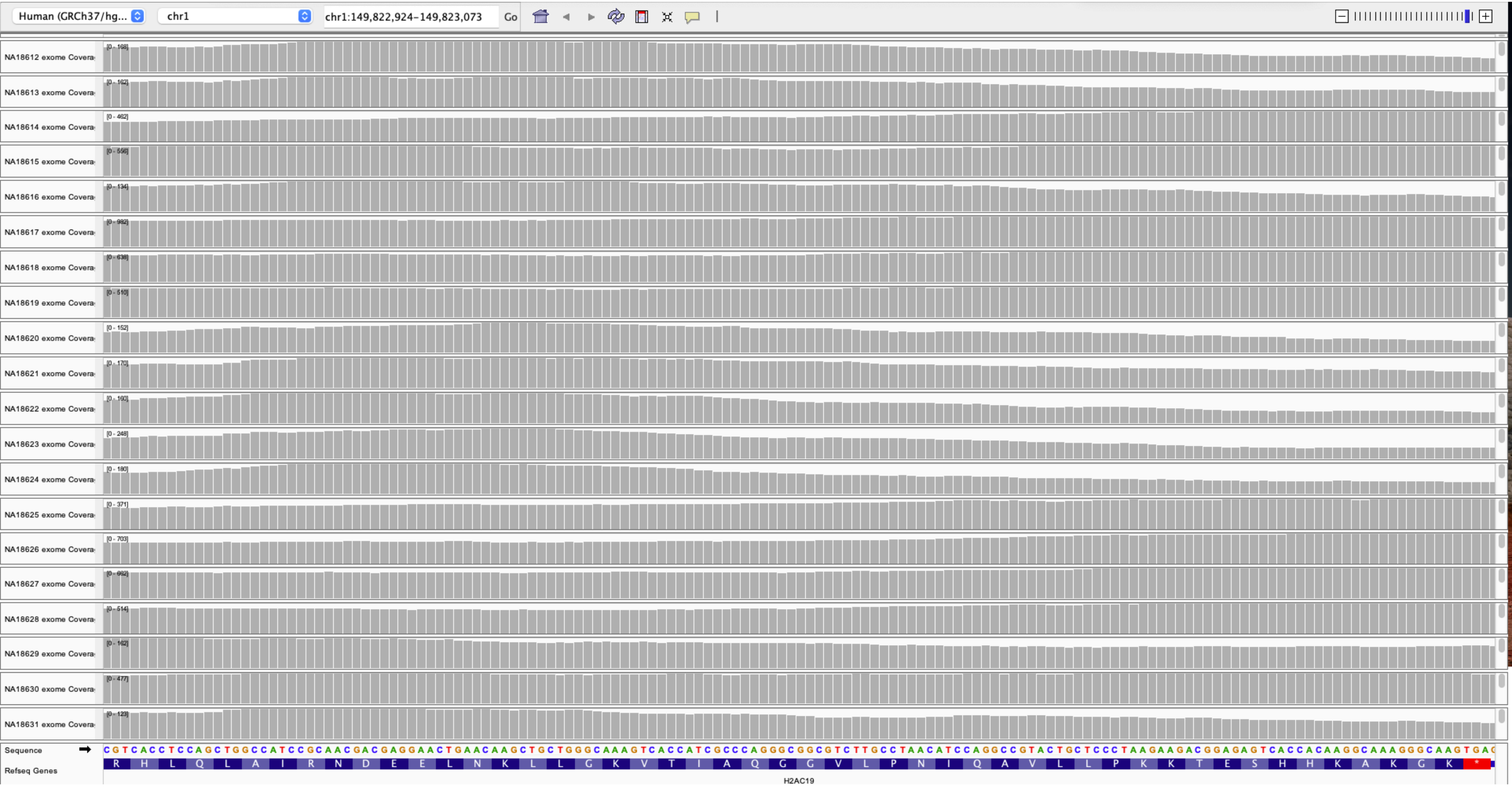

CHB: NA18632-NA18747

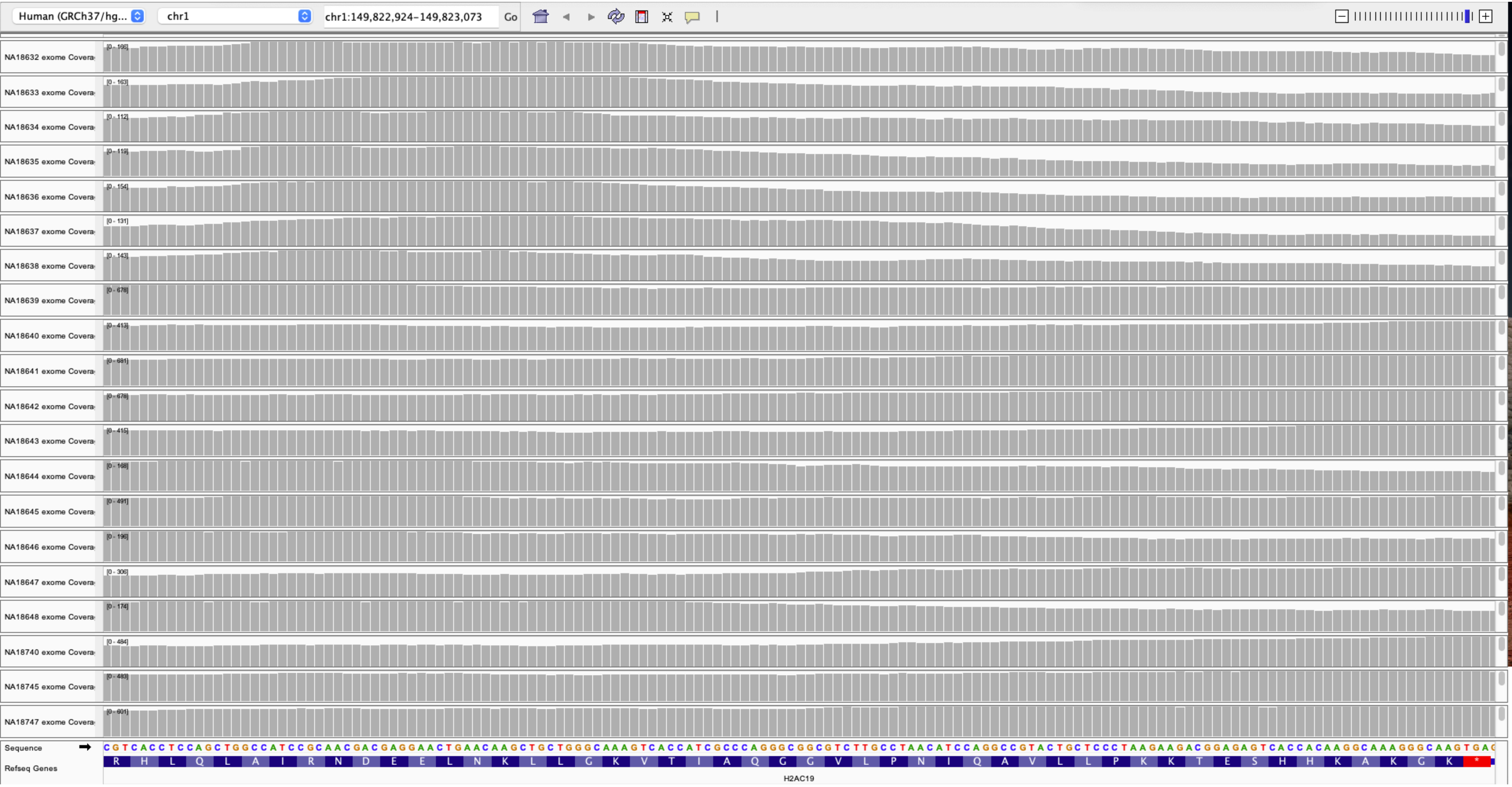

CHB: NA18748-NA18757

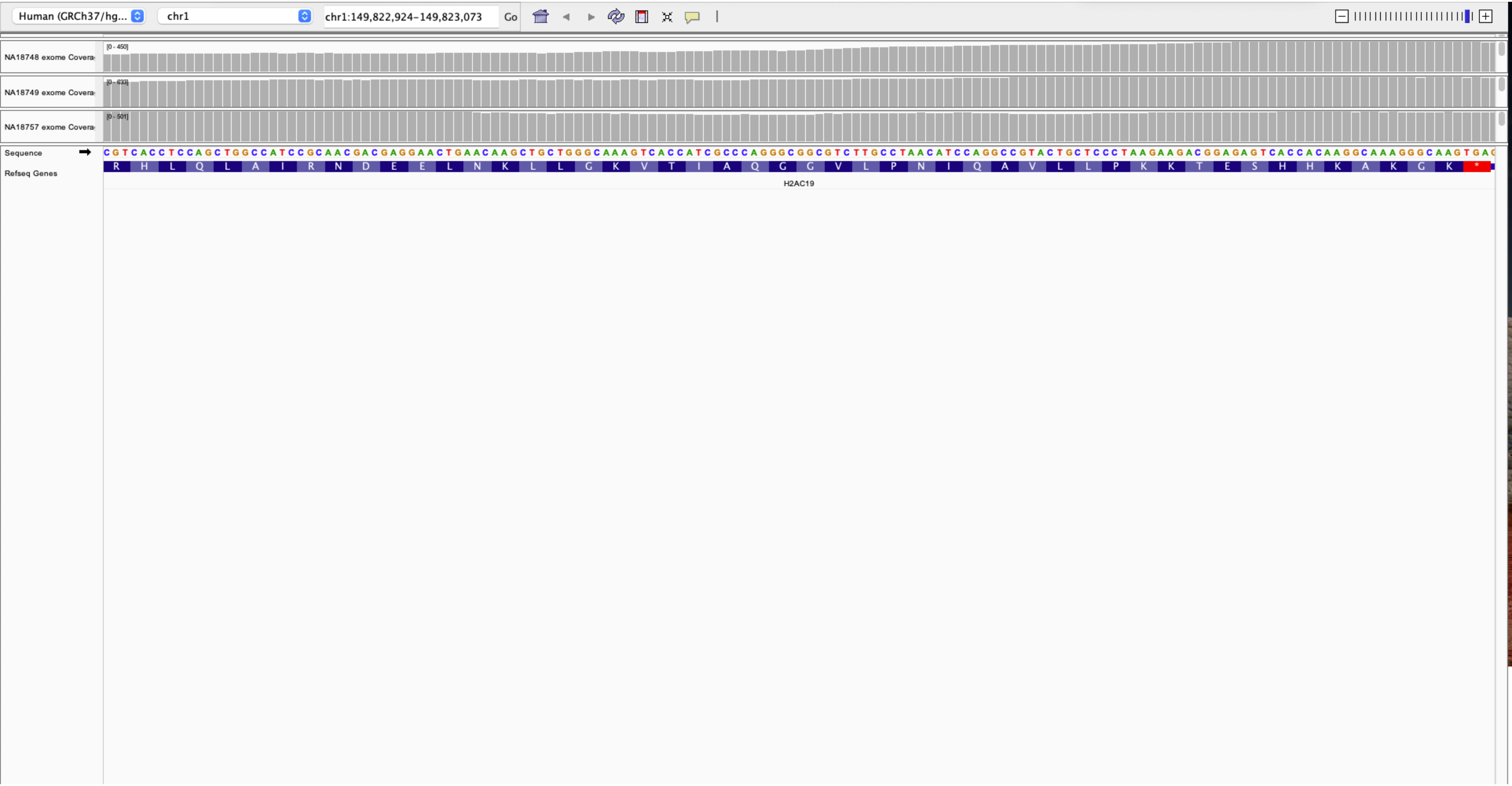

CHS:
(108 samples)

CHS: HG00403-HG00452

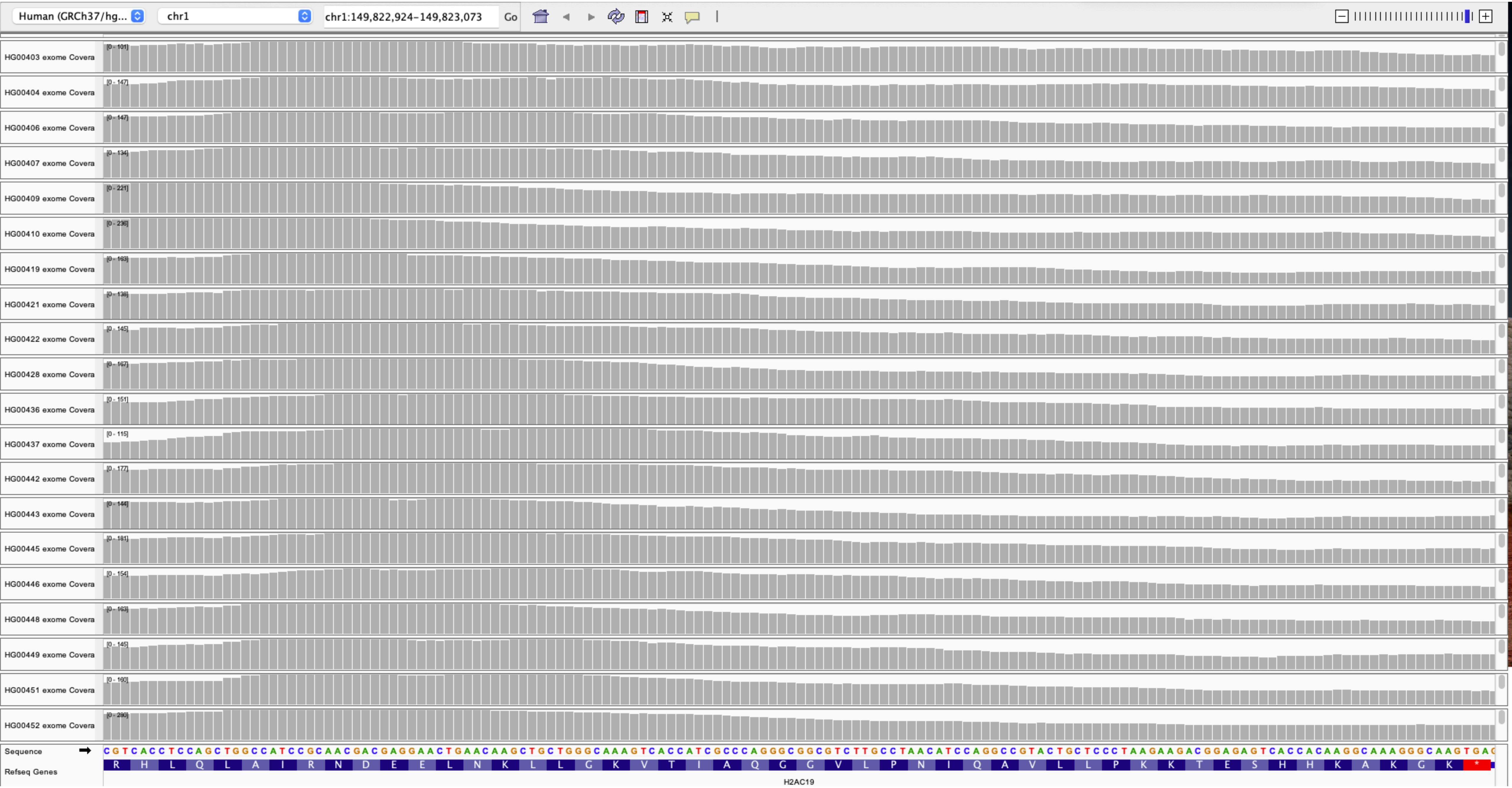

CHS: HG00457-HG00536

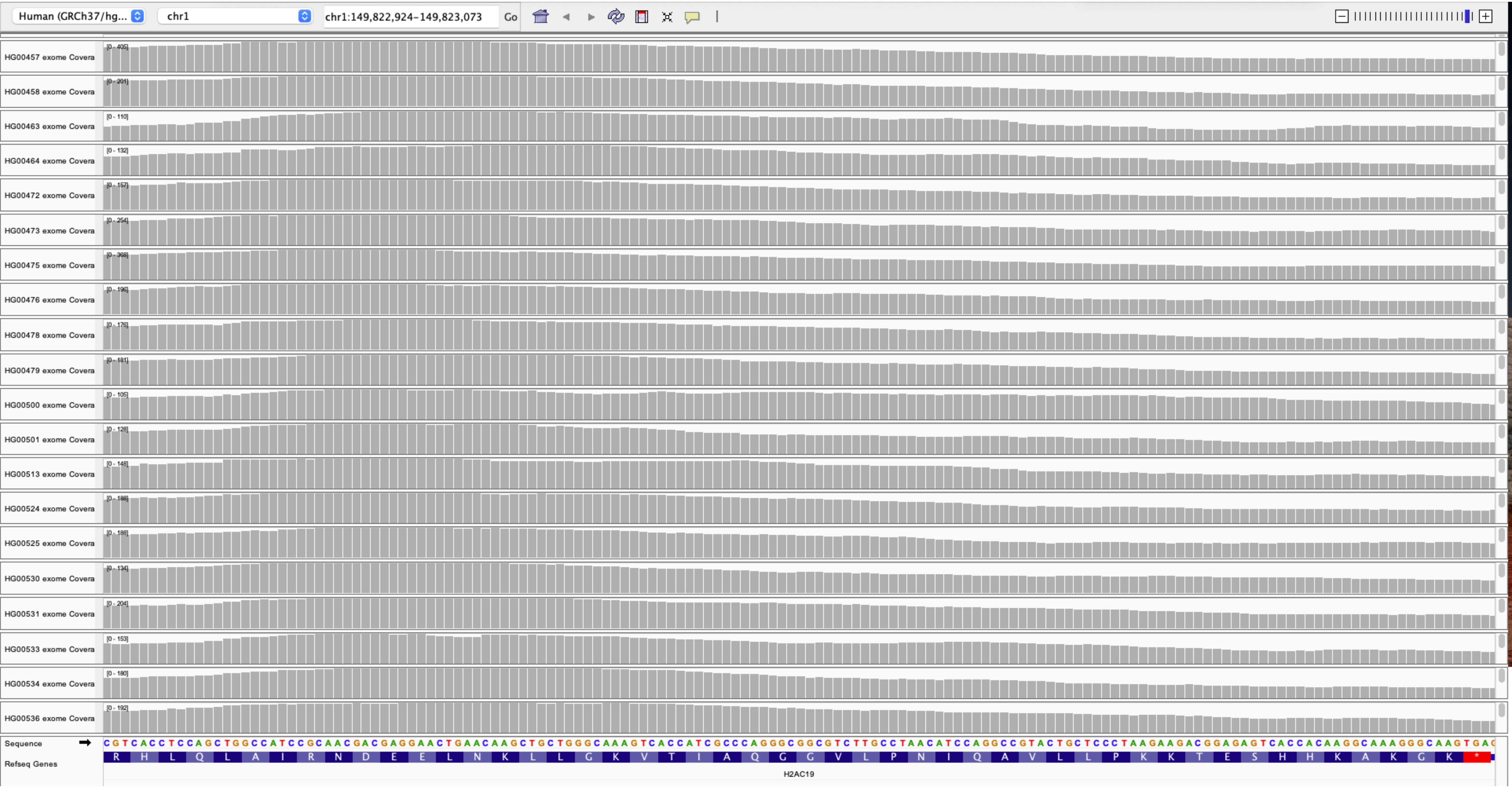

CHS: HG00537-HG00598

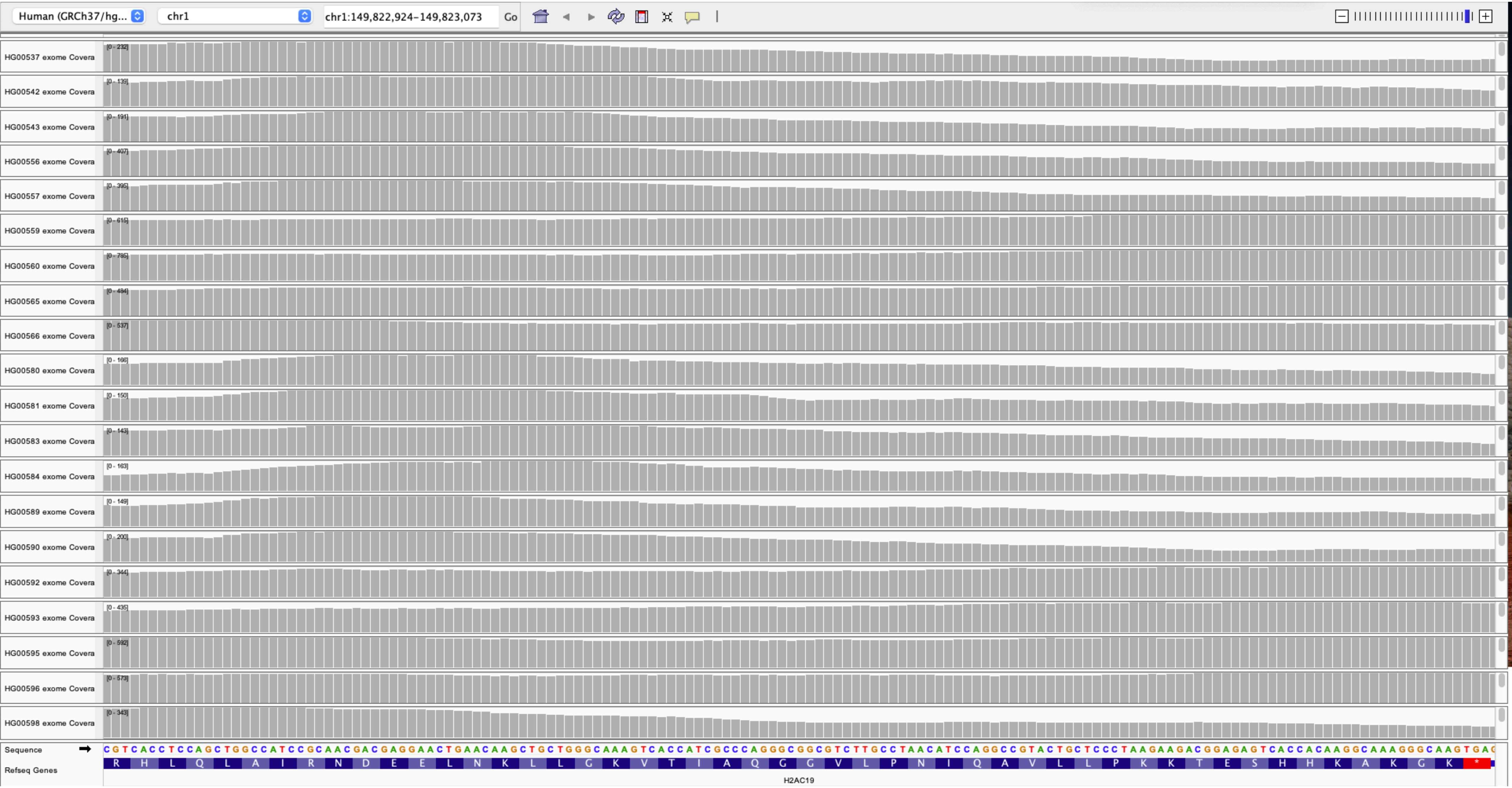

CHS: HG00599-HG00650

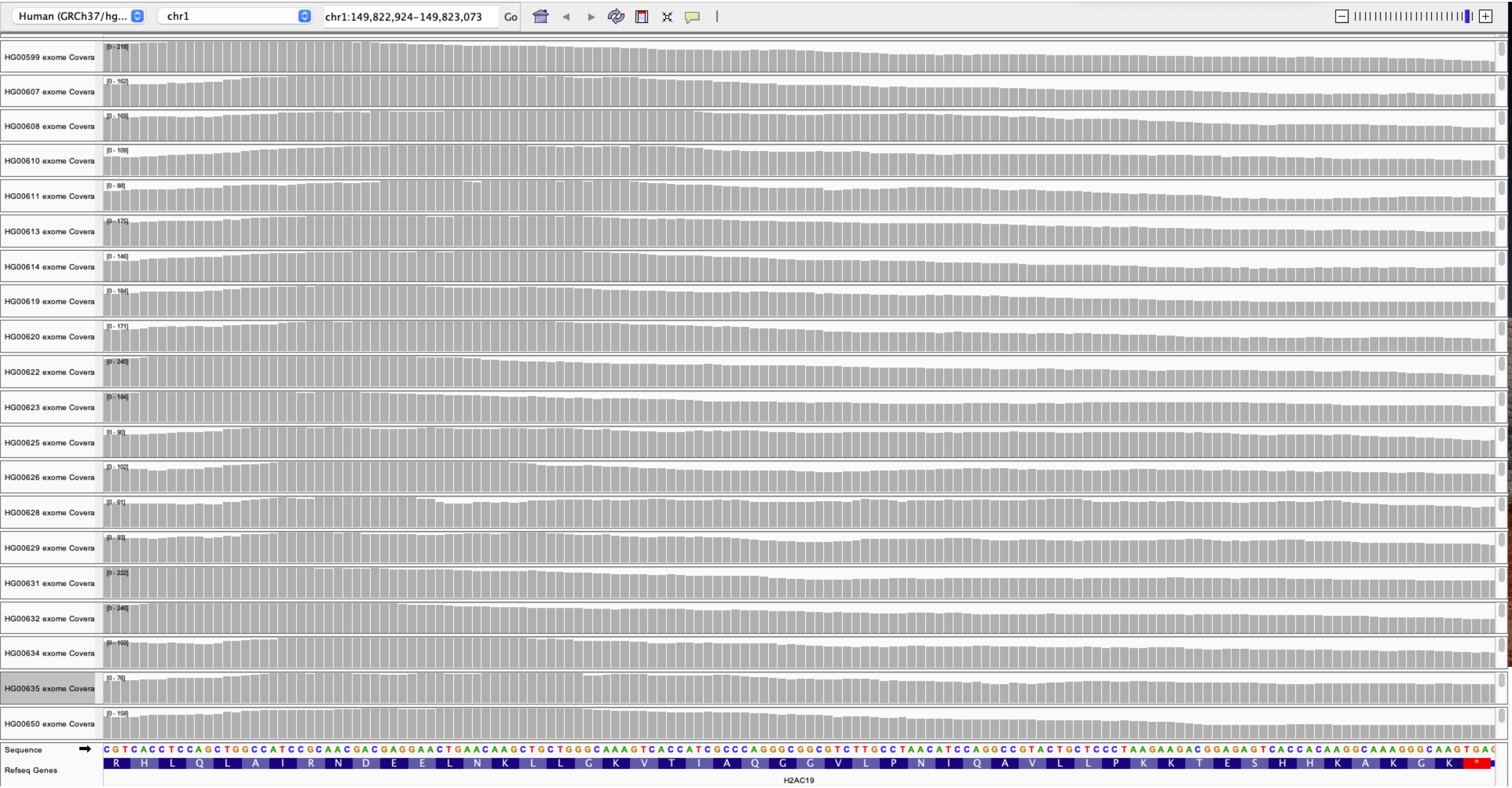

CHS: HG00651-HG00701

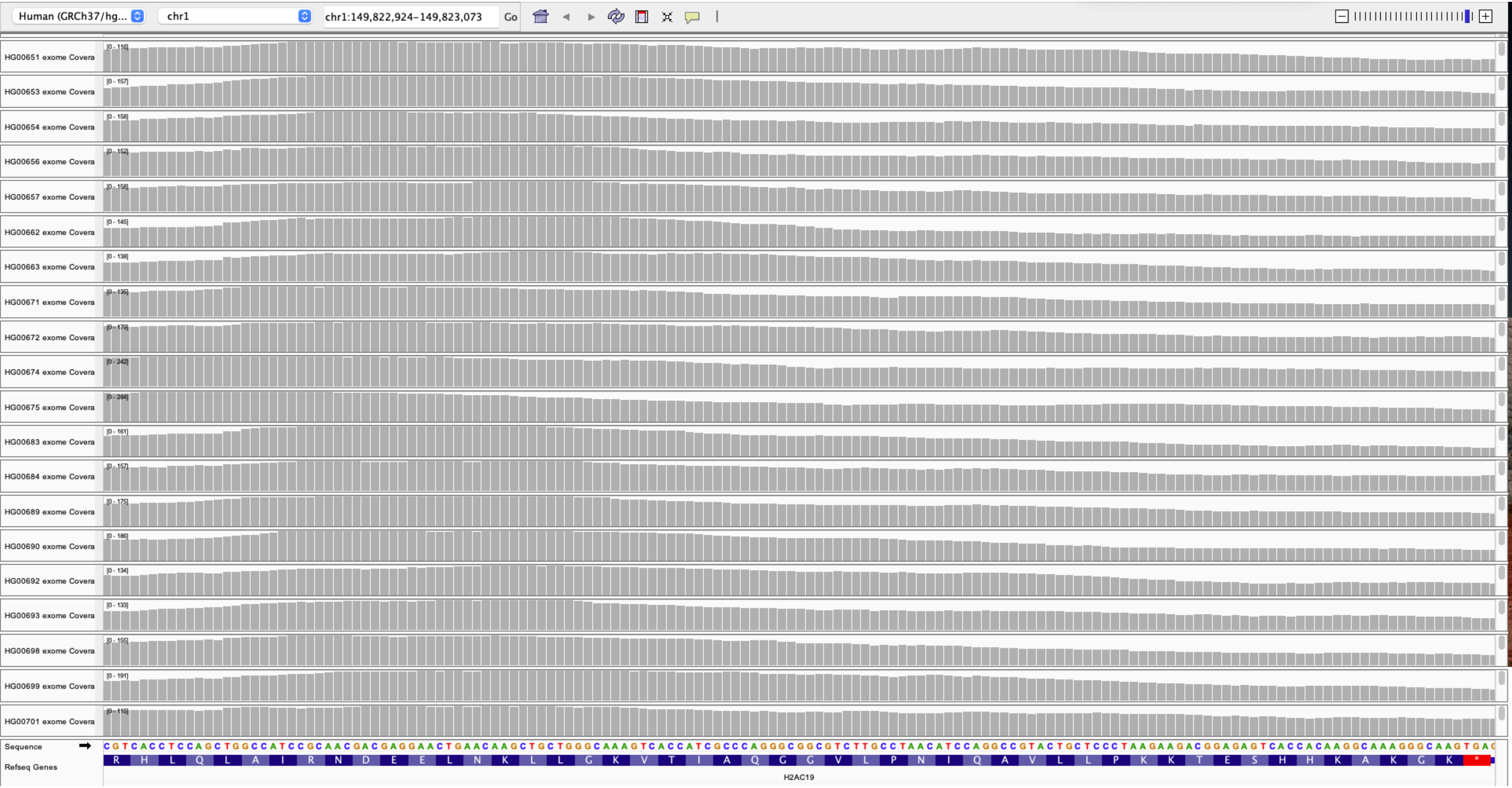

CHS: HG00702-HG00729

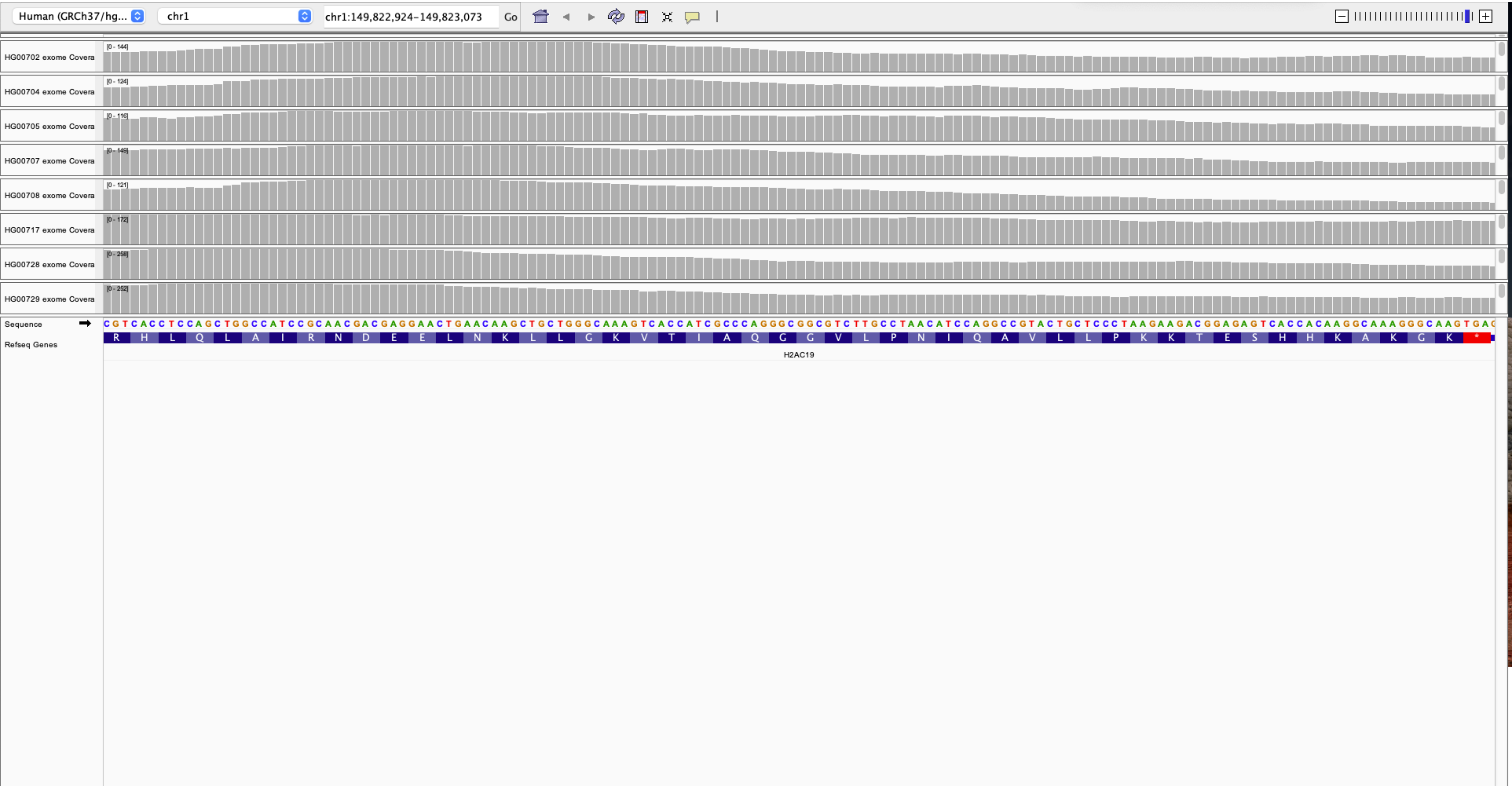

JPT: Japanese in Tokyo, Japan
(104 samples)

JPT: NA18939-NA18960

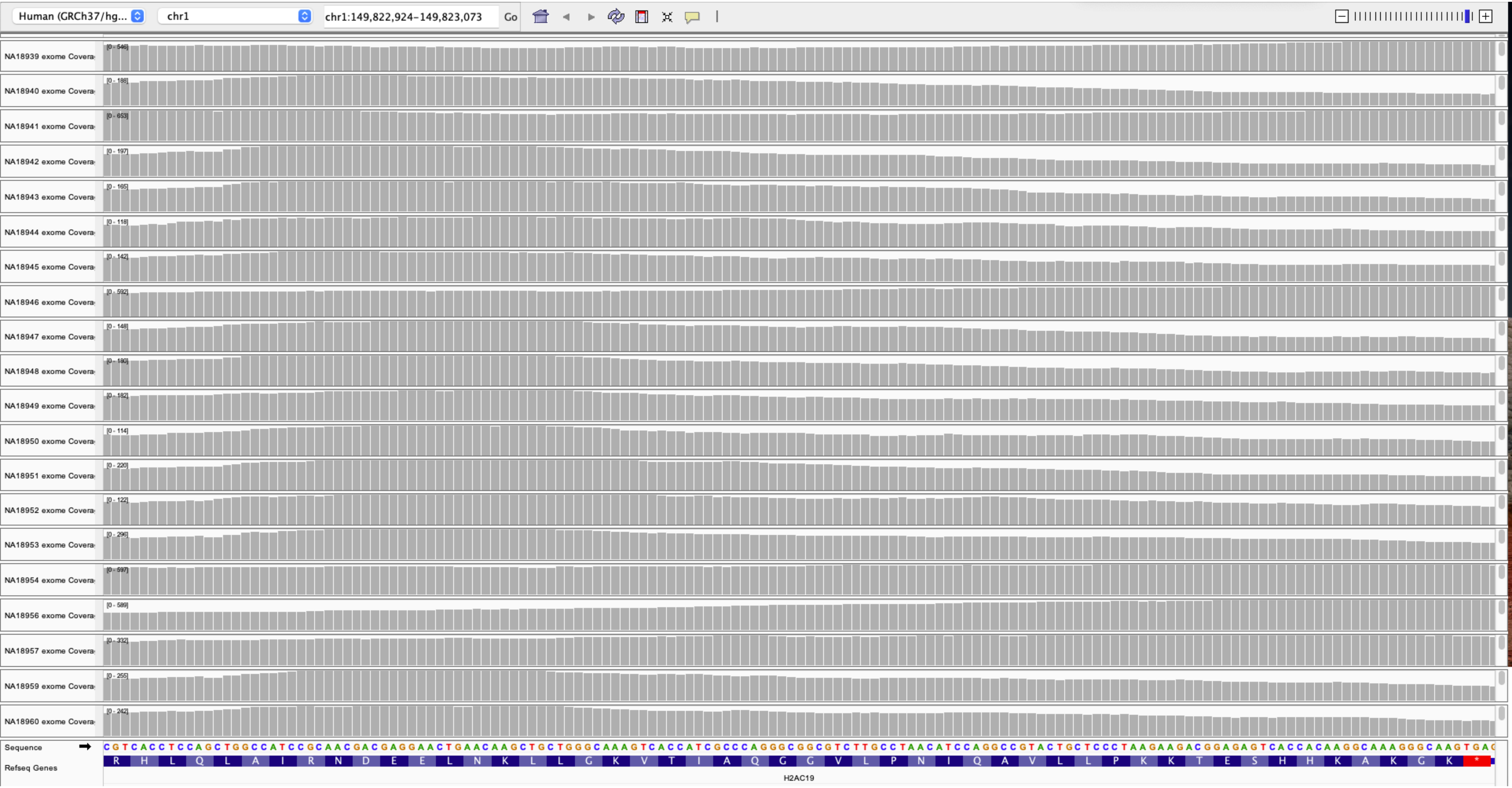

JPT: NA18961-NA18980

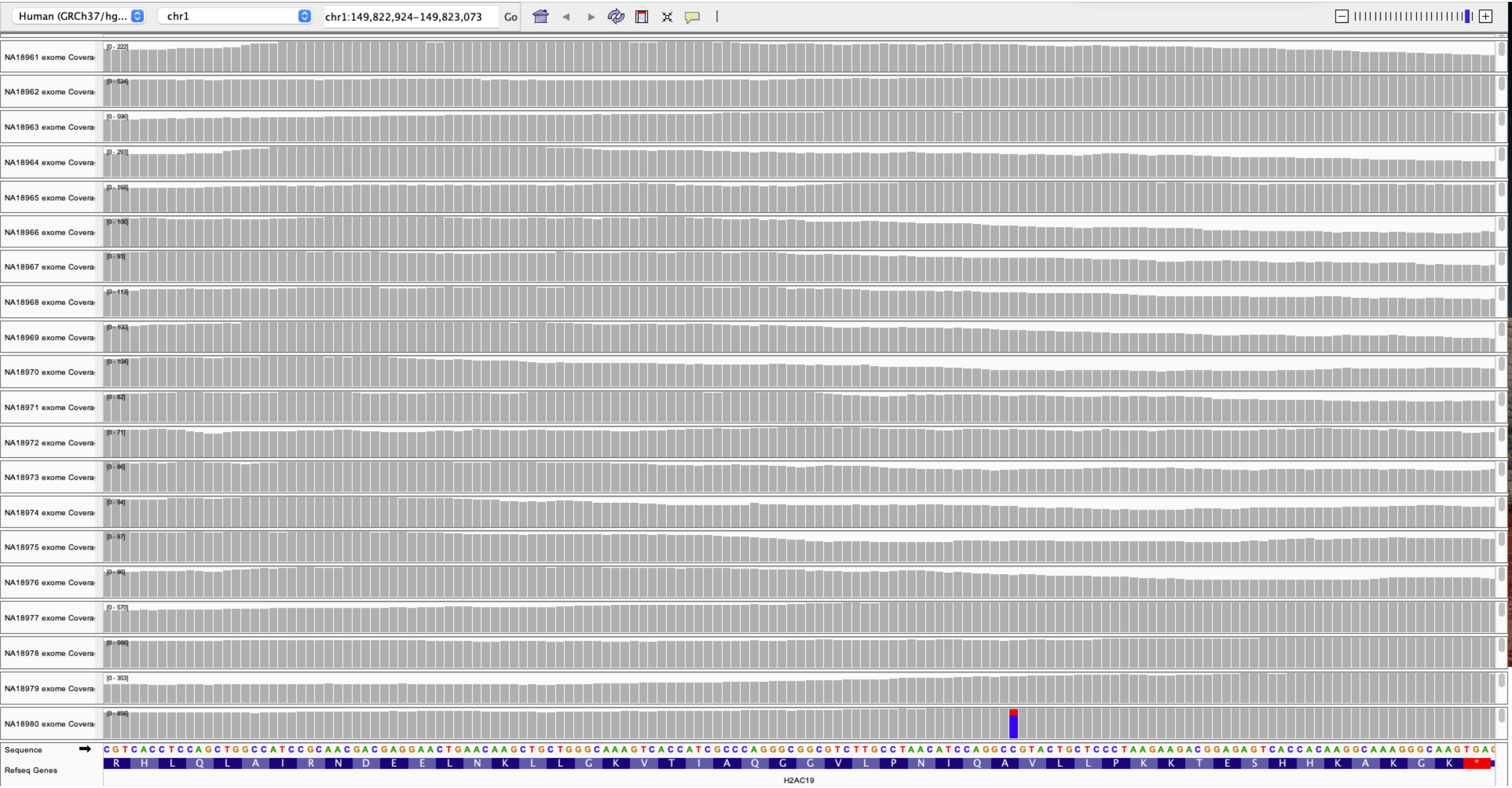

JPT: NA18981-NA19001

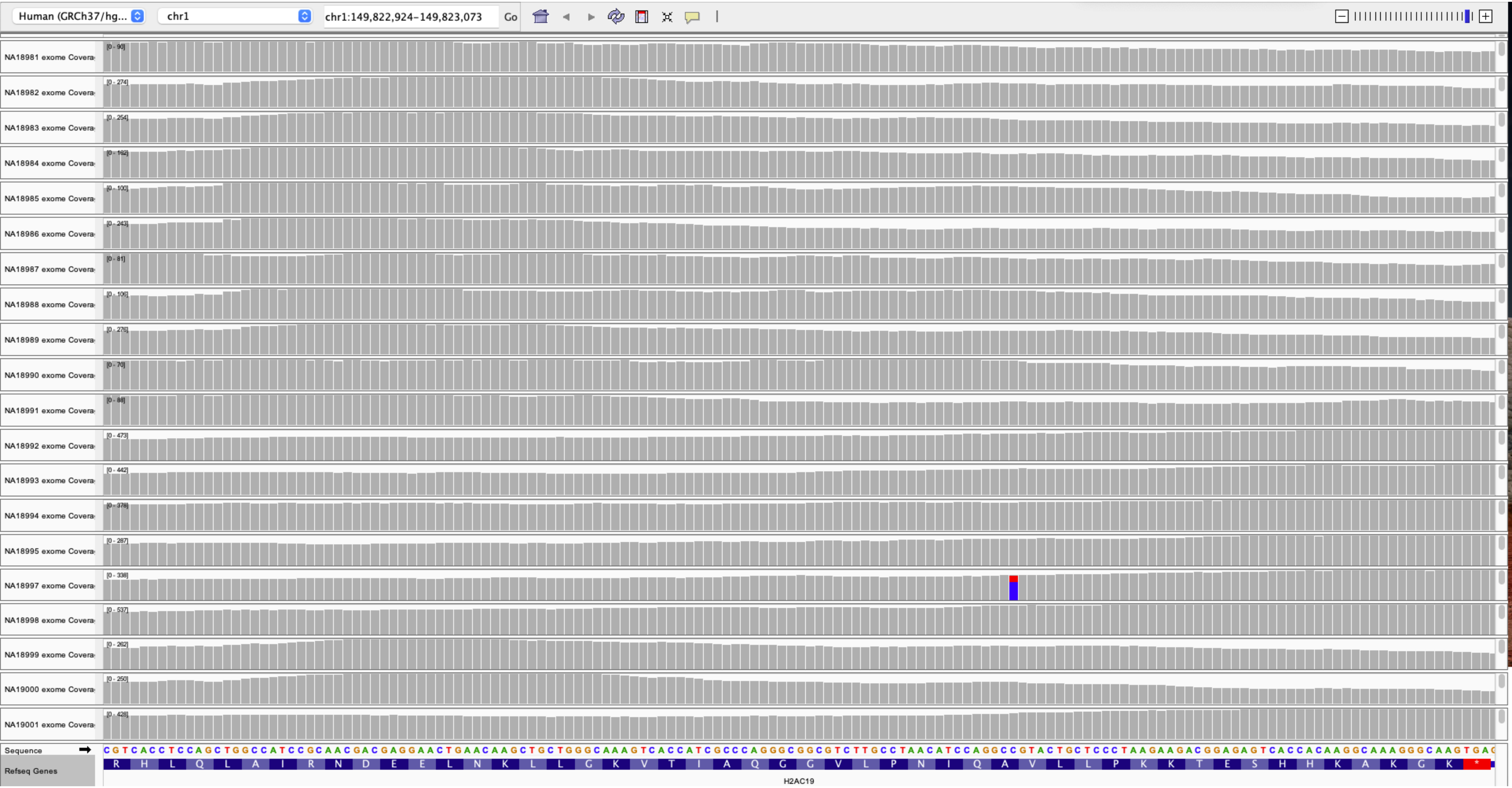

JPT: NA19002-NA19064

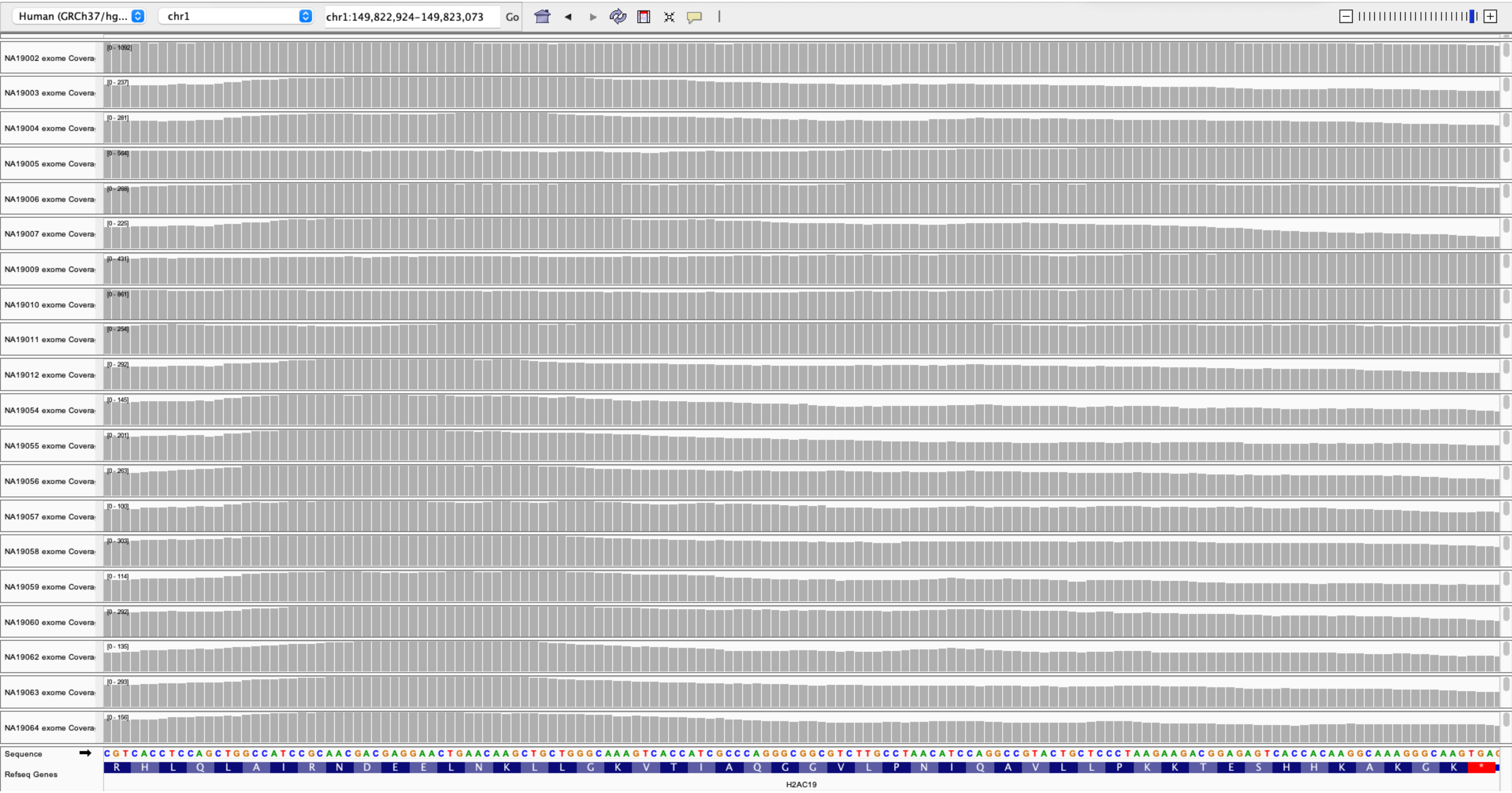

JPT: NA19065-NA19087

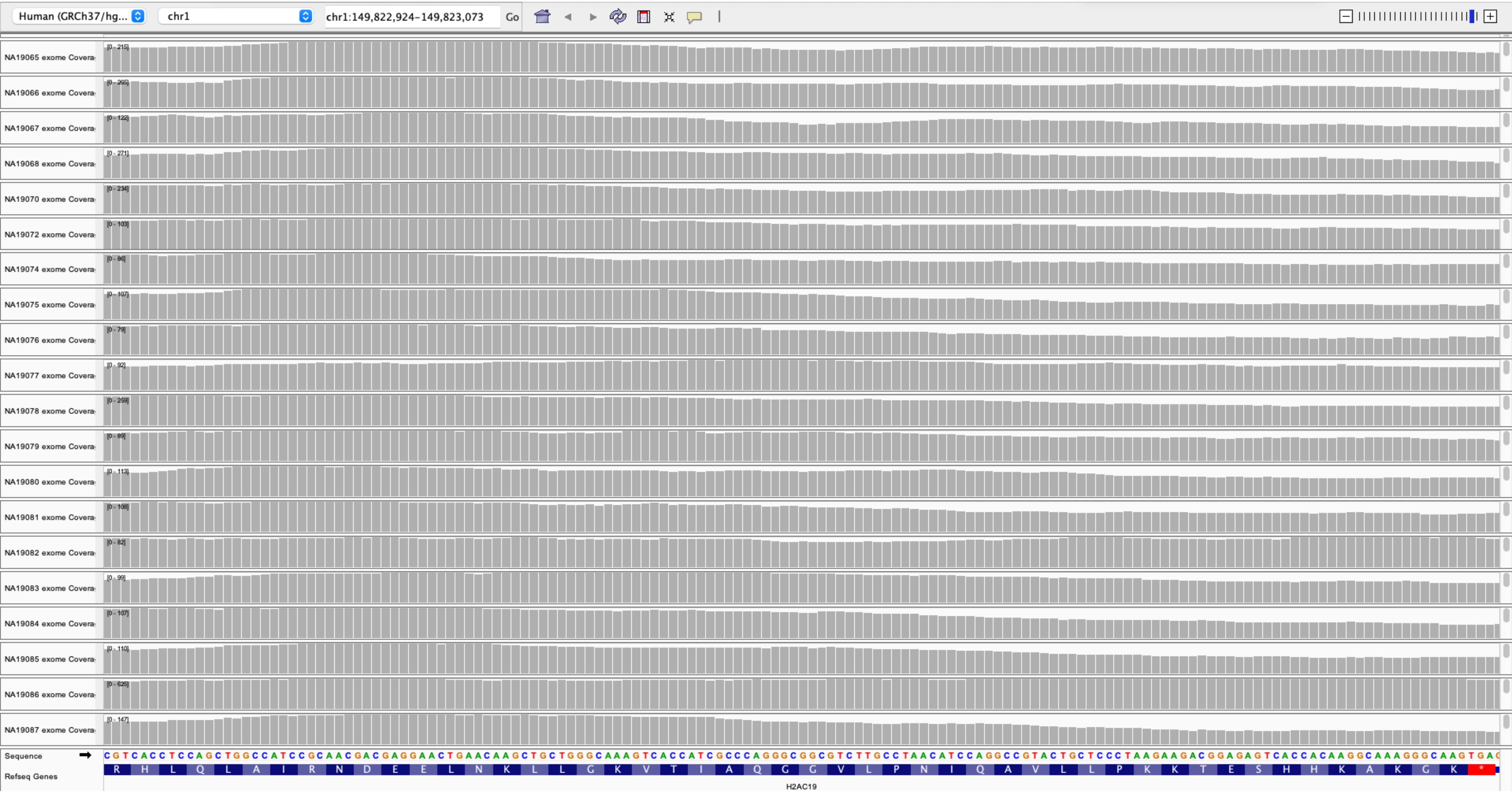

JPT: NA19088-NA19091

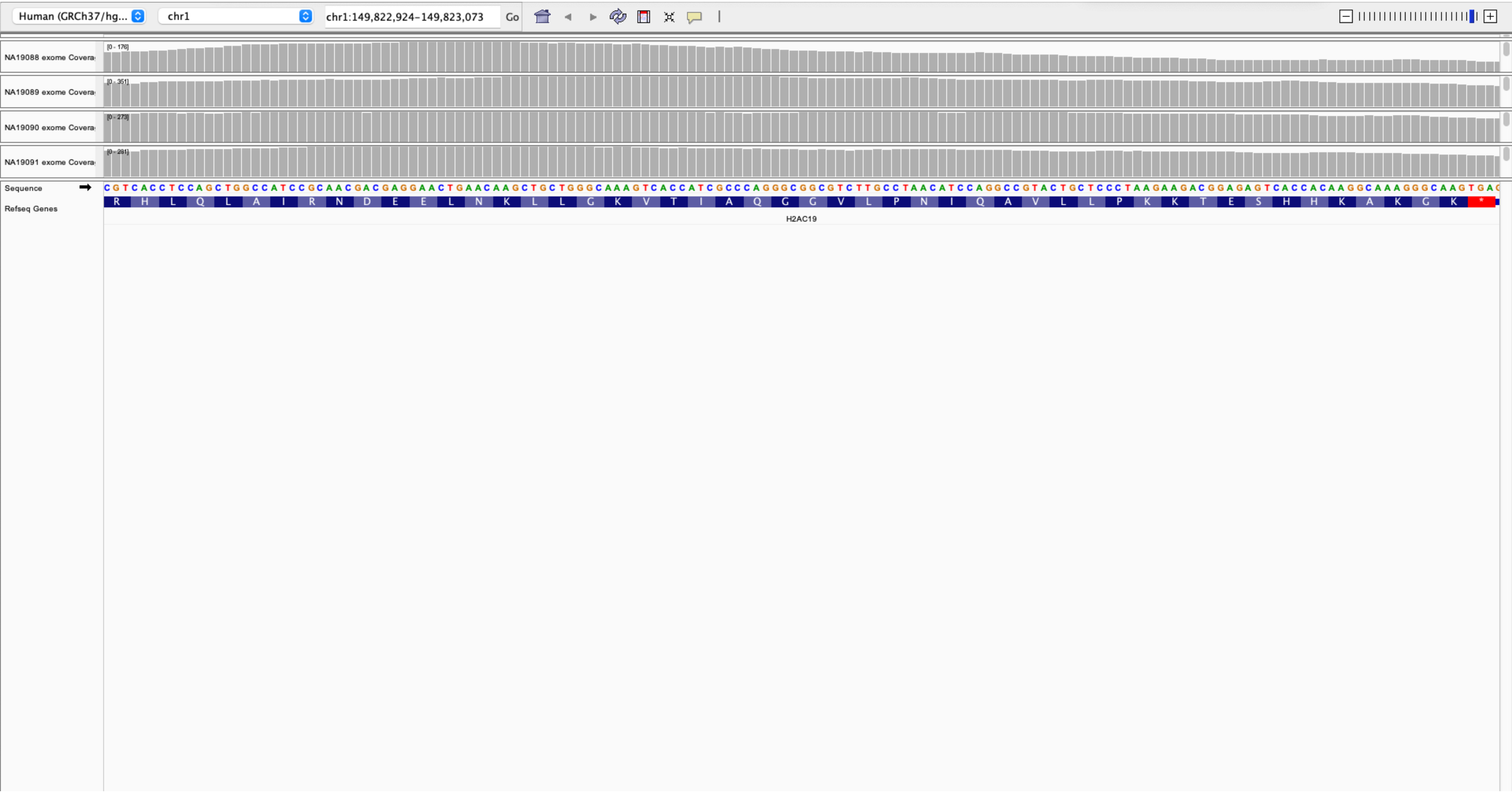

KHV: Kinh in Ho Chi Minh City, Vietnam (101 samples)

KHV: HG01595-HG01853

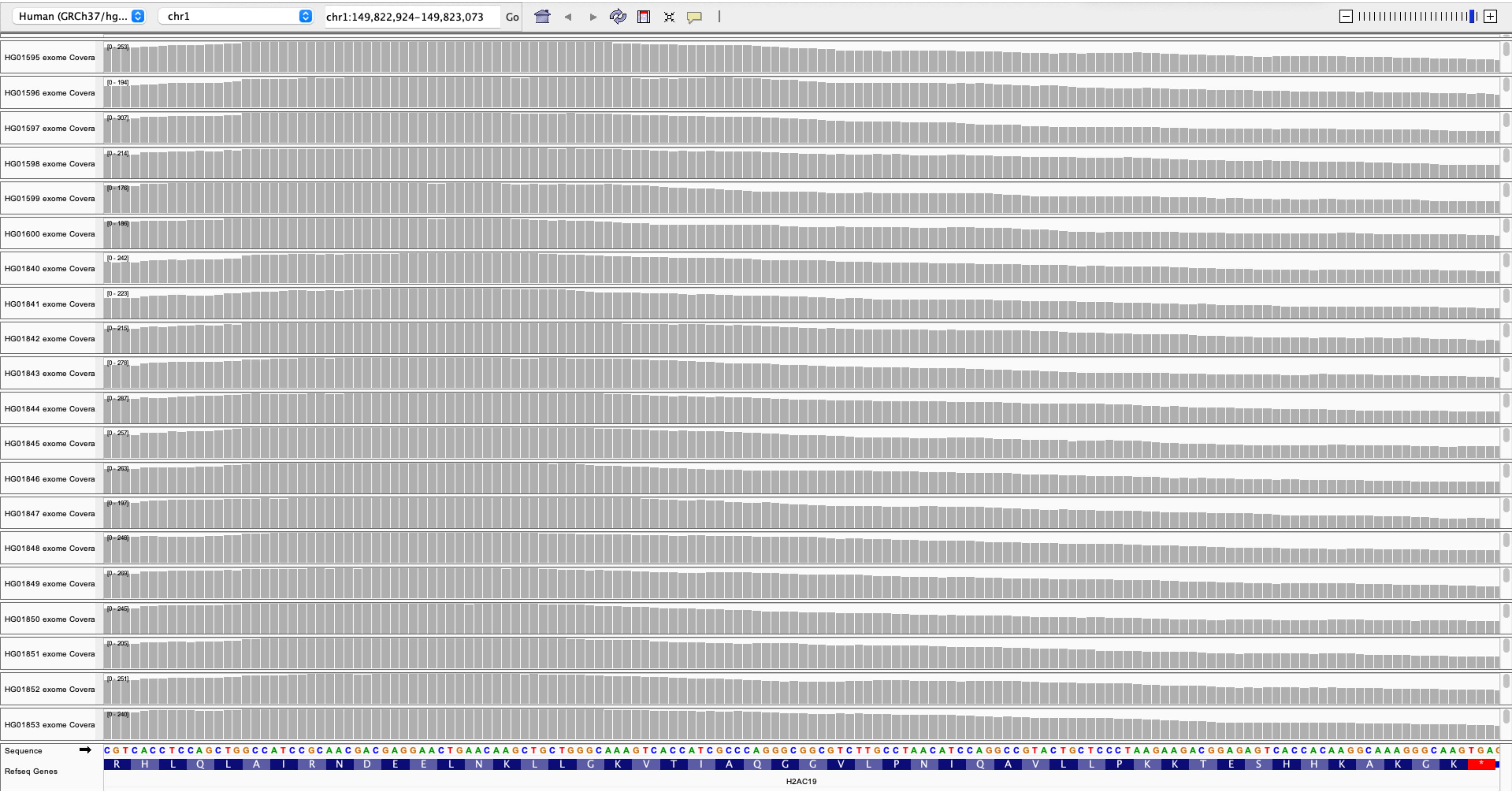

KHV: HG01855-HG01878

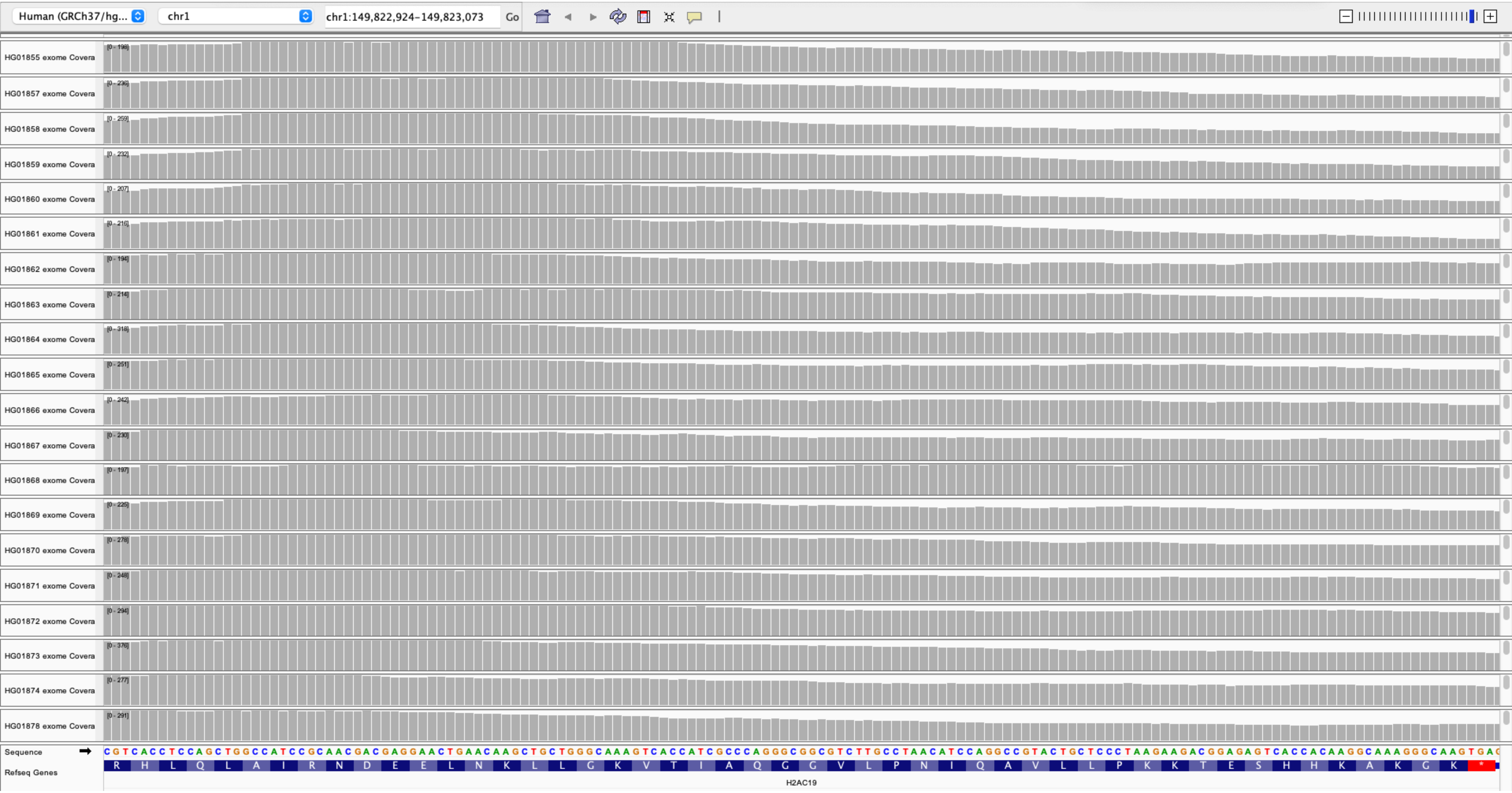

KHV.HG02016-HG02057

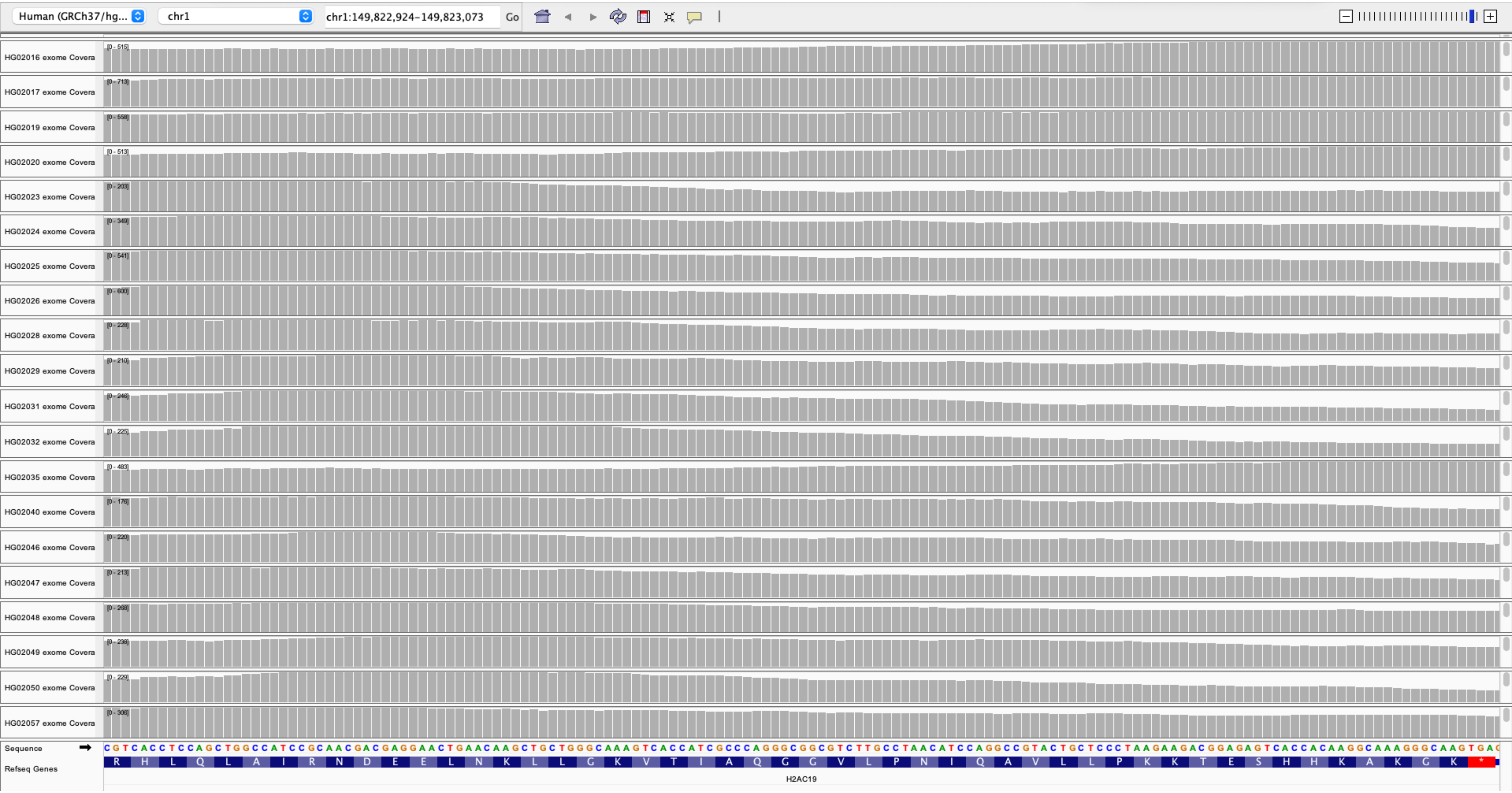

KHV: HG02058-HG02088

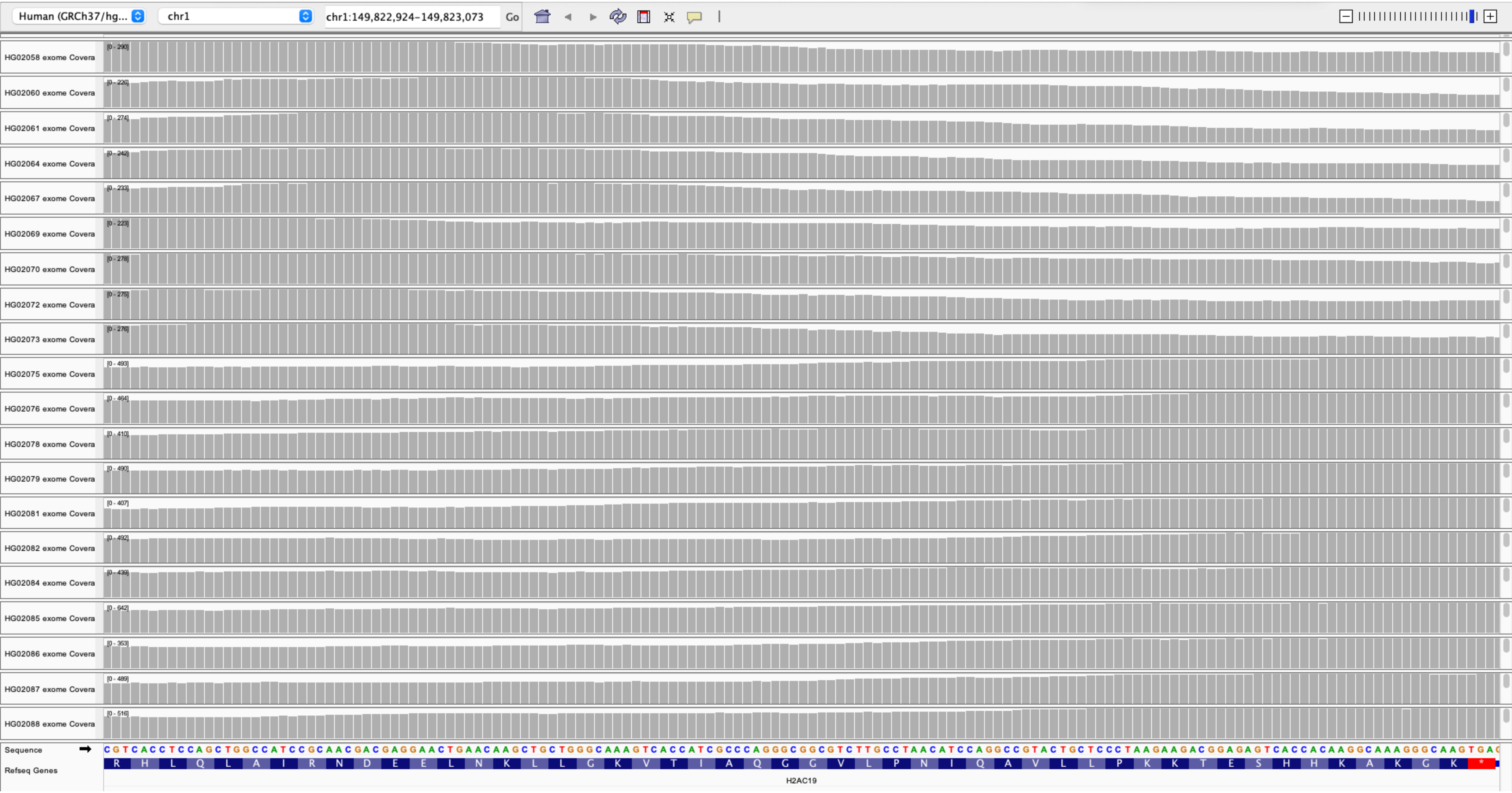

KHV: HG02113-HG02521

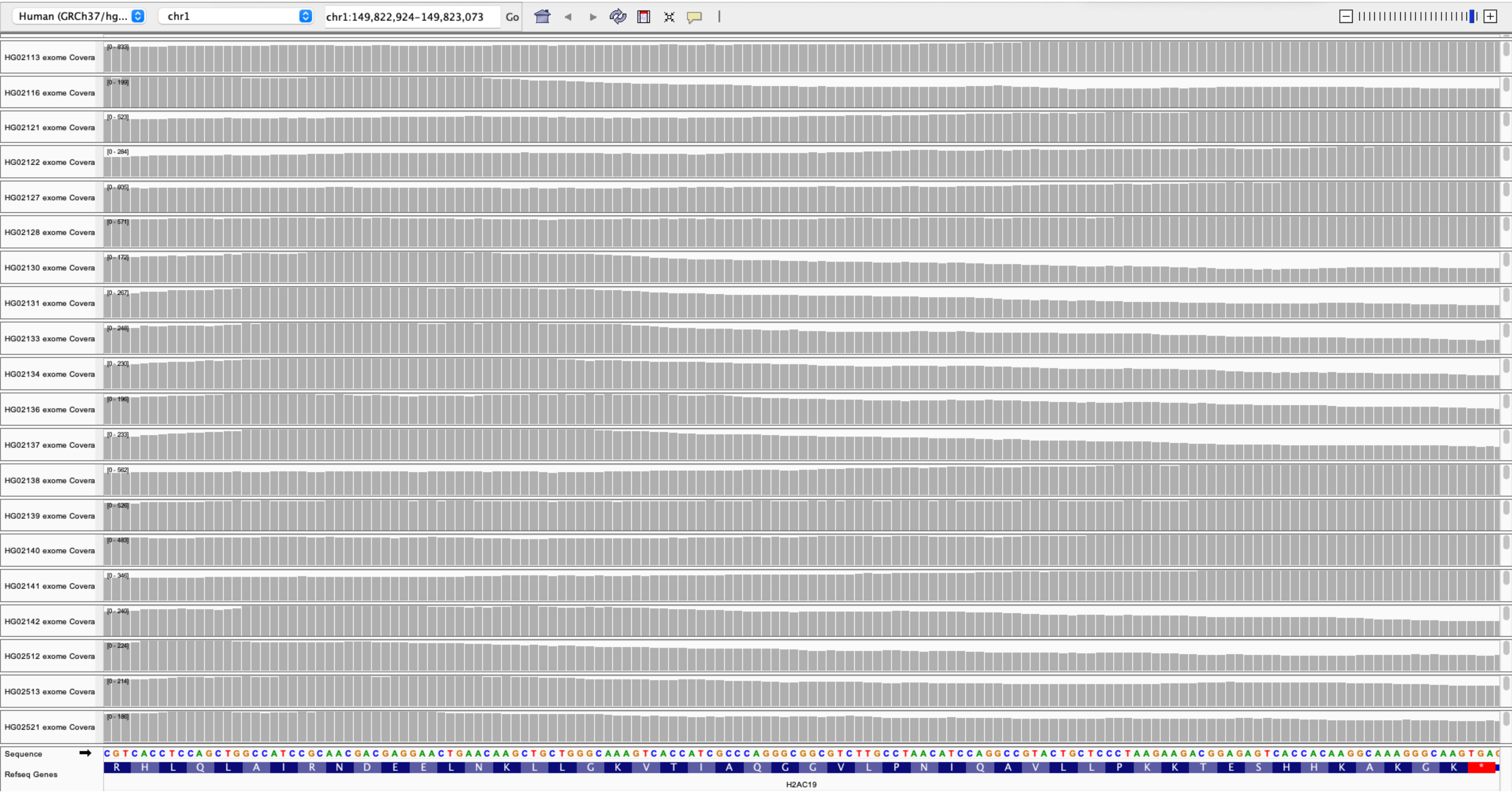

KHV: HG02522

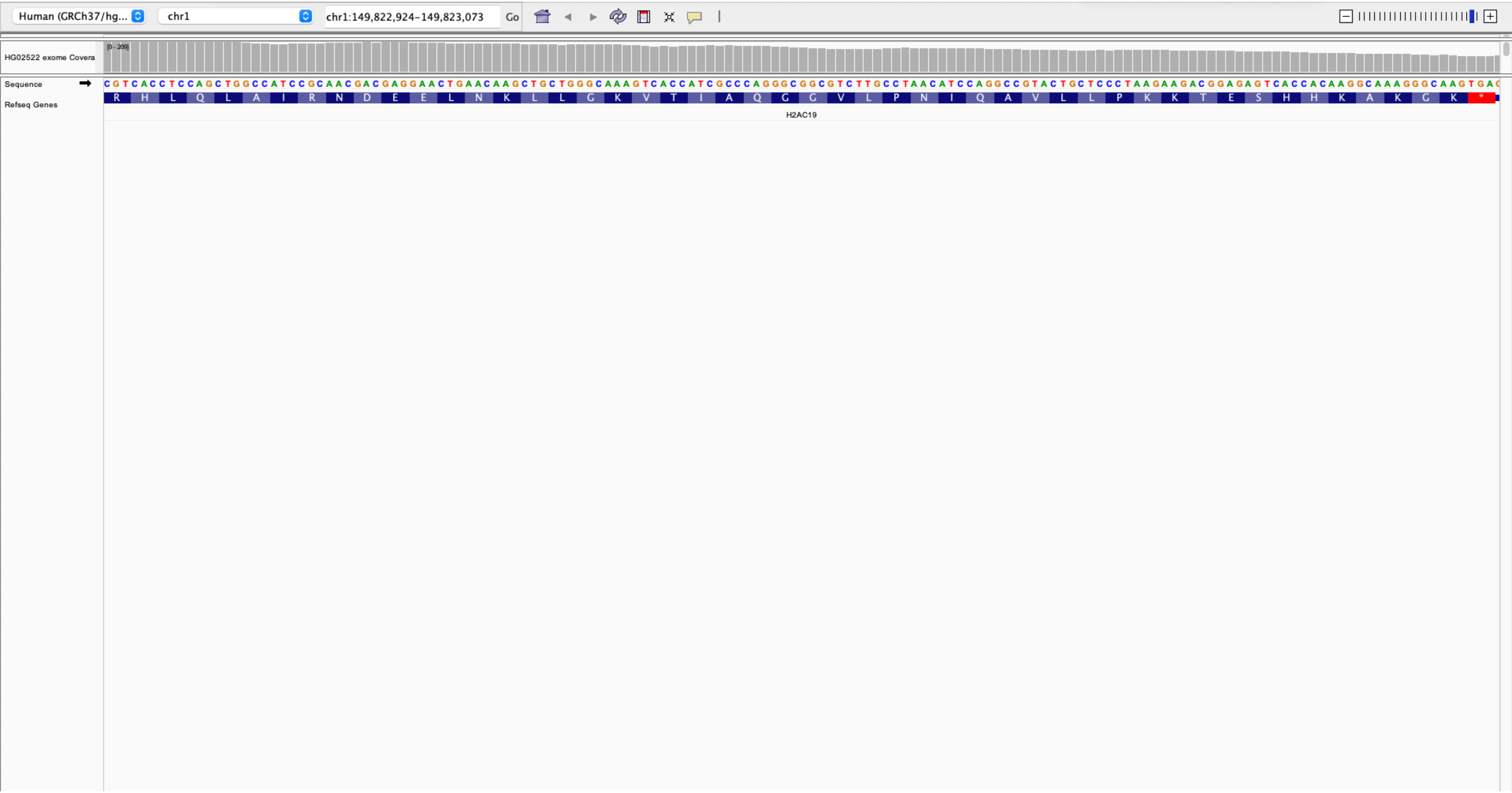
Human (GRCh37/hg...
chr1
chr1:149,822,924-149,823,073
Go
HG02522 exome Covera
[0 - 209]
Sequence
Refseq Genes
H2AC19

GIH: Gujarati Indians in Houston, TX, USA (106 samples)

GIH: NA20845-NA20869

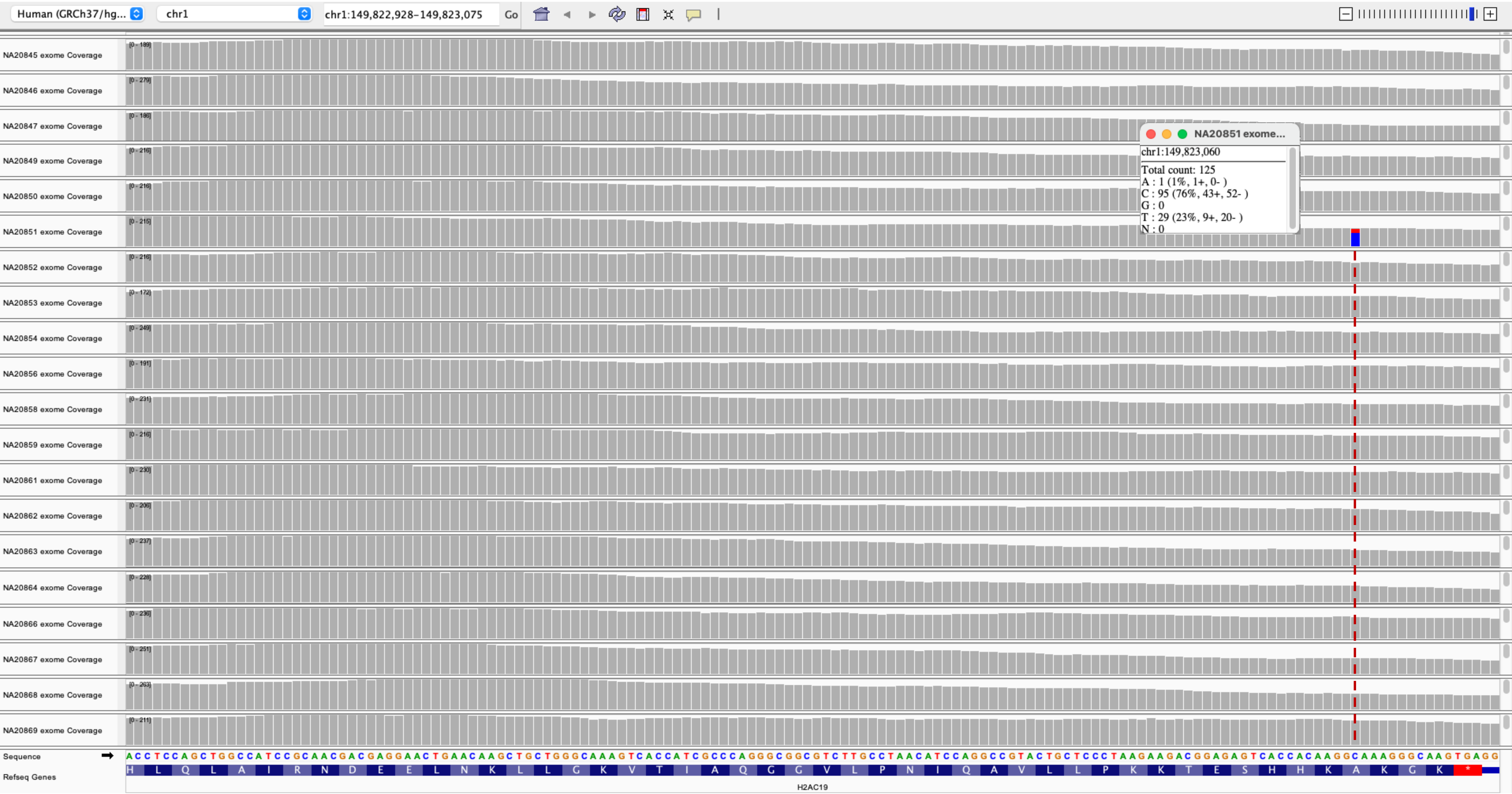

GIH: NA20870-NA20893

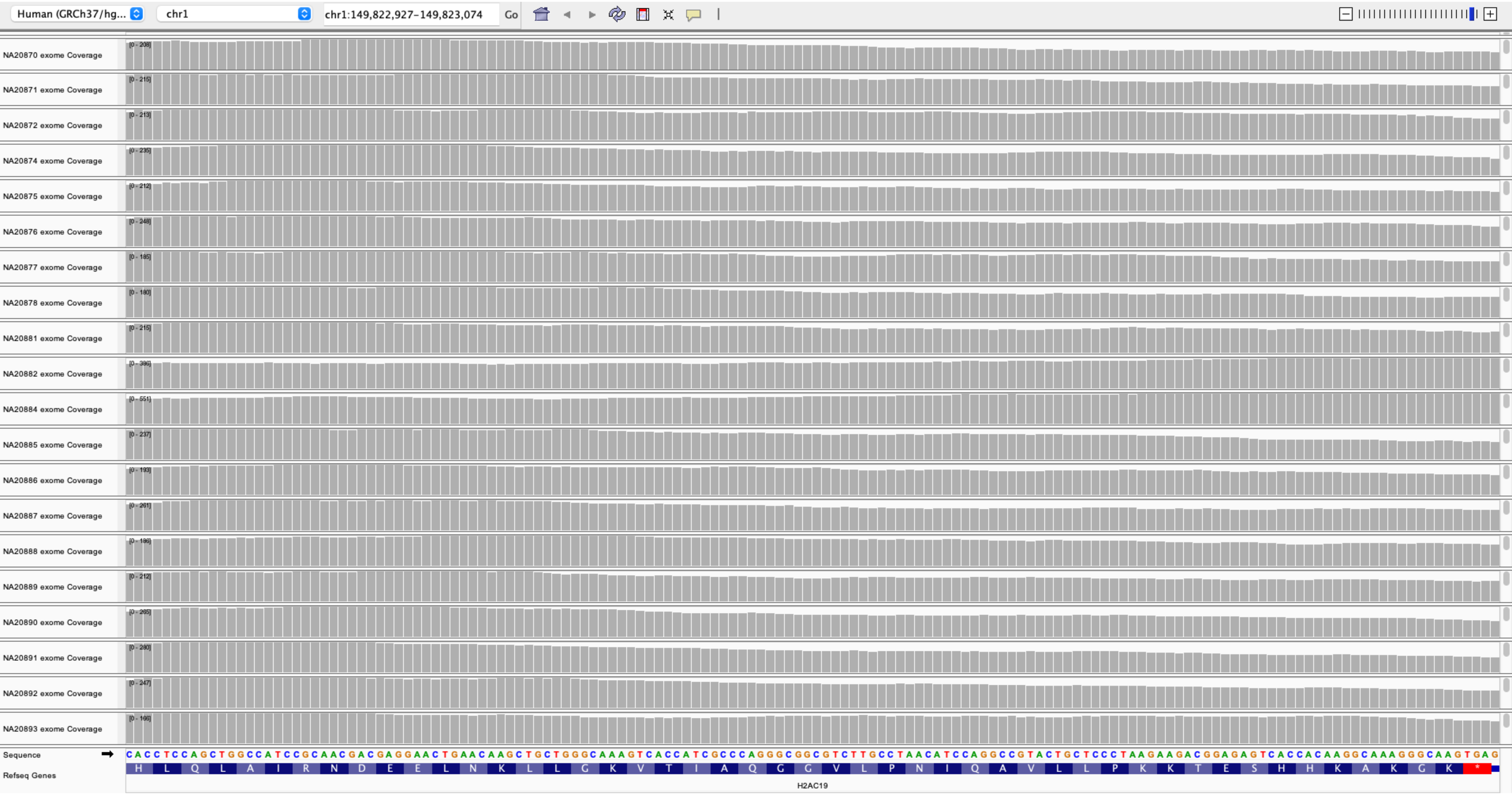

GIH: NA20894-NA21089

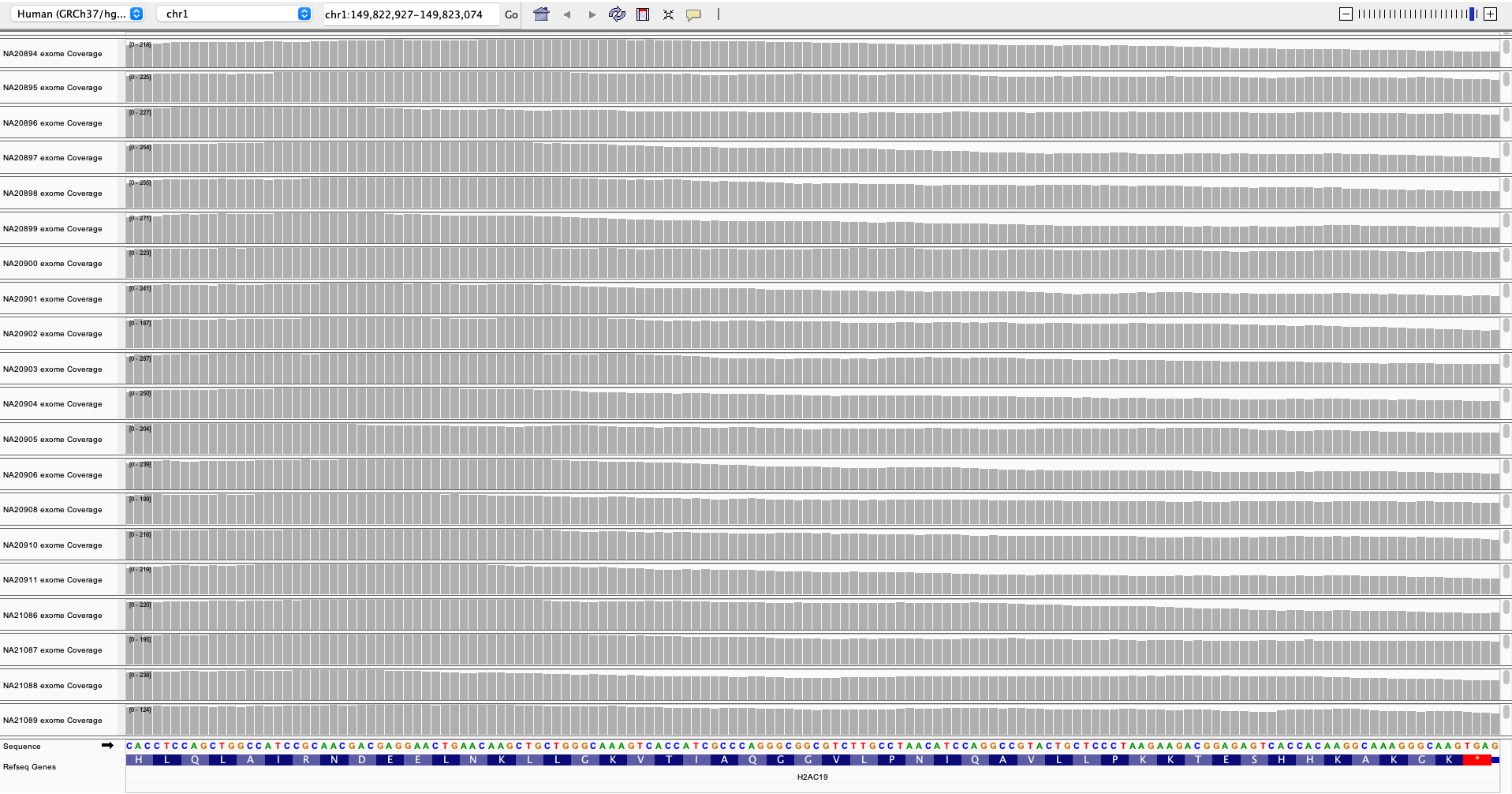

GIH: NA21090-NA21110

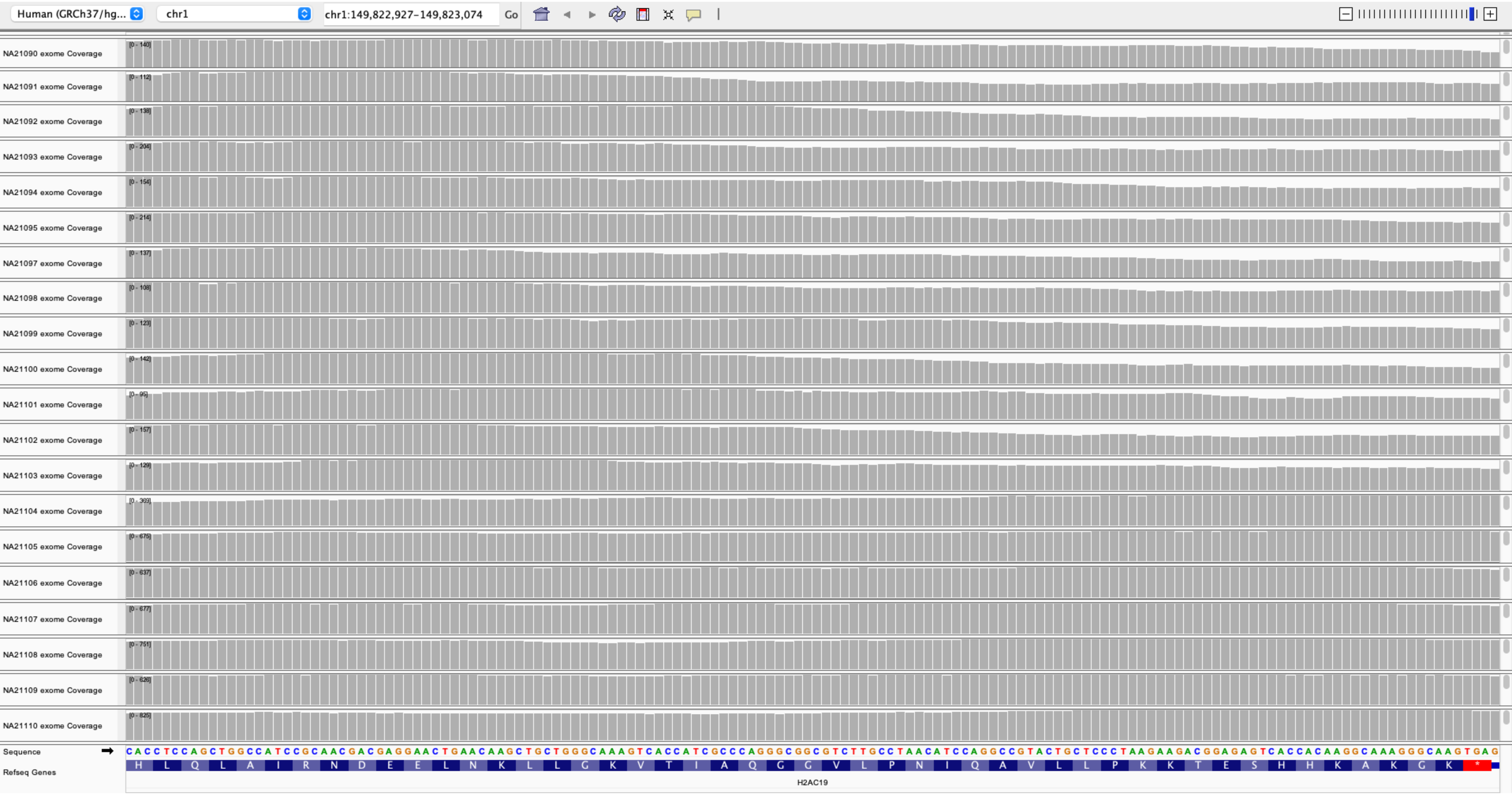

GIH: NA21111-NA21133

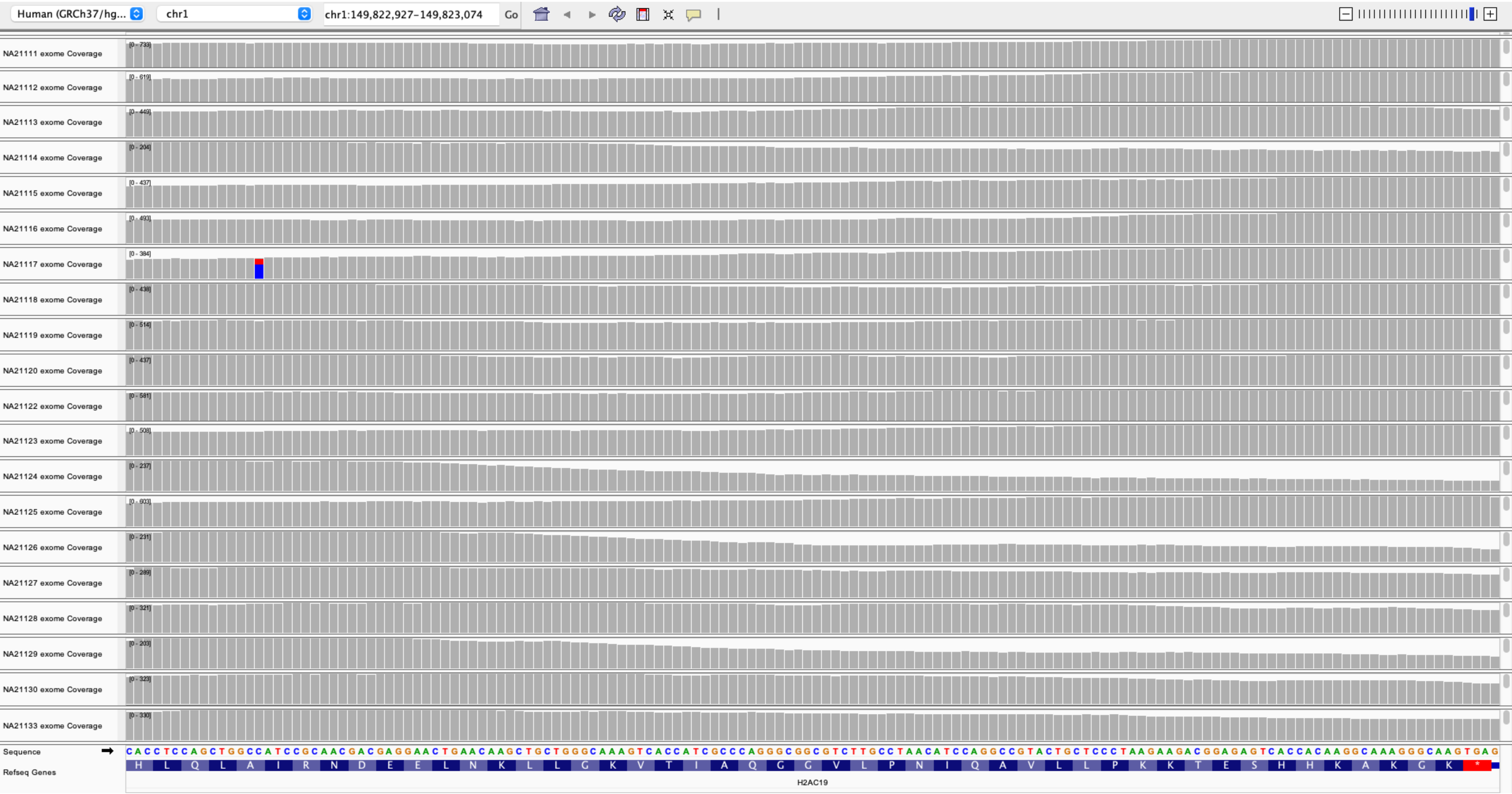

GIH: NA21135-NA21144

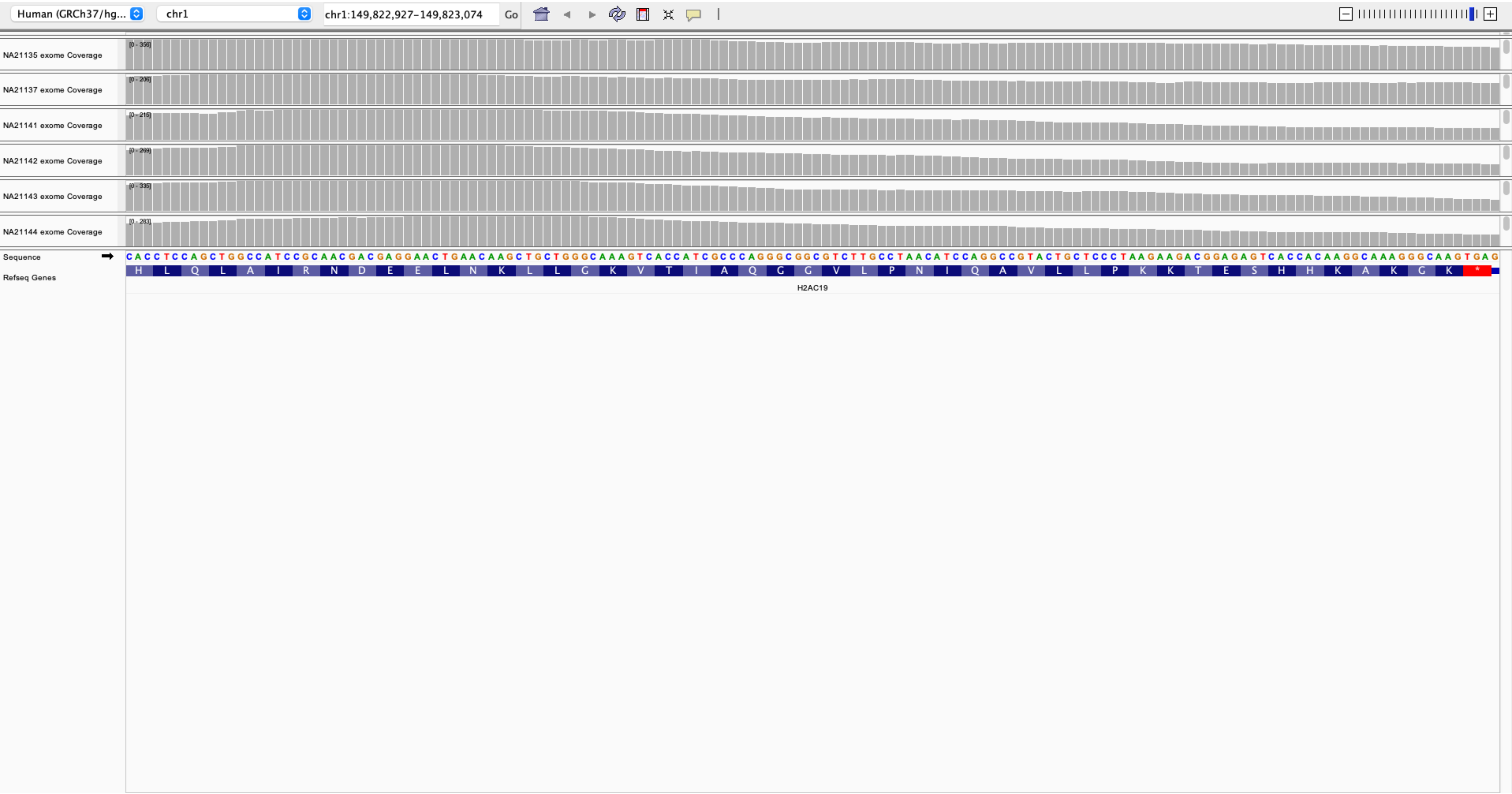

ITU: Indian Telugu in the UK (103 samples)

ITU: HG03713-HG03777

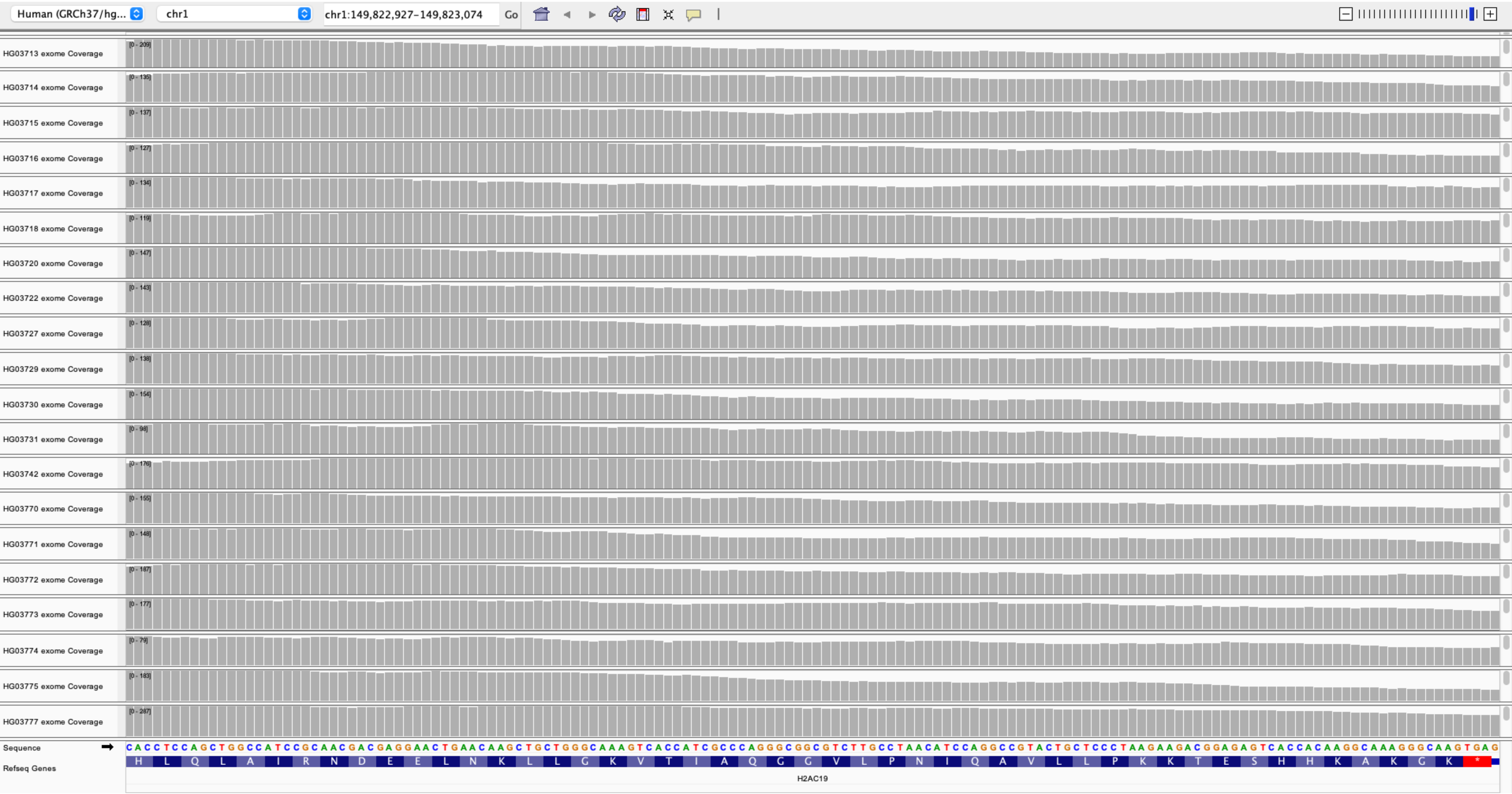

ITU: HG03778-HG03868

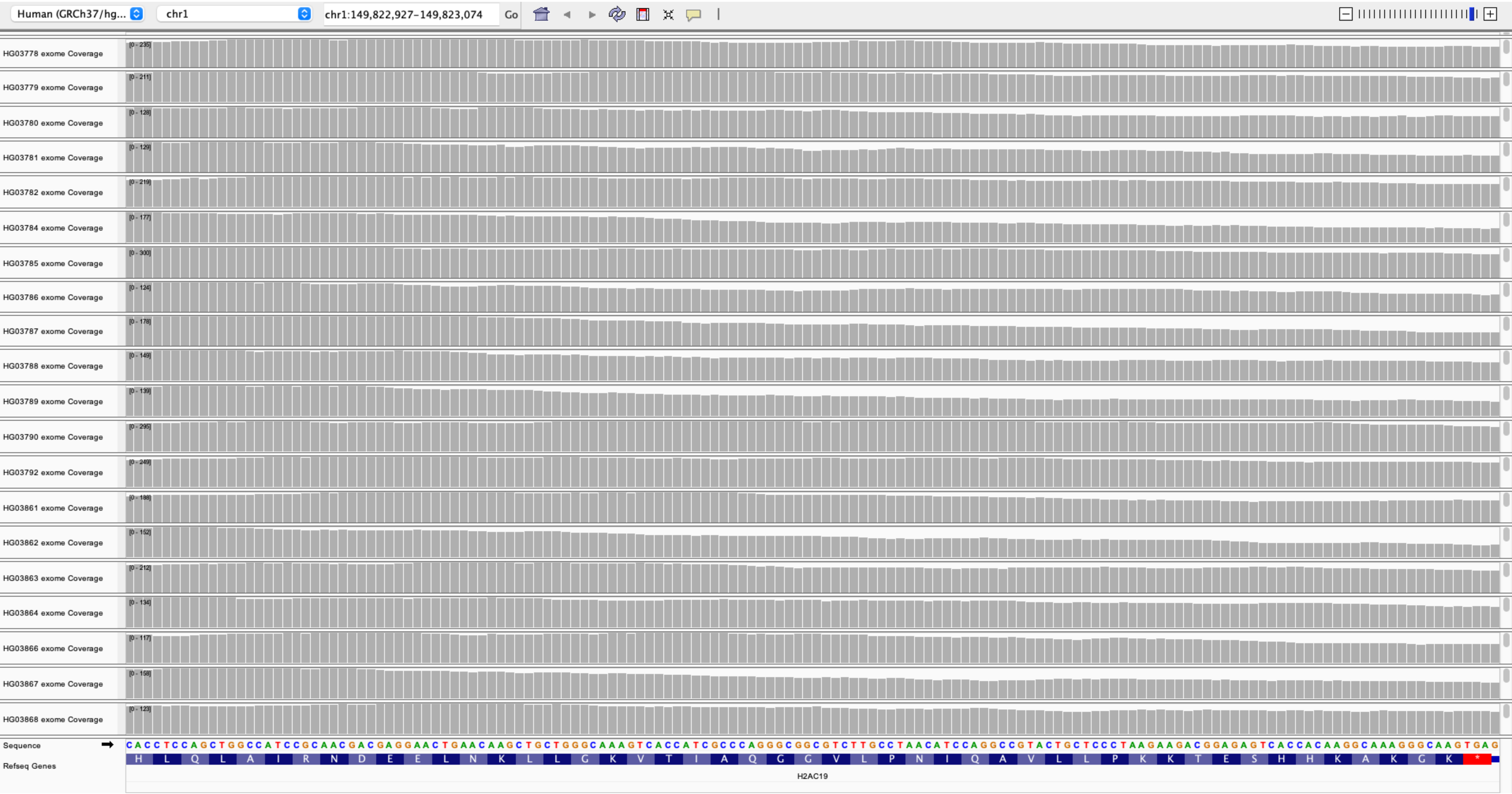

ITU: HG03869-HG03978

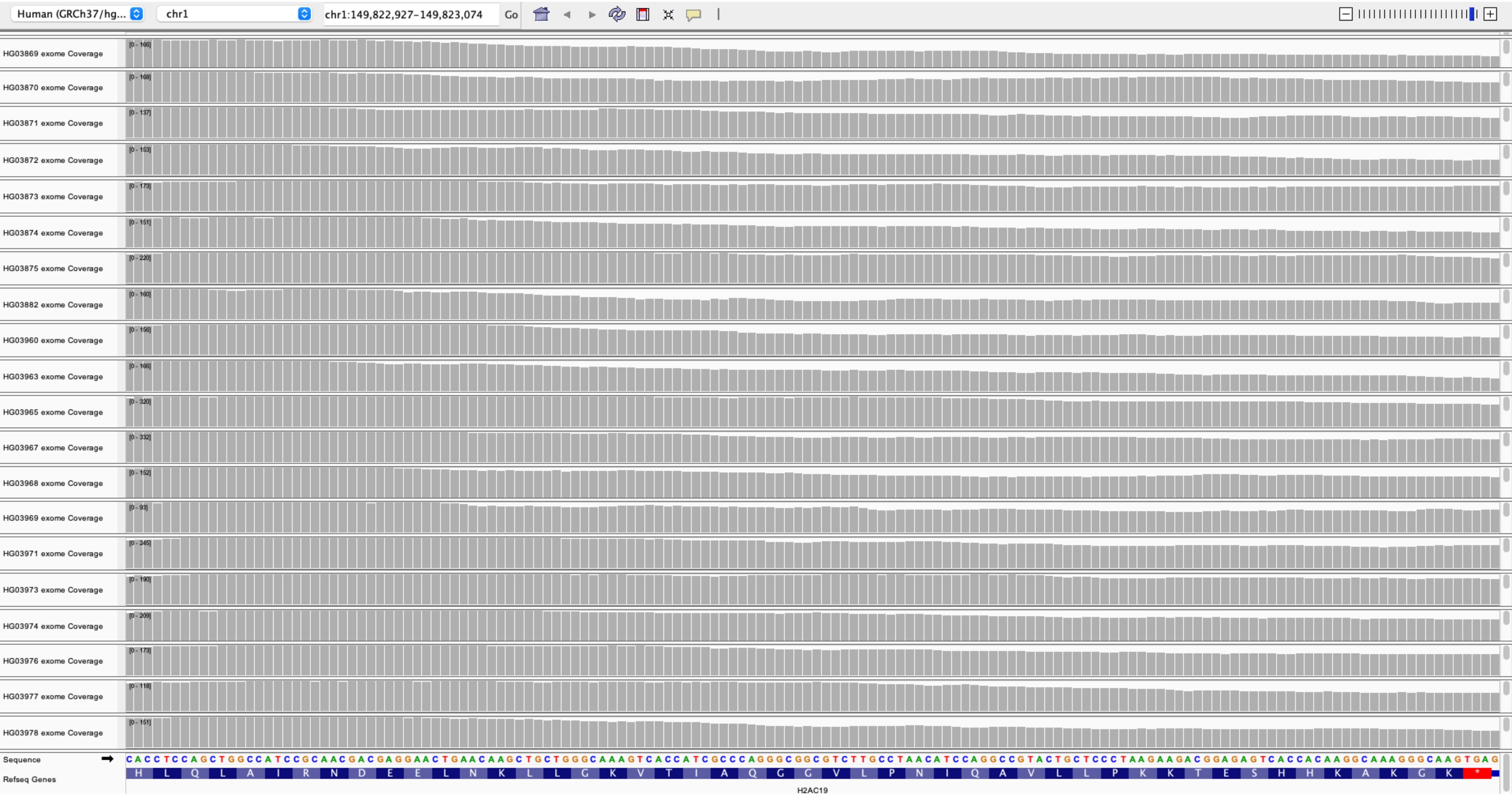

ITU: HG04001-HG04070

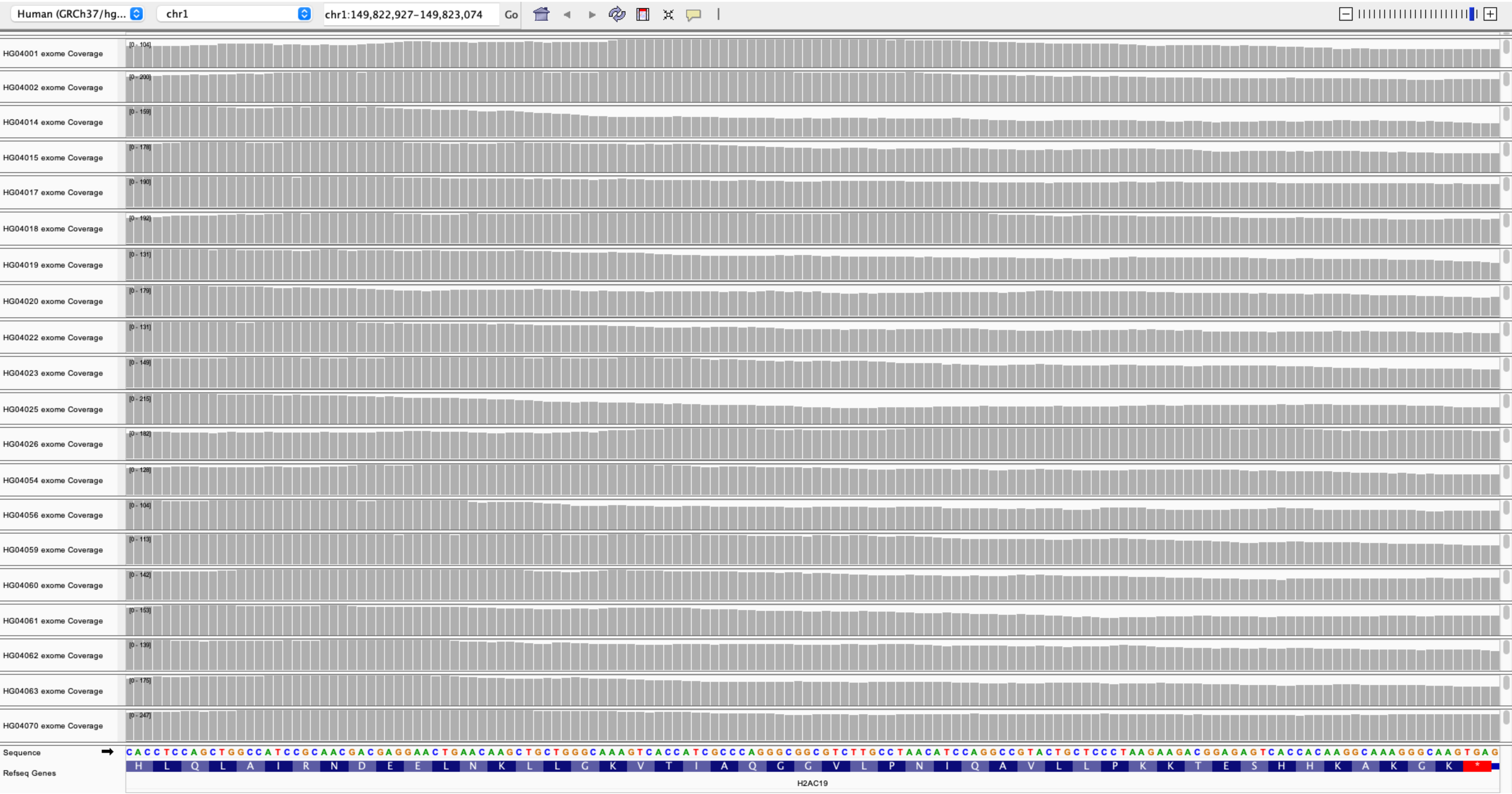

ITU: HG04076-HG04225

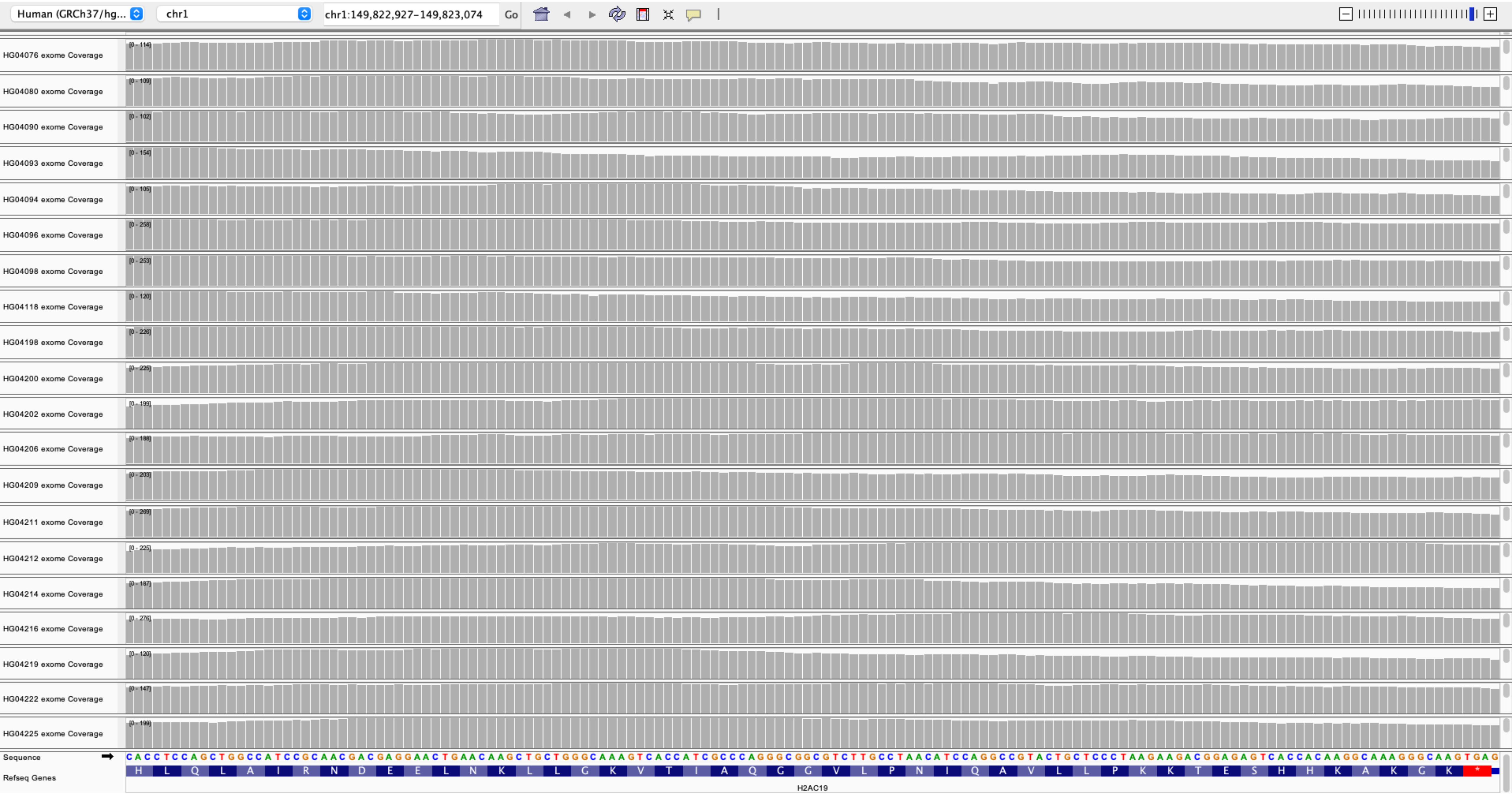

ITU: HG04235-HG04239

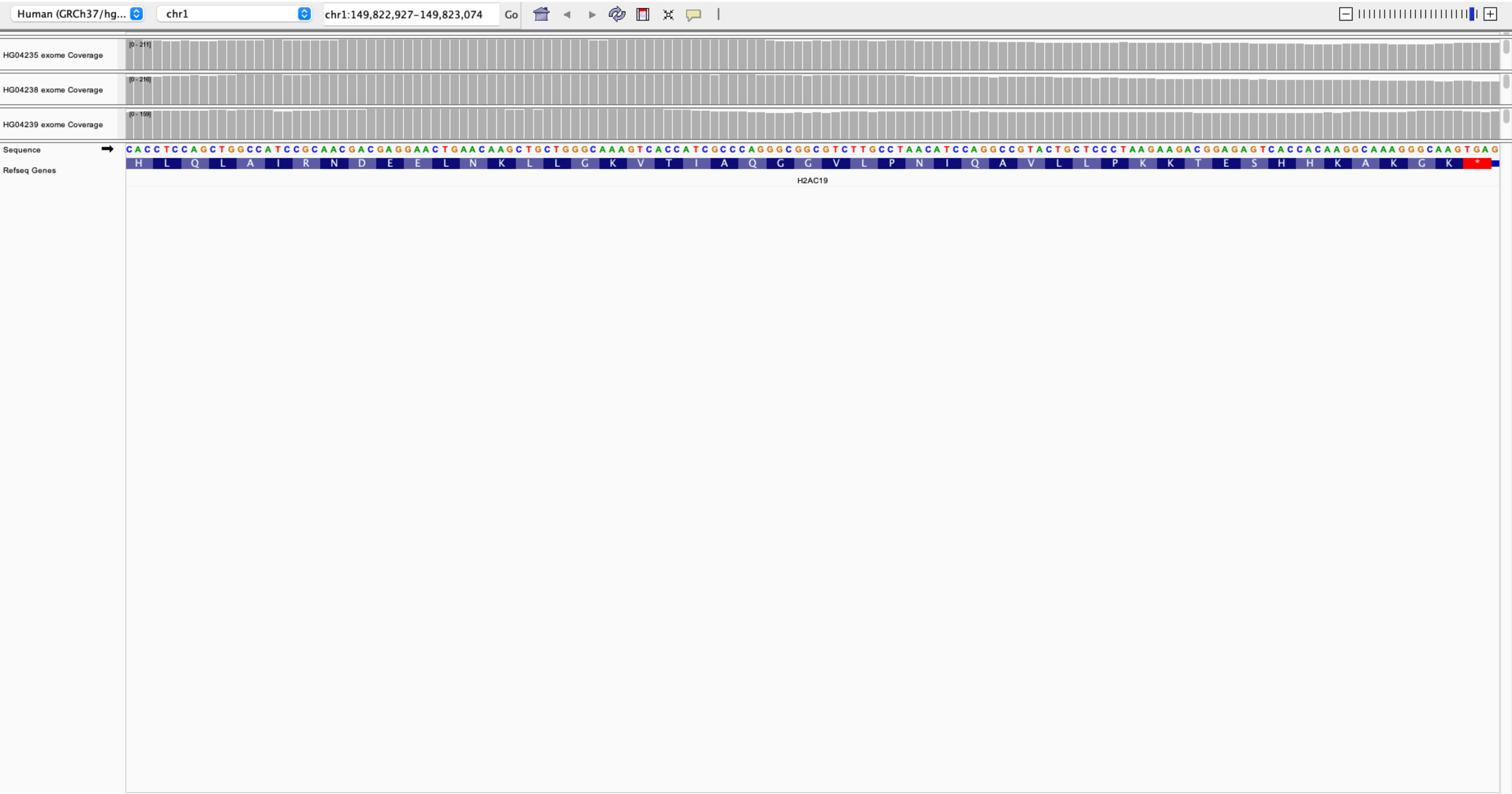

PJL: Punjabi in Lahore, Pakistan (96 samples)

PJL: HG01583-HG02657

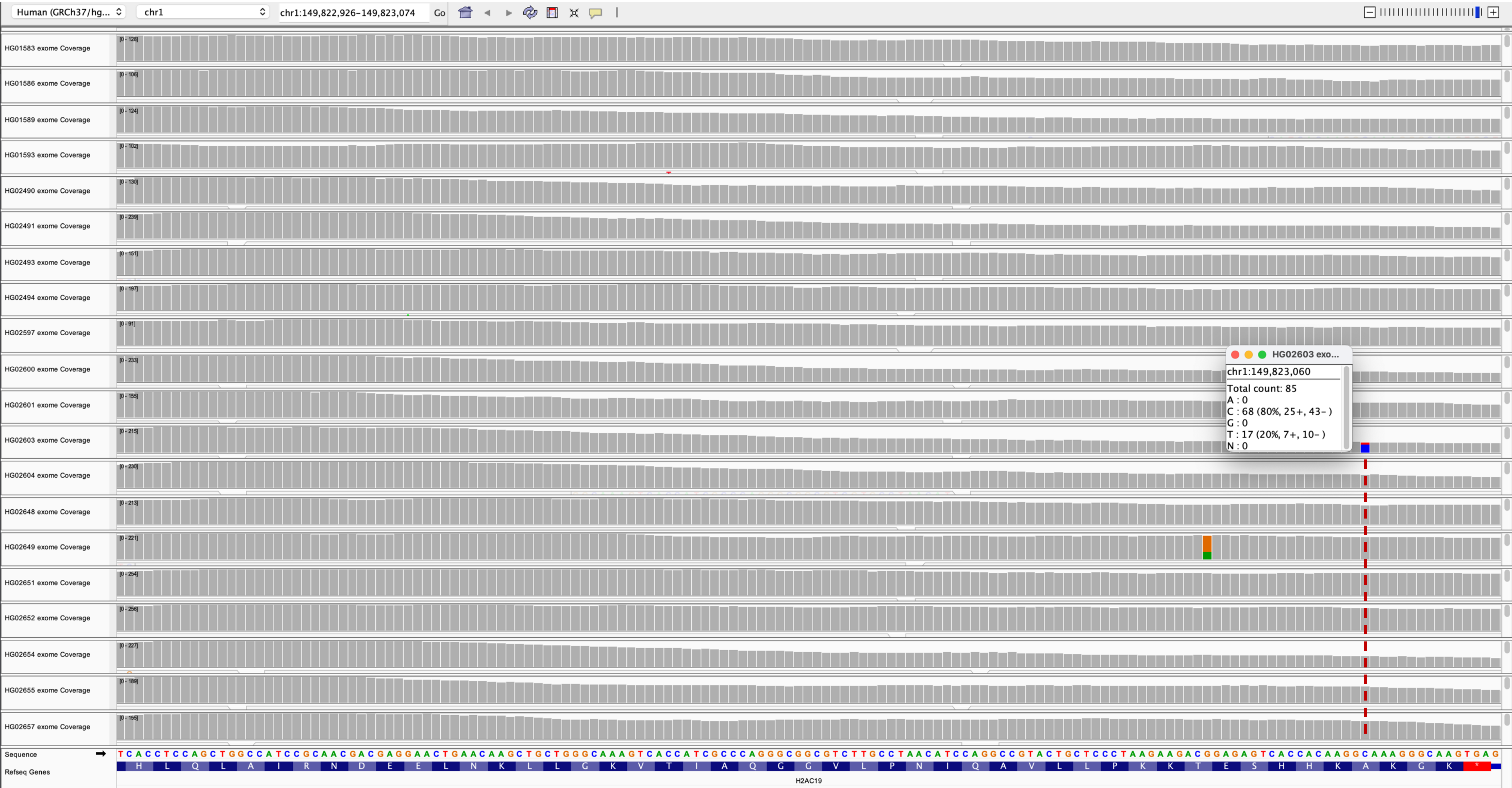

PJL: HG02658-HG02728

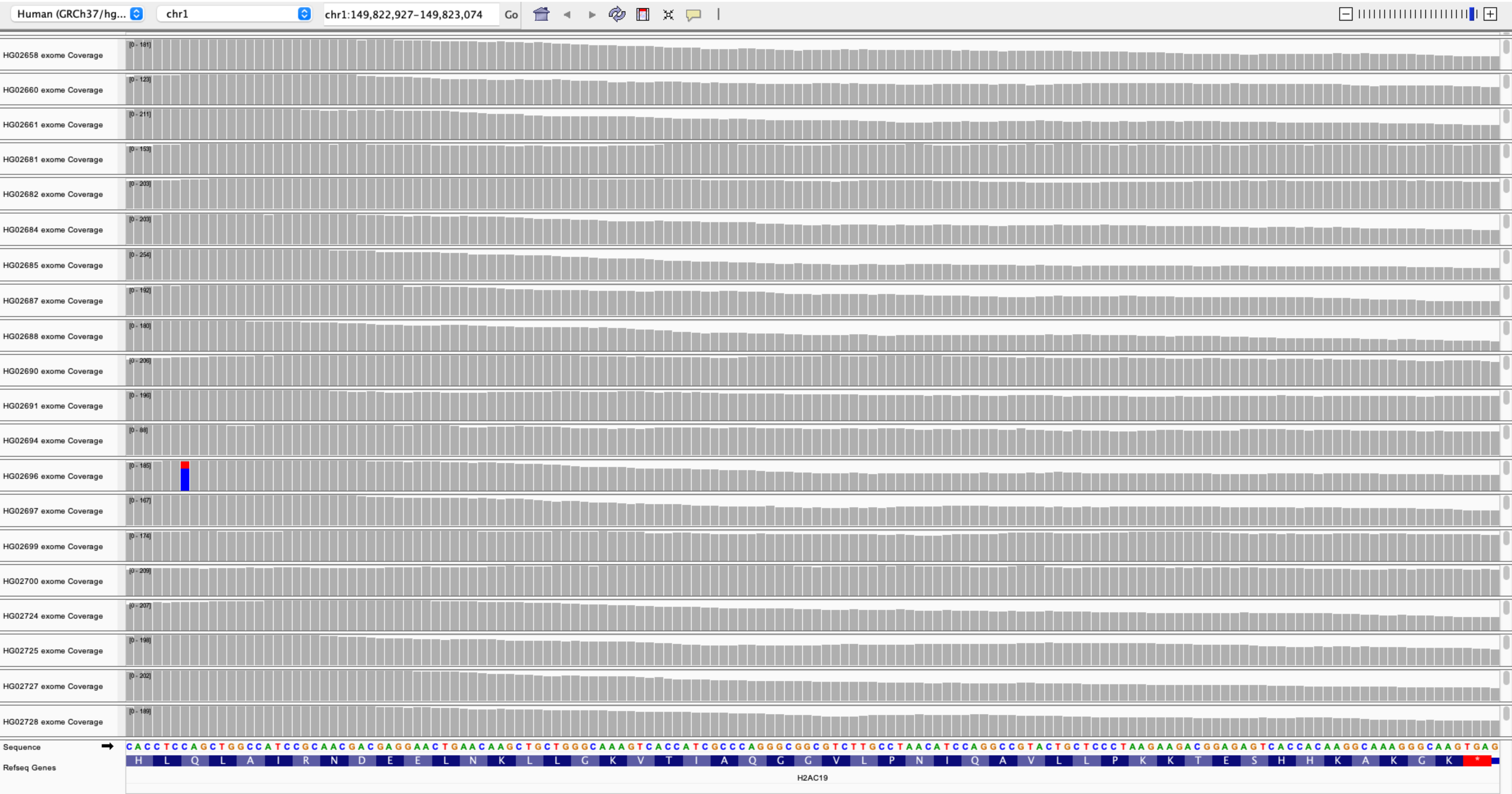

PJL: HG02731-HG03018

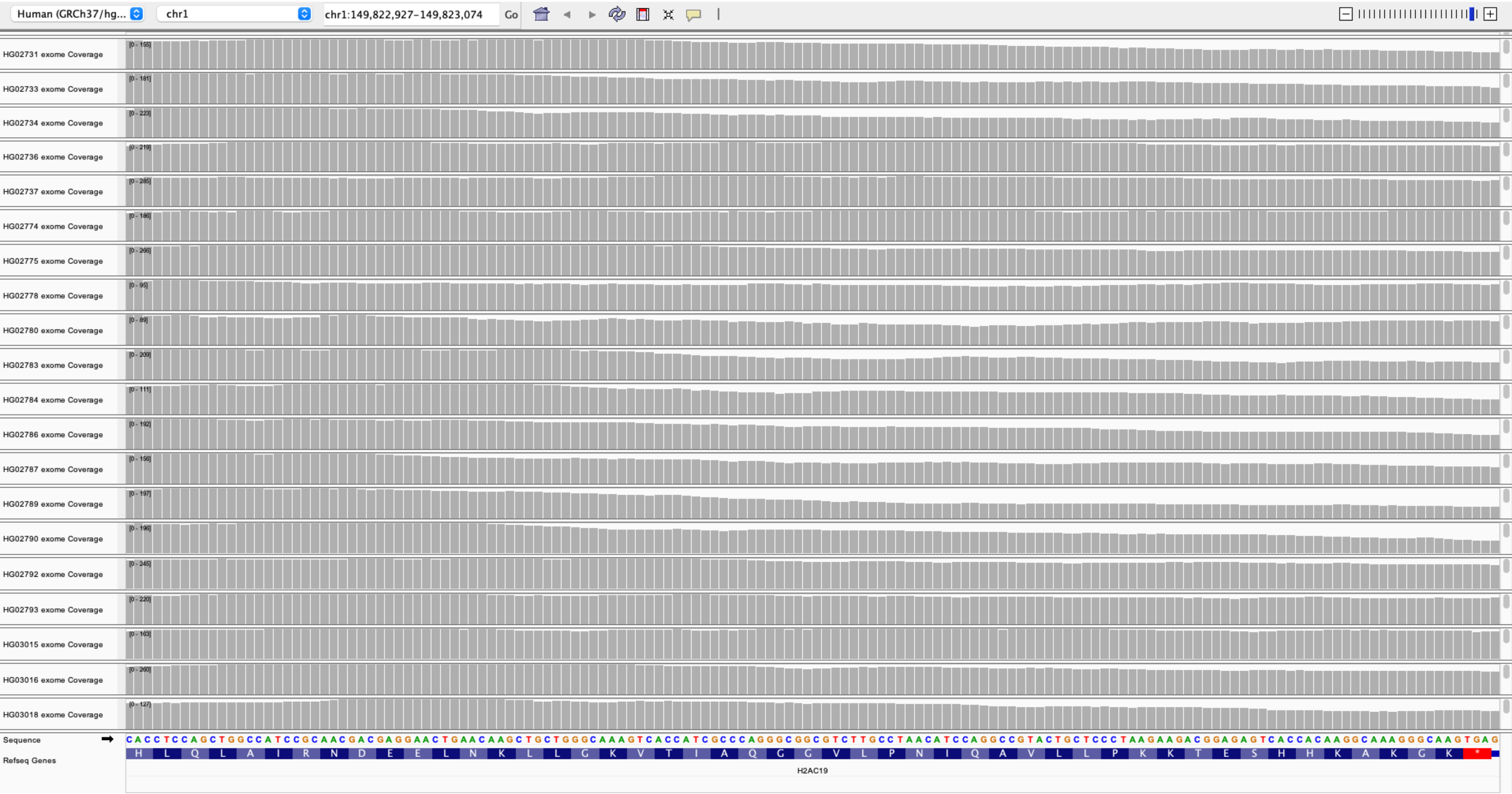

PJL: HG03019-HG03640

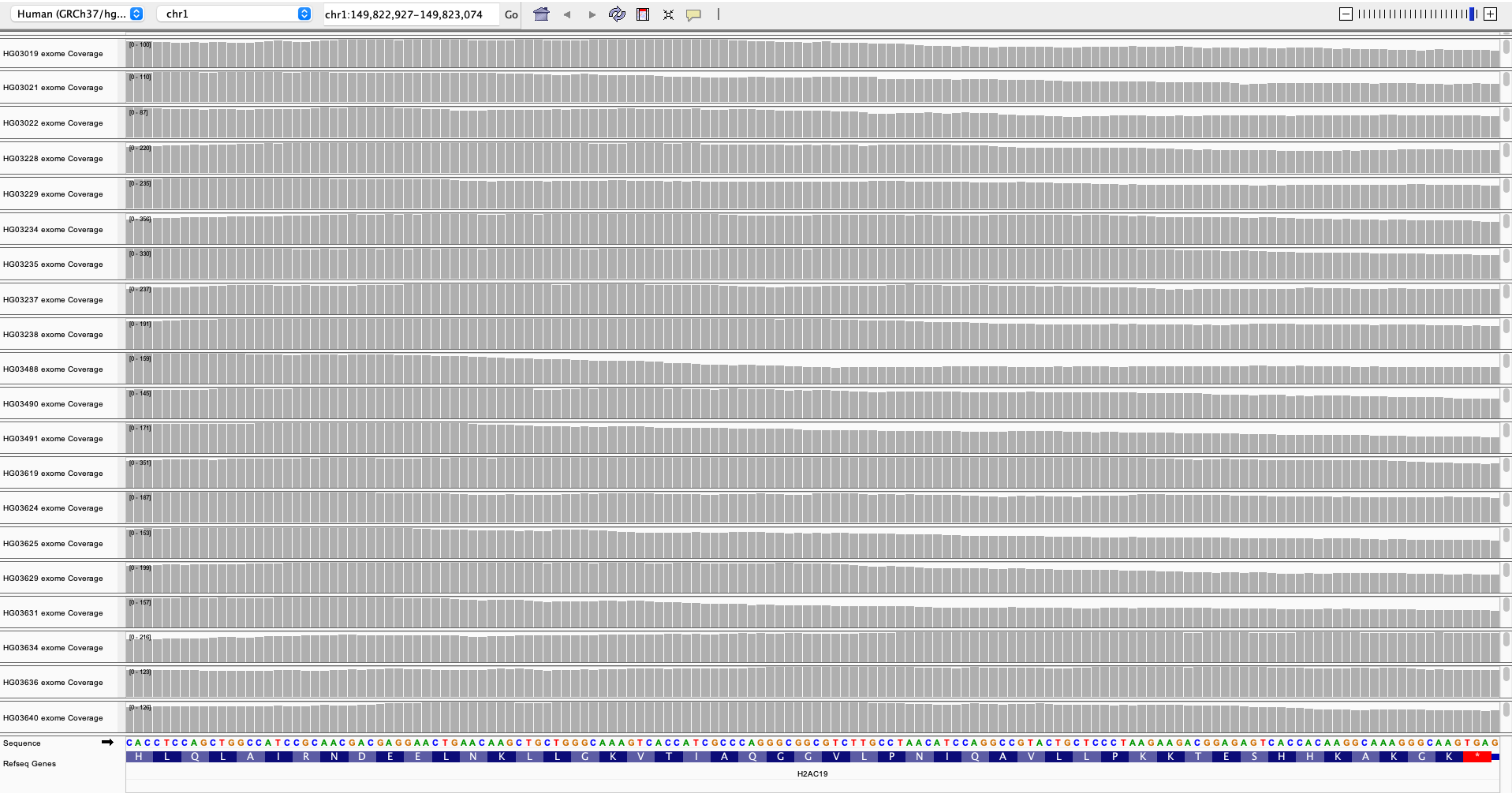

PJL: HG03649-HG03767

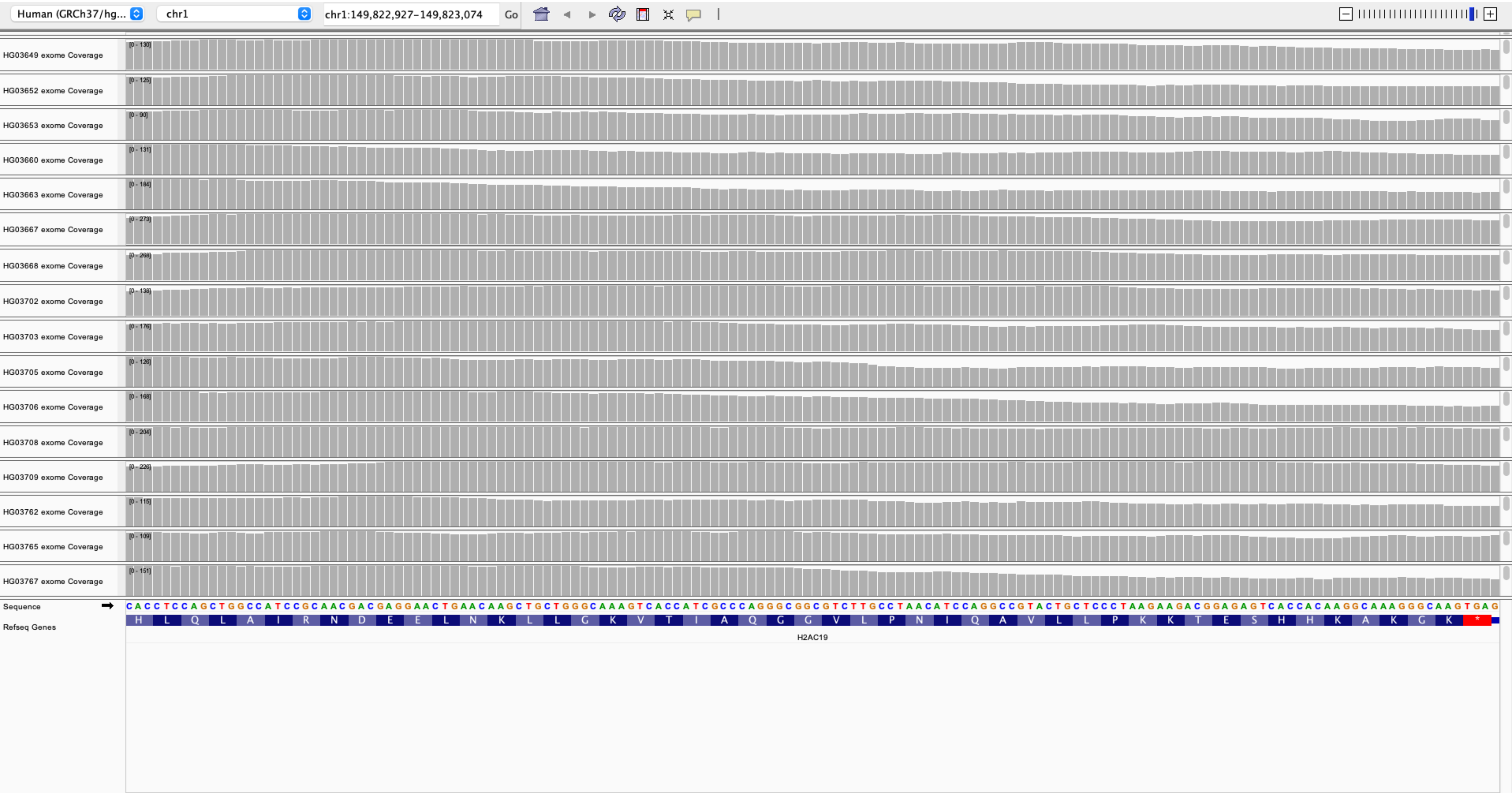

STU: Sri Lankan Tamil in the UK (samples)

STU: HG03642-HG03694

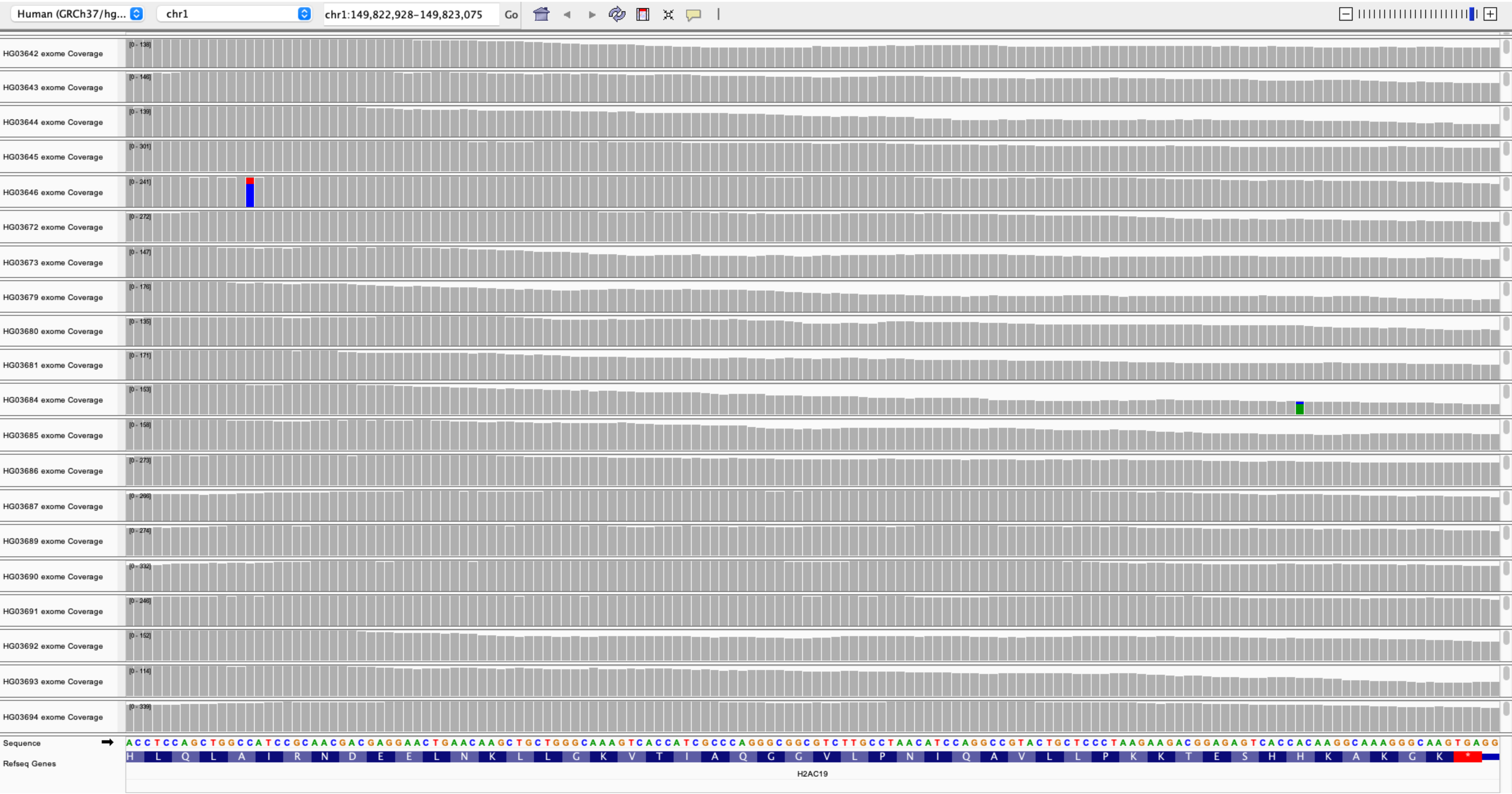

STU: HG03695-HG03756

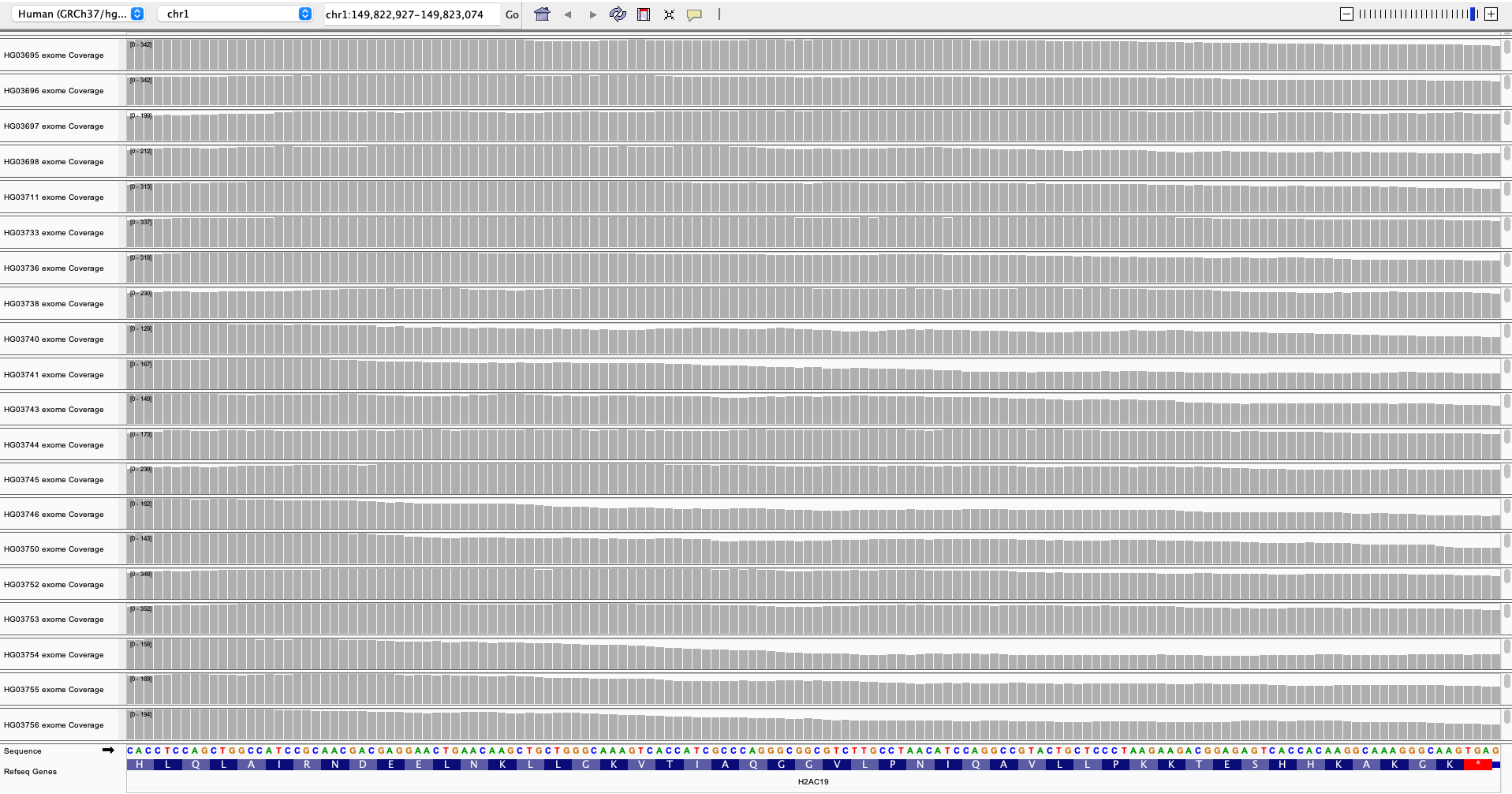

STU: HG03757-HG03888

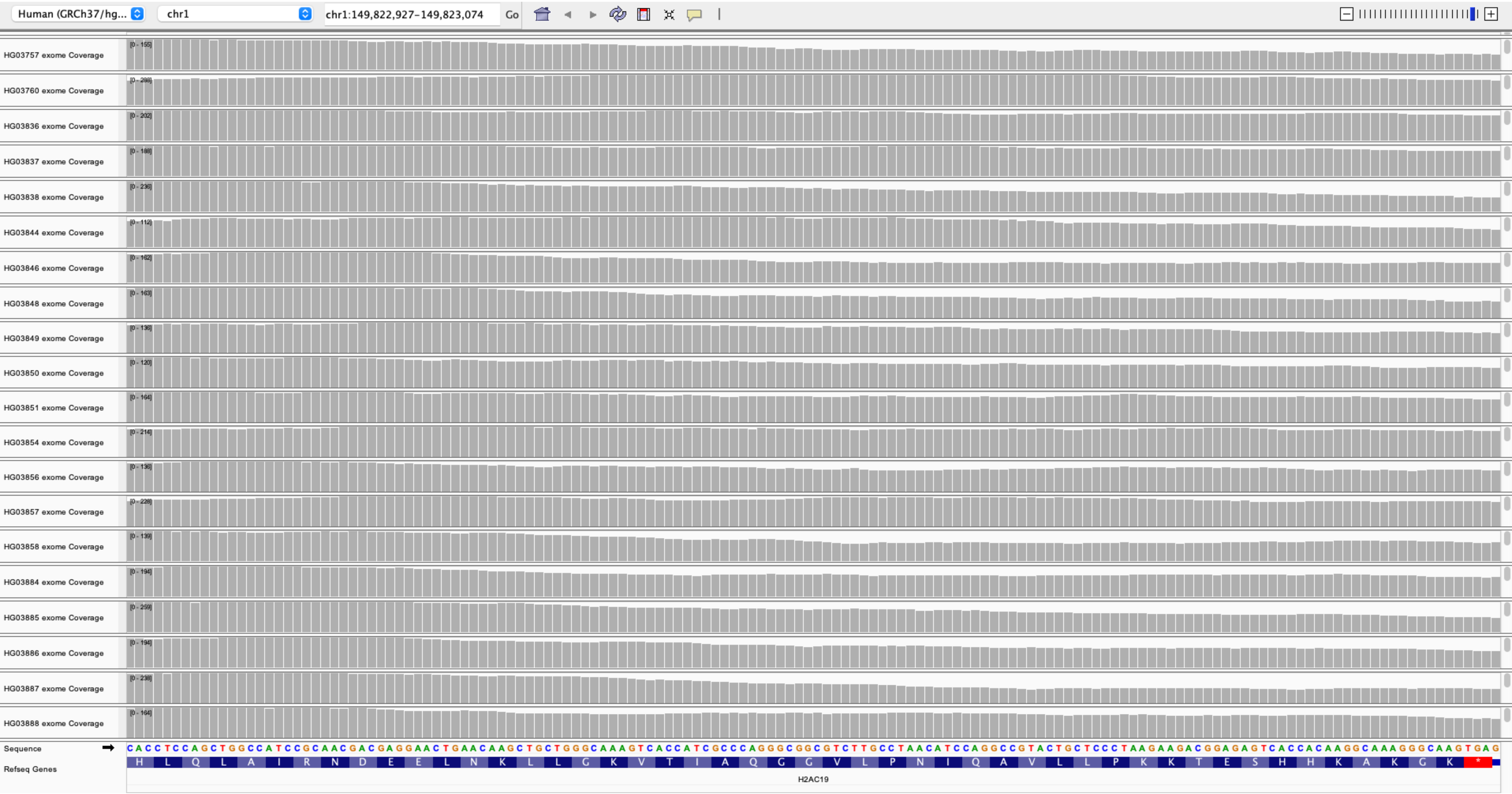

STU: HG03890-HG03986

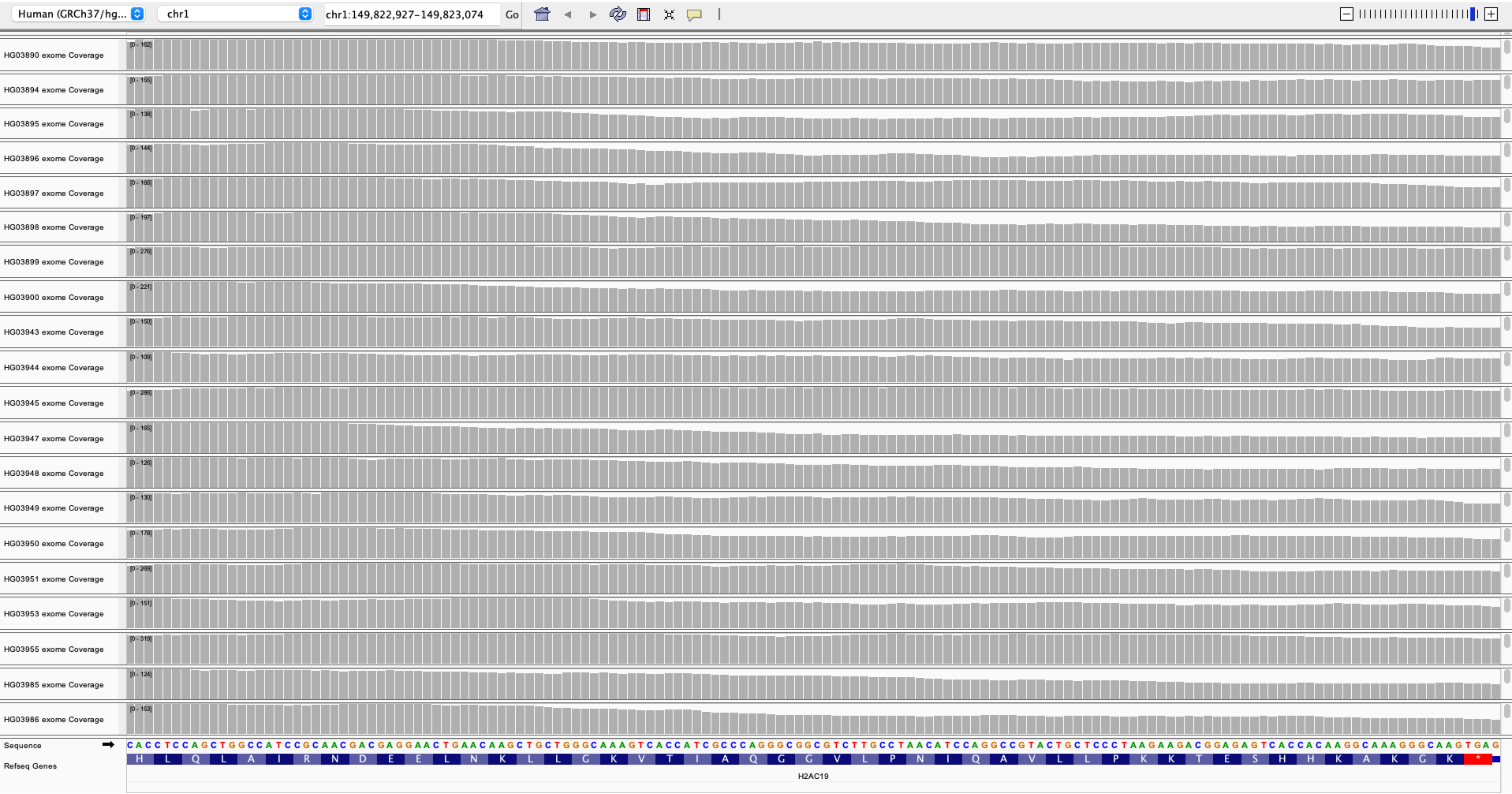

STU: HG03989-HG04107

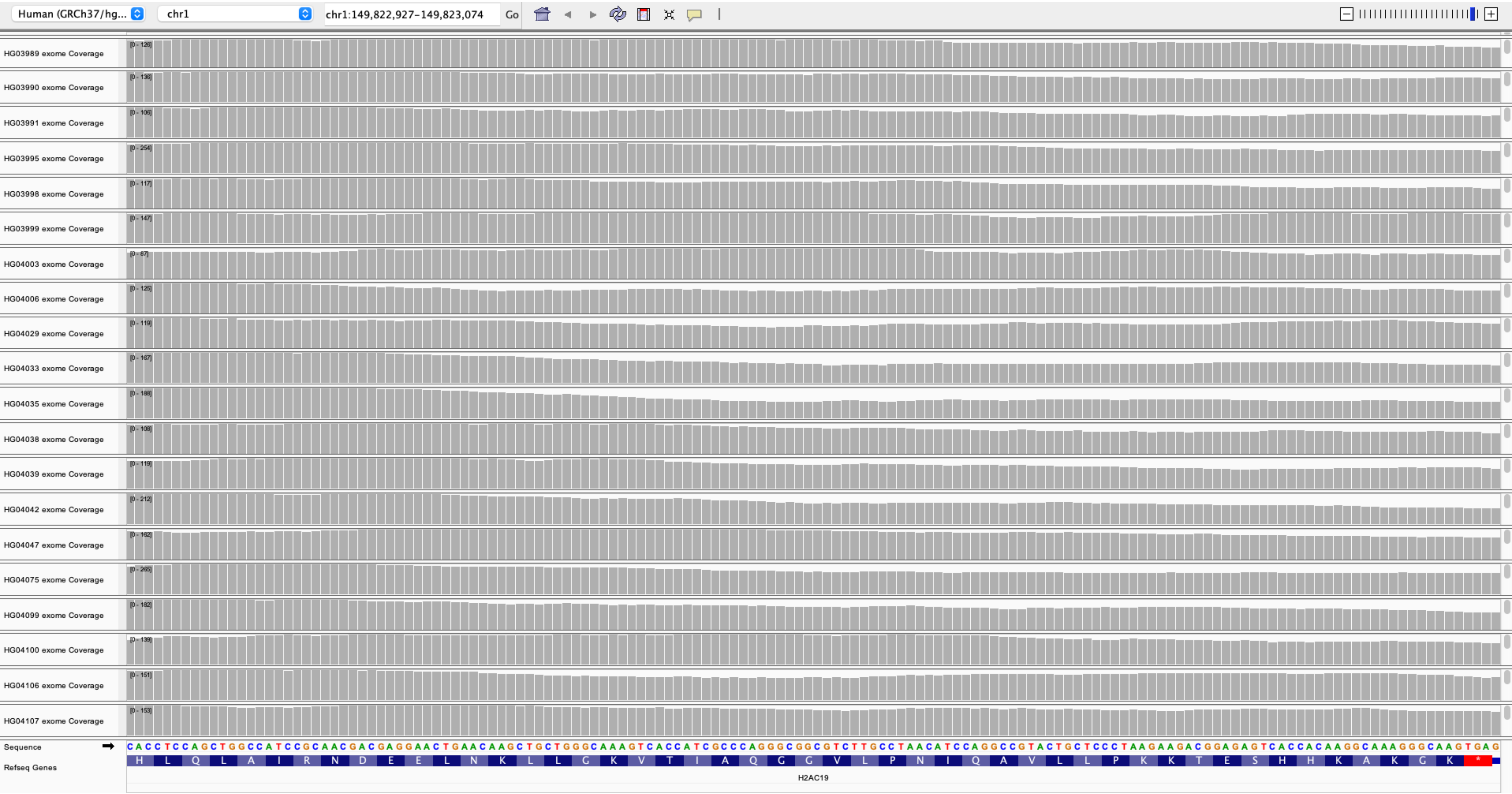

STU: HG04210-HG04229

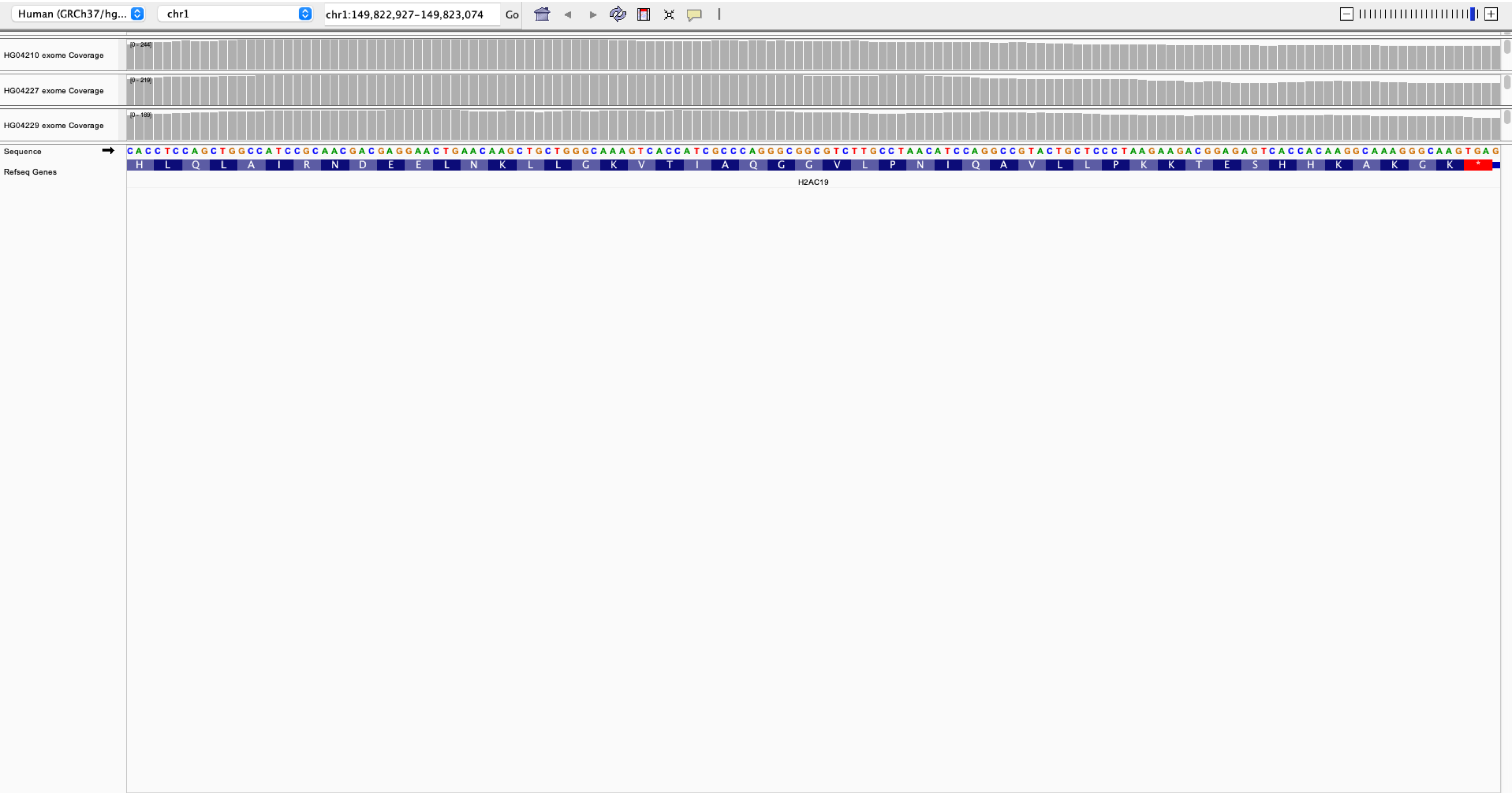

Supplement: Supplementary Figure 2 — Multi-sequence alignment of the c-terminal domain of histone H2A variants. [file Supplementaryfile2.pdf]
