# Supplementary material for: A novel H2A-A127 variant is associated with human cancer and enhances tumor-related phenotypes in Drosophila melanogaster models
Source: Front Oncol. 2026 Jul 13;16:1814908. doi: 10.3389/fonc.2026.1814908 (PMC13402181; doi:10.3389/fonc.2026.1814908)
Supplement: Supplementary Figure 3 — Alignment of human H2AC19 with orthologous H2A proteins from chimpanzee (PANTR; H2AC19), mouse (Hist2h2aa1), Saccharomyces cerevisiae (yeast; HTA1 and HTA2), and Drosophila melanogaster (DROME; His2A). [file Supplementaryfile3.pdf]

Supplementary Figures

Figure S1. Coverage tracks for the 5 AML samples carrying A127V in the validation cohort.

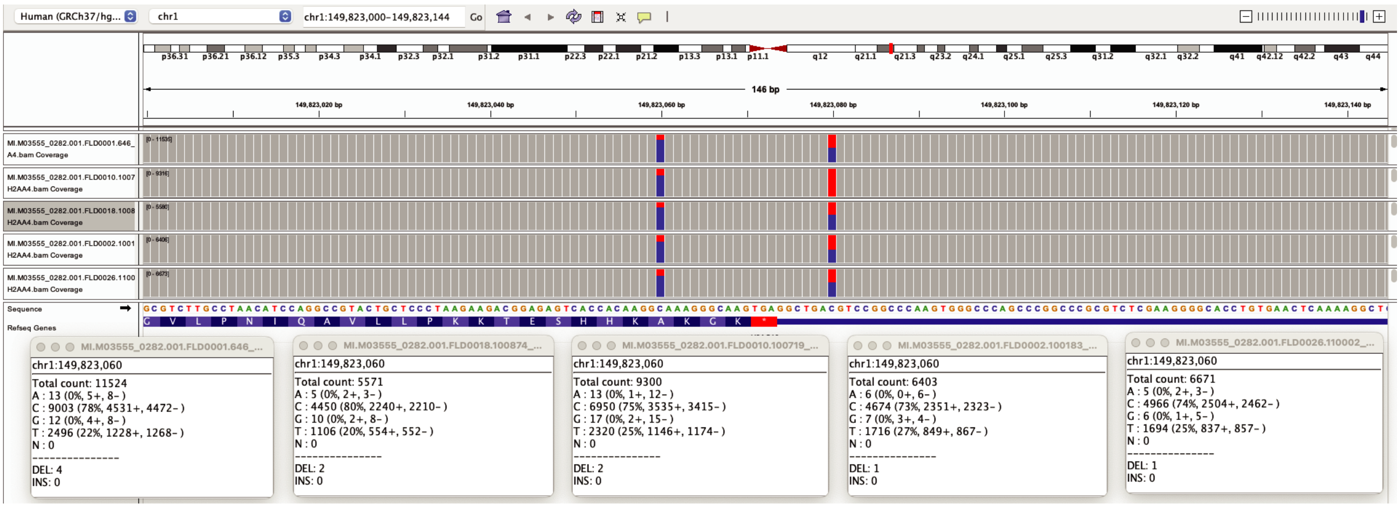

Figure S2. Multi-sequence alignment of the c-terminal domain of histone H2A variants.

```
H2AB1      ----------------------------   115
H2AB2      ----------------------------   115
H2AB3      ----------------------------   115
H2AZ1      LIGKKGQQKTV-----------------   128
H2AZ2      LIGKKGQQKTA-----------------   128
H2AX       LLPKKTSATVGPKAPSGGKKATQASQEY   143
H2AC1      LLPKKTESHHHKAQSK------------   131  H2AC1-A128
H2AC21     LLPKKTESHKPGKNK-------------   130
H2AJ       LLPKKTESQKTKSK--------------   129
H2AC20     LLPKKTESHKAKSK--------------   129  H2AC20-A126
H2AC14     LLPKKTESHHKTK---------------   128
H2AC11     LLPKKTESHHKAKGK-------------   130  H2AC11-A127
H2AC12     LLPKKTESHHKAK---------------   128  H2AC12-A127
H2AC13     LLPKKTESHHKAKGK-------------   130  H2AC13-A127
H2AC15     LLPKKTESHHKAKGK-------------   130  H2AC15-A127
H2AC16     LLPKKTESHHKAKGK-------------   130  H2AC16-A127
H2AC17     LLPKKTESHHKAKGK-------------   130  H2AC17-A127
H2AC4      LLPKKTESHHKAKGK-------------   130  H2AC4-A127
H2AC8      LLPKKTESHHKAKGK-------------   130  H2AC8-A127
H2AC7      LLPKKTESHHKAKGK-------------   130  H2AC7-A127
H2AC19     LLPKKTESHHKAKGK-------------   130  H2AC19-A127
H2AC18     LLPKKTESHHKAKGK-------------   130  H2AC18-A127
H2AC6      LLPKKTESHHKAKGK-------------   130  H2AC6-A127
H2AW       LLPKKTESHHKAKGK-------------   130  H2AW-A127
```

Figure S3.

```
HTA1_YEAST             MSGGKGGKAGSAAKASQSRSAKAGLTFPVGRVHRLLRRGNYAQRIGSGAPVYLTAVLEYL     60
HTA2_YEAST             MSGGKGGKAGSAAKASQSRSAKAGLTFPVGRVHRLLRRGNYAQRIGSGAPVYLTAVLEYL     60
H2AC19_HUMAN           -MSGRGKQGGKARAKAKSRSSRAGLQFPVGRVHRLLRKGNYAERVGAGAPVYMAAVLEYL     59
H2AC19_PANTR           -MSGRGKQGGKARAKAKSRSSRAGLQFPVGRVHRLLRKGNYAERVGAGAPVYMAAVLEYL     59
Hist2h2aa1_MOUSE       -MSGRGKQGGKARAKAKSRSSRAGLQFPVGRVHRLLRKGNYAERVGAGAPVYMAAVLEYL     59
His2A_DROME            -MSGRGK-GGKVKGKAKSRSNRAGLQFPVGRIHRLLRKGNYAERVGAGAPVYLAAVMEYL     58
                         .*:*  .*..   ::*** :*** *****:*****:****:*:*:*****::**:***

HTA1_YEAST             AAEILELAGNAARDNKKTRIIPRHLQLAIRNDDELNKLLGNVTIAQGGVLPNIHQNLLPK     120
HTA2_YEAST             AAEILELAGNAARDNKKTRIIPRHLQLAIRNDDELNKLLGNVTIAQGGVLPNIHQNLLPK     120
H2AC19_HUMAN           TAEILELAGNAARDNKKTRIIPRHLQLAIRNDEELNKLLGKVTIAQGGVLPNIQAVLLPK     119
H2AC19_PANTR           TAEILELAGNAARDNKKTRIIPRHLQLAIRNDEELNKLLGKVTIAQGGVLPNIQAVLLPK     119
Hist2h2aa1_MOUSE       TAEILELAGNAARDNKKTRIIPRHLQLAIRNDEELNKLLGKVTIAQGGVLPNIQAVLLPK     119
His2A_DROME            AAEVLELAGNAARDNKKTRIIPRHLQLAIRNDEELNKLLSGVTIAQGGVLPNIQAVLLPK     118
                       :**:****************************:******. ************:  ****

HTA1_YEAST             KSAKATKASQEL      132
HTA2_YEAST             KSAKTAKASQEL      132
H2AC19_HUMAN           KTESHHKAKGK-      130
H2AC19_PANTR           KTESHHKAKGK-      130
Hist2h2aa1_MOUSE       KTESHHKAKGK-      130
His2A_DROME            KTEKKA------      124
                       *: .
```

Figure S4.

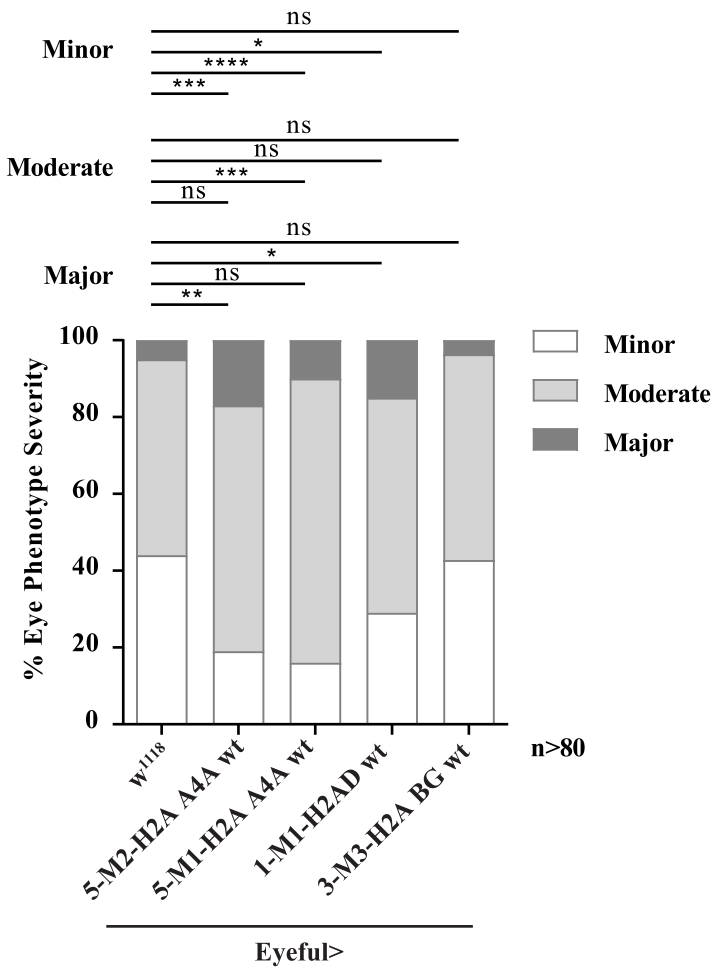

Figure S5.

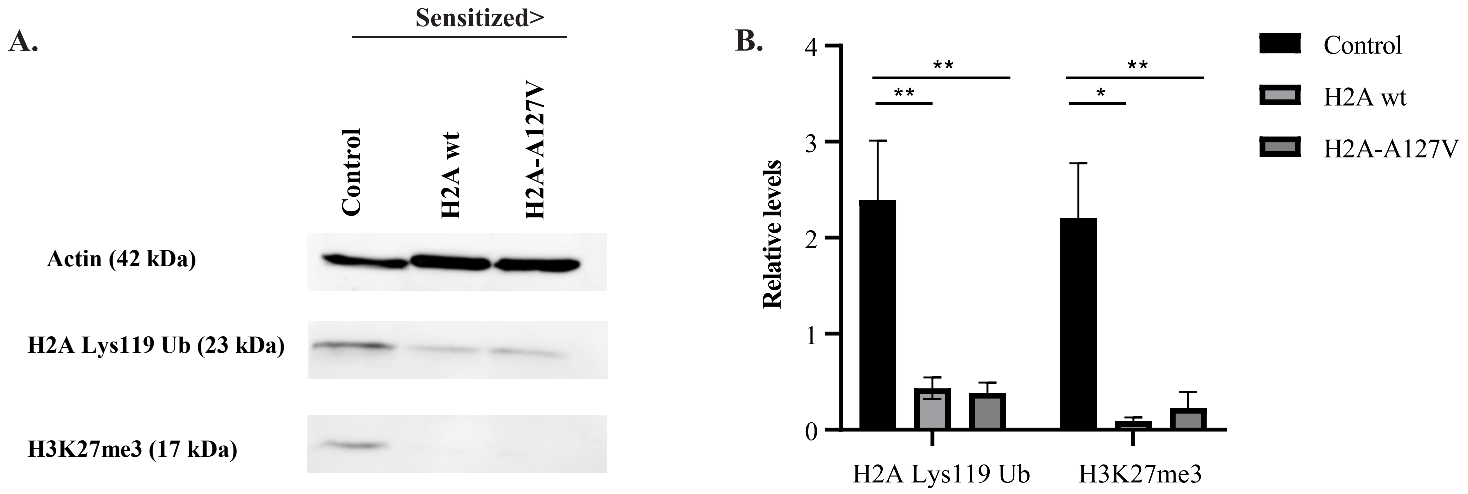

Figure S6.1

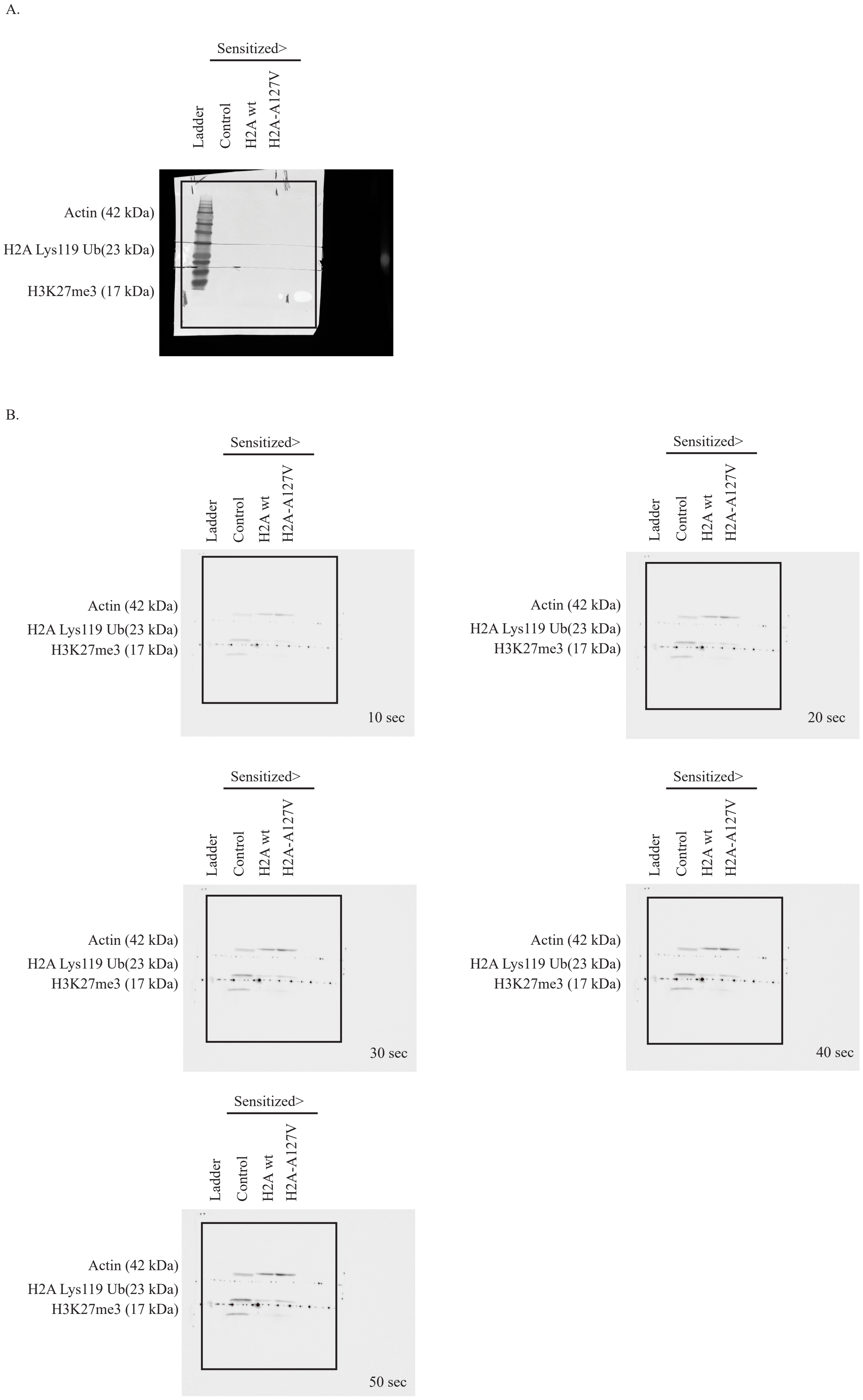
A.
Sensitized>
Ladder
Control
H2A wt
H2A-A127V
Actin (42 kDa)
H2A Lys119 Ub(23 kDa)
H3K27me3 (17 kDa)
B.
Sensitized>
Ladder
Control
H2A wt
H2A-A127V
Actin (42 kDa)
H2A Lys119 Ub(23 kDa)
H3K27me3 (17 kDa)
10 sec
Sensitized>
Ladder
Control
H2A wt
H2A-A127V
Actin (42 kDa)
H2A Lys119 Ub(23 kDa)
H3K27me3 (17 kDa)
20 sec
Sensitized>
Ladder
Control
H2A wt
H2A-A127V
Actin (42 kDa)
H2A Lys119 Ub(23 kDa)
H3K27me3 (17 kDa)
30 sec
Sensitized>
Ladder
Control
H2A wt
H2A-A127V
Actin (42 kDa)
H2A Lys119 Ub(23 kDa)
H3K27me3 (17 kDa)
40 sec
Sensitized>
Ladder
Control
H2A wt
H2A-A127V
Actin (42 kDa)
H2A Lys119 Ub(23 kDa)
H3K27me3 (17 kDa)
50 sec

Figure S6.2

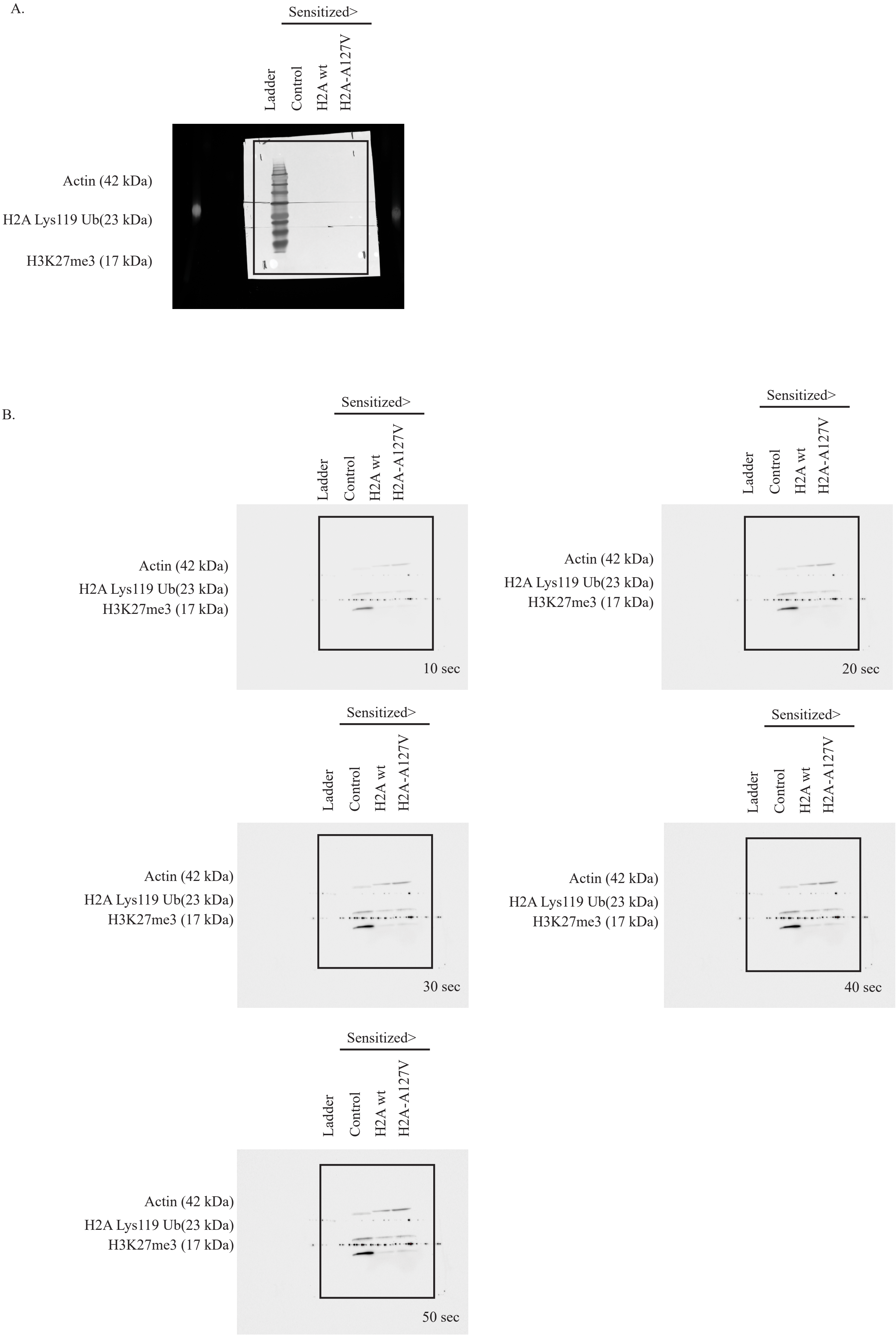
A.
Sensitized>
Ladder
Control
H2A wt
H2A-A127V
Actin (42 kDa)
H2A Lys119 Ub(23 kDa)
H3K27me3 (17 kDa)
B.
Sensitized>
Ladder
Control
H2A wt
H2A-A127V
Actin (42 kDa)
H2A Lys119 Ub(23 kDa)
H3K27me3 (17 kDa)
10 sec
20 sec
30 sec
40 sec
50 sec

Figure S6.3

A.

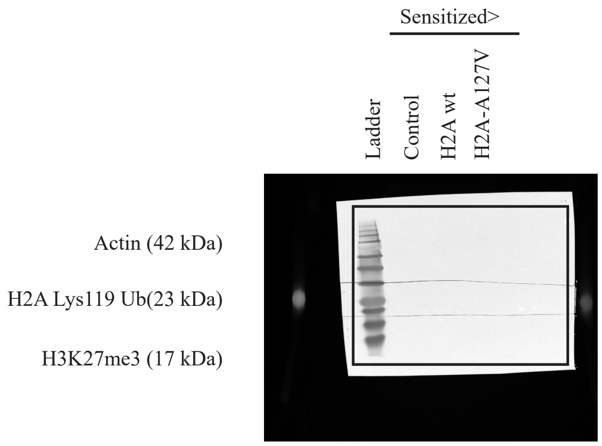

B.

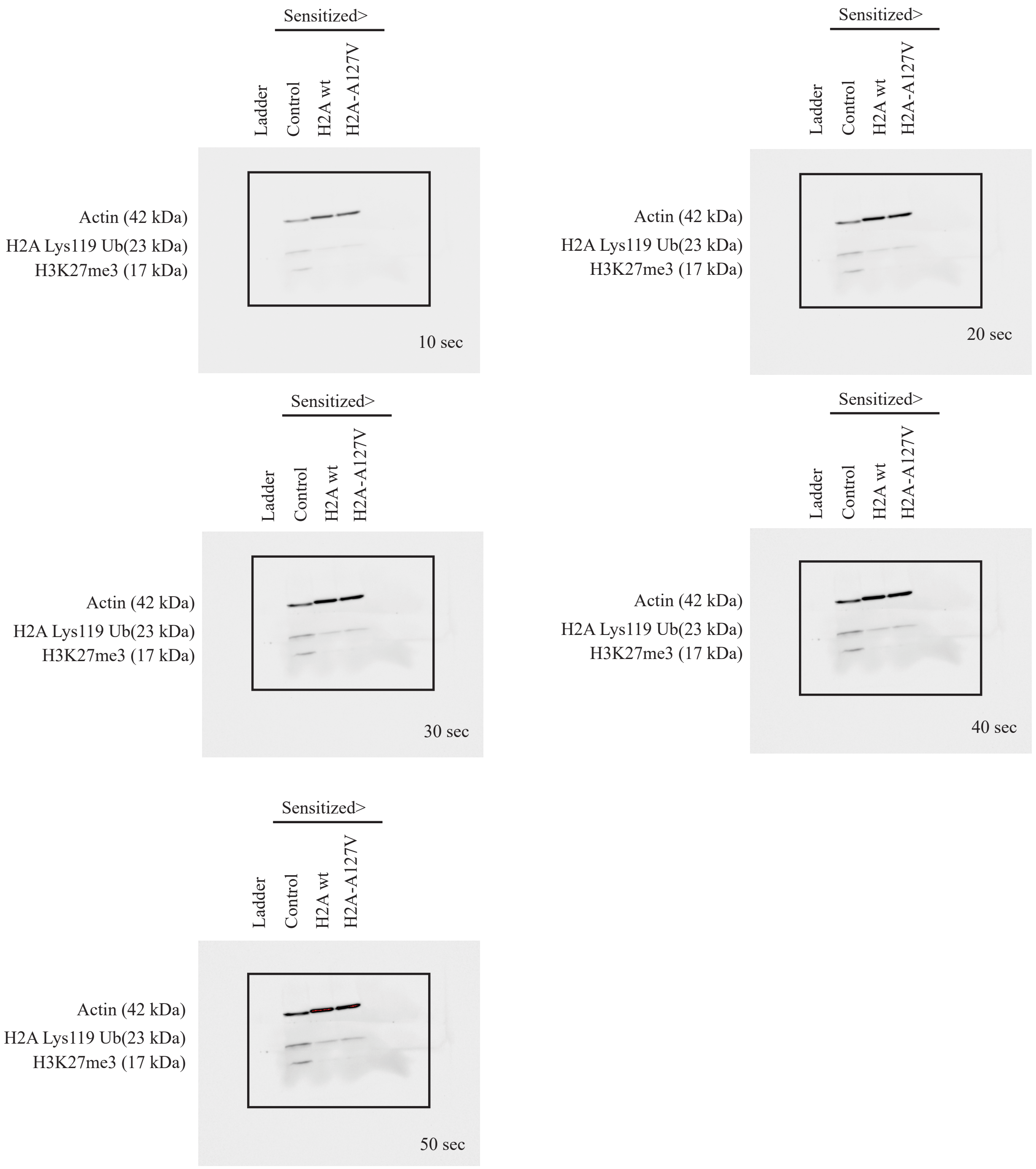

Figure S7.

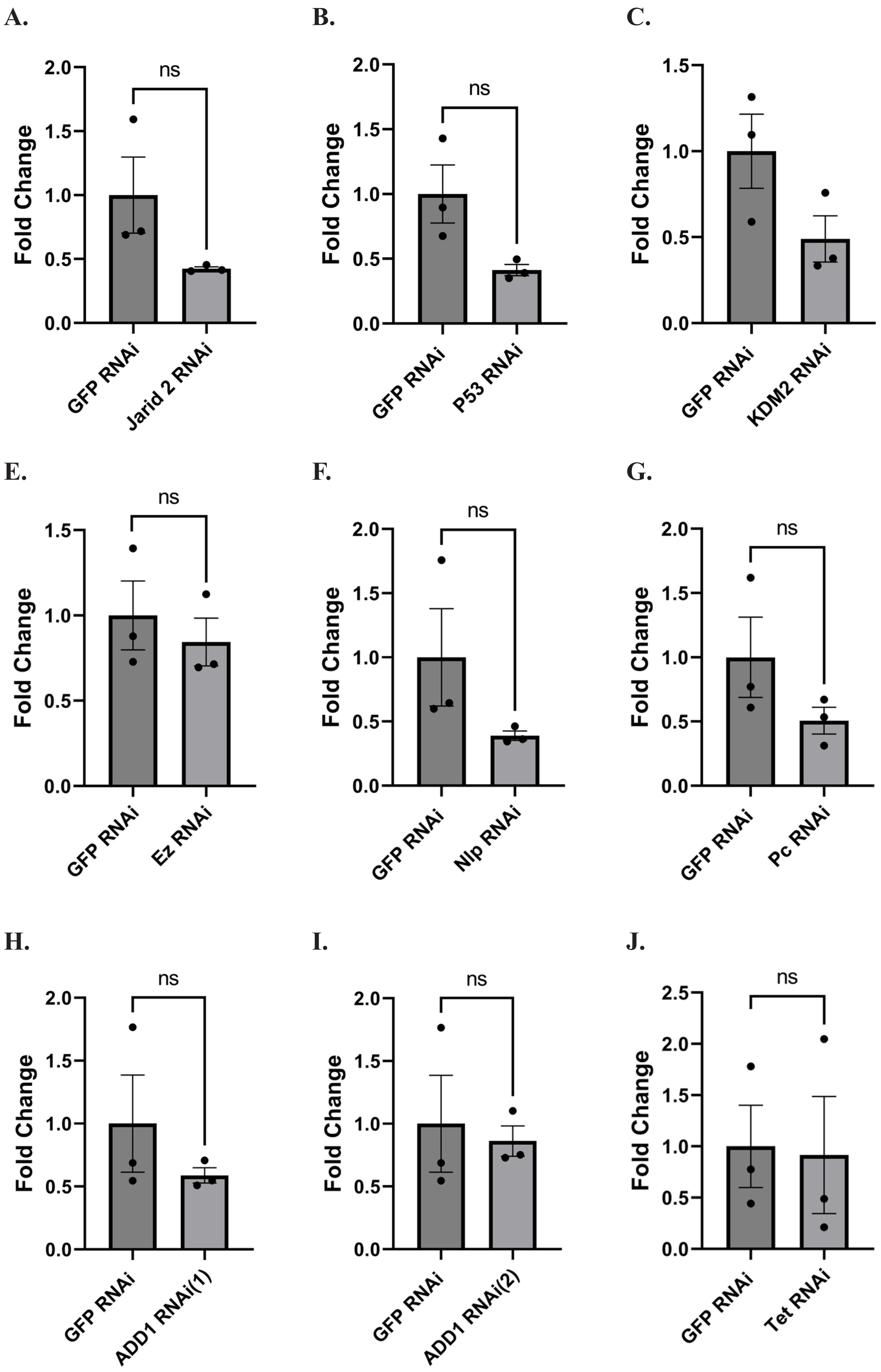
A.
ns
Fold Change
2.0
1.5
1.0
0.5
0.0
GFP RNAi
Jarid 2 RNAi
B.
ns
Fold Change
GFP RNAi
P53 RNAi
C.
Fold Change
1.5
1.0
0.5
0.0
GFP RNAi
KDM2 RNAi
E.
ns
Fold Change
GFP RNAi
Ez RNAi
F.
ns
Fold Change
GFP RNAi
Nlp RNAi
G.
ns
Fold Change
GFP RNAi
Pc RNAi
H.
ns
Fold Change
GFP RNAi
ADD1 RNAi(1)
I.
ns
Fold Change
GFP RNAi
ADD1 RNAi(2)
J.
ns
Fold Change
2.5
2.0
1.5
1.0
0.5
0.0
GFP RNAi
Tet RNAi
