# Supplementary material for: A novel H2A-A127 variant is associated with human cancer and enhances tumor-related phenotypes in Drosophila melanogaster models
Source: Front Oncol. 2026 Jul 13;16:1814908. doi: 10.3389/fonc.2026.1814908 (PMC13402181; doi:10.3389/fonc.2026.1814908)
Supplement: Supplementary Figure 4 — Scoring the severity of eye phenotype (%) for different H2A wt lines under the control of eyeful driver, at 25 °C (***, p-value <0.001 and ****, p-value <0.0001; chi-square test). 5-M2-H2A A4A wt and 5-M1-H2A A4A wt (the H2A wt line used in this study) are different batches of transgenic lines sent by the company, having the H2A inserted in the same locus. 1-M1-H2AD wt and 3-M3-H2A BG wt are transgenic flies of other H2A variants. The same scoring system as Figure 1 was used: minor (enlarged eyes with no folds), moderate (1–4 folds), and major (>4 folds) severity. Flies were collected from more than 3 biological replicates. [file Supplementaryfile4.pdf]

**Table S1**

These statistical analyses were performed using GraphPad Prism 8 program. P-values lower than 0.05 were considered statistically significant.

| Figure Panel | Statistical Test | Comparison | P-value |
|---|---|---|---|
| Figure 1B | Chi-square test | Minor: Control vs. H2A wt | 0.0067 |
| Figure 1B | Chi-square test | Minor: Control vs. H2A-A127V | <0.0001 |
| Figure 1B | Chi-square test | Minor: H2A wt vs. H2A-A127V | 0.1497 |
| Figure 1B | Chi-square test | Moderate: Control vs. H2A wt | 0.0157 |
| Figure 1B | Chi-square test | Moderate: Control vs. H2A-A127V | <0.0001 |
| Figure 1B | Chi-square test | Moderate: H2A wt vs. H2A-A127V | 0.0110 |
| Figure 1B | Chi-square test | Major: Control vs. H2A wt | <0.0001 |
| Figure 1B | Chi-square test | Major: Control vs. H2A-A127V | <0.0001 |
| Figure 1B | Chi-square test | Major: H2A wt vs. H2A-A127V | 0.0005 |
| Figure 1D | Chi-square test | Metastasis: Control vs. H2A wt | 0.2060 |
| Figure 1D | Chi-square test | Metastasis: Control vs. H2A-A127V | 0.0311 |
| Figure 1D | Chi-square test | Metastasis: H2A wt vs. H2A-A127V | 0.0070 |
| Figure 2B | Chi-square test | Eye folds phenotype, ADD1 RNAi (1): Control RNAi vs. H2A wt | <0.0001 |
| Figure 2B | Chi-square test | Eye folds phenotype, ADD1 RNAi (1): Control RNAi vs. H2A-A127V | <0.0001 |
| Figure 2B | Chi-square test | Eye folds phenotype, ADD1 RNAi (1): H2A wt vs. H2A-A127V | 0.0474 |
| Figure 2B | Chi-square test | Eye folds phenotype, ADD1 RNAi (2): Control RNAi vs. H2A wt | 0.1071 |
| Figure 2B | Chi-square test | Eye folds phenotype, ADD1 RNAi (2): Control RNAi vs. H2A-A127V | 0.3168 |
| Figure 2B | Chi-square test | Eye folds phenotype, ADD1 RNAi (2): H2A wt vs. H2A-A127V | 0.5064 |
| Figure 2B | Chi-square test | Eye folds phenotype, Asx RNAi: Control RNAi vs. H2A wt | 0.2313 |
| Figure 2B | Chi-square test | Eye folds phenotype, Asx RNAi: Control RNAi vs. H2A-A127V | 0.1968 |
| Figure 2B | Chi-square test | Eye folds phenotype, Asx RNAi: H2A wt vs. H2A-A127V | 0.9060 |
| Figure 2B | Chi-square test | Eye folds phenotype, dTET RNAi: Control RNAi vs. H2A wt | 0.7158 |
| Figure 2B | Chi-square test | Eye folds phenotype, dTET RNAi: Control RNAi vs. H2A-A127V | 0.0731 |
| Figure 2B | Chi-square test | Eye folds phenotype, dTET RNAi: H2A wt vs. H2A-A127V | 0.1382 |
| Figure 2B | Chi-square test | Eye folds phenotype, lozenge RNAi: Control RNAi vs. H2A wt | 0.0348 |
| Figure 2B | Chi-square test | Eye folds phenotype, lozenge RNAi: Control RNAi vs. H2A-A127V | 0.0099 |

| | | | |
|---|---|---|---|
| Figure 2B | Chi-square test | Eye folds phenotype, lozenge RNAi: H2A wt vs. H2A-A127V | 0.6429 |
| Figure 2B | Chi-square test | Eye folds phenotype, Nlp RNAi: Control RNAi vs. H2A wt | 0.9901 |
| Figure 2B | Chi-square test | Eye folds phenotype, Nlp RNAi: Control RNAi vs. H2A-A127V | 0.6153 |
| Figure 2B | Chi-square test | Eye folds phenotype, Nlp RNAi: H2A wt vs. H2A-A127V | 0.6066 |
| Figure 2B | Chi-square test | Eye folds phenotype, p53 RNAi: Control RNAi vs. H2A wt | 0.1308 |
| Figure 2B | Chi-square test | Eye folds phenotype, p53 RNAi: Control RNAi vs. H2A-A127V | 0.5527 |
| Figure 2B | Chi-square test | Eye folds phenotype, p53 RNAi: H2A wt vs. H2A-A127V | 0.3552 |
| Figure 2B | Chi-square test | Eye folds phenotype, GFP RNAi: Control RNAi vs. H2A wt | <0.0001 |
| Figure 2B | Chi-square test | Eye folds phenotype, GFP RNAi: Control RNAi vs. H2A-A127V | 0.0352 |
| Figure 2B | Chi-square test | Eye folds phenotype, GFP RNAi: H2A wt vs. H2A-A127V | 0.0167 |
| Figure 2D | Chi-square test | Eye folds phenotype, KDM2 RNAi: Control RNAi vs. H2A wt | <0.0001 |
| Figure 2D | Chi-square test | Eye folds phenotype, KDM2 RNAi: Control RNAi vs. H2A-A127V | <0.0001 |
| Figure 2D | Chi-square test | Eye folds phenotype, KDM2 RNAi: H2A wt vs. H2A-A127V | 0.7790 |
| Figure 2D | Chi-square test | Eye folds phenotype, Pc RNAi: Control RNAi vs. H2A wt | <0.0001 |
| Figure 2D | Chi-square test | Eye folds phenotype, Pc RNAi: Control RNAi vs. H2A-A127V | 0.0154 |
| Figure 2D | Chi-square test | Eye folds phenotype, Pc RNAi: H2A wt vs. H2A-A127V | 0.0118 |
| Figure 2D | Chi-square test | Eye folds phenotype, Ez RNAi: Control RNAi vs. H2A wt | 0.0014 |
| Figure 2D | Chi-square test | Eye folds phenotype, Ez RNAi: Control RNAi vs. H2A-A127V | 0.0021 |
| Figure 2D | Chi-square test | Eye folds phenotype, Ez RNAi: H2A wt vs. H2A-A127V | 0.8759 |
| Figure 2D | Chi-square test | Eye folds phenotype, Jarid 2 RNAi: Control RNAi vs. H2A wt | <0.0001 |
| Figure 2D | Chi-square test | Eye folds phenotype, Jarid 2 RNAi: Control RNAi vs. H2A-A127V | <0.0001 |
| Figure 2D | Chi-square test | Eye folds phenotype, Jarid 2 RNAi: H2A wt vs. H2A-A127V | 0.0051 |
| Figure 2D | Chi-square test | Eye folds phenotype, Su (z) 12 RNAi: Control RNAi vs. H2A wt | 0.0102 |
| Figure 2D | Chi-square test | Eye folds phenotype, Su (z) 12 RNAi: Control RNAi vs. H2A-A127V | 0.0068 |
| Figure 2D | Chi-square test | Eye folds phenotype, Su (z) 12 RNAi: H2A wt vs. H2A-A127V | 0.9688 |
| Figure 3A | Chi-square test | Minor Growth: Ez RNAi vs. H2A wt+Ez RNAi | <0.0001 |
| Figure 3A | Chi-square test | Minor Growth: Ez RNAi vs. H2A-A127V+Ez RNAi | <0.0001 |
| Figure 3A | Chi-square test | Minor Growth: H2A wt+Ez RNAi vs. H2A-A127V+Ez RNAi | 0.0018 |
| Figure 3A | Chi-square test | Major Growth: Ez RNAi vs. H2A wt+Ez RNAi | <0.0001 |

| | | | |
|---|---|---|---|
| Figure 3A | Chi-square test | Major Growth: Ez RNAi vs. H2A-A127V+Ez RNAi | <0.0001 |
| Figure 3A | Chi-square test | Major Growth: H2A wt+Ez RNAi vs. H2A-A127V+Ez RNAi | 0.2432 |
| Figure 3B | Chi-square test | Ectopic Growth: Ez RNAi vs. H2A wt+Ez RNAi | 0.3161 |
| Figure 3B | Chi-square test | Ectopic Growth: Ez RNAi vs. H2A-A127V+Ez RNAi | 0.0045 |
| Figure 3B | Chi-square test | Ectopic Growth: H2A wt+Ez RNAi vs. H2A-A127V+Ez RNAi | 0.0192 |

**Table S1. Table summarizing the P-values of all comparisons from the eyeful, AML and PRC1/2 screens.**

## Table S2

| Parameter | parameter | Leucegene Total | H2A-WT | H2A-A127V | Fisher p-value |
|---|---|---|---|---|---|
| **Cytogenetic risk** | Favorable | 60 (19.6%) | 56 (19.4%) | 4 (22.2%) | 0.626 |
| | Intermediate | 150 (49%) | 143 (49.7%) | 7 (38.9%) | |
| | Adverse | 96 (31.4%) | 89 (30.9%) | 7 (38.9%) | |
| **Karyotype** | Normal Karyotype | 68 (25.4%) | 63 (25%) | 5 (31.2%) | 0.562 |
| | Abnormal Karyotype | 200 (74.6%) | 189 (75%) | 11 (68.8%) | |
| **Complex Karyotype** | No | 199 (74.3%) | 190 (75.4%) | 9 (56.2%) | 0.135 |
| | Yes | 69 (25.7%) | 62 (24.6%) | 7 (43.8%) | |
| **TP53 status** | TP53 wt | 167 (79.1%) | 156 (79.2%) | 11 (78.6%) | 1 |
| | TP53 mt | 44 (20.9%) | 41 (20.8%) | 3 (21.4%) | |
| **FLT3-ITD** | No | 298 (68.2%) | 284 (68.4%) | 14 (63.6%) | 0.643 |
| | Yes | 139 (31.8%) | 131 (31.6%) | 8 (36.4%) | |
| **IDH1/2 status** | IDH1/2 wt | 359 (82.2%) | 339 (81.7%) | 20 (90.9%) | 0.394 |
| | IDH1/2 mt | 78 (17.8%) | 76 (18.3%) | 2 (9.09%) | |
| **NPM1 status** | NPM1 wt | 298 (68.2%) | 284 (68.4%) | 14 (63.6%) | 0.643 |
| | NPM1 mt | 139 (31.8%) | 131 (31.6%) | 8 (36.4%) | |

**Table S2. Cytogenetic and mutational characteristics of H2A-A127V and H2A-wt AML samples in the Leucegene dataset.**

## Table S3

| Population | Number of samples | Number of samples with H2AC18/19-A127V |
|---|---|---|
| **African ancestry** | | |
| ACB: African Caribbean in Barbados | 96 | 0 |
| ASW: People with African Ancestry in Southwest USA | 66 | 0 |
| ESN: Esan in Nigeria | 99 | 0 |
| GWD: Gambian in Western Division, Mandinka | 113 | 1 |
| LWK: Luhya in Webuye, Kenya | 101 | 0 |
| MSL: Mende in Sierra Leone | 85 | 1 |
| YRI: Yoruba in Ibadan, Nigeria | 109 | 0 |
| **Americas** | | |
| CLM: Colombians in Medellin, Colombia | 94 | 1 |
| MXL: People with Mexican Ancestry in Los Angeles, CA, USA | 97 | 1 |
| PEL: Peruvians in Lima, Peru | 86 | 1 |
| PUR: Puerto Ricans in Puerto Rico | 105 | 2 |
| **East Asian ancestry** | | |
| CDX: Chinese Dai in Xishuangbanna, China | 99 | 0 |
| CHB: Han Chinese in Beijing, China | 103 | 0 |
| CHS: Southern Han Chinese | 108 | 0 |
| KHV: Kinh in Ho Chi Minh City, Vietnam | 101 | 0 |
| JPT: Japanese in Tokyo, Japan | 104 | 0 |
| **South Asian ancestry** | | |
| BEB: Bengali in Bangladesh | 86 | 0 |
| GIH: Gujarati Indians in Houston, TX, USA | 106 | 1 |
| ITU: Indian Telugu in the UK | 103 | 0 |
| PJL: Punjabi in Lahore, Pakistan | 96 | 1 |

| | | |
|---|---|---|
| STU: Sri Lankan Tamil in the UK | 103 | 0 |
| **European ancestry** | | |
| CEU: Utah residents (CEPH) with Northern and Western European ancestry | 99 | 7 |
| FIN: Finnish in Finland | 99 | 4 |
| GBR: British in England and Scotland | 92 | 4 |
| IBS: Iberian Populations in Spain | 107 | 9 |
| TSI: Toscani in Italia | 108 | 4 |
| **Total** | 2565 | 37 |

**Table S3. Table illustrating the enrichment of H2AC18/19-A127V mutation in several populations using the 1000 Genomes Project.**

# Table S4 + S5

| Human genes frequently mutated in AML | Fly orthologue | Function | References |
|---|---|---|---|
| Alpha-thalassemia/mental retardation syndrome X-linked (ATRX) | ADD domain-containing protein 1 (ADD1) | Heterochromatin Protein 1 interactor that interacts with methylated H3K9 | PMID: 37464424<br>PMID: 33838631<br>PMID: 21067377 |
| Additional Sex Comb-Like 1 (ASXL1) | Polycomb protein Asx (Asx) | Chromatin binding protein with deubiquitination activity of the product of His2A. | PMID: 22058207 |
| Ten–Eleven Translocation 2 (TET2) | Ten-Eleven Translocation (TET) family protein (dTet) | Dioxygenase that demethylates DNA methylated in the 6th position of adenine (N(6)-methyladenosine) DNA and catalyzes the conversion of the modified genomic base 5methylcytosine (5mC) into 5hydroxymethylcytosine (5hmC) | PMID: 22430270 |
| Runt-Related Transcription Factor (RUNX1) | lozenge (RUNX2,3) | Alpha-subunit of the transcription factor complex core binding factor, which is involved in transcription regulation. | PMID: 21343560 |
| NPM1 nucleophosmin/nucleoplasmin 1 | Nucleoplasmin NLP | Binds to core histones and functions in the ATP-facilitated assembly of approximately regularly spaced nucleosomal arrays. | PMID: 23436734 |
| Tumor Protein p53 (TP53) | p53 | Transcriptional factor required for adaptive responses to genotoxic stress, including cell death, compensatory proliferation, and DNA repair | PMID: 37835510 |

**Table S4. Table summarizing highly conserved human genes that are *frequently mutated in AML* and the *Drosophila melanogaster orthologues.***

| Components of PRC1 and PRC2 complex | Fly orthologue | Function | References |
|---|---|---|---|
| lysine demethylase 2A (KDM2A) | Lysine demethylase 2 (KDM2) | Histone demethylase that specifically demethylates 'Lys-36' of histone H3 | PMID: 30059280 |
| Chromobox Homolog 2 (CBX2) | Pc | Chromatin binding protein that interacts with histone H3K27me3. It is involved in epigenetic silencing | PMID: 25367972 |
| Enhancer of zeste 2 (EZH2) polycomb repressive complex 2 subunit | Enhancer of zeste Ez | Catalytic component of the Polycomb Repressive Complex 2 (PRC2) methyltransferase that methylates histone H3 lysine27. | PMID: 19026780 |
| Jumonji and AT-rich interaction domain containing 2 (JARID2) | Jarid 2 Jumonji, AT rich interactive domain 2 | Nuclear protein that functions as a DNA-binding transcriptional repressor. It functions as a scaffold for the recruitment of various complexes, including Polycomb repressive complex 2. It binds mono-ubiquitylated H2A lysine 119 to mediate crosstalk between Polycomb complexes PRC1 and PRC2 | PMID: 27892467 |
| Polycomb repressive complex 2 subunit (SUZ12) | Suppressor of zeste 12 Su(z)12 | Subunit of Polycomb repressive complex 2 (PRC2), where it is required for the histone methyltransferase activity that yields trimethylation of histone H3 on lysine 27 (H3-K27me3) | PMID: 15225548 |

**Table S5. Table summarizing highly conserved human genes of PRC1 and PRC2 components and the *Drosophila melanogaster* *orthologues*.**
